# RBM15 promotes hepatocellular carcinoma progression by regulating N6-methyladenosine modification of YES1 mRNA in an IGF2BP1-dependent manner

**DOI:** 10.1038/s41420-021-00703-w

**Published:** 2021-10-27

**Authors:** Xianlei Cai, Yunhao Chen, Da Man, Beng Yang, Xiaode Feng, Deguo Zhang, Junru Chen, Jian Wu

**Affiliations:** 1 Division of Hepatobiliary and Pancreatic Surgery, Department of Surgery, First Affiliated Hospital, School of Medicine, Zhejiang University, 310003 Hangzhou, China; 2 Department of General Surgery, Ningbo Medical Center Lihuili Hospital, 315000 Ningbo, China; 3 NHC Key Laboratory of Combined Multi-organ Transplantation, 310003 Hangzhou, China; 4 Key Laboratory of the Diagnosis and Treatment of Organ Transplantation, CAMS, 310003 Hangzhou, China; 5 Key Laboratory of Organ Transplantation, 310003 Hangzhou, China; 6 Zhejiang Provincial Research Center for Diagnosis and Treatment of Hepatobiliary Diseases, 310003 Hangzhou, China

**Keywords:** Oncogenes, Methylation

## Abstract

The function of the N6-methyladenosine (m^6^A) methyltransferase RNA-binding motif protein 15 (RBM15) in hepatocellular carcinoma (HCC) has not been thoroughly investigated. Here we determined the clinical value, biological functions, and potential mechanisms of RBM15 in HCC. Expression of RBM15 was identified using tissue microarrays and online databases. A risk-prediction model based on RBM15 was developed and validated. We determined the biological role of RBM15 on HCC cells in vitro and in vivo. RNA sequencing was used to screen candidate targets of RBM15. Subsequently, the m^6^A dot blot assay, methylated RNA immunoprecipitation qPCR, dual-luciferase reporter assays, RNA decay assay, and RNA immunoprecipitation qPCR were employed to explore the mechanisms of RBM15. Our study showed that RBM15 was highly expressed in HCC, and overexpression of RBM15 indicated a worse outcome. A new nomogram combining RBM15 with age and TNM stage was developed and validated to predict the outcome of HCC patients; our nomogram increased the prediction accuracy of the TNM system. Functionally, RBM15 facilitates the proliferation and invasiveness of HCC. RBM15-mediated m^6^A modification contributed to a post-transcriptional activation of YES proto-oncogene 1 (YES1) in an insulin-like growth factor 2 mRNA-binding protein 1 (IGF2BP1)-dependent manner. In addition, YES1 was confirmed as an oncogene in HCC cells by activating the mitogen-activated protein kinase (MAPK) pathway. In conclusion, RBM15-mediated m^6^A modification might facilitate the progression of HCC via the IGF2BP1–YES1–MAPK axis. RBM15 may be a promising biomarker in the outcome prediction of HCC.

## Introduction

Hepatocellular carcinoma (HCC) is one of the most common digestive system tumors, characterized by high malignancy, morbidity, and mortality. Liver cancer is among the four cancers with the highest mortality rates, accounting for 8.2% of total cancer deaths [1]. The recurrence and metastasis rates of HCC are high, and the outcomes of HCC patients are poor, especially in advanced diseases [2]. In recent years, significant progress has been made in the treatment of HCC, from single surgery to comprehensive treatment, such as interventional therapy, targeted therapy, and immunotherapy; nevertheless, the outcome of HCC remains unsatisfactory [3–7]. Therefore, it is necessary to clarify the pathogenesis of HCC, to develop new treatment strategies, and to improve the prognosis of patients with HCC.

Much evidence showed that disorders of epigenetic regulation are important causes of HCC. Previous studies focused on transcription levels [8, 9]; however, RNA methylation modification has attracted increasing attention. Among them, N6-methyladenosine (m^6^A) is a necessary modification of mammalian messenger RNA (mRNA). The functions of m^6^A modification include dynamic interactions of methyltransferases (writers), demethylases (erasers), and effector proteins (readers) [10, 11]. The classical methyltransferase complex consists of methyltransferase-like 3 (METTL3), methyltransferase-like 14 (METTL14), and Wilms tumor-associated protein (WTAP) [12]. Several proteins have been found to regulate methylation modification in recent years, including METTL16 [13], ZC3H13 [14], and RNA-binding motif protein 15 (RBM15) [15]. m^6^A modification is involved in many biological processes. A variety of methyltransferases (including METTL3, METTL14, FTO, and WTAP) facilitated tumor progression in acute leukemia [16], prostate cancer [17], gastric cancer [18], and colorectal cancer [19]. In HCC, METTL3 was found to enhance m^6^A methylation modification of SOCS2 in a YTHDF2-dependent manner [20]. In our previous studies, WTAP silenced ETS1 in a HuR-dependent manner, promoting the growth of HCC [10]; ALKBH5 suppressed the malignancy of HCC via m^6^A-guided epigenetic modification of LYPD1 [21].

RBM15 is a member of the split-end protein family [22]. It is an essential regulator of RNA m^6^A methylation modification and a critical component of the methyltransferase complex that participates in homeostasis of hematopoietic cells, alternative splicing of mRNAs [23], and X chromosome inactivation of Xist-RNA [15]. RBM15 regulated m^6^A modification by binding target RNA and recruiting methyltransferase complexes [15]. Investigators found that RBM15 mutation was related to the occurrence and recurrence of phyllodes tumors [24]. Wang et al. found that RBM15-mediated m^6^A modification of TMBIM6 promoted laryngeal squamous cell carcinoma [25]. Nevertheless, the potential roles of RBM15 in HCC remain unclear.

In our present study, we revealed the clinical values and biological functions of RBM15 in HCC and proposed that RBM15 may be a novel biomarker and therapeutic target for HCC treatment.

## Results

### High expression of RBM15 is associated with poor outcomes of HCC

**Fig. 1:**
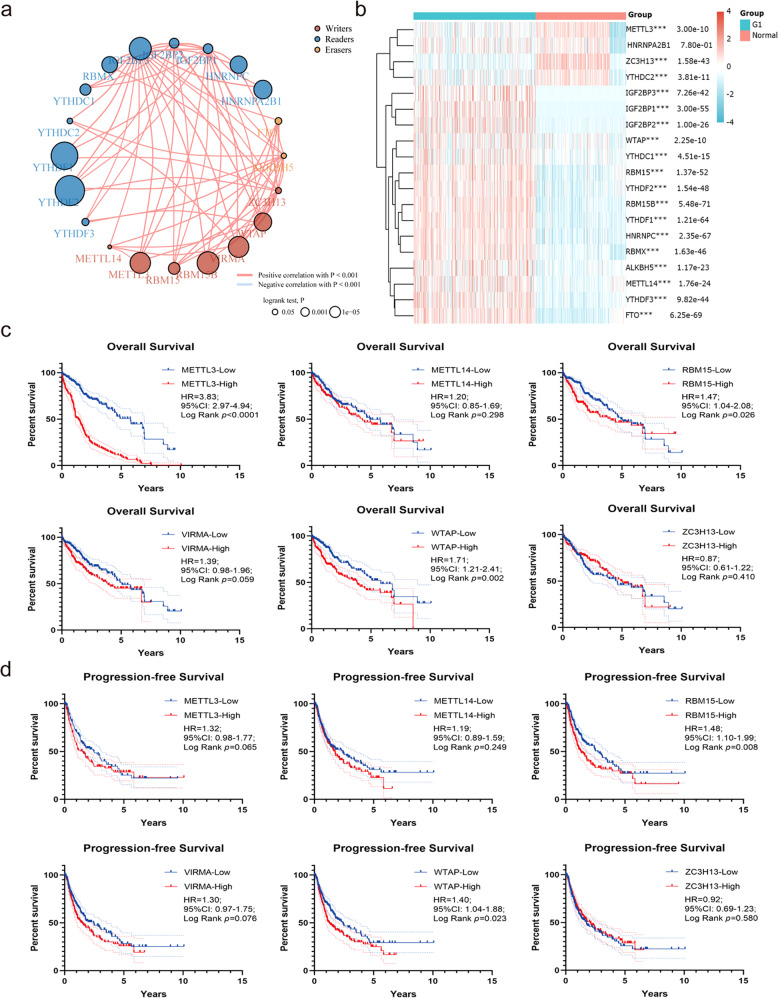
Bioinformatics analyses of m6A modification-related genes in HCC. **a** Inter-relation between m6A “writers,” “readers,” and “erasers”; **b** heatmap of m6A modification-related genes based on TCGA and GTEx data (**a**, **b** were drawn online by ACLBI, http://www.aclbi.com/static/index.html#/); **c** Kaplan–Meier analyses of overall survival of TCGA data based on different “writers” expression; **d** Kaplan–Meier analyses of progression-free survival of TCGA data based on different “writers” expression.

To determine the clinical value of m^6^A modification-related enzymes in HCC, we analyzed the inter-relation between “writers,” “readers,” and “erasers” (Fig. 1a) and the expression of various enzymes between tumors and normal tissues (Fig. 1b). We generated Kaplan–Meier curves for overall survival (OS) (Fig. 1c) and progression-free survival (PFS) (Fig. 1d) for the most common methyltransferases. We found that only WTAP and RBM15 were both associated with OS and PFS in HCC. The biological role of WTAP was described in our previous study [10], while RBM15 was unexplored. Therefore, we paid close attention to RBM15.

**Fig. 2:**
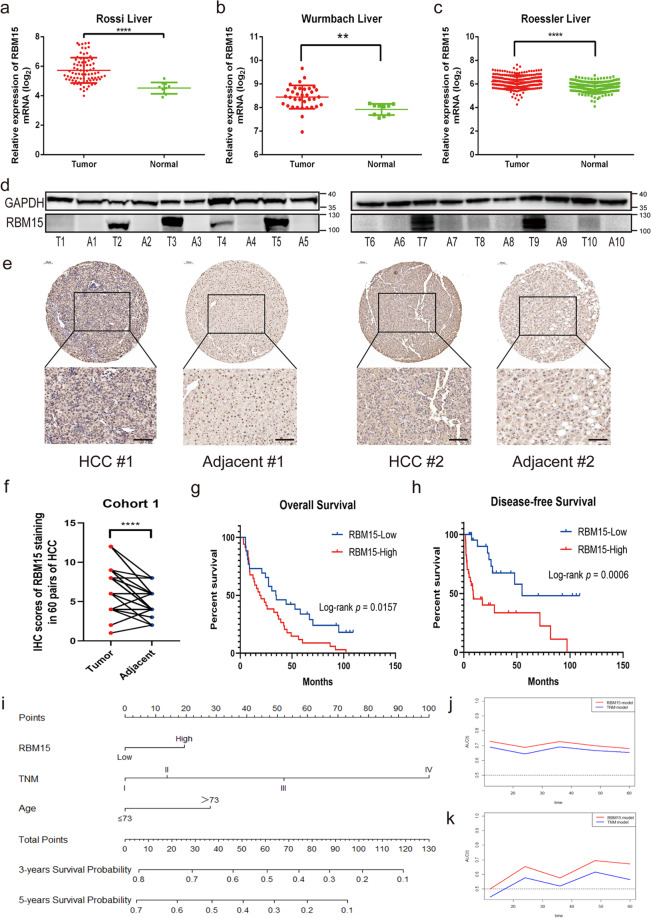
High-expression of RBM15 is associated with poor outcomes of HCC. **a**–**c** The expression of RBM15 mRNA was compared between tumors and normal tissues based on GEO datasets (GSE14520, GSE62232, and GSE6764; **p < 0.01, ****p < 0.0001; *t* test); **d** the expression of RBM15 protein was analyzed in ten pairs of HCC tissue (T: tumor; A: adjacent tissue); **e** representative IHC images of RBM15 staining in tumor and adjacent tissues of cohort-1 patients (scale bar, 100 μm; magnifications, ×100 and ×200); **f** IHC scores of matched tumors and adjacent tissues of cohort-1 were calculated based on RBM15 staining (****p < 0.0001; *t* test); **g** Kaplan–Meier analysis of overall survival of cohort-1 patients based on RBM15 expression; **h** Kaplan–Meier analysis of disease-free survival of cohort-1 patients based on RBM15 expression; **i** nomogram to predict 3- and 5-year overall survival. Each risk factor corresponded to a point by drawing a line straight upward to the points axis. The sum of the points located on the total points axis showed the probability of overall survival by drawing a line straight down to the survival axis. **j** The time-dependent ROC curves comparing the RBM15 model with the TNM model based on the training group (cohort-2); **k** the time-dependent ROC curves comparing the RBM15 model with the TNM model based on the validation group (cohort-1). The data are presented as mean ± SD.

We measured mRNA and protein levels of RBM15 in HCC and found that RBM15 was significantly upregulated in HCC (Fig. 2a–d). Immunohistochemical (IHC) staining with tissue microarray (TMA) from cohort-1 confirmed the results (Fig. 2e, f). Analysis of the TMA cohort revealed that RBM15 expression was associated with alpha-fetoprotein and patients with high RBM15 expression had worse OS and disease-free survival (DFS) (Table 1 and Fig. 2g, h). RBM15 was an independent risk factor for OS (hazard ratio (HR) = 2.06; 95% confidence interval (CI): 1.16–3.68) and DFS (HR = 3.57; 95% CI: 1.57–8.12) (Supplementary Table 5).

**Table 1:** Clinical characteristics of cohort-1 depending on the RBM15 expression levels.

Factor	RBM15-low	RBM15-high	*n*	*χ*^2^	*p*
All case	26	34	60		
Age				0.331	0.565
≤60	19	27	46		
>60	7	7	14		
AJCC stage				0.087	0.768
Stage I–II	12	17	29		
Stage III–IV	14	17	31		
Tumor number				2.527	0.112
Single	19	18	37		
Multiple	7	16	23		
Tumor size				0.432	0.555
≤5 cm	5	9	14		
>5 cm	21	25	46		
Tumor encapsulation				2.986	0.117
Intact	11	22	33		
Broken	15	12	27		
Microvascular infiltration				0.160	0.689
Yes	5	8	13		
No	21	26	47		
HbsAg				1.750	0.307
Negative	3	1	4		
Positive	23	33	56		
Cirrhosis				0.271	0.602
Yes	20	28	48		
No	6	6	12		
AFP				4.103	0.043
≤400 μg/L	19	16	35		
>400 μg/L	7	18	25		

*AFP* alpha-fetoprotein.

*p* value was measured by Chi-Square test or Fisher’s exact test.

### RBM15-associated nomogram increased prediction accuracy of the tumor, node, metastasis (TNM) system

The univariate and multivariate Cox analysis results showed that RBM15 was also a significant independent risk factor (HR = 1.49; 95% CI: 1.01–2.21) with age and TNM stage (Table 2). Based on these results, we developed a prediction model combining RBM15, age, and TNM stage and generated a graphical nomogram (Fig. 2i). To determine the clinical value of RBM15, a head-to-head comparison model depending on the TNM stage was also developed. We found that the predictive accuracy of the RBM15 model calculated by area under the curve was higher than the TNM model (Fig. S1a, b), confirmed by the time-dependent receiver operating characteristic (ROC) curve (Fig. 2j). Cohort-1 was used to validate the prediction models, revealing similar results in protein stratification (Fig. 2k). All findings indicate that RBM15 is a biomarker to predict the outcome of HCC and had a good value for translational clinics.

**Table 2:** Univariate and multivariate Cox analysis of cohort-2 (training cohort) for overall survival.

Factors	Univariate analysis			Multivariate analysis		
	HR	95% CI	*p*	HR	95% CI	*p*
Gender						
Female	1		0.175			
Male	0.77	0.52–1.13				
Age						
≤73	1		0.051	1		0.043
>73	1.62	1.00–2.64		1.49	1.01–2.21	
Histologic grade						
G1	1		0.686			
G2	1.21	0.66–2.21				
G3	1.21	0.64–2.28				
G4	1.91	0.68–5.38				
TNM stage						
I stage	1		0.000	1		0.000
II stage	1.49	0.89–2.50		1.33	0.79–2.24	
III stage	3.06	1.98–4.73		2.95	1.90–4.56	
IV stage	6.44	1.97–20.99		7.84	2.38–25.86	
Family history of HCC						
No	1		0.617			
Yes	1.11	0.74–1.67				
RBM15						
Low	1		0.044	1		0.043
High	1.47	1.01–2.15	2.15	1.49	1.01–2.21	

*HR* hazard ratio, *CI* confidence interval.

*p* value was measured by Cox proportional hazard regressions.

### RBM15 promotes the growth and migration/invasion capability of HCC cells in vitro

**Fig. 3:**
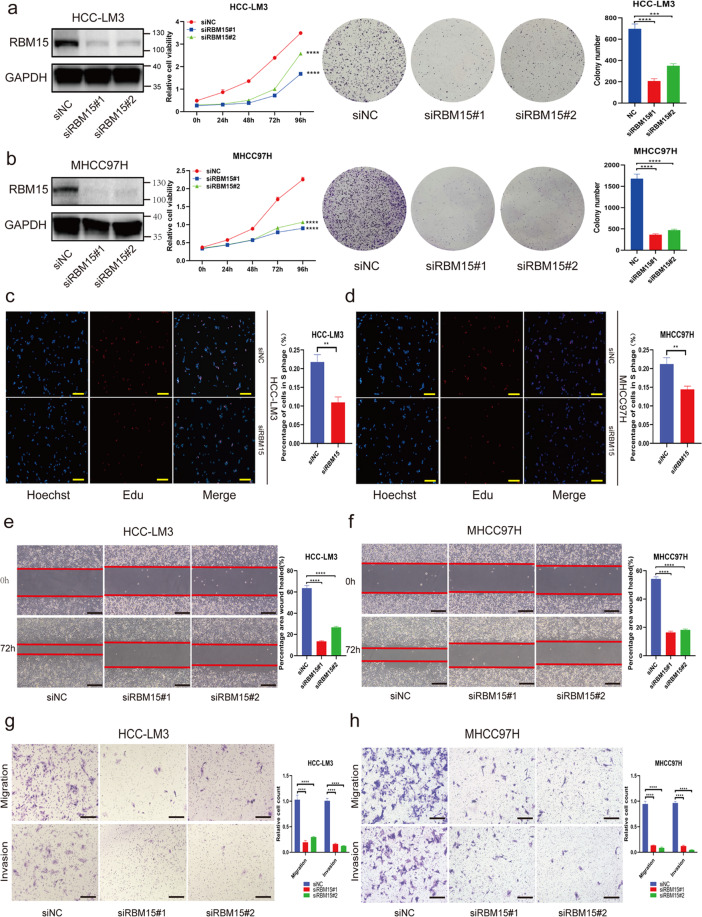
RBM15 promotes tumor growth and migration/invasion capability of HCC cells in vitro. **a**, **b** Negative control or siRNA (si-RBM15#1 and #2) was transfected into HCC-LM3 (**a**) and MHCC97H (**b**), respectively. The efficiency of knockdown was tested by western blotting and the proliferation capacities of HCC cells were detected by CCK-8 and colony-formation assays (***p < 0.001, ****p < 0.0001; two-way ANOVA and *t* test); **c**, **d** EdU assays were applied to compare the proliferation abilities of HCC-LM3 (**c**) and MHCC97H cells (**d**) (scale bar, 200 μm); bar charts showed the percentage of cells in S phage based on the results of EdU assays (**p < 0.01; *t* test); **e**, **f** wound-healing assays were performed to compared the migration capabilities of HCC-LM3 (**e**) and MHCC97H (**f**) cells (scale bars, 200 μm); the percentage of healed area were quantified by bar charts (****p < 0.0001; *t* test); **g**, **h** transwell assays were applied to detect the migration and invasion abilities of HCC-LM3 (**g**) and MHCC97H (**h**) cells after silencing RBM15 (scale bars, 200 μm); bar charts showed the relative count of two groups of HCC cells, which passed through the chamber membranes when referred to negative control groups (****p < 0.0001; *t* test); The data are presented as mean ± SD.

To investigate the biological functions of RBM15 in HCC, Huh7, HCC-LM3, and MHCC97H were chosen to establish RBM15-knockdown models. The transfection efficiency was validated using western blotting (Fig. 3a, b) and quantitative polymerase chain reaction (qPCR; Fig. S2a). We found that knockdown of RBM15 inhibited the proliferation capability of HCC cells according to Cell Counting Kit-8 (CCK-8) and colony-formation assays (Figs. 3a, b and S3a), and 5-ethynyl-2′-deoxyuridine (EdU) assay also confirmed that RBM15 promoted cell growth in vitro (Figs. 3c, d and S3b).

To explore the effects of RBM15 on migration and invasion, we performed a wound-healing assay and observed that RBM15 tended to accelerate the migration of HCC cells (Figs. 3e, f and S3c). The transwell assays demonstrated that RBM15 deficiency inhibited the migration and invasion abilities of HCC cells (Figs. 3g, h and S3d).

### RBM15 modulates the mitogen-activated protein kinase (MAPK) pathway via the candidate target YES proto-oncogene 1 (YES1)

**Fig. 4:**
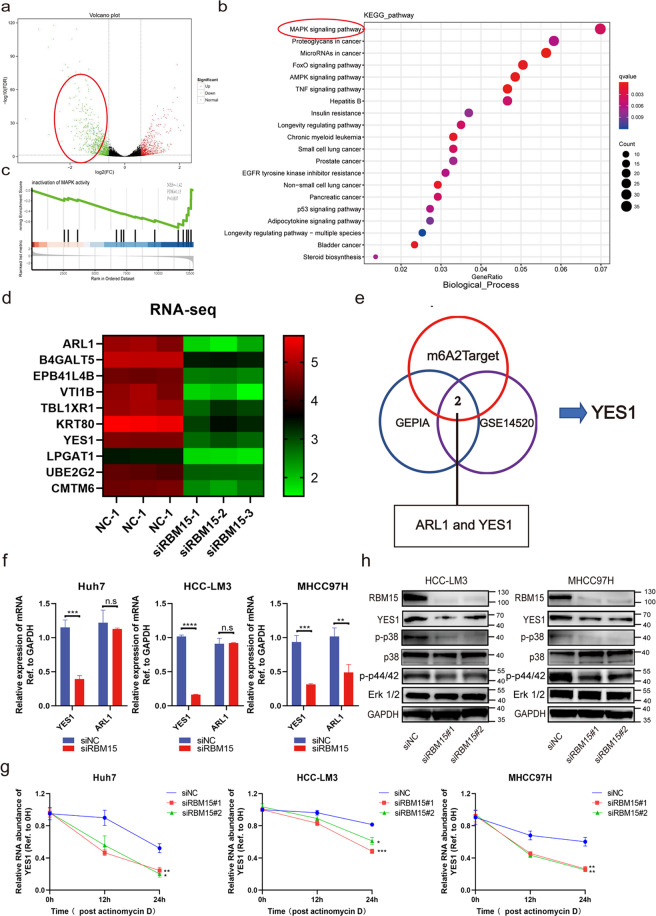
RBM15 modulates the MAPK pathway via the candidate target YES1. **a** Volcano plot of RNA-sequencing showed the distribution of differentially expressed genes. In order to investigate the carcinogenicity of RBM15, we focused on the downregulated genes when RBM15 was silenced (marked with red circle); **b** KEGG pathway enrichment analysis of downregulated DEGs indicated that RBM15 may promote HCC tumorigenesis via the MAPK signaling; **c** GSEA analysis showed that downregulated DEGs were enriched in the inactivation of MAPK activity; **d** heatmap of the top ten downregulated genes that were closely associated with RBM15; **e** a Venn diagram was generated for these ten genes to verify the expression levels in GEPIA database and GSE14520 and to predict potential m6A modification of these candidates in m6A2Target database. Finally, two genes (ARL1 and YES1) were selected according to the overlaps; **f** RT-qPCR was conducted in Huh7, HCC-LM3, and MHCC97H cells with RBM15 silencing to validate the overlapped genes (**p < 0.01, ***p < 0.001, ****p < 0.0001; *t* test); **g** the RNA decay assays were performed in Huh7, HCC-LM3, and MHCC97H cells after treatment with Actinomycin D (*p < 0.05, **p < 0.01, ***p < 0.001; two-way ANOVA); **h** expression of YES1 and downstream hub proteins in the MAPK pathway were evaluated by western blotting when RBM15 was silenced in HCC-LM3 and MHCC97H cells; the data are presented as mean ± SD.

To investigate the mechanism mediating the observed RBM15-dependent phenotypes, RNA sequencing (RNA-seq) was employed using RBM15-knockdown and negative control HCC-LM3 cells. A total of 659 upregulated genes and 861 downregulated genes were identified (Fig. 4a). Finally, we focused on the 861 downregulated genes associated with RBM15 (Supplementary Table 6). The gene ontology (GO) analyses for the differentially expressed genes (DEGs) were conducted (Fig. S4a–c). Kyoto Encyclopedia of Genes and Genomes (KEGG) pathway enrichment analysis of downregulated DEGs indicated that RMB15 might promote HCC tumorigenesis via the MAPK signaling pathway (Fig. 4b); Gene Set Enrichment Analysis (GSEA) analysis also showed a similar result (Fig. 4c). The top ten genes associated with RBM15 are listed (Fig. 4d).

A Venn diagram was generated for these ten genes to verify high expression levels in the GEPIA database and GSE14520 and predict potential m^6^A modification of candidate targets in the m6A2Target database (Fig. 4e). Finally, two candidate genes (ARL1 and YES1) were selected according to the overlaps. We found that silencing of RBM15 decreased the expression of YES1, and ARL1 did not achieve such significant variation (Fig. 4f). The stability of YES1 mRNA was decreased by the RBM15 knockdown (Fig. 4g). Furthermore, western blotting confirmed that loss of RBM15 attenuated the expression of YES1 protein and inactivated the MAPK pathway (Fig. 4h).

### RBM15-mediated m^6^A modification strengthens the stability of YES1 mRNA in an IGF2BP1-dependent manner

**Fig. 5:**
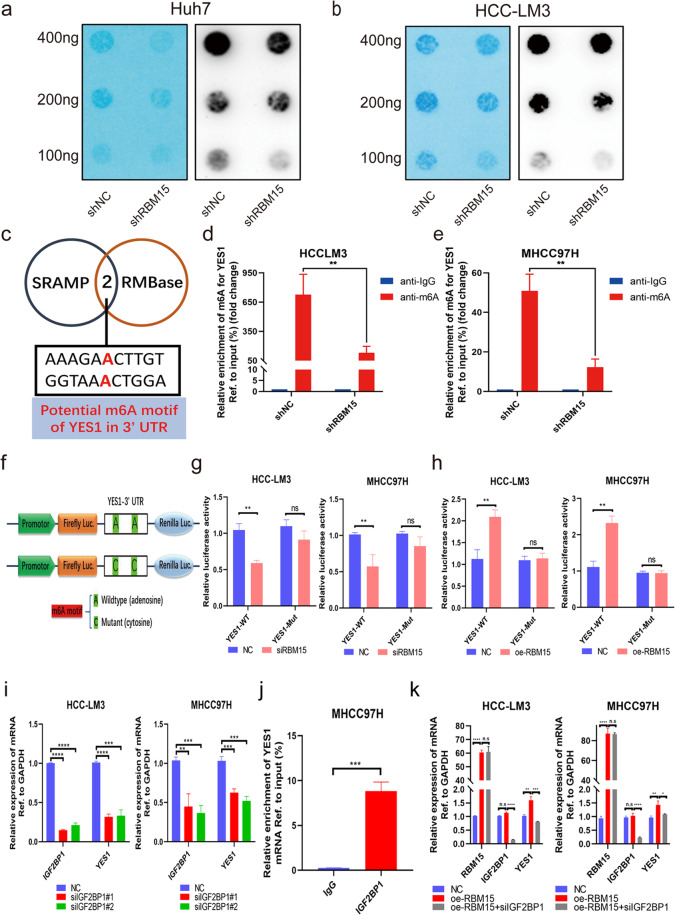
RBM15 regulates YES1 in an IGF2BP1-m^6^A-dependent pattern. **a**, **b** Global m^6^A level of RNA extracted from RBM15-knockdown Huh7 and HCC-LM3 cells was investigated by m^6^A dot blot assays. The intensity of dot immunoblotting indicated the m^6^A level of total RNAs, while methylene blue staining was applied to measured input RNA; **c** a Venn diagram was generated for predicting potential m^6^A motif of YES1 in 3’ UTR from SRAMP and RMBase databases. Two candidate m^6^A motifs were selected; **d**, **e** showed m^6^A modification of YES1 by MeRIP-qPCR analyses in HCC-LM3 and MHCC97H cells. Knockdown of RBM15 induced a decreased m^6^A abundance on YES1 compared with the control group (**p < 0.01; *t* test); **f** construction pattern of luciferase reporters. The wild-type or mutant type (m^6^A motif mutated) sequence of YES1 3’ UTR was inserted into a pcDNA 3.1 vector, which contained Firefly and Renilla elements; **g** relative luciferase activity of the wild-type or mutant group was determined in RBM15-silenced HCC-LM3 and MHCC97H cells (normalized to Renilla activity; **p < 0.01; *t* test); **h** relative luciferase activity of the wild-type or mutant group was determined in RBM15 overexpression HCC-LM3 and MHCC97H cells (normalized to Renilla activity; **p < 0.01; *t* test); **i** YES1 mRNA expression was decreased after knockdown of IGF2BP1 in HCC-LM3 and MHCC97H cells (**p < 0.01, ***p < 0.001, ****p < 0.0001; *t* test). **j** RIP-qPCR validated that YES1 mRNA could bind to IGF2BP1 protein (***p < 0.001; *t* test); **k** the upregulation YES1 mRNA induced by RBM15 overexpression was reduced by knockdown of IGF2BP1 (*p < 0.05, **p < 0.01, ***p < 0.001, ****p < 0.0001; *t* test). The data are presented as mean ± SD.

Dot blot assays were used to evaluate the function of RMB15 in regulating m^6^A modification. Knockdown of RBM15 led to a significant decrease in m^6^A levels in Huh7 and HCC-LM3 cells (Fig. 5a, b). SRAMP and RMBase databases were used to predict potential m^6^A motifs of YES1 in the 3’ untranslated region (Fig. 5c). Then methylated RNA immunoprecipitation (MeRIP)-qPCR assays indicated that knockdown of RBM15 decreased m^6^A levels in HCC cells (Fig. 5d, e). We performed luciferase reporter assays to illuminate the essential role of m^6^A modification on YES1 mRNA (Fig. 5f). As expected, cells transfected with YES1 wild-type plasmid had lower luciferase activity when RBM15 was silenced, while cells transfected with YES1-mutant plasmid with m^6^A motif mutation remained unaffected (Fig. 5g); In contrast, when RBM15 was overexpressed, cells transfected with YES1 wild-type plasmid had higher luciferase activity (Fig. 5h).

Moreover, we investigated potential “readers” that would be responsible on m^6^A-modified transcripts. Using m6a2target database, IGF2BP1, IGF2BP3, and YTHDF1 were found as candidates of “readers” (data not shown). These three candidates were upregulated in HCC tissues (Fig. S5a–c), and they were silenced in HCC cells to evaluate the alterations of YES1 expression. We observed that YES1 was significantly attenuated when IGF2BP1 was silenced, consistent with the notion that IGF2BP1 was involved in stabilizing target mRNA [26] (Fig. 5i). Interestingly, the expression of YES1 was found to be positively correlated with IGF2BP1 (Fig. S5d); high expression of IGF2BP1 also indicated a worse outcome (Fig. S5e). Nevertheless, knockdown of IGF2BP3 and YTHDF1 did not achieve such significant effects (Fig. S5f). The result of the RIP assay validated that IGF2BP1 protein could physically bind to YES1 mRNA (Fig. 5j). Moreover, the upregulation YES1 induced by RBM15 overexpression was rescued by knockdown of IGF2BP1 (Fig. 5k). To summarize, YES1 was regulated by RBM15-mediated m^6^A modification followed by the recognition and stabilization of IGF2BP1.

### YES1 is an oncogenic driver in HCC cells in vitro

**Fig. 6:**
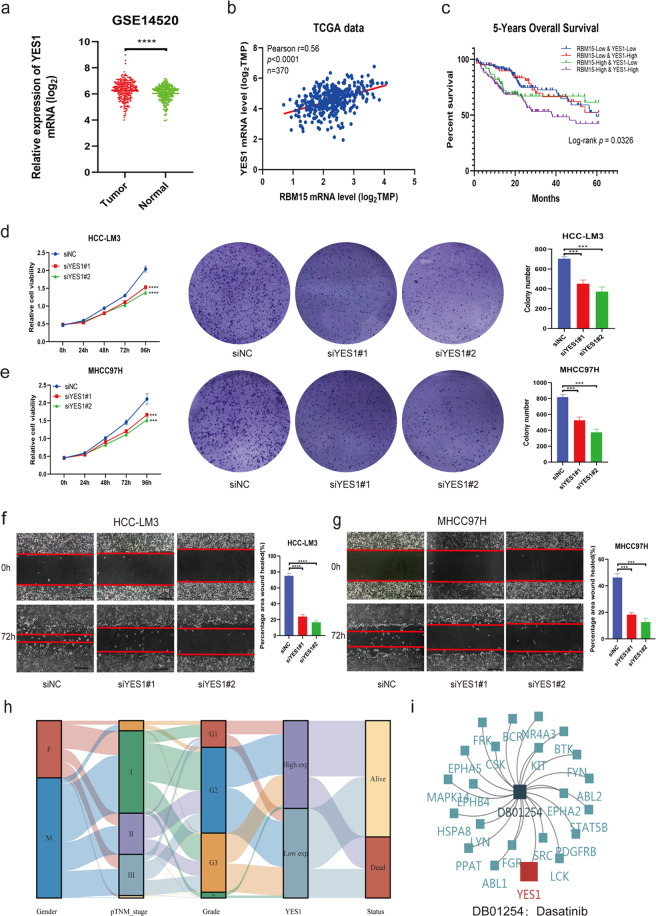
YES1 promotes tumor growth of HCC cell in vitro. **a** The expression of YES1 mRNA was compared between tumors and normal tissues based on GEO database (GSE14520) (****p < 0.0001; *t* test); **b** TCGA data analysis showed a positive correlation of RBM15 and YES1 based on mRNA expression; **c** overall survival analysis according to the co-expression of RBM15 and YES1 in TCGA data. Both high expression of RBM15 and YES1 indicated the worst prognosis; **d**, **e** negative control or siRNA (si-YES1#1 and #2) was transfected into HCC-LM3 (**d**) and MHCC97H (**e**), respectively. The proliferation capacities of HCC cells were detected by CCK-8 and colony-formation assays. Colony numbers were quantified by bar charts (***p < 0.001, ****p < 0.0001; two-way ANOVA and *t* test); **f**, **g** wound-healing assays were performed to compare the migration capabilities of HCC-LM3 (**f**) and MHCC97H (**g**) cells (scale bars, 200 μm). The percentage of healed area was quantified by bar charts (***p < 0.001, ****p < 0.0001; *t* test); **h** Sankey diagram showed the inter-relation between the YES1 expression and other clinical characteristics; **i** dasatinib was a potential drug for patients with YES1 overexpression or other relevant targets. The data are presented as mean ± SD.

To validate the oncogenic role of YES1 in HCC, we compared the mRNA levels of YES1 between tumor and normal tissues and analyzed the co-expression between YES1 and RBM15. We found that YES1 was overexpressed in HCC (Fig. 6a) and positively correlated with RBM15 (Fig. 6b). High expression levels of both RBM15 and YES1 indicated the worst outcomes (Fig. 6c). CCK-8 and colony-formation assays showed that knockdown of YES1 suppressed HCC cell growth and viability (Figs. 6d, e and S2b). Wound-healing assays indicated that silencing of YES1 inhibited the migration of HCC cells (Fig. 6f, g).

To demonstrate applicability to clinical practice, a Sankey diagram was drawn to reveal the inter-relations between the YES1 expression and other clinical characteristics (Fig. 6h). The targeted drug (dasatinib) had potential effects on HCC patients with YES1 overexpression (Fig. 6i). Bioinformatics analyses indicated that YES1 was associated with immune infiltration, including the infiltration of B cell, CD8+ T cell, CD4+ T cell, macrophage, neutrophil, and dendritic cell (Fig. S6a–f). Taken together, these findings suggest that YES1 is an oncogenic driver in HCC.

### The effects of RBM15 silencing are rescued by overexpression of YES1

**Fig. 7:**
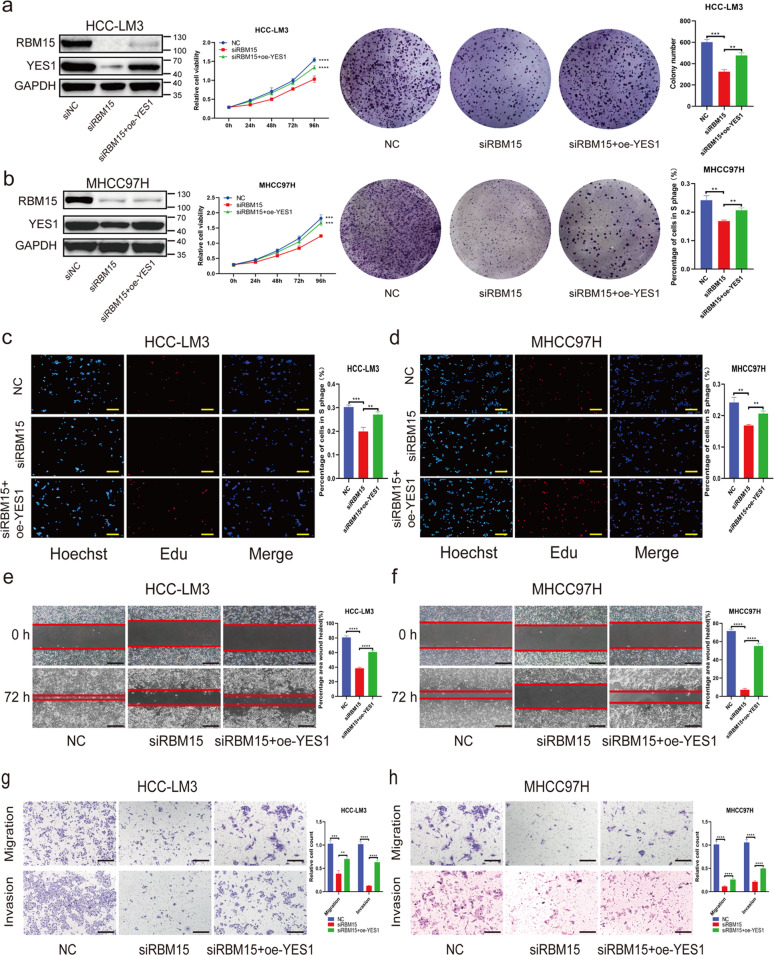
Overexpression of YES1 could rescue the phenotype induced by RBM15 knockdown. **a**–**d** Rescue experiments (CCK8, colony-formation assays, and EdU assays) were performed to test the influence of YES1 overexpression on growth of RBM15-knockdown HCC cells (**p < 0.01, ***p < 0.001, ****p < 0.0001; two-way ANOVA and *t* test); **e**–**h** rescue experiments (wound-healing assays and transwell assays) were performed to test the influence of YES1 overexpression on migration/invasion ability of RBM15-knockdown HCC cells (**p < 0.01, ***p < 0.001, ****p < 0.0001; *t* test; scale bars, 200 μm). The data are presented as mean ± SD.

To confirm the influence of the RBM15–YES1 axis on the observed phenotypes, we performed several functional rescue assays. We found that silencing of RBM15 resulted in decreased proliferation ability of HCC cells, which could be reverted by YES1 overexpression (Fig. 7a–d). Overexpression of YES1 also rescued the decreased migration and invasion capacity of HCC cells (Fig. 7e–h). Taken together, these findings suggest that dysregulation of the RBM15–YES1 axis accounts for the proliferation or mobility of HCC cells.

### RBM15 depletion suppresses the growth of HCC cells in vivo

**Fig. 8:**
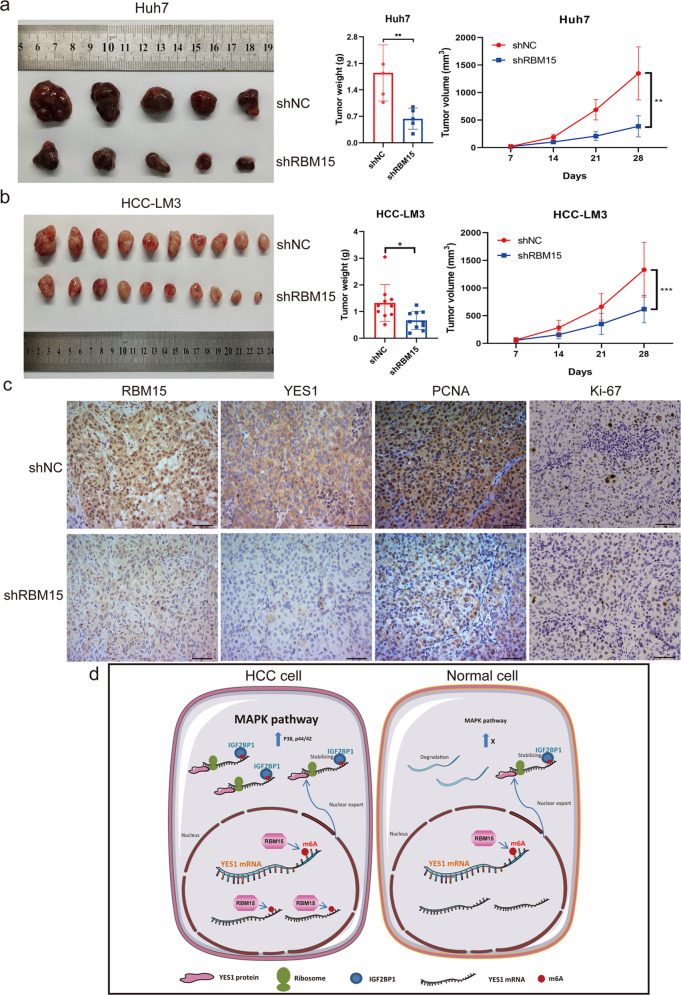
RBM15 promotes tumor growth of HCC cells in vivo. **a**, **b** Tumor xenograft models were constructed with stable RMB15-knockdown Huh7 cells (*n* = 5) and HCC-LM3 cells (*n* = 10). Tumor sizes were recorded per week consecutively to plot tumor growth curves. After collecting tumors from sacrificed mice, tumor weights were measured and compared between shRBM15 and shNC cells (*p < 0.05, **p < 0.01, ***p < 0.001; two-way ANOVA and *t* test); **c** representative IHC staining images of xenograft tumors. Knockdown of RBM15 decreased the expression of YES1, PCNA, and Ki-67 (scale bars, 200 μm); the data are presented as mean ± SD; **d** abstract graph illustrated our findings on RBM15-mediated m^6^A regulation. The data are presented as mean ± SD.

To explore the contribution of RBM15 to HCC growth in vivo, we performed in vivo experiments in athymic nude mice with RBM15 stably depleted Huh7 and HCC-LM3 cells (Fig. S2c). We found that volumes and weights of xenografted tumors were significantly lower after the knockdown of RBM15 than the negative controls (Fig. 8a, b). IHC of xenograft tumors revealed that the silencing of RBM15 attenuated the expression of YES1 and decreased the expression of PCNA and Ki-67, which are biomarkers of proliferation (Fig. 8c). In summary, these findings suggest that RBM15 promotes tumor growth in vivo, consistent with in vitro results.

## Discussion

m^6^A modification is an area of intense interest in oncology research. Based on clinical data, we explored the methyltransferases that predicted the outcomes in patients with HCC best. Only RBM15 and WTAP significantly correlated with OS and PFS in HCC patients. Because our team previously described the role of WTAP in HCC in detail, the present work mainly focused on RBM15. After constructing the prognosis prediction model, we found that RBM15 combined with clinical and pathological features better predicted outcomes of HCC. The data in the validation group confirmed these results at the protein level, suggesting that RBM15 might be used as a biomarker in the outcome prediction of HCC.

In clinical practice, clinicians mainly use the TNM stage to predict outcomes. Several studies found that gene information combined with clinical features could better predict outcomes [27]. In the present study, a prediction model with RBM15 combined with TNM stage and age was constructed. Compared with the TNM stage alone, the new model predicted outcomes of HCC more accurately. For example, a 60-year-old patient (0 points) with high RBM15 expression (about 20 points) and TNM stage III (about 52 points) has a 3-year OS rate of about 58% and a 5-year OS rate of about 36%. The development and validation of the clinical prediction model is a highlight of this study, which increases the clinical value and the possibility for clinical application of RBM15.

Reviewing the literature, we found that the biological functions of methyltransferases such as METTL3 [20], METTL14 [28], and VIRMA (Vir Like M6A Methyltransferase Associated) [29] in HCC have been reported. However, there is a gap in the research of RBM15 in the field of HCC, and there remain many ambiguities about the regulation mode of m^6^A; therefore, the mechanisms remain to be further explored. Our experiments showed that RBM15 promotes the progression of HCC in vitro and in vivo. Inhibition of RBM15 weakens the proliferation and invasion capability of HCC cells and the m^6^A methylation level of HCC cells as a whole, suggesting that RBM15 plays an “m^6^A writer” role in HCC.

While exploring downstream molecules using RNA-seq and pathway enrichment, the star gene YES1 was identified. YES1 is a Src-family protein kinase (SFKs) member, a family of non-receptor tyrosine kinases. SFKs are involved in a variety of carcinogenesis-related signal pathways [30]. YES1 is a well-known proto-oncogene that participates in the growth, differentiation, and invasion of cancers, such as colon carcinoma [31], rhabdomyosarcoma [32], esophageal cancer [33] and others. Pang et al. [34] found that, in HCC, LINC00998-encoded peptides mediated membrane anchoring, activation of YES1, and carcinogenesis through the MAPK pathway. The present study showed that m^6^A modification was also one of the regulatory mechanisms of YES1. The RNA-seq results converged the YES1 and MAPK pathways, and we simply validated the results of Pang et al. [34] using in vitro experiments. In addition to the MAPK pathway, YES1 has been found to regulate YAP1 and play a role through the Hippo pathway [33]. SFL/multikinase inhibitors such as dasatinib and bosutinib are used in current clinical practice; however, they are primarily used to treat chronic myeloid leukemia with positive Philadelphia chromosomes and are seldom used to treat solid tumors. Selective inhibitors of SFKs have not yet been marketed. Hamanaka et al. [33] synthesized a novel YES1 inhibitor (CH695755) that inhibited the progression of YES1-amplified cancers. Using bioinformatics analysis, the present study showed that dasatinib might have therapeutic potential in HCC, and YES1 expression was positively associated with immune infiltration, suggesting that YES1 inhibitor combined with immunotherapy might have a synergistic function in patients with HCC. This cautious speculation needed further study.

“Readers” functions are also indispensable in m^6^A modification. Through database matching, three “readers” (IGF2BP1, IGF2BP3, and YTHDF1) were identified. Expression of IGF2BP1 positively correlated with YES1, and knockdown of IGF2BP1 reduced YES1 expression levels. The RIP assay confirmed the direct binding between IGF2BP1 protein and YES1 mRNA. The results of these assays indicated that IGF2BP1 was a bridge connecting RBM15, YES1, and MAPK signaling.

There are still several limitations to our study. First, we used small interfering RNA to perform phenotype experiments. Although there are many studies supporting this approach, it is better to repeat phenotype experiments with shRBM15 cells according to more stable knockdown effects. Second, we did not conduct MeRIP-seq. This may result in some omissions of important downstream genes. We will continue to explore other genes and mechanisms modified by RBM15 in the future.

In summary, RBM15 plays an oncogenic role in HCC. Upregulation of RBM15 facilitates the progression of HCC via m^6^A-IGF2BP1-dependent epigenetic stabilization of YES1 (Fig. 8d). As a novel biomarker, RBM15 has promising clinical application value. Our findings provide potential avenues for exploring efficient therapeutic strategies for HCC.

## Materials and methods

### Cells, samples, and patients

Human hepatocellular carcinoma cell lines HCC-LM3 (RRID: CVCL_6832), MHCC97H (RRID: CVCL_4972), and Huh7 (RRID: CVCL_0336) were used in this work. All cell lines have been authenticated using short tandem repeat. All samples were obtained from 70 HCC patients who underwent hepatectomy from 2015 to 2018 in First Affiliated Hospital of Zhejiang University. Ten specimens were used to measure the expression levels of RBM15 in HCC and normal tissues using western blotting assays. Another 60 samples with clinicopathological data were subjected to TMA construction and were set as cohort-1. Cohort-2 was derived from TCGA data (https://tcga-data.nci.nih.gov/). A total of 371 liver cancer patients’ clinical data with tumor RNA expression data were collected. Considering the influence of surgical factors, cases with <30 days follow-up were excluded. Cases with insufficient clinical data were also excluded. Finally, cohort-2 consisted of 320 cases. We used the median value of RNA expression as the cut-off to divide patients into high- or low-expression groups. Institutional Ethics Committee in First Affiliated Hospital of Zhejiang University approved the study. Written informed consents were acquired from patients following the Declaration of Helsinki.

### Bioinformatics analysis and RNA-seq

Gene expression profiles of GSE14520, GSE62232, and GSE6764 were identified from the Gene Expression Omnibus (GEO) database (https://www.ncbi.nlm.nih.gov/geo/). GEO2R was used to identify the expression of RBM15. We used HCC-LM3 cells and commissioned Biomarker Technologies Corporation (Beijing China) to performed RNA-seq on an Illumina NovaSeq 6000 (Illumina, USA). R package “DESeq2” was used to identify DEGs. An adjusted *p* value <0.05 and Fold Change >1.5 were set as cut-off criteria for screening out DEGs. GO, KEGG enrichment analyses, and GSEA were performed based on the filtered-out DEGs. Other online bioinformatics analysis websites were also employed, including ACLBI (https://www.aclbi.com/static/), GEPIA [35] (http://gepia.cancer-pku.cn/), m6A2Target (http://m6a2target.canceromics.org/), Kaplan–Meier Plotter [36] (http://kmplot.com/), TISIDB [37] (http://cis.hku.hk/TISIDB/), and TIMER [38] (https://cistrome.shinyapps.io/timer/).

### Development and validation of the risk-prediction model

To determine the clinical value of RMB15, we developed a nomogram combining clinical characteristics and RBM15 expression in RNA stratification (the RBM15 model) according to cohort-2 for prediction of OS at 3 and 5 years in individual HCC patients. We built another nomogram based on the pathologic TNM stage (the TNM model) for head-to-head comparison with the RBM15 model. Cohort-1 was used to validate the new model in protein stratification and was also compared with the TNM model.

### m^6^A dot blot assay

After dissolving in 3× volumes of RNA incubation butter and denaturing within 5 min at 65 °C, the mRNA samples were divided into subgroups of 400, 200, and 100 ng. Then the samples were dissolved in SSC butter (Sigma-Aldrich, Germany) loaded to Amersham Hybond-N+ membranes (GE Healthcare, USA) installed in a Bio-Dot Apparatus (Bio-Rad, USA). After crosslinking with ultraviolet light, the membranes were stained with 0.02% methylene blue (Sangon Biotech, China). Blue dots were scanned to indicate the input RNA content. The membranes were incubated with m^6^A antibody and dot blots were visualized after incubation with secondary antibody.

### Methylated RNA immunoprecipitation

According to the manufacturer’s protocol, the Magna MeRIP™m^6^A Kit (Millipore, Germany) was used for the MeRIP assay. Briefly, a total of 150 μg RNA were isolated and fragmented into ≤100 nucleotides and immunoprecipitated with magnetic beads pre-coated anti-m^6^A antibody or anti-mouse IgG. Then the m6A-modified RNA fragments were eluted for qPCR. The m^6^A sites of target genes were predicted using SRAMP (http://www.cuilab.cn/sramp) and RBMase (http://rna.sysu.edu.cn/rmbase/). All the primers are listed in Supplementary Table 1.

### Statistical analysis

Chi-Square test was used to evaluate qualitative data, while two-way analysis of variance or two-tailed Student’s *t* test was used for quantitative data. Kaplan–Meier curves were drawn, and univariate and multivariate Cox proportional hazard regressions were employed to identify independent risk factors. We set type I error = 0.1 as inclusion and exclusion criteria in the stepwise multivariate Cox analysis. The procedure of prediction model building was described in our previous study [27]. Time-dependent ROC curves [39] were drawn to compare the RBM15 and TNM models’ predictive capacities.

Experiments were repeated three times independently. The data were presented as mean ± SD. GraphPad Prism 8.0 software and R software for Windows (version 3.6.1) were employed for statistical analyses. The statistical significance level was set at *p < 0.05, **p < 0.01, ***p < 0.001, and ****p < 0.0001.

Other methodologies are described in Supplementary Materials (as shown in Supplementary Methods, Supplementary Tables 1–4, and Supplementary Cell Short Tandem Repeat Certificates).

## Supplementary information

supplementary materials merged

Supplementary methods

Supplementary figure legends

Supplementary figure 1

Supplementary figure 2

Supplementary figure 3

Supplementary figure 4

Supplementary figure 5

Supplementary figure 6

supplementary table 1

supplementary table 2

supplementary table 3

supplementary table 4

supplementary table 5

supplementary table 6

STR for cells

## Data Availability

The datasets used and/or analyzed during the current study are available from the corresponding author on reasonable request.

## Code availability

Computer code for nomogram is described in Supplementary Materials (as shown in Supplementary Methods).
