# Supplementary material for: RBM15 promotes hepatocellular carcinoma progression by regulating N6-methyladenosine modification of YES1 mRNA in an IGF2BP1-dependent manner
Source: Cell Death Discov. 2021 Oct 27;7:315. doi: 10.1038/s41420-021-00703-w (PMC8551180; doi:10.1038/s41420-021-00703-w)
Supplement: Supplementary file 1 — supplementary materials merged [file 41420_2021_703_MOESM1_ESM.pdf]

Cell culture

Human hepatocellular carcinoma cell lines Huh7 (RRID: CVCL_0336), HCC-LM3 (RRID: CVCL_6832), MHCC97H (RRID: CVCL_4972) were purchased from Shanghai Institutes of Biological Sciences (Shanghai, China). All cells were routinely cultured in the minimum necessary medium (MEM, BI, Israel) supplemented with 10% fetal bovine serum (FBS, BI), penicillin (100units/ml) and streptomycin (100g/ml), and cultured in a thermostatic incubator (Thermo Scientific, USA) with a humidified environment of 5% CO^2 and 37 °C.

RNA extraction and RT-qPCR

Total RNA was isolated using FastPure Cell/Tissue Total RNA Isolation Kit V2 （Vazyme Biotech, Nanjing, China） and cDNA was synthesized with HiScript II Q RT SuperMix for qPCR (Vazyme Biotech, Nanjing, China) according to the manufacturer’s protocol. Bio-Rad QX100 Droplet Digital PCR system (USA) was used to measure expression RNA by SYBR Green (Vazyme Biotech, Nanjing, China). The method for calculating the relative RNA amount was described in our previously study. All premiers were obtained from Tsingke Biological Technology (Beijing, China) and presented in Supplementary Table 1

Western blotting

Total proteins were extracted from tissues or cells using precooled RIPA buffer (Beyotime, Shanghai, China) containing protease and phosphatase inhibitors (Thermo Scientific, USA). Protein quantification was performed using the Bicinchoninic Acid protein assay kit (Thermo Scientific, USA). The same amount of protein samples was separated by 4-12% SDS-PAGE (GenScript, Nanjing, China) and then transferred to a 0.45 μm PVDF (Millipore, USA). After blocking with 5% skim milk in TBST buffer for 1 h, the membrane was incubated with the corresponding primary antibody at 4 ℃ overnight. After washing with TBST buffer for 3 times, the membrane was then incubated with enzyme-labeled secondary antibody at room temperature for 1 h.

Imaging system (Bio-Rad, USA) and enhanced chemiluminescence detection kit (Servicebio, Wuhan, China) were used for Western blotting. GAPDH was chosen as loading control. All the antibodies used in the study were listed in Supplementary Table 2.

Immunohistochemistry (IHC)

The IHC staining of TMA cohort was applied to determine expression of RBM15 protein and the connection between RBM15 and prognosis of HCC patients. IHC intensity scores and percentage of positive cells was defined as we described in our previous study [10]. All score was assessed by two pathologists who were unaware of the prognosis of patients independently. Besides, subcutaneous tumor specimens from mice were fixed with formalin and embedded in paraffin. Then IHC staining of RBM15, YES1 and PCNA were performed on these tumors.

RNA interference and plasmids.

Small interfering RNAs (siRNA) against RBM15, YES1, IGF2BP1, IGF2BP3, YTHDF1 and negative control RNAs (siNC) were synthesized by Shangya Biotechnology (Hangzhou, China). pcDNA3.1-RBM15, pcDNA3.1-YES1 and empty vector were obtained from RPTbio (Hangzhou, China) for overexpression assays. Transient transfection was performed according to the manufacturer's protocol by jetPRIME Polyplus Kit (France). All the sequences were summarized in Supplementary Table 3.

Construction of stable knockdown cells

Lentiviruses expression small hairpin RNAs (shRNAs) targeting RBM15 (shRBM15#1, shRBM15#2 and shNC) were obtained from Zorin(Shanghai, China). Huh7 and HCC-LM3 cells were applied to establish stable RBM15 knockdown models. Infected cells were selected by 3μg/ml puromycin for two weeks before subsequent assays. All the targeted sequences were listed in Supplementary Table 3.

Cell proliferation assay, colony formation and EdU incorporation assay.

Cell proliferation ability was determined by Cell Counting Kit-8 according to the manufacturer's protocol. In colony formation assay, 1.5× 103 cells were plated in 6-well cell culture plate with three repetitions. After incubation of two weeks, the plates were fixed by paraformaldehyde and stained with 1% crystal violet for 10min. In Edu assay, A 5-ethynyl-20-deoxyuridine (EdU) assay kit (Ribobio, Guangzhou, China) was used to evaluate the proliferation ability of HCC cells according to the manufacturer's protocol. The results were visualized by a fluorescence microscope.

Migration and invasion assays

In migration or invasion assays, a 24-well plate was used with a transwell filter insert (Corning, NY, USA) with a pore size of 8 μm. In the invasion experiment, diluted matrix was added to the transwell filter insert in advance. 8×10^4 HCC cells in serum-free medium were placed in the upper cavity, and then the medium containing 10% fetal bovine serum was added to the lower cavity. After 48 h (migration) or 72 h (invasion) culture at 37 ° C, the submembranous cells were fixed and stained with crystal violet. Then cell counts in five random domains were then performed under the microscope.

Subcutaneous xenograft experiments

Four-week-old male Balb/c nude mice were obtained from Shanghai Experimental Animal Center of Chinese Academic of Sciences (Shanghai,China). 5×10^6 Huh7 and HCC-LM3 cells resuspended in 100μl PBS were subcutaneously injected to the left flank of the mice (randomly selected, five mice per group for Huh7 cells in the first time and ten mice per group for HCC-LM3 cells in the second time. No blinding was performed). Tumor sizes were measured regularly. After feeding of more than 3 weeks, mice were sacrificed and tumors were surgically dissected for histology analyses. The tumor volume was calculated with the equation: $(\text{length}\times\text{width}^2)/2$. The animal experiments were approved by the Ethics Committee for Laboratory Animals of the

First Affiliated Hospital, Zhejiang University.

RNA immunoprecipitation (RIP)

Magna RIP kit (Millipore, Germany) was applied to conduct RIP assay in accordance with manufacturer's recommendation. In Brief, magnetic beads were mixed with anti-IGF2BP1 (Abclonal, China) and anti-rabbit IgG (Millipore, Germany) and added to sufficient cell lysates. Then, target RNA-protein complexes were eluted and purified for qPCR.

Luciferase reporter assay

cDNAs containing 3' UTR sequence of YES1 were cloned into luciferase reporter vectors (pcDNA3.1 vector including firefly and renilla luciferase). For mutant report plasmids, two adenosine (A) in m6A sties were replaced with cytosine (C). RBM15-knockdown HCC cells were transfected with wild-type or mutated YES1 reporter plasmids. After 24h, the luciferase activity was tested using Dual Luciferase Reporter Assay Kit (Vazyme Biotech Co.,Ltd, China) The inserted sequences were listed Supplementary Table 4

RNA decay assay

RNA decay assay was performed to evaluate RNA stability. HCC cells were cultured in 6-well plates followed by treatment of RBM15 knock-down. Actinomycin D (MCE, HY-17559) was added into each well. After 0, 12 and 24h, we collected cells to quantify the relative abundance of YES1 mRNA (relative to 0h).

Computer code for Rstudio

```
library(rms)
library(foreign)
library(survival)
setwd("C:/R")
data<-read.csv("rbm15.csv")
View(data)
str(data)
data$TNM<-factor(data$TNM,labels=c('I', 'II', 'III', 'IV'))
data$RBM15<-factor(data$RBM15,labels=c('Low','High'))
data$Age<-factor(data$Age,labels=c('≤73',' > 73'))
str(data)
dev<- data
y<-Surv(dev$OS,dev$Status ==1,type="right")
mod1<-coxph(y~ RBM15 + TNM+Age, data=dev)
summary(mod1)
y<-Surv(dev$OS,dev$Status ==1,type="right")
mod2<-coxph(y~ TNM, data=dev)
summary(mod2)
ddist <- datadist(dev)
options(datadist='ddist')
units(dev$OS) <- "Months"
fcox1 <- cph(Surv(OS, Status) ~ RBM15 + TNM+Age, surv=T,x=T, y=T,data=dev)
med <- Quantile(fcox1)
nom.sur1 <- nomogram(fcox1, fun=function(x) med(lp=x), funlabel="Median Survival Time",lp=F)
plot(nom.sur1)
surv <- Survival(fcox1)
nom1 <- nomogram(fcox1, fun=list(function(x) surv(36, x), function(x) surv(60, x)),
funlabel=c("3-years Survival Probability", "5-years Survival Probability"),lp=F)
plot(nom1)
library(nomogramEx)
nomogramEx(nomo=nom1, np=2, digit=9)
dev$RBM15point <- ifelse(dev$RBM15=="Low",0, 19.45)
dev$TNMpoint <- ifelse(dev$TNM=="I",0, ifelse(dev$TNM=="II",13.75, ifelse(dev$TNM=="
III",52.29, 100)))
dev$Agepoint <- ifelse(dev$Age=="≤73",0, 27.89)
dev$points1 <- dev$RBM15point + dev$Agepoint + dev$TNMpoint
dev$RBM15point <- ifelse(dev$RBM15=="Low",0, 100)
dev$TNMpoint <- ifelse(dev$TNM=="I",44.05, ifelse(dev$TNM=="II",0, ifelse(dev$TNM=="
III",42.68, 90)))
dev$points1 <- dev$RBM15point + dev$TNMpoint
fcox2 <- cph(Surv(OS, Status) ~ TNM, surv=T,x=T, y=T,data=dev)
med <- Quantile(fcox2)
nom.sur2 <- nomogram(fcox2, fun=function(x) med(lp=x), funlabel="Median Survival Time",lp=F)
```

```
plot(nom.sur2)
surv <- Survival(fcox2)
nom2 <- nomogram(fcox2, fun=list(function(x) surv(36, x),    function(x) surv(60, x)),
funlabel=c("3-years Survival Probability",    "5-years Survival Probability"),lp=F)
plot(nom2)
library(nomogramEx)
nomogramEx(nomo=nom2, np=2, digit=9)
source("stdca.R")
dev$three.years.Survival.Probabilitynew1=c(summary(survfit(mod1,newdata=dev),times=36)$sur
v)
dev$three.years.Survival.Probabilitynew2=c(summary(survfit(mod2,newdata=dev),times=36)$sur
v)
library(survival)
library(survivalROC)
nobs<- NROW(dev)
cutoff1<- 36
SROC1= survivalROC(Stime = dev$OS, status = dev$Status, marker = dev$points1, predict.time
=cutoff1, method= "KM" )
cut.op1= SROC1$cut.values[which.max(SROC1$TP-SROC1$FP)]
cut.op1 #
plot(SROC1$FP,SROC1$TP, type="l", xlim=c(0,1), ylim=c(0,1),
     xlab = paste( " False Positive","\n", "AUC = ",round(SROC1$AUC,3)),
     ylab = " True Positive", col="red")
abline(0,1)
legend("bottomright",c("ROC curve of 3-years overall survival"),col="red",lty=c(1,1))
SROC2= survivalROC(Stime = dev$OS, status = dev$Status, marker = dev$points2, predict.time
=cutoff1, method= "KM" )
cut.op2= SROC2$cut.values[which.max(SROC2$TP-SROC2$FP)]
cut.op2 #
plot(SROC2$FP,SROC2$TP, type="l", xlim=c(0,1), ylim=c(0,1),
     xlab = paste( " False Positive ","\n", "AUC = ",round(SROC2$AUC,3)),
     ylab = " True Positive", col="blue")
abline(0,1)
legend("bottomright",c("ROC curve of 3-years overall survival"),col="blue",lty=c(1,1))
nobs<- NROW(dev)
cutoff1<- 60
SROC1= survivalROC(Stime = dev$OS, status = dev$Status, marker = dev$points1, predict.time
=cutoff1, method= "KM" )
cut.op1= SROC1$cut.values[which.max(SROC1$TP-SROC1$FP)]
cut.op1
plot(SROC1$FP,SROC1$TP, type="l", xlim=c(0,1), ylim=c(0,1),
     xlab = paste( " False Positive","\n", "AUC = ",round(SROC1$AUC,3)),
```

```
        ylab = " True Positive", col="red")
abline(0,1)
legend("bottomright",c("ROC curve of 5-years overall survival"),col="red",lty=c(1,1))
SROC2= survivalROC(Stime = dev$OS, status = dev$Status, marker = dev$points2, predict.time
=cutoff1, method= "KM" )
cut.op2= SROC2$cut.values[which.max(SROC2$TP-SROC2$FP)]
cut.op2
plot(SROC2$FP,SROC2$TP, type="l", xlim=c(0,1), ylim=c(0,1),
        xlab = paste( " False Positive ","\n", "AUC = ",round(SROC2$AUC,3)),
        ylab = " True Positive", col="blue")
abline(0,1)
legend("bottomright",c("ROC curve of 5-years overall survival"),col="blue",lty=c(1,1))
library(timeROC)
model1ROC <- timeROC(T=dev$OS,delta=dev$Status,marker=dev$points1,cause=1, weighting =
"marginal",times=c(12, 24, 36,48, 60),iid=TRUE)
plotAUCcurve(model1ROC)
model2ROC <- timeROC(T=dev$OS,delta=dev$Status,marker=dev$points2,cause=1, weighting =
"marginal",times=c(12, 24, 36,48, 60),iid=TRUE)
plotAUCcurve(model2ROC)

plotAUCcurve(model1ROC,conf.int=F,col="red")
plotAUCcurve(model2ROC,conf.int=F,col="blue",add=TRUE)
legend("topright",c("RBM15 model","TNM model"),col=c("red","blue"),lty=1,lwd=2)
```

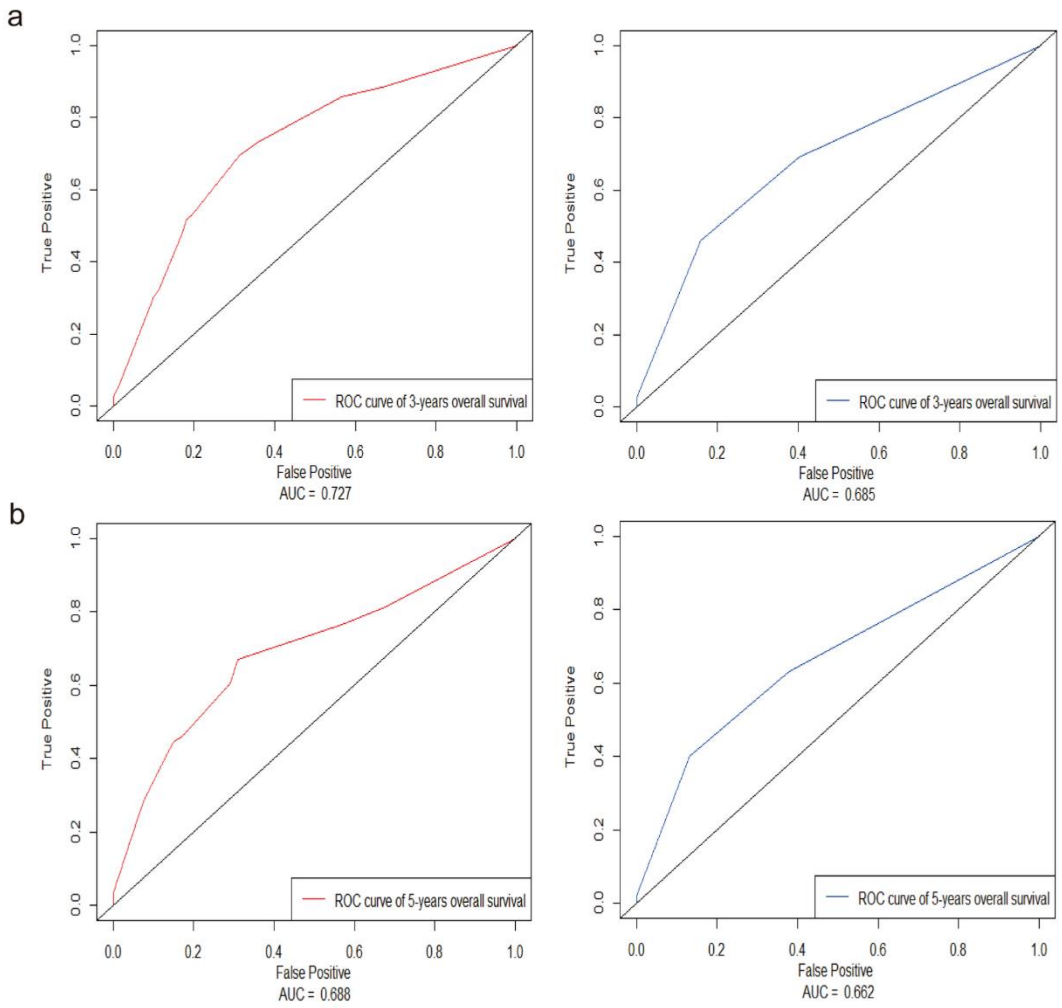

Fig S1 **The ROC curves represented the discrimination of models measured by the C-index**. **a**. for 3-years overall survival (red curve for RBM15 model and blue curve for TNM model); **b** for 5-years overall survival (red curve for RBM15 model and blue curve for TNM model)

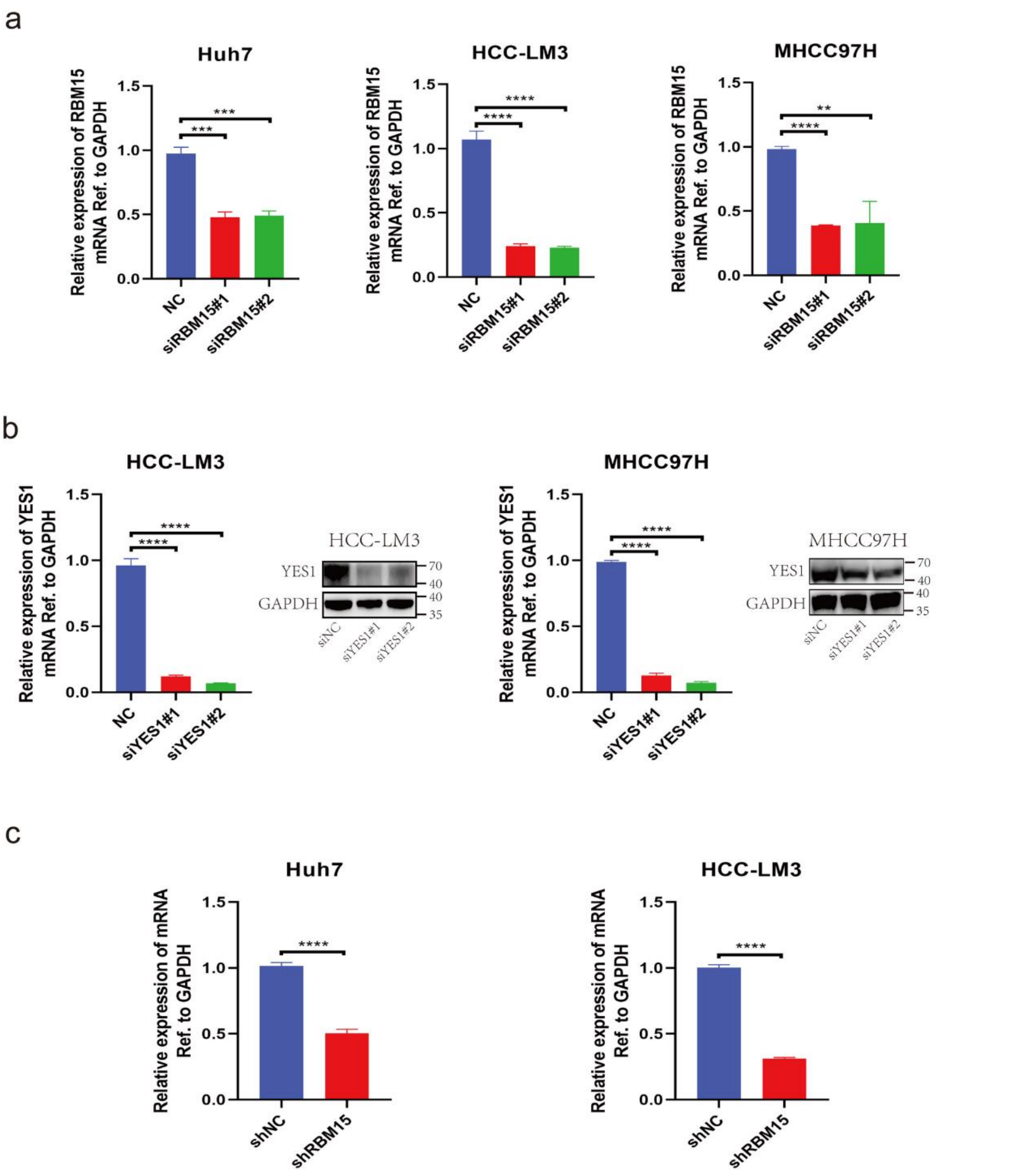

Fig S2 **Knockdown efficiency on HCC cells via qPCR. a** The efficiency of RBM15 knockdown on Huh7, HCC-LM3, MHCC97H and SNU449; **b** The efficiency of YES1 Knockdown on HCC-LM3 and MHCC97H. **c** The efficiency of stable RBM15 Knockdown on Huh7 and HCC-LM3 (** $p < 0.01$, *** $p < 0.001$, **** $p < 0.0001$; and *t*-test). The data are presented as means ± SD.

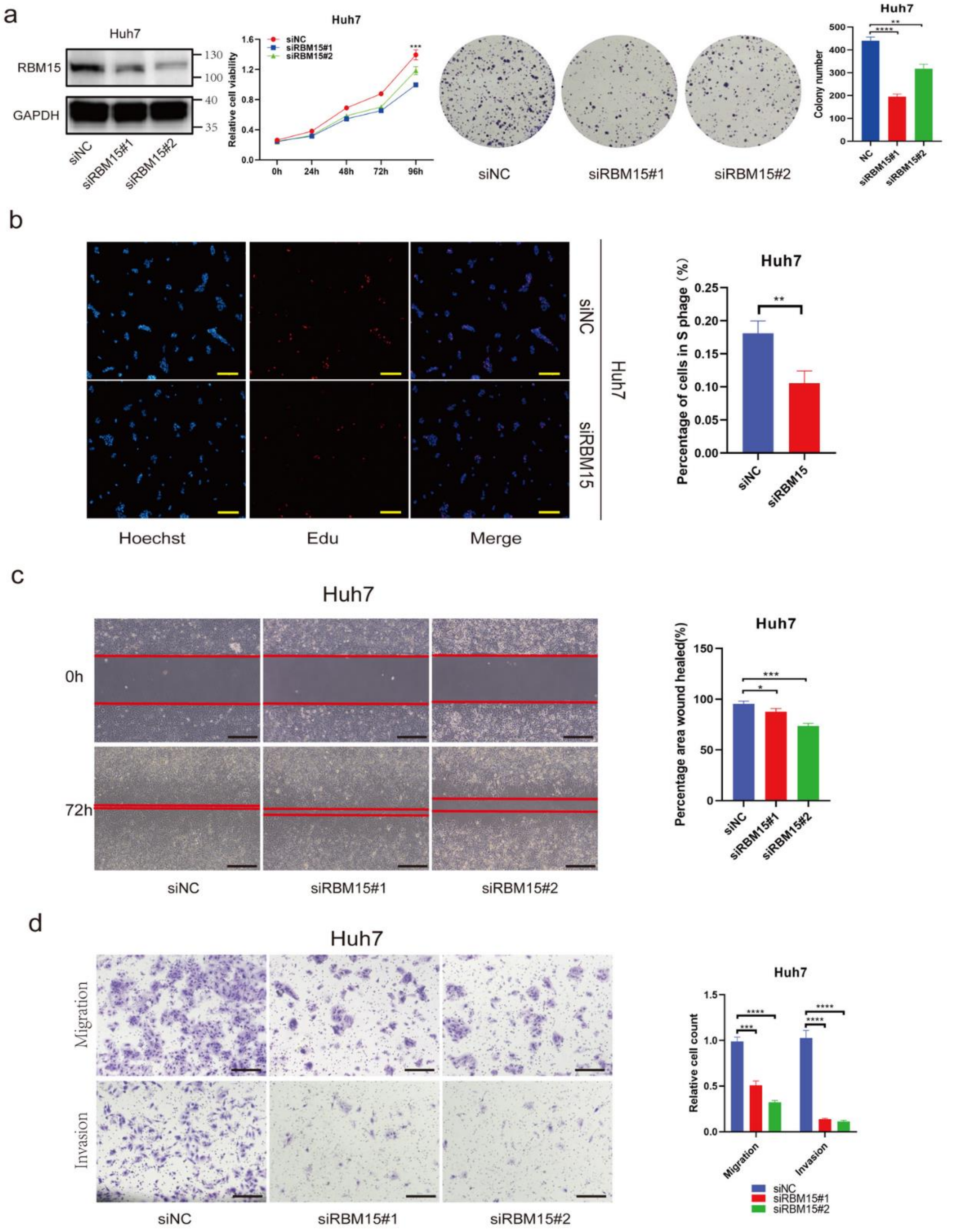

Figure S3 **RBM15 promotes tumor growth and migration / invasion capability of HCC cell in vitro. a** Negative control or siRNA (si-RBM15#1 and #2) was transfected into Huh7. The efficiency of knockdown was tested by western blotting and the proliferation capacities of HCC cells were detected by CCK-8 and colony formation assays (** p < 0.01, *** p < 0.001, **** p < 0.0001; two-way ANOVA and *t*-test); **b** Edu assay was applied to compare the proliferation abilities of Huh7 cells(scale bar, 200μm); Bar charts showed the percentage of cells in S phage based on the results of Edu assay (** p < 0.01; *t*-test); **c** Wound healing assays were performed to compared the migration capabilities of Huh7 cells (scale bars, 200μm); The percentage of healed area were quantified by bar charts (* p < 0.1, *** p < 0.001, **** p < 0.0001; *t*-test); **d** Transwell assays were

applied to detect the migration and invasion abilities of Huh7 cells after silencing RBM15 (scale bars, 200μm); Bar charts showed the relative count of HCC cells which passed through the chamber membranes when referred to negative control groups (*** $p < 0.001$, **** $p < 0.0001$; *t*-test); The data are presented as means ± SD.

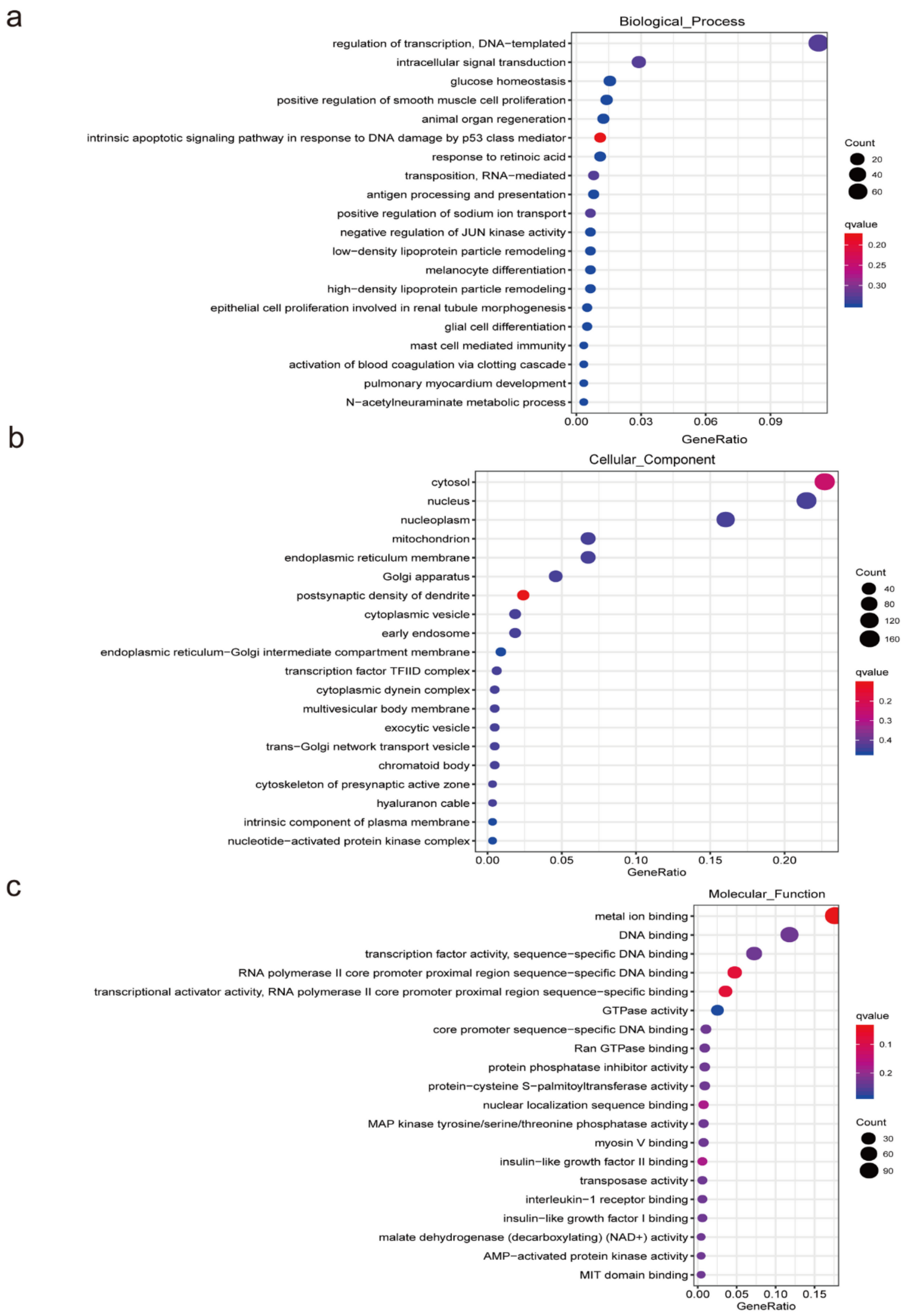

Figure S4 **The results of GO analysis for the identification of DEGs in HCC.** **a** Biological process; **b** Cellular component; **c** Molecular function

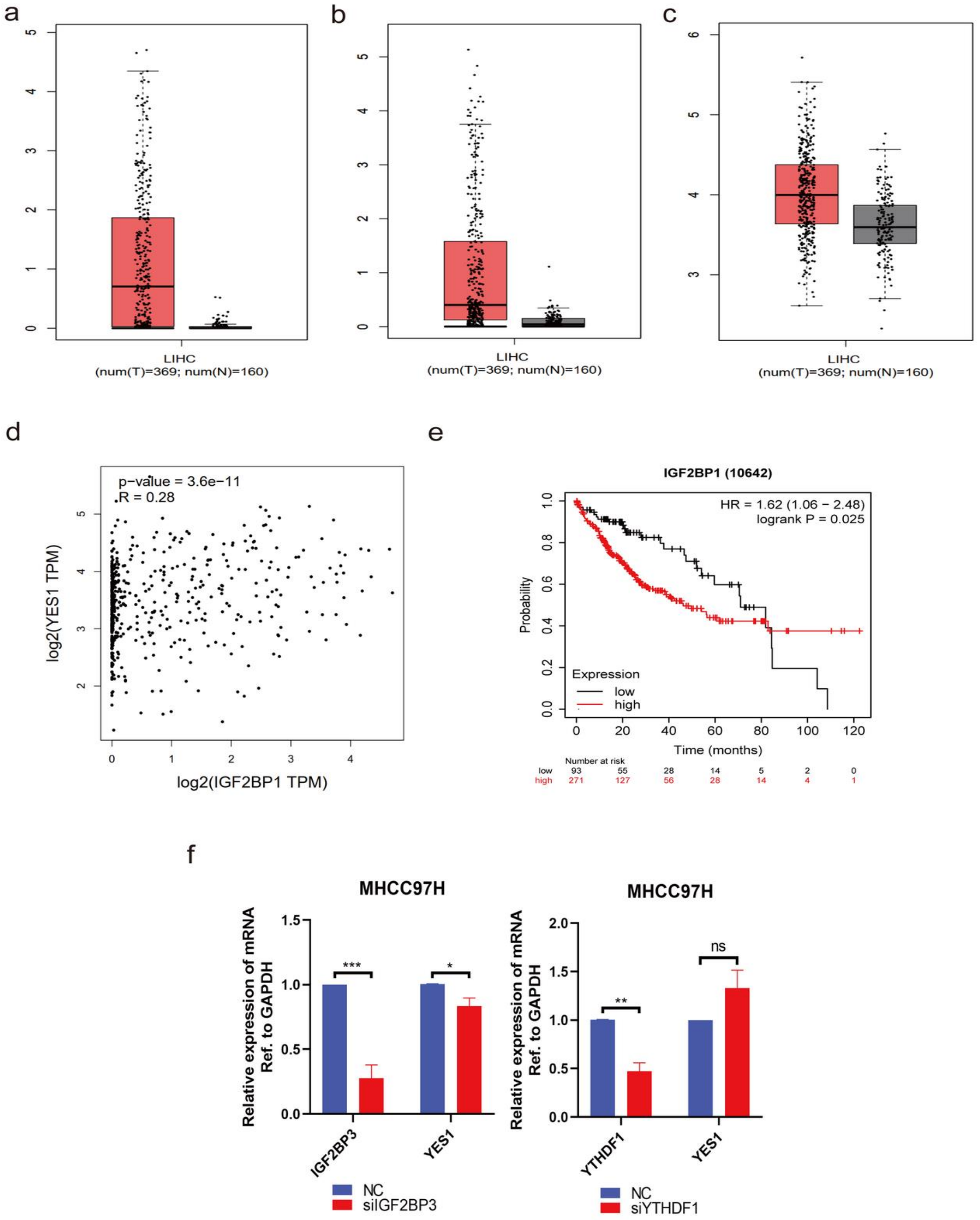

Figure S5 **other results for identifying the "reader". a** Expression of IGF2BP1 between tumor and normal tissues in HCC; **b** expression of IGF2BP3 between tumor and normal tissues in HCC; **c** expression of YTHDF1 between tumor and normal tissues in HCC; **d** YES1 was positively associated with IGF2BP1 expression; **e** High expression of IGF2BP1 indicated worse prognosis; **f** The alteration of YES1 after knockdown of IGF2BP3 and YTHDF1 (* $p < 0.05$, ** $p < 0.01$, *** $p < 0.001$; *t*-test); The data are presented as means ± SD.

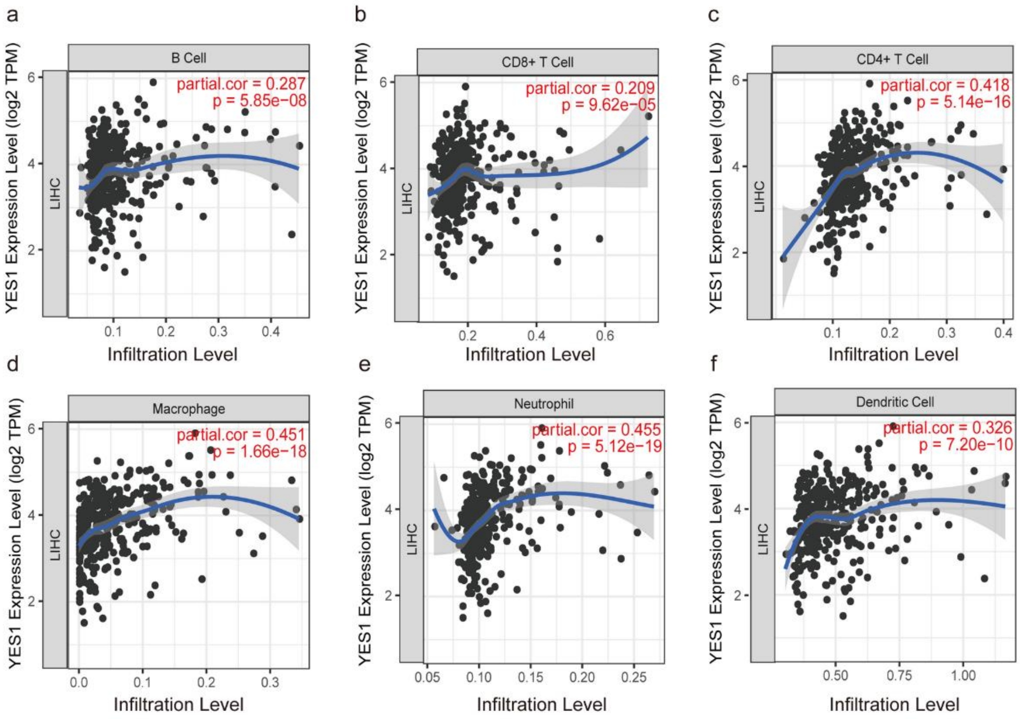

Figure S6 **YES1 is associated with immune infiltration**. **a** YES1 and B cell; **b** YES1 and CD8^{+} T cell; **c** YES1 and CD4^{+} T cell; **d** YES1 and Macrophage; **e** YES1 and Neutrophil; **f** YES1 and Dendritic cell.

Supplementary Table 1. Primers employed in this study

Gene symbol	Forward primer	Reverse primer
GAPDH	GGAGCGAGATCCCTCCAAAAT	GGCTGTTGTCATACTTCTCATGG
RBM15	AGCCGCGAGTATGATACCG	GCCCGAAGAATTTTTGGTGCTC
YES1	CTCAGGGGTAACGCCTTTTGG	CACCACCTGTTAAACCAGCAG
ARL1	TCTGTTTGGAACTCGGGAAATG	AAGGTTTTTGTACGTCACCGT
YES1-MeRIP-primer	TGCACAAATCTGCCAAAATATAAAG	TTTGTGCAACCATATCTGGGA
IGF2BP1	CAAAGGAGCCGGAAAATTCAAAT	CGTCTCACTCTCGGTGTTCA
IGF2BP3	TATATCGGAAACCTCAGCGAGA	GGACCGAGTGCTCAACTTCT
YTHDF1	ACCTGTCCAGCTATTACCCG	TGGTGAGGTATGGAATCGGAG

Supplementary Table 2. Antibody employed in this study

Antibody	Source	Identifier	RRID
GAPDH	Proteintech	Cat#10494-1-AP	AB_2263076
RBM15	Cell Signaling Technology	Cat#A4936	AB_2765940
Anti-m6A antibody	Synaptic systems	Cat#202003	AB_2279214
YES1	Proteintech	Cat#20243-1-AP	AB_10697656
p-Src	Cell Signaling Technology	Cat# 2113	AB_2106051
t-p38	Cell Signaling Technology	Cat#8690T	AB_10999090
p-p38	Cell Signaling Technology	Cat#4511T	AB_2139682
t-p44/p42 (Erk1/2)	Cell Signaling Technology	Cat#4695T	AB_390779
p-p44/p42	Cell Signaling Technology	Cat#4370T	AB_2315112
IGF2BP1 (RIP)	ABclonal	Cat#A13581	AB_2760443
PCNA (IHC)	Cell Signaling Technology	Cat#13110	AB_2636979

Supplementary Table 3. RNA interference and plasmids employed in this study

Gene symbol	Targeted sequence
siRBM15#1	GGUGAUAGUUGGGCAUAUA
siRBM15#2	UAGCAGGGCCCAAUGGUUA
shRBM15	GGUGAUAGUUGGGCAUAUA
siYES1#1	CCCACUACAGUGUCACCAU
siYES1#2	GGUGGAUACUAUAUCACAA
siIGF2BP1#1	CUCCAAAGUUCGUAUGGUUAU
siIGF2BP1#2	UGAAGAUCCUGGCCCAUAAUA
siIGF2BP3#1	CGGUGAAUGAACUUCAGAAUU
siIGF2BP3#2	GCUGCUGAGAAGUCGAUUACU
siYTHDF1#1	CCGCGUCUAGUUGUUCAUGAA
siYTHDF1#2	CAGGCUGGAGAAUAACGACAA
RBM15 pcDNA 3.1	ATGAGGACTGCGGGGCGGGACCCTGTGCCGCGGCG<br>GAGTCCAAGATGGCGGCGTGCGGTTCCGCTGTGTG<br>AAACGAGCGCGGGGCGGCGGGTTACTCAGCTCCGC<br>GGAGACGACCTCCGACGACCCGCAACAATGAAGGG<br>AAAAGAGCGCTCGCCAGTGAAGGCCAAACGCTCCC<br>GTGGTGGTGAGGACTCGACTTCCCGCGGTGAGCGG<br>AGCAAGAAGTTAGGGGGCTCTGGTGGCAGCAATGG<br>GAGCAGCAGCGGAAAGACCGATAGCGGCGGTGGG<br>TCGCGGCGGAGTCTCCACCTGGACAAGTCCAGCAG<br>TCGAGGTGGCAGCCGCGAGTATGATACCGGTGGGG<br>GCAGCTCCAGTAGCCGCTTGCATAGTTATAGCTCCC<br>CGAGCACCAAAAATTCTTCGGGCGGGGGCGAGTCG<br>CGCAGCAGCTCCCGGGGTGGAGGCGGGGAGTCAC<br>GTTCCTCTGGGGCCGCCTCCTCAGCTCCCGGCGGCG<br>GGGACGGCGCGGAATACAAGACTCTGAAGATAAGC<br>GAGTTGGGGTCCCAGCTTAGTGACGAAGCGGTGGA<br>GGACGGCCTGTTTCATGAGTTCAAACGCTTCGGTGA<br>TGTAAGTGTGAAAATCAGTCATCTGTCGGGTTCTGG<br>CAGCGGGGATGAGCGGGTAGCCTTTGTGAACTTCC<br>GGCGGCCAGAGGACGCGCGGGCGGCCAAGCATGC<br>CAGAGGCCGCCTGGTGCTCTATGACCGGCCTCTGA<br>AGATAGAAGCTGTGTATGTGAGCCGGCGCCGCAGC<br>CGCTCCCCTTTAGACAAAGATACTTATCCTCCATCAG<br>CCAGTGTGGTCGGGGCCTCTGTAGGTGGTCACCGG<br>CACCCCCCTGGAGGTGGTGGAGGCCAGAGATCACT<br>TTCCCCTGGTGGCGCTGCTTTGGGATACAGAGACTA<br>CCGGCTGCAGCAGTTGGCTCTTGGCCGCCTGCCCCC<br>TCCACCTCCGCCACCATTGCCTCGAGACCTGGAGA<br>GAGAAAGAGACTACCCGTTCTATGAGAGAGTGCGC<br>CCTGCATACAGTCTTGAGCCAAGGGTGGGAGCTGG

	AGCAGGTGCTGCTCCTTTCAGAGAAGTGGATGAGA<br>TTTCACCCGAGGATGATCAGCGAGCTAACCGGACG<br>CTCTTCTTGGGCAACCTAGACATCACTGTAACGGAG<br>AGTGATTTAAGAAGGGCGTTTGATCGCTTTGGAGTC<br>ATCACAGAAGTAGATATCAAGAGGCCTTCTCGCGGC<br>CAGACTAGTACTTACGGCTTTCTCAAATTTGAGAAC<br>TTAGATATGTCTCACCGGGCCAAATTAGCAATGTCT<br>GGCAAAATTATAATTCGGAATCCTATCAAAATTGGTT<br>ATGGTAAAGCTACACCCACCACCCGCCTCTGGGTGG<br>GAGGCCTGGGACCTTGGGTTCCTCTTGCTGCCCTGG<br>CACGAGAATTTGATCGATTTGGCACCATACGCACCA<br>TAGACTACCGAAAAGGTGATAGTTGGGCATATATCC<br>AGTATGAAAGCCTGGATGCAGCGCATGCTGCCTGG<br>ACCCATATGCGGGGCTTCCCACTTGGTGGCCCAGAT<br>CGACGCCTTAGAGTAGACTTTGCCGACACCGAACAT<br>CGTTACCAGCAGCAGTATCTGCAGCCTCTGCCCTTG<br>ACTCATTATGAGCTGGTGACAGATGCTTTTGGACAT<br>CGGGCACCAGACCCTTTGAGGGGTGCTCGGGATAG<br>GACACCACCCTTACTATACAGAGATCGTGATAGGGA<br>CCTTTATCCTGACTCTGATTGGGTGCCACCCCCACC<br>CCCAGTCCGAGAACGCAGCACTCGGACTGCAGCTA<br>CTTCTGTGCCTGCTTACGAGCCACTGGATAGCCTAG<br>ATCGCAGGCGGGATGGTTGGTCCTTGGACCGGGAC<br>AGAGGTGATCGAGATCTGCCCAGCAGCAGAGACCA<br>GCCTAGGAAGCGAAGGCTGCCTGAGGAGAGTGGA<br>GGACGTCATCTGGATAGGTCTCCTGAGAGTGACCGC<br>CCACGAAAACGTCACTGCGCTCCTTCTCCTGACCGC<br>AGTCCAGAATTGAGCAGTAGCCGGGATCG
YES1 pcDNA 3.1	GACGGATCGGGAGATCTCCCGATCCCCTATGGTGCA<br>CTCTCAGTACAATCTGCTCTGATGCCGCATAGTTAA<br>GCCAGTATCTGCTCCCTGCTTGTGTGTTGGAGGTCG<br>CTGAGTAGTGCGCGAGCAAAATTTAAGCTACAACA<br>AGGCAAGGCTTGACCGACAATTGCATGAAGAATCT<br>GCTTAGGGTTAGGCGTTTTGCGCTGCTTCGCGATGT<br>ACGGGCCAGATATACGCGTTGACATTGATTATTGACT<br>AGTTATTAATAGTAATCAATTACGGGGTCATTAGTTC<br>ATAGCCCATATATGGAGTTCCGCGTTACATAACTTAC<br>GGTAAATGGCCCGCCTGGCTGACCGCCCAACGACC<br>CCCGCCCATTGACGTCAATAATGACGTATGTTCCCAT<br>AGTAACGCCAATAGGGACTTTCCATTGACGTCAATG<br>GGTGGAGTATTTACGGTAAACTGCCCACTTGGCAGT<br>ACATCAAGTGTATCATATGCCAAGTACGCCCCCTATT<br>GACGTCAATGACGGTAAATGGCCCGCCTGGCATTAT<br>GCCCAGTACATGACCTTATGGGACTTTCCTACTTGG

	CAGTACATCTACGTATTAGTCATCGCTATTACCATGG<br>TGATGCGGTTTTGGCAGTACATCAATGGGCGTGGAT<br>AGCGGTTTGACTCACGGGGATTTCCAAGTCTCCACC<br>CCATTGACGTCAATGGGAGTTTGTTTTGGCACCAAA<br>ATCAACGGGACTTTCCAAAATGTCGTAACAACTCCG<br>CCCCATTGACGCAAATGGGCGGTAGGCGTGTACGGT<br>GGGAGGTCTATATAAGCAGAGCTCTCTGGCTAACTA<br>GAGAACCCACTGCTTACTGGCTTATCGAAATTAATA<br>CGACTCACTATAGGGAGACCCAAGCTGGCTAGTTAA<br>GCTTGGTACCGAGCTCGGATCCGCCACCatgggctgcatta<br>aaagtaaagaaaacaaaagtccagccattaaatacagacctgaaaatactccagag<br>cctgtcagtacaagtgtgagccattatggagcagaacccactacagtgtcaccatgtc<br>cgtcatcttcagcaaagggaacagcagttaatttcagcagtctttccatgacaccattt<br>ggaggatcctcaggggtaacgccttttggaggtgcatcttcctcattttcagtggtgcc<br>aagttcatatcctgctggtttaacaggtggtgttactatatttgtggccttatatgattatg<br>aagctagaactacagaagacctttcatttaagaagggtgaaagatttcaaataattaac<br>aatacggaaggagattggtgggaagcaagatcaatcgctacaggaaagaatggtta<br>tatcccgagcaattatgtagcgcctgcagattccattcaggcagaagaatggtattttg<br>gcaaaatggggagaaaagatgctgaaagattacttttgaatcctggaaatcaacgag<br>gtattttcttagtaagagagagtgaaacaactaaaggtgcttattccctttctattcgtga<br>ttgggatgagataaggggtgacaatgtgaaacactacaaaattaggaaacttgacaa<br>tggtggatactatatcacaaccagagcacaatttgatactctgcagaaattggtgaaa<br>cactacacagaacatgctgatggtttatgccacaagttgacaactgtgtgtccaactgt<br>gaaacctcagactcaaggtctagcaaaagatgcttgggaaatccctcgagaatctttg<br>cgactagaggttaaactaggacaaggatgtttcggcgaagtgtggatgggaacatg<br>gaatggaaccacgaaagtagcaatcaaaacactaaaaccaggtacaatgatgccag<br>aagctttccttcaagaagctcagataatgaaaaaattaagacatgataaacttgttcca<br>ctatatgctgttgtttctgaagaaccaatttacattgtcactgaatttatgtcaaaaggaa<br>gcttattagatttccttaaggaaggagatggaaagtatttgaagcttccacagctggtt<br>gatatggctgctcagattgctgatggtatggcatatattgaaagaatgaactatattcac<br>cgagatcttcgggctgctaatattcttgtaggagaaaatcttgtgtgcaaaatagcaga<br>ctttggtttagcaaggttaattgaagacaatgaatacacagcaagacaaggtgcaaaa<br>tttccaatcaaatggacagctcctgaagctgcactgtatggtcggtttacaataaagtct<br>gatgtctggtcatttggaattctgcaaacagaactagtaacaaagggccgagtgccat<br>atccaggtatggtgaaccgtgaagtaCtagaacaagtggagcgaggatacaggat<br>gccgtgccctcagggctgtccagaatccctccatgaattgatgaatctgtgttggaag<br>aaggaccctgatgaaagaccaacatttgaatatattcagtccttcttggaagactactt<br>cactgctacagagccacagtaccagccaggagaaatttaCTCGAGTCTA<br>GAGGGCCCTTCGACTACAAAGACCATGACGGTGAT<br>TATAAAGATCATGACATCGACTACAAGGATGACGAT<br>GACAAGTGAGTTTAAACCCGCTGATCAGCCTCGAC<br>TGTGCCTTCTAGTTGCCAGCCATCTGTTGTTTGCCCC<br>TCCCCCGTGCCTTCCTTGACCCTGGAAGGTGCCACT<br>CCCACTGTCCTTTCCTAATAAAATGAGGAAATTGCA

	TCGCATTGTCTGAGTAGGTGTCATTCTATTCTGGGGG<br>GTGGGGTGGGGCAGGACAGCAAGGGGGAGGATTG<br>GGAAGACAATAGCAGGCATGCTGG<br>GGATGCGGTGGGCTCTATGGCTTCTGAGGCGGAAA<br>GAACCAGCTGGGGCTCTAGGGGGTATCCCCACGCG<br>CCCTGTAGCGGCGCATTAAGCGCGGCGGGTGTGGT<br>GGTTACGCGCAGCGTGACCGCTACACTTGCCAGCG<br>CCCTAGCGCCCGCTCCTTTCGCTTTCTTCCCTTCCTT<br>TCTCGCCACGTTCGCCGGCTTTCCCCGTCAAGCTCT<br>AAATCGGGGGCTCCCTTTAGGGTTCCGATTTAGTGC<br>TTTACGGCACCTCGACCCCAAAAAACTTGATTAGGG<br>TGATGGTTCACGTAGTGGGCCATCGCCCTGATAGAC<br>GGTTTTTCGCCCTTTGACGTTGGAGTCCACGTTCTT<br>TAATAGTGGACTCTTGTTCCAAACTGGAACAACACT<br>CAACCCTATCTCGGTCTATTCTTTTGATTTATAAGGG<br>ATTTTGCCGATTTCGGCCTATTGGTTAAAAAATGAGC<br>TGATTTAACAAAAATTTAACGCGAATTAATTCTGTG<br>GAATGTGTGTCAGTTAGGGTGTGGAAAGTCCCCAG<br>GCTCCCCAGCAGGCAGAAGTATGCAAAGCATGCAT<br>CTCAATTAGTCAGCAACCAGGTGTGGAAAGTCCCC<br>AGGCTCCCCAGCAGGCAGAAGTATGCAAAGCATGC<br>ATCTCAATTAGTCAGCAACCATAGTCCCGCCCCTAA<br>CTCCGCCCATCCCGCCCCTAACTCCGCCCAGTTCCG<br>CCCATTCTCCGCCCCATGGCTGACTAATTTTTTTTAT<br>TTATGCAGAGGCCGAGGCCGCCTCTGCCTCTGAGCT<br>ATTCCAGAAGTAGTGAGGAGGCTTTTTTGGAGGCCT<br>AGGCTTTTGCAAAAAGCTCCCGGGAGCTTGTATATC<br>CATTTTCGGATCTGATCAAGAGACAGGATGAGGATC<br>GTTTCGCATGATTGAACAAGATGGATTGCACGCAGG<br>TTCTCCGGCCGCTTGGGTGGAGAGGCTATTCGGCTA<br>TGACTGGGCACAACAGACAATCGGCTGCTCTGATG<br>CCGCCGTGTTCCGGCTGTCAGCGCAGGGGCGCCCG<br>GTTCTTTTTGTCAAGACCGACCTGTCCGGTGCCCTG<br>AATGAACTGCAGGACGAGGCAGCGCGGCTATCGTG<br>GCTGGCCACGACGGGCGTTCCTTGCGCAGCTGTGC<br>TCGACGTTGTCACTGAAGCGGGAAGGGACTGGCTG<br>CTATTGGGCGAAGTGCCGGGGCAGGATCTCCTGTCA<br>TCTCACCTTGCTCCTGCCGAGAAAGTATCCATCATG<br>GCTGATGCAATGCGGCGGCTGCATACGCTTGATCCG<br>GCTACCTGCCCATTCGACCACCAAGCGAAACATCGC<br>ATCGAGCGAGCACGTACTCGGATGGAAGCCGGTCT<br>TGTCGATCAGGATGATCTGGACGAAGAGCATCAGG<br>GGCTCGCGCCAGCCGAACTGTTCGCCAGGCTCAAG<br>GCGCGCATGCCCGACGGCGAGGATCTCGTCGTGAC

	CCATGGCGATGCCTGCTTGCCGAATATCATGGTGGA<br>AAATGGCCGCTTTTCTGGATTCATCGACTGTGGCCG<br>GCTGGGTGTGGCGGACCGCTATCAGGACATAGCGTT<br>GGCTACCCGTGATATTGCTGAAGAGCTTGGCGGCGA<br>ATGGGCTGACCGCTTCCTCGTGCTTTACGGTATCGC<br>CGCTCCCGATTCGCAGCGCATCGCCTTCTATCGCCTT<br>CTTGACGAGTTCTTCTGAGCGGGACTCTGGGGTTCG<br>CGAAATGACCGACCAAGCGACGCCCAACCTGCCAT<br>CACGAGATTTCGATTCCACCGCCGCCTTCTATGAAA<br>GGTTGGGCTTCGGAATCGTTTTCCGGGACGCCGGCT<br>GGATGATCCTCCAGCGCGGGGATCTCATGCTGGAGT<br>TCTTCGCCCACCCCAACTTGTTTATTGCAGCTTATAA<br>TGGTTACAAATAAAGCAATAGCATCACAAATTTCAC<br>AAATAAAGCATTTTTTTCACTGCATTCTAGTTGTGGT<br>TTGTCCAAACTCATCAATGTATCTTATCATGTCTGTAT<br>ACCGTCGACCTCTAGCTAGAGCTTGGCGTAATCATG<br>GTCATAGCTGTTTCCTGTGTGAAATTGTTATCCGCTC<br>ACAATTCCACACAACATACGAGCCGGAAGCATAAA<br>GTGTAAAGCCTGGGGTGCCTAATGAGTGAGCTAACT<br>CACATTAATTGCGTTGCGCTCACTGCCCGCTTTCCA<br>GTCGGGAAACCTGTCGTGCCAGCTGCATTAATGAAT<br>CGGCCAACGCGCGGGGAGAGGCGGTTTGCGTATTG<br>GGCGCTCTTCCGCTTCCTCGCTCACTGACTCGCTGC<br>GCTCGGTCGTTCGGCTGCGGCGAGCGGTATCAGCTC<br>ACTCAAAGGCGGTAATACGGTTATCCACAGAATCAG<br>GGGATAACGCAGGAAAGAACATGTGAGCAAAAGG<br>CCAGCAAAAGGCCAGGAACCGTAAAAAGGCCGCG<br>TTGCTGGCGTTTTTCCATAGGCTCCGCCCCCCTGAC<br>GAGCATCACAAAAATCGACGCTCAAGTCAGAGGTG<br>GCGAAACCCGACAGGACTATAAAGATACCAGGCGT<br>TTCCCCCTGGAAGCTCCCTCGTGCGCTCTCCTGTTC<br>CGACCCTGCCGCTTACCGGATACCTGTCCGCCTTTC<br>TCCCTTCGGGAAGCGTGGCGCTTTCTCATAGCTCAC<br>GCTGTAGGTATCTCAGTTCGGTGTAGGTCGTTCGCT<br>CCAAGCTGGGCTGTGTGCACGAACCCCCCGTTCAG<br>CCCGACCGCTGCGCCTTATCCGGTAACTATCGTCTT<br>GAGTCCAACCCGGTAAGACACGACTTATCGCCACT<br>GGCAGCAGCCACTGGTAACAGGATTAGCAGAGCGA<br>GGTATGTAGGCGGTGCTACAGAGTTCTTGAAGTGGT<br>GGCCTAACTACGGCTACACTAGAAGAACAGTATTTG<br>GTATCTGCGCTCTGCTGAAGCCAGTTACCTTCGGAA<br>AAAGAGTTGGTAGCTCTTGATCCGGCAAACAAACC<br>ACCGCTGGTAGCGGTGGTTTTTTTGTTTGCAAGCAG<br>CAGATTACGCGCAGAAAAAAAGGATCTCAAGAAGA

	TCCTTTGATCTTTTCTACGGGGTCTGACGCTCAGTG GAACGAAAACTCACGTTAAGGGATTTTGGTCATGA GATTATCAAAAAGGATCTTCACCTAGATCCTTTTAAA TTAAAAATGAAGTTTTAAATCAATCTAAAGTATATAT GAGTAAACTTGGTCTGACAGTTACCAATGCTTAATC AGTGAGGCACCTATCTCAGCGATCTGTCTATTTCGTT CATCCATAGTTGCCTGACTCCCCGTCGTGTAGATAA CTACGATACGGGAGGGCTTACCATCTGGCCCCAGTG CTGCAATGATACCGCGAGACCCACGCTCACCGGCTC CAGATTTATCAGCAATAAACCAGCCAGCCGGAAGG GCCGAGCGCAGAAGTGGTCCTGCAACTTTATCCGC CTCCATCCAGTCTATTAATTGTTGCCGGGAAGCTAG AGTAAGTAGTTCGCCAGTTAATAGTTTGCGCAACGT TGTTGCCATTGCTACAGGCATCGTGGTGTCACGCTC GTCGTTTGGTATGGCTTCATTCAGCTCCGGTTCCCA ACGATCAAGGCGAGTTACATGATCCCCCATGTTGTG CAAAAAAGCGGTTAGCTCCTTCGGTCCTCCGATCGT TGTCAGAAGTAAGTTGGCCGCAGTGTTATCACTCAT GGTTATGGCAGCACTGCATAATTCTCTTACTGTCATG CCATCCGTAAGATGCTTTTCTGTGACTGGTGAGTAC TCAACCAAGTCATTCTGAGAATAGTGTATGCGGCGA CCGAGTTGCTCTTGCCCGGCGTCAATACGGGATAAT ACCGCGCCACATAGCAGAACTTTAAAAGTGCTCATC ATTGGAAAACGTTCTTCGGGGCGAAAACTCTCAAG GATCTTACCGCTGTTGAGATCCAGTTCGATGTAACC CACTCGTGCACCCAACTGATCTTCAGCATCTTTTAC TTTCACCAGCGTTTCTGGGTGAGCAAAAACAGGAA GGCAAAATGCCGCAAAAAAGGGAATAAGGGCGAC ACGGAAATGTTGAATACTCATACTCTTCCTTTTTCAA TATTATTGAAGCATTTATCAGGGTTATTGTCTCATGA GCGGATACATATTTGAATGTATTTAGAAAAATAAACA AATAGGGGTTCCGCGCACATTTCCCCGAAAAGTGCC ACCTGACGTC

Supplementary Table 4 the inserted sequences employed in luciferase reporter assay

Factor	Sequences
YES1-3UTR-WT:	TTCAAGTAGCCTATTTTATATGCACAAATCTGCCAAAATATAAAGAACTTGTGTA GATTTTCTACAGGAATCAAAAGAAGAAAATCTTCTTTACTCTGCATGTTTTTAAT GGTAAACTGGAATCCCAGATATGGTTGCACAAAACCACTTTTTTTTCCCCAAGT ATTAAACTCTAATGTACCAATGATGAATTTATCAGCGTATTTCAGGGTCCAAACA AAATAGAGCTAAGATACTGATGACAGTGTGGGTGACAGCATGGTAATGAAGGAC AGTGAGGCTCCTGCTTATTTATAAATCATTTCCTTTCTTTTTTTCCCCAAAGTCAG AATTGCTCAAAGAAAATTATTTATTGTTACAGATAAAACTTGAGAGATAAAAAGC TATACCATAATAAAATCTAAAATTAAGGAATATCATGGGACCAAATAATTCCATTCC AGTTTTTTAAAGTTTCTTGCATTTATTATTCTCAAAAGTTTTTTCTAAGTTAAACAG TCAGTATGCAATCTTAATATATGCTTTCTTTTGCATGGACATGGGCCAGGTTTTTCA AAAGGAATATAAACAGGATCTCAAACTTGATTAAATGTTAGACCACAGAAGTGGA ATTTGAAAGTATAATGCAGTACATTAATATTCATGTTCATGGAACTGAAAGAATAAG AACTTTTTCACTTCAGTCCTTTTCTGAAGAGTTTGACTTAGAATAATGAAGGTAAC TAGAAAGTGAGTTAATCTTGTATGAGGTTGCATTGATTTTTTAAGGCAATATATAAT TGAAACTACTGTCCAATCAA

YES1-3UTR-Mut	TTCAAGTAGCCTATTTTATATGCACAAATCTGCCAAAATATAAAGACCTTGTGTAGATTTT<br>CTACAGGAATCAAAAGAAGAAAATCTTCTTTACTCTGCATGTTTTTAATGGTAACCTGGA<br>ATCCCAGATATGGTTGCACAAAACCACTTTTTTTTCCCCAAGTATTAAACTCTAATGTAC<br>CAATGATGAATTTATCAGCGTATTTCAGGGTCCAAACAAAATAGAGCTAAGATACTGAT<br>GACAGTGTGGGTGACAGCATGGTAATGAAGGACAGTGAGGCTCCTGCTTATTTATAAA<br>TCATTTCCTTTCTTTTTTTCCCCAAAGTCAGAATTGCTCAAAGAAAATTATTTATTGTTA<br>CAGATAAAACTTGAGAGATAAAAAGCTATACCATAATAAAATCTAAAATTAAGGAATAT<br>CATGGGACCAAATAATTCCATTCCAGTTTTTTAAAGTTTCTTGCATTTATTATTCTCAAA<br>AGTTTTTTCTAAGTTAAACAGTCAGTATGCAATCTTAATATATGCTTTCTTTTGCATGGA<br>CATGGGCCAGGTTTTTCAAAAGGAATATAAACAGGATCTCAAACTTGATTAAATGTTAG<br>ACCACAGAAGTGGAATTTGAAAGTATAATGCAGTACATTAATATTCATGTTCATGGAAC<br>TGAAAGAATAAGAACTTTTTCACTTCAGTCCTTTTCTGAAGAGTTTGACTTAGAATAAT<br>GAAGGTAACTAGAAAGTGAGTTAATCTTGTATGAGGTTGCATTGATTTTTTAAGGCAAT<br>ATATAATTGAAACTACTGTCCAATCAA

Supplementary Table 5. Univariate and multivariate Cox analysis of Cohort-1 for overall survival and disease-free survival

Factors	Overall Survival						Disease Free Survival					
	Univariate analysis			Multivariate analysis			Univariate analysis			Multivariate analysis		
	HR	95%CI	*P*	HR	95%CI	*P*	HR	95%CI	*P*	HR	95%CI	*P*
Age			0.506						0.675			
≤60	1						1					
> 60	0.80	0.42-1.54					0.83	0.35-1.97				
AJCC stage			0.845						**0.030**			0.057
Stage I-II	1						1			1		
Stage III-IV	1.06	0.62-1.80					2.38	1.09-5.20		2.15	0.98-4.76	
Tumor number												
Single	1		0.088				1		0.768			
Multiple	1.61	0.93-2.79					1.12	0.52-2.42				
Tumor size			0.396						0.564			
≤5cm	1						1					
> 5cm	1.31	0.70-2.46					0.80	0.37-1.72				
Tumor encapsulation			**0.011**			0.302			0.418			
Intact	1			1			1					
Broken	2.06	1.18-3.01		1.38	0.75-2.54		1.36	0.65-2.84				
Microvascular infiltration			**0.000**			**0.000**			0.610			
No	1			1			1					
Yes	4.92	2.43-9.93		4.80	2.25-10.252		0.73	0.22-2.47				
HbsAg			0.995									
Negative	1						1					
Positive	1.00	0.36-2.81					1.24	0.29-5.27				

Cirrhosis			0.372						0.741			
Yes	1						1					
No	1.39	0.68-2.84					1.16	0.47-2.85				
AFP			0.503						0.105			
≤400 μg/L	1						1					
> 400 μg/L	1.21	0.70-2.09					1.83	0.88-3.81				
RBM15			**0.018**			**0.014**			**0.001**			**0.002**
Low	1			1			1			1		
High	1.98	1.13-3.48		2.06	1.16-3.68		3.82	1.68-8.68		3.57	1.57-8.12	

Supplementary Table 6. Down-regulated DEGs associated with RBM15

Symbol	NC1_FPKM	NC2_FPKM	NC3_FPKM	KD1_FPKM	KD2_FPKM	KD3_FPKM	FDR	log2FC
ARL1	28.74541	30.53362	24.81846	3.644433	3.095859	4.618634	3.5E-115	-3.14422
B4GALT5	36.47572	35.52025	35.42973	10.46131	10.88463	10.14649	3.5E-115	-1.76694
EPB41L4B	22.87252	22.10918	23.13082	5.989935	6.127581	5.606162	2.8E-106	-1.92874
VTI1B	27.17256	31.3709	23.74763	3.627992	4.102638	2.849195	3.7E-106	-2.60436
TBL1XR1	28.21743	31.05428	28.7971	6.760668	9.314571	8.136907	1.1E-80	-2.0808
KRT80	51.40853	49.62909	46.82152	8.444625	11.27863	9.860626	7.47E-78	-2.26293
YES1	24.9545	24.55897	24.47059	6.609574	7.710465	6.764911	9.84E-77	-1.78789
LPGAT1	10.66443	10.38001	10.27737	3.554056	3.477351	3.248721	1.68E-75	-1.60904
UBE2G2	20.78699	20.27532	18.58916	7.06915	7.005584	6.96706	1.04E-72	-1.80197
CMTM6	22.14637	23.23198	24.01542	5.595442	4.758319	6.005949	6.34E-68	-2.03705
STK17B	9.500625	8.873977	9.19608	2.454562	2.802248	2.873529	5.74E-66	-1.81326
SH3BGRL2	7.069653	7.16356	6.792233	1.325508	1.650054	1.491665	3.4E-65	-2.16969
RPA1	37.23274	39.88624	38.9987	15.8671	15.6534	14.5586	6.18E-65	-1.3482
EFCAB14	14.85284	14.27577	13.98861	4.10724	4.620489	4.434974	1.36E-62	-1.68379
RDH10	27.97268	24.96361	28.13972	8.718064	7.695217	8.326754	1.53E-61	-1.70255
COPZ1	117.1477	115.6929	106.7922	64.25903	52.77477	46.04437	2.32E-61	-1.32041
UXS1	104.831	107.0737	108.4104	40.49685	44.31445	43.47657	2.71E-59	-1.32643
NCEH1	31.6104	29.46279	30.18974	8.774097	10.55039	9.498687	5.89E-59	-1.66613
LRRC58	12.83303	10.99749	11.19432	3.541142	3.421082	2.937582	4.34E-55	-1.78874
NIPAL3	5.946472	7.254726	6.510549	3.192789	3.484843	3.395693	4.89E-54	-1.85395
CGGBP1	21.90593	21.0152	20.34236	7.659192	7.019073	8.689735	1.57E-53	-1.42425
LIN7C	10.10886	8.905188	9.792185	2.584715	2.323075	1.960693	1.95E-53	-2.00736

AIDA	25.48758	24.77009	23.33848	4.962245	6.704221	6.065707	5.68E-53	-1.99216
TTL	8.665182	8.330934	8.175505	3.599081	3.684361	3.453499	4.51E-52	-1.23227
AMMECR1	17.6363	15.08504	16.97448	4.707182	5.468175	5.166052	2.23E-51	-1.6617
TM7SF3	24.21615	23.70859	21.69289	6.320369	5.31951	5.886151	4.51E-48	-2.02743
MBTPS2	7.918523	8.124281	8.139624	2.656119	2.873389	2.632615	8.17E-48	-1.5435
NT5E	30.58139	29.66998	32.42075	12.26692	13.15077	12.98989	1.55E-47	-1.39473
LARP4B	19.23254	17.8592	16.82643	5.676357	6.227329	4.525928	3.51E-47	-1.68806
ZMPSTE24	23.70745	24.09095	25.50526	9.21589	7.622664	7.948209	3.56E-46	-1.54292
GPD2	13.24944	12.51312	12.58571	5.546091	5.726716	4.469391	8.4E-46	-1.46687
DNAJC22	24.31088	27.03758	25.0775	9.446737	6.598421	8.098181	1.22E-45	-1.4651
PBK	22.74305	23.21787	24.63073	7.105157	6.832409	7.795319	2.4E-44	-1.56518
ASF1A	21.72884	20.52573	21.3968	7.110408	6.453619	7.668694	4.14E-44	-1.55801
MRPL18	50.41539	48.88665	56.32006	16.81074	13.41591	14.53518	4.67E-44	-1.75194
PALLD	78.2922	72.57678	69.55036	29.14315	32.87326	29.83082	5.38E-44	-1.26629
MCM8	11.63453	17.57978	11.53906	4.746821	4.381496	3.855104	3.14E-43	-1.42783
ATL3	17.23831	18.44042	18.89965	8.268048	8.725532	7.88878	6E-43	-1.183
RANBP9	14.68298	15.11321	13.93107	5.738005	5.625168	5.637755	1.53E-42	-1.35186
SOS1	32.28664	27.68707	26.3257	9.384988	16.40633	9.117814	1.95E-42	-1.54481
FNDC3A	5.124842	4.857385	4.522923	1.961323	1.357867	0.966089	3.75E-42	-1.89334
PPAT	16.53039	14.84487	15.46475	6.710206	7.244393	5.63161	4.89E-42	-1.36815
MYL12A	195.6394	210.6262	233.1606	66.26916	78.53112	76.48437	2.15E-41	-1.51154
PTBP3	60.6395	54.49788	59.57019	11.71421	21.42881	17.28635	5.22E-41	-1.65078
SCML1	8.031746	7.146125	8.068526	1.327005	1.613765	0.943644	7.07E-41	-2.36998
CHML	17.04749	15.30676	17.02728	4.039838	6.045872	3.553244	7.1E-41	-1.60454
TMED7	16.11637	15.27174	15.19069	6.879817	6.633448	6.431461	3.37E-40	-1.2217
SURF6	13.80975	13.40221	13.37753	4.475162	5.345902	5.07243	6.46E-40	-1.42818

NUP43	9.074417	9.712737	9.941089	3.484334	3.48224	3.361411	6.6E-40	-1.43059
GTF2A2	30.69527	33.46264	31.98316	11.98004	10.79524	12.33501	1.16E-39	-1.41196
SLC12A7	36.58762	34.80914	34.64353	16.86096	16.74454	16.33723	3.74E-38	-1.11371
RCOR1	11.8228	11.18577	11.41897	4.773748	5.122908	4.418197	7.24E-38	-1.26018
CACYBP	82.00807	87.21892	77.50968	32.54011	27.95286	32.47719	1.1E-37	-1.32186
TMED10	26.44424	26.07769	26.048	13.51605	13.34831	13.27592	5.18E-36	-0.96777
APPBP2	4.635037	5.000998	4.578528	1.76365	1.85054	1.690503	6.88E-35	-1.39919
WDR12	5.806025	6.794574	5.776958	2.216131	2.297171	2.298622	9.61E-35	-1.40913
Homo_sapiens_newGene_5798	2.742919	2.783887	2.684989	0.05271	0.06483	0.049082	1.33E-34	-3.62083
SLC7A1	7.465758	6.903471	6.042484	2.064974	2.458197	2.026174	1.7E-34	-1.60089
ACAA1	29.33656	30.40313	27.24112	12.2805	12.79827	12.73597	3.49E-34	-1.20026
SMAD2	10.10417	10.50718	9.627896	3.840884	2.910047	2.161134	3.99E-34	-1.48117
STARD3NL	38.65163	43.25494	39.07102	15.66163	15.35146	15.89301	6.32E-34	-1.27551
C4orf46	8.968511	10.28763	10.0518	3.79377	3.454438	3.541422	1.3E-33	-1.41267
TTC5	3.719056	3.221918	4.29573	0.873836	0.952433	0.946263	1.97E-33	-1.82948
MRPL3	82.71386	90.13824	92.93935	20.52705	29.41912	26.40518	2.72E-33	-1.75989
PANK3	6.729047	6.244533	5.750834	1.961233	1.954035	1.342043	3.25E-33	-1.77465
NCOA7	7.772473	6.871818	6.811866	3.053184	2.162007	1.485315	7.55E-33	-1.6357
TMEM64	15.4949	20.03576	15.27968	5.414983	5.509087	5.500961	2.72E-31	-1.56857
CYP4F11	49.46324	47.77955	51.04465	24.56561	26.72605	24.81564	3.17E-31	-0.95367
SLC25A32	30.2669	29.43946	27.90813	10.49195	14.70656	12.81231	5.47E-31	-1.32115
RBM24	6.593407	6.348631	6.546616	1.894361	1.998407	2.14283	6.44E-31	-1.6355
PPP3R1	29.42695	27.29655	27.55698	12.28576	12.7581	13.67312	9.57E-31	-1.11786
SRGN	55.80234	56.15007	61.30757	21.26296	21.80149	25.23936	1.24E-30	-1.32213
SEMA3C	18.34705	16.60185	17.31725	5.880544	6.696572	5.512563	4.03E-30	-1.34926
CDCA4	26.79758	27.18217	27.32498	10.42112	10.92754	12.38699	6.91E-30	-1.2441

OSTM1	8.989867	8.851957	9.541959	4.065916	3.748229	4.058576	8.24E-30	-1.19534
SUN1	49.75098	53.71259	45.38528	20.35826	25.3843	23.26214	1.2E-29	-1.21032
TCEAL1	22.11175	17.2901	17.24595	7.70697	6.961827	6.337223	6.95E-29	-1.42974
ARSJ	7.348838	5.964859	6.523873	2.144513	2.71178	2.470695	6.98E-29	-1.34894
TMEM167A	14.24825	13.66037	14.09811	6.998728	6.793293	7.189332	7.03E-29	-1.00393
MTMR4	5.731653	4.894519	5.503007	1.814869	2.3516	2.264308	8.39E-29	-1.43407
SHC1	97.12461	93.449	93.09438	45.20124	50.37817	49.78532	8.69E-29	-0.96825
IPO5	27.9596	28.47363	26.69692	14.10638	14.38398	13.41899	9.26E-29	-1.00361
DYNLT3	10.4342	10.10166	10.94862	4.210216	4.196458	4.144729	3.32E-28	-1.34973
KPNA3	10.27929	9.922277	9.348353	4.544899	4.589352	3.973187	3.86E-28	-1.17499
CENPK	13.22847	12.93681	12.24721	3.132801	4.308562	3.244181	4.64E-28	-1.7839
GABARAPL1	13.41808	14.52679	14.08764	5.793927	5.609308	6.471405	5.57E-28	-1.40614
ACER3	3.095125	3.188405	3.042457	1.403768	1.295152	1.330003	7.14E-28	-1.34477
PGM2L1	1.449122	1.328896	1.333997	0.359239	0.402301	0.31839	9.34E-28	-1.81856
KDM1B	5.091975	5.305512	5.696611	1.218872	1.500253	1.266553	1.1E-27	-1.91829
BIRC3	8.9765	8.21479	7.650458	3.381236	2.785023	2.58549	1.28E-27	-1.45502
RAB18	13.19586	14.13112	13.21509	6.624243	6.725588	6.835261	1.97E-27	-1.00172
CLCN4	1.643039	1.654243	1.79818	0.270899	0.4082	0.391356	2.03E-27	-2.06716
MTX3	3.591093	3.172221	3.249399	1.768451	1.783899	1.190344	2.23E-27	-1.33964
ASAP2	7.576759	6.907536	6.578509	2.225885	2.857077	2.336651	3.22E-27	-1.46804
CBFB	20.41524	20.35312	18.85222	6.890871	7.73454	5.471429	5.46E-27	-1.48557
SLC11A2	14.8244	15.25956	16.38023	9.914783	7.80862	8.190593	5.82E-27	-1.03795
TNFRSF10A	16.94606	14.52407	14.41618	5.272395	6.285357	5.085163	7.47E-27	-1.42607
IMPAD1	22.51526	20.65914	20.43617	10.25817	11.39743	9.592266	8.99E-27	-1.04832
SLC20A1	55.8212	55.07697	54.96985	26.87999	30.34899	29.25604	1.09E-26	-0.96649
RAB3B	7.284594	6.492922	6.418174	2.728925	2.768727	2.102386	1.36E-26	-1.37856

SOWAHC	11.66386	10.6426	10.98314	5.109266	5.291384	4.482429	1.64E-26	-1.15281
PTAR1	9.108173	7.624466	7.203566	3.483466	3.665979	2.645468	1.95E-26	-1.50601
NSUN2	51.37794	49.24675	42.11924	22.89124	21.47856	21.45992	2.09E-26	-1.12781
TAP2	8.814885	10.11751	10.47754	3.901484	4.404969	3.984285	2.27E-26	-1.23753
CNOT11	26.12259	27.8333	28.16505	15.0183	13.74295	13.89682	2.71E-26	-0.95636
MAPK6	16.57918	15.23276	16.12069	6.584673	7.957207	6.922993	3.76E-26	-1.14934
ANKMY2	18.18636	16.64084	16.22232	7.816237	7.953649	7.490295	7.98E-26	-1.11684
PLAGL2	8.303217	7.591846	7.420292	3.508458	3.456738	2.94041	8.52E-26	-1.21892
STARD4	3.589239	2.979835	3.068562	1.107762	1.11394	1.075178	8.57E-26	-1.75611
ZNF41	2.742916	2.202939	2.254151	1.535485	1.015293	0.7685	1.6E-25	-2.39096
MSMO1	43.53156	43.98046	38.38704	18.03536	19.423	18.73788	2.57E-25	-1.17323
GFM1	38.90868	39.58482	35.03025	17.95841	20.1794	18.07922	5.23E-25	-0.96913
WSB2	93.30106	93.42344	92.7495	54.04521	54.21297	55.0107	1.43E-24	-0.83213
TPMT	8.385954	8.67761	7.658602	3.309901	3.59033	3.07761	1.64E-24	-1.28259
KIF21A	22.43393	21.97004	18.93271	9.444958	10.28451	9.583585	2.6E-24	-1.12308
INSIG1	13.26528	11.65511	13.39876	4.782569	5.991433	5.656058	2.91E-24	-1.2622
IMPA1	16.84089	17.93502	17.0366	8.359181	9.349678	7.762737	3.97E-24	-1.13871
NAA20	113.1942	118.4719	119.4946	73.59705	62.01468	67.05653	5.67E-24	-0.85238
GABPA	4.36247	3.969309	4.121811	1.549622	1.769725	1.437508	6.09E-24	-1.34322
SPIN4	4.943042	5.215259	4.169314	1.483756	1.691591	1.635987	7.42E-24	-1.51285
G3BP1	59.81687	42.79146	26.5865	31.49876	18.59114	18.28229	1.14E-23	-0.93283
SPRED1	11.82176	10.05951	10.62419	5.051626	5.355017	4.274687	1.51E-23	-1.15663
IL1RAP	7.838084	7.712685	6.872734	2.590118	3.4911	2.977892	2.11E-23	-1.37833
CDC27	23.17664	19.82941	19.15695	12.0795	13.47602	10.59601	2.21E-23	-0.94777
CCND1	139.1134	136.2727	138.0125	69.15014	79.29709	76.2469	2.88E-23	-0.88449
NDFIP2	4.752372	5.558014	4.801637	1.321543	1.438177	1.430486	2.91E-23	-1.6517

PRKACB	11.19748	9.775894	10.81342	4.395522	4.879783	4.668973	3.04E-23	-1.22992
NCL	278.387	289.7608	275.8538	144.8015	172.4992	163.2467	3.1E-23	-0.88704
BET1	24.36208	26.79758	21.02708	7.77715	6.868331	9.062654	4.35E-23	-1.53088
INPP5F	4.714703	4.408676	3.810432	2.362571	2.024554	1.791155	4.35E-23	-1.28561
CDIPT	26.87911	31.36225	23.46855	6.655445	4.412656	7.625311	4.77E-23	-1.95254
MTRR	9.881795	9.486517	9.642146	4.722337	5.218885	4.955717	6.01E-23	-1.1017
BAG2	10.10868	10.34493	10.65415	5.598313	5.205192	5.66578	8.39E-23	-0.9199
UHMK1	24.10898	21.93385	20.66206	10.33561	11.17314	8.904852	1.19E-22	-1.12184
ADGRG6	3.664738	3.006914	3.101842	1.222898	1.079796	0.881604	2.1E-22	-1.61039
MFSD1	18.9713	26.57475	22.33842	8.254621	10.08968	9.010436	5.34E-22	-1.41323
RYK	14.76074	16.95537	16.90864	7.341099	8.517071	6.805195	6.24E-22	-1.13356
SSR3	37.21162	44.6404	43.06923	18.10707	19.90166	21.83652	7.78E-22	-1.01395
RAPGEF2	11.48605	13.31991	10.75643	4.532859	5.706817	4.865592	1.1E-21	-1.23916
LOXL2	27.89292	25.58984	29.52561	12.59791	14.46004	14.03016	1.72E-21	-1.03254
FEM1C	7.935014	7.961368	7.306895	2.619575	3.536672	2.649478	1.81E-21	-1.35509
CYP24A1	21.99578	20.30965	21.89847	12.02216	11.02736	11.17119	1.83E-21	-0.90103
SLC7A11	41.54996	36.24771	32.96609	14.80072	15.72407	10.91259	2.05E-21	-1.3905
SCML2	2.574931	2.630419	2.117033	0.63662	0.951523	0.784737	2.18E-21	-1.75764
NOC3L	10.80086	9.711715	10.00207	5.151358	5.017905	4.744494	2.41E-21	-1.02706
TMEM123	119.6228	112.8223	114.6146	73.11109	72.84435	67.91801	3.81E-21	-0.71416
ATL2	26.27011	29.13751	23.48508	11.6993	10.83639	10.14523	3.91E-21	-1.16567
CPOX	37.30756	36.60058	37.27827	22.85481	21.34954	20.62861	4.05E-21	-0.7907
SREBF2	18.78513	18.03993	17.7576	9.65824	10.38486	10.37111	4.45E-21	-0.84388
SGPL1	7.333767	6.444595	6.999157	3.106386	3.577725	3.432783	4.81E-21	-1.13041
NUP58	7.715537	8.040838	7.269484	2.317415	3.212612	2.764125	5.38E-21	-1.36547
KBTBD2	17.01362	16.12193	17.19275	8.915002	8.931198	7.547903	6.26E-21	-0.99034

FADS1	34.27963	35.258	36.30966	21.51343	21.60522	21.32516	7.53E-21	-0.74787
OXR1	12.12461	11.59068	15.57219	6.087603	6.8465	8.166373	8.29E-21	-1.12167
DDX3Y	11.07223	9.844075	9.864278	4.752701	4.768159	4.565199	1.09E-20	-1.13555
CASD1	6.011863	5.546245	5.427166	1.599069	2.220747	1.926185	1.61E-20	-1.54525
NDP	1.600342	2.209137	2.697861	0.118744	0.09664	0.205278	1.62E-20	-2.77262
THRAP3	25.65501	24.79688	24.79598	14.79021	15.46089	14.07742	1.75E-20	-0.77122
MFSD9	7.704881	8.531549	8.089143	4.775223	4.182095	3.928963	2.47E-20	-1.12429
PTP4A2	45.10903	48.41587	49.00073	26.00409	25.9025	27.67983	2.66E-20	-0.84863
MED1	15.66588	14.45025	13.60835	5.802045	6.548816	5.18337	2.66E-20	-1.3382
ARL4C	89.87814	82.67106	89.53975	50.88504	53.69322	52.86658	3.85E-20	-0.75287
ARPP19	53.71417	50.72828	52.94299	37.92917	36.89229	34.96872	6.01E-20	-0.71759
CBX5	22.24585	20.16635	19.55238	10.91144	11.33261	9.506923	6.62E-20	-1.02439
FECH	10.96567	12.68416	10.73422	5.359246	5.830276	5.277712	8.92E-20	-1.0577
LSM14B	23.7199	22.83346	22.8761	12.90723	13.12476	12.74654	1.11E-19	-0.81495
STRN	7.928499	7.117821	6.968051	3.481186	3.914494	3.268039	1.92E-19	-1.02602
NIPA1	5.569887	4.763236	4.647272	1.366564	1.091767	1.425392	2.87E-19	-1.81114
KBTBD11	2.411434	1.909177	2.244423	0.701707	0.777002	0.556155	3.45E-19	-1.58495
YWHAZ	655.3565	635.5443	455.2493	431.0689	414.6036	397.5927	3.87E-19	-0.74294
AGFG1	70.55584	66.51491	59.44682	38.19145	29.81644	15.98317	4.29E-19	-0.71384
DEGS1	42.89526	44.75615	44.76272	25.76421	27.22587	24.62784	5.27E-19	-0.77912
TNPO1	44.15944	37.81254	37.9994	23.35851	25.55563	19.69523	5.81E-19	-0.83618
EXOC6B	5.811896	5.028815	4.801325	1.684841	2.324273	1.915243	6.51E-19	-1.32997
MCL1	93.2441	86.46058	88.22351	49.39501	43.41928	40.63908	7.91E-19	-0.95927
DYNC1LI2	15.82476	15.22778	15.18177	9.052386	9.54248	8.555409	8.92E-19	-0.81463
CCNA2	26.83374	28.99108	28.39015	16.04403	16.37053	16.55548	9.91E-19	-0.78935
RPE	22.16522	20.95941	20.20835	11.66675	10.90943	10.43862	1.06E-18	-1.01866

SPCS3	24.23807	24.51232	25.9616	13.68107	14.73635	14.75982	1.14E-18	-0.79758
CIPC	2.265748	2.502096	2.060084	0.699914	0.495584	0.65394	1.28E-18	-1.71312
MTMR6	2.766779	2.83773	2.828365	1.049333	1.27452	1.120099	1.82E-18	-1.25192
SPOPL	2.804689	3.438055	2.476344	1.241171	1.509421	1.255682	2.25E-18	-1.41413
CCDC6	14.56431	13.15987	13.03625	6.905591	7.544363	6.145671	3.06E-18	-0.97889
DUSP5	44.79121	41.96184	42.9837	21.36766	24.39772	24.60231	3.48E-18	-0.88281
HEATR3	8.537539	8.090407	8.657671	4.543245	4.447668	4.336493	4.99E-18	-0.92249
CHMP3	30.07757	31.48601	28.80084	16.24634	17.62192	17.55134	6.9E-18	-0.8404
GARS	72.08261	75.52407	70.79706	45.51598	42.47635	44.8303	1.11E-17	-0.65572
ACSL4	26.65087	23.13815	23.91711	14.95645	13.81764	13.09186	1.24E-17	-0.91073
ATP6V1C1	36.99153	36.6022	36.91336	34.42245	30.45066	29.6539	1.7E-17	-0.82442
RBM15	9.025878	7.925065	6.814704	3.427954	2.838424	2.67792	2.16E-17	-1.33601
WEE1	9.163701	14.56589	13.75213	3.811411	3.990032	4.376202	2.91E-17	-1.64656
AGO2	16.35505	13.94398	13.94083	7.056006	8.507078	6.849019	3.2E-17	-0.94604
ACAT2	21.70306	22.02354	21.87402	12.5341	12.67861	13.46653	3.48E-17	-0.76834
HIF1A	76.83529	70.47671	69.58522	36.58319	42.17984	32.97167	4.03E-17	-0.95251
TRIM44	10.83646	10.30052	9.668257	5.532959	6.2437	5.453698	4.25E-17	-0.84113
FAM136A	55.73652	56.41453	57.3703	29.79557	31.40548	34.24843	4.73E-17	-0.82863
LNPEP	3.726953	3.513608	3.300715	1.249776	1.619048	1.026977	5.09E-17	-1.37031
EI24	67.0332	73.93672	77.27892	44.81265	45.63562	46.06135	6.28E-17	-0.72798
FMNL2	10.89688	9.250035	8.716004	4.69085	4.80507	4.102136	7.03E-17	-1.0679
EEF2K	7.449889	6.452866	6.665805	3.837879	4.109549	3.618809	7.24E-17	-0.89767
Homo_sapiens_newGene_16497	4.606889	4.413771	5.969942	1.647341	1.90668	1.744482	7.69E-17	-1.39266
CPEB4	2.396564	2.606552	2.312761	0.936398	1.652871	0.832327	7.69E-17	-1.59315
SLC5A3	3.555299	2.952891	3.024264	1.247619	1.207809	0.791129	8.05E-17	-1.46609
HMGCS1	19.82673	22.19371	21.64977	11.10676	12.47705	11.36339	1.03E-16	-0.86326

ZNF367		13.31506	13.9844	14.78643	7.273821	8.178779	7.218453	1.41E-16	-0.89125
SUCLA2		12.5955	12.12384	11.74449	5.375322	5.067763	6.009795	1.64E-16	-1.23871
RPL23A		363.8268	388.2195	380.262	231.5468	213.1132	239.8081	1.8E-16	-0.72203
ARL6IP6		11.14733	10.44345	10.43118	5.05542	4.896258	5.757687	1.82E-16	-1.02605
RPAP1		7.3845	6.715033	8.226437	3.772675	4.205421	3.633325	1.95E-16	-0.94312
F3		23.37973	20.42126	21.58859	6.964597	9.847327	9.359399	1.97E-16	-1.26652
CMAS		46.62217	47.5994	51.13067	25.78902	26.86149	28.77567	2E-16	-0.84691
RMI1		11.37784	9.376592	8.97182	4.512714	3.603985	4.043521	2.11E-16	-1.28064
UBA5		10.8663	11.52649	10.95958	4.788308	7.539801	5.649494	2.5E-16	-1.00624
GBP1		5.965701	5.596421	5.700931	2.675035	2.611281	2.831291	2.82E-16	-1.06599
YOD1		5.806047	5.655834	5.211443	2.777705	2.756347	2.162854	3.83E-16	-1.09146
ICK		6.324473	6.055271	5.772827	3.079903	3.212217	2.53991	4.14E-16	-1.02437
EIF5A2		6.651932	6.205779	6.284606	3.439471	3.429292	3.348607	5.02E-16	-0.88243
IPO8		12.06407	12.07273	10.76796	6.668486	5.263856	5.013671	5.66E-16	-1.03897
	44082	7.478356	7.793283	7.019019	3.379082	3.631173	3.408889	7.32E-16	-1.16328
ENTPD7		7.332263	6.010925	6.540262	2.088161	2.943421	2.182554	8.44E-16	-1.39518
RAD51AP1		31.42594	23.38833	25.75236	13.22828	11.07605	14.01113	1.02E-15	-0.97945
ZDHHC21		0.631452	0.523198	0.490443	0.115248	0.130117	0.125988	1.17E-15	-1.86217
CNIH1		20.60832	21.51056	22.46148	18.40413	17.89039	14.04872	1.26E-15	-0.76527
SAMD5		8.678831	7.626638	8.155488	3.559248	4.192167	2.914772	1.58E-15	-1.16252
RDX		37.65006	43.42393	42.58406	22.29322	24.81245	20.96097	2.38E-15	-0.87478
DLGAP5		10.61486	10.97102	10.26757	6.067578	5.75058	5.200274	2.63E-15	-0.88492
SORBS3		27.30915	26.54208	29.31085	14.29817	11.53609	14.32198	2.76E-15	-0.97968
BACH1		8.493102	9.03717	7.307593	4.426062	4.650349	3.658126	3.35E-15	-1.03436
WDR44		16.3697	14.8689	15.43604	9.057102	9.374413	8.154789	3.52E-15	-0.80263
CKAP2		9.12677	8.741675	9.671244	4.823429	4.256554	3.643732	3.71E-15	-1.10599

SQLE	50.98898	47.91178	44.45987	23.76758	22.7918	27.90547	4E-15	-0.93881
GFOD1	2.862937	2.481991	2.903987	1.120257	1.213916	1.591291	5.25E-15	-1.04811
AEN	37.10089	36.75949	34.90144	22.33849	23.46721	22.34596	5.35E-15	-0.66253
GNPNAT1	19.00389	20.18081	19.74256	10.55325	11.96621	11.37053	5.98E-15	-0.752
TMEM167B	6.912259	7.039153	7.063794	3.774759	3.770813	3.147627	6.91E-15	-0.96307
ARMCX6	13.30957	15.34839	15.21913	7.654574	7.649048	7.283055	8.31E-15	-0.94875
ARNTL2	14.45347	14.68413	13.36949	6.589275	9.191108	7.866166	1.02E-14	-0.96513
GULP1	10.25835	11.00815	11.06925	5.060496	5.554861	5.455069	1.1E-14	-0.9657
IST1	30.52334	28.16011	23.73543	11.36104	11.61353	10.34614	1.12E-14	-1.20657
TRPM7	6.451991	6.301129	5.476909	3.006068	3.827423	2.910405	1.43E-14	-0.93567
PLSCR3	10.09458	9.821503	7.827215	4.654759	2.872148	3.382321	1.58E-14	-1.36057
CEP55	48.90282	50.01187	46.40598	25.66005	29.32371	29.54343	1.6E-14	-0.7853
STK38L	10.3976	9.805267	9.947583	5.739444	6.232678	5.998526	1.76E-14	-0.82189
HLTF	10.17434	10.43132	9.563752	5.198139	5.894264	5.564802	1.79E-14	-0.88421
PHTF2	24.89809	24.88267	21.79815	16.61914	15.77644	16.30132	2.15E-14	-0.64873
SAR1A	24.53507	23.23443	24.26388	14.12154	13.42295	15.50966	2.51E-14	-0.77878
NCF2	2.659327	2.470366	2.257126	0.082403	0.213897	0.517841	2.64E-14	-2.28945
RADX	6.882922	6.790021	6.377358	3.096515	3.64213	2.786542	2.92E-14	-1.0728
HK1	56.11864	52.92432	57.30827	33.854	37.37098	35.096	3.01E-14	-0.66123
TRAK2	5.135375	5.033579	5.331987	3.135163	2.989747	2.805375	3.08E-14	-0.81915
GLS	24.47036	22.85467	22.95781	14.84188	15.85935	15.11057	3.92E-14	-0.62487
TRIP13	22.13151	21.91917	23.27708	11.54863	13.2258	13.34422	4.48E-14	-0.81948
INIP	7.211404	9.517282	8.855678	4.194379	4.26897	4.087782	4.55E-14	-0.87402
MEST	16.10456	16.04321	16.18165	9.827129	10.08613	9.373134	4.92E-14	-0.73266
PRKAG2	9.131658	9.316425	10.79351	4.72394	5.639905	5.834415	5E-14	-0.95598
TOMM20	67.20547	70.59664	70.43481	47.55357	44.8702	46.65873	5.77E-14	-0.59544

TMEM184C	17.63875	16.93259	17.22371	11.35374	10.59925	9.813076	5.85E-14	-0.71496
MIGA1	6.713398	6.627362	5.250706	2.90705	3.60607	2.680474	7.15E-14	-1.0635
PRKAA1	11.41797	11.50502	10.78999	5.974282	6.751081	5.146386	7.25E-14	-0.94067
TMEM65	7.18335	6.725716	7.016698	4.422152	4.410115	3.973945	7.73E-14	-0.71612
NR1D2	16.77191	17.13018	15.50977	9.719325	10.71407	9.618622	7.81E-14	-0.72988
DENND6A	7.417016	6.177359	6.32716	4.426109	4.148103	3.657603	8.58E-14	-0.86062
DNAJB1	65.06618	65.5985	67.3208	45.03189	44.42218	40.6795	8.6E-14	-0.6166
GTPBP2	7.119647	6.819847	7.090555	3.270126	3.181738	4.079252	1.04E-13	-1.0432
RAB2B	3.09146	3.115138	2.570623	1.059878	0.865841	0.999733	1.09E-13	-1.40435
GTF2A1	8.254318	7.514576	8.195298	4.602631	5.044914	4.673191	1.16E-13	-0.73887
RUFY2	6.389568	5.752575	4.930779	2.72725	2.59066	2.642966	1.2E-13	-1.09442
HMGN3	20.03348	20.77726	23.48129	10.0173	9.754955	10.88128	1.64E-13	-1.04024
FRS2	9.501539	11.44444	10.09513	3.880956	5.12377	3.854337	1.68E-13	-1.06981
OTUD4	16.95606	14.13736	13.42535	7.362926	8.642451	6.555514	2.3E-13	-1.0068
GNG12	17.41603	17.56238	17.15998	10.71003	11.71763	10.48114	2.6E-13	-0.6733
MTMR9	3.299303	2.465097	2.455044	1.276433	1.176106	1.150483	3.51E-13	-1.06559
CHFR	18.7826	19.49096	19.60222	10.58693	11.87202	11.72118	3.64E-13	-0.7814
MAN2A1	14.76396	11.96924	12.72323	6.696976	6.803632	5.083696	3.79E-13	-1.05819
CLOCK	6.215948	5.483184	4.833722	2.385078	3.126217	2.501648	4.16E-13	-1.13711
HSD17B4	16.88602	15.8896	21.17297	10.17075	9.876658	9.435896	4.17E-13	-0.73575
HIPK3	13.34142	11.80737	11.55838	7.041966	6.978996	5.557452	6.53E-13	-0.90181
FAM126B	2.430783	1.823126	1.620177	0.590078	0.771707	0.452125	7.52E-13	-1.45367
SNX16	3.386069	3.133492	2.911372	1.156682	1.149191	1.415915	8.79E-13	-1.41436
ADIPOR1	42.93837	44.29485	43.983	27.92338	29.16892	24.77856	9.01E-13	-0.69033
CHMP2B	70.58084	79.33971	81.57708	43.51065	51.02797	46.96428	9.11E-13	-0.84026
ERCC6L	7.951437	7.058427	6.666328	3.848551	4.10389	3.535043	9.38E-13	-0.90204

GCC2	6.393803	6.38629	6.41249	3.950153	4.439037	3.525884	1.03E-12	-0.83568
RAB1A	71.59407	69.76597	60.20862	45.59993	39.68786	48.74541	1.03E-12	-0.66864
CISD1	13.52626	13.60797	13.7572	8.045673	7.037371	8.107063	1.15E-12	-0.81545
RC3H2	14.26861	12.18252	8.282001	7.521554	7.132274	6.498641	1.28E-12	-0.98009
PANX2	4.877983	5.569793	5.695592	2.378375	2.128975	2.49254	1.64E-12	-1.14472
RAB27A	6.222607	6.211638	6.840766	3.455708	3.27017	3.216854	1.82E-12	-0.87594
THAP5	12.7885	11.88544	10.82448	5.898362	5.848984	5.643667	1.93E-12	-0.85389
MAPRE3	20.9217	20.96057	17.01007	9.456418	9.105046	10.45798	2.03E-12	-0.9901
XPO1	67.01318	63.49806	56.67646	35.87082	37.27725	33.98922	2.41E-12	-0.66894
KLHL5	33.12888	28.15199	26.7409	20.53925	17.97177	16.34656	2.46E-12	-0.73538
PLCXD2	4.330872	4.271847	3.498971	1.523661	2.044582	1.742864	2.69E-12	-1.09508
GBA2	19.67119	18.83308	18.91991	13.40644	12.98385	11.85359	2.78E-12	-0.65425
PARD6B	13.65076	12.40973	12.91102	7.848026	7.994533	7.421515	3.07E-12	-0.81031
ERO1A	9.687148	9.703666	10.06329	8.016826	8.603018	7.196596	3.9E-12	-0.7414
SMC2	10.7746	10.85374	10.56068	6.216256	6.803939	5.450583	4.29E-12	-0.80877
PTCH1	4.079058	5.085064	3.851122	1.79288	2.836701	1.598213	4.73E-12	-1.0533
SHCBP1	24.27875	24.90097	24.6174	15.97412	17.01171	16.23468	5.51E-12	-0.61689
MRPS17	17.70673	17.07312	17.47656	10.24392	9.477661	10.78488	5.63E-12	-0.78102
RAB27B	13.82501	12.81213	12.90891	8.748221	8.180927	7.219507	6.69E-12	-0.78027
RGPD6	4.56442	4.625857	4.639326	2.559291	2.290737	2.077101	7.06E-12	-1.03833
SGPP1	5.917478	4.490981	4.576412	2.256473	2.288659	2.099186	7.58E-12	-1.12473
CCSAP	3.393229	3.419591	3.428414	1.859235	1.954253	1.738079	8.29E-12	-0.87295
KLHL23	1.805701	1.593877	2.144243	0.800727	0.626572	0.531481	9.27E-12	-1.64425
C1GALT1C1	13.27914	14.84317	14.09754	7.947792	7.349044	8.003788	9.31E-12	-0.84804
MFSD6	2.824887	2.650981	2.614496	0.914518	1.276509	0.842706	9.39E-12	-1.20999
LSM11	1.81191	1.578429	1.813332	0.782836	0.807308	0.819006	9.84E-12	-1.06763

CDC6	29.83413	31.82767	31.91443	18.73549	17.67968	19.97907	9.96E-12	-0.72248
RAB11FIP2	4.167715	3.49985	3.417639	1.328316	1.906521	1.386859	1.06E-11	-1.19533
SLC19A2	6.59315	6.360991	6.001145	3.488715	3.868033	2.773787	1.14E-11	-0.94232
FGF2	6.237356	5.874445	5.047178	2.929284	3.282708	2.602343	1.16E-11	-0.99639
ANAPC16	12.75846	13.59927	12.71404	8.093117	8.182948	8.367302	1.18E-11	-0.67454
SNX24	4.836359	3.693586	4.650316	1.625248	1.665535	2.799391	1.22E-11	-1.44879
C12orf4	10.56824	9.739733	9.667588	4.55724	5.624994	5.765092	1.34E-11	-0.84236
RGPD8	3.550859	3.360179	2.837991	1.33243	2.0163	1.134494	1.45E-11	-1.04745
RCAN1	15.23863	14.5758	11.92251	6.893516	7.25179	7.339698	1.47E-11	-0.91049
VDR	6.041012	5.696419	5.161799	1.573372	2.795959	2.328482	1.55E-11	-1.096
SMIM13	4.481725	4.329517	4.18933	2.422983	1.883112	1.708157	1.56E-11	-1.07222
ELMSAN1	11.35328	10.95906	9.904436	6.12056	6.660741	5.559598	1.67E-11	-0.73689
BHLHB9	3.238935	3.374683	3.254836	1.457981	1.669095	1.699145	1.69E-11	-0.99856
ARHGEF10	5.844513	5.180374	5.917323	2.860811	3.330633	3.346007	1.76E-11	-0.84145
EPHA2	51.71989	43.66747	45.67346	25.91855	30.17494	27.87429	1.88E-11	-0.74939
NUP188	27.89024	24.43793	23.55746	14.10058	16.27403	14.77255	1.99E-11	-0.75016
CARD19	27.28097	29.8809	30.76416	8.580099	7.175841	13.18709	1.99E-11	-1.3363
FAM198B	13.11818	12.59412	12.71541	7.56751	7.884911	6.057211	2.02E-11	-0.81633
ZCCHC10	7.039029	7.596799	5.98032	3.477768	2.188537	1.602965	2.19E-11	-1.48661
TAP1	7.339839	7.420756	8.056802	4.046252	4.239741	4.447639	2.58E-11	-0.83347
PURB	9.025339	8.475496	8.374969	5.30374	5.690684	5.743305	2.8E-11	-0.63697
HAT1	48.84898	51.39066	51.34627	31.38014	26.89838	32.32214	3.07E-11	-0.74307
SEC24D	8.494981	8.432493	8.77424	4.176253	5.342668	4.501257	3.51E-11	-0.86265
PYURF	55.43224	57.90345	56.39559	38.54265	33.66727	36.63901	4.12E-11	-0.64876
ZDHHC17	6.180944	4.898178	5.125258	3.379234	3.880966	3.133892	4.71E-11	-0.80717
PREP	11.12373	10.71946	11.40913	6.379931	4.632369	7.93417	4.85E-11	-1.02162

SNRPD3	48.87669	65.19426	62.83256	31.76618	28.05584	31.33653	5E-11	-0.92944
APOL6	2.651716	2.415776	2.323042	1.245072	1.43622	1.087921	5.14E-11	-0.94867
PFN2	73.77328	77.11352	76.01695	46.2541	50.946	48.48445	5.14E-11	-0.65626
ZBTB33	14.00491	11.99122	10.9872	7.092353	7.292213	6.770395	5.28E-11	-0.80961
AP4E1	3.003888	2.473489	2.375972	1.86534	1.89987	1.095606	5.38E-11	-1.14992
RBPMS2	7.342079	9.843316	8.378478	2.372323	3.3066	3.781612	5.38E-11	-1.26478
GNA13	58.82754	54.98437	53.40198	34.64964	39.01143	32.8903	5.58E-11	-0.66145
Homo_sapiens_newGene_4925	3.138613	2.478959	2.131828	0.83472	0.826132	0.717056	5.64E-11	-1.50768
SLC26A2	0.76741	0.975979	0.684889	0.302604	0.26454	0.221309	5.87E-11	-1.38322
USP46	2.379063	2.854683	2.309152	0.685662	1.209177	0.978755	5.98E-11	-1.29303
MORC4	61.86181	61.17106	58.61087	35.68161	42.2288	39.26199	7.26E-11	-0.64772
Homo_sapiens_newGene_16135	6.288061	6.116919	6.232737	2.902456	2.138687	2.74479	7.38E-11	-1.25564
TUBD1	6.466142	5.382719	5.520253	2.488615	2.130697	2.336288	7.87E-11	-1.12398
PRDM1	2.715328	2.396633	2.858109	1.264971	1.432215	1.199537	8.11E-11	-0.99408
FAM220A	9.807083	10.18494	9.321479	5.649728	4.747351	5.492578	8.39E-11	-0.87112
SREK1IP1	2.436548	2.539484	2.433381	1.422019	1.081547	1.188111	9.56E-11	-0.97646
PPP1CC	80.47932	91.81699	79.92663	53.25435	50.19107	55.45184	1.06E-10	-0.68549
COX7A2L	37	47.86542	47.27987	29.0114	27.61552	25.4222	1.19E-10	-0.70049
BAX	106.7446	117.4919	111.0928	66.65822	64.68192	73.76826	1.2E-10	-0.70292
PCSK6	9.797878	7.600414	8.673409	5.27625	5.668654	4.571962	1.43E-10	-0.76898
DHCR7	43.50794	35.44779	41.42539	20.72864	18.09447	18.66029	1.43E-10	-0.93296
CEP97	2.891288	2.545895	2.174169	1.003531	1.360991	1.419131	1.59E-10	-1.09853
SERTAD2	23.75719	21.63441	20.14284	10.89255	14.00876	10.78663	1.62E-10	-0.86399
CDK6	12.57552	10.94926	9.351342	6.10655	6.831169	5.602173	1.64E-10	-0.89079
MVK	12.75115	12.15391	12.45875	7.480469	7.90275	7.663496	1.73E-10	-0.76398
SLC7A5	313.3391	297.1553	300.9046	130.5171	75.07403	145.5702	1.8E-10	-1.27156

RAD1	8.497853	6.400993	6.200901	6.628508	6.260266	2.230433	1.8E-10	-0.81563
CLIC4	21.99539	21.27961	22.0923	14.59685	14.05015	11.77424	1.87E-10	-0.69811
LPIN1	2.245588	1.925706	1.852425	0.908318	1.058488	1.018367	1.99E-10	-1.06737
LRRC59	102.5718	106.0634	109.4681	62.66976	70.57056	73.32314	2.03E-10	-0.64544
RGPD5	3.759609	4.224288	3.983339	2.73006	1.595774	2.309284	2.05E-10	-0.98837
SLC43A3	5.906282	6.229545	5.462065	1.972729	2.382826	2.993865	2.09E-10	-1.15973
ANLN	72.66121	65.90334	66.9992	44.94278	46.1034	39.53627	2.2E-10	-0.65477
AREL1	6.879568	6.630849	7.190785	3.418638	4.030425	3.267237	2.51E-10	-0.88823
ZSCAN25	14.23848	10.63966	11.11799	7.431986	8.149695	7.941595	2.76E-10	-0.72213
MPST	23.90412	29.52179	27.96863	12.99731	7.816951	12.28224	3.03E-10	-1.17619
CYTH4	3.175506	1.989826	2.498714	0.84998	0.910229	0.969657	3.03E-10	-1.29418
DPY19L4	11.40593	9.526063	11.5193	7.494471	6.207956	4.385621	3.35E-10	-0.96507
FAM3C	28.07145	27.02363	25.31987	14.17328	15.52677	17.54748	3.98E-10	-0.76073
UBASH3B	28.90571	26.30843	27.22947	14.22931	18.46521	15.8695	4.5E-10	-0.76041
SLC16A6	4.38271	3.504933	3.206278	1.757175	1.773936	1.905121	5E-10	-1.23226
CCNH	17.31402	15.36931	14.78209	8.753974	9.586015	9.94904	5.1E-10	-0.79664
RASSF8	5.197741	4.419874	6.315344	2.992074	2.923313	4.106702	5.15E-10	-0.77546
LEPROTL1	12.42852	11.64056	12.1491	8.564748	7.601353	8.041526	5.52E-10	-0.63616
NFU1	17.92232	18.45881	20.62011	6.487596	8.394645	10.2107	5.79E-10	-1.09496
PIK3C2A	15.5336	13.49208	12.83607	7.723339	8.0109	5.570572	6.05E-10	-0.95136
MPV17	28.11762	32.3186	32.47694	15.80627	12.58889	16.81756	6.28E-10	-0.97876
HAUS6	4.986204	4.604542	4.11638	2.216249	2.731826	2.015705	6.37E-10	-0.94768
ABT1	10.26331	10.27989	10.18031	6.334494	6.166654	6.732177	6.39E-10	-0.67878
EOGT	6.046958	4.926733	5.867849	3.008654	3.317833	2.620379	6.53E-10	-0.9181
SLC25A30	1.642449	1.590182	1.40967	0.586483	0.482519	0.388027	6.81E-10	-1.37732
C5orf51	5.316044	4.885446	5.009355	3.140126	2.843904	2.398362	7.15E-10	-0.84739

RAB8B	6.531288	8.295492	6.705046	3.300155	4.259683	5.091741	7.5E-10	-0.89225
DUSP2	5.366708	6.601095	7.20129	2.966496	2.28502	2.726433	8.15E-10	-1.17779
CYP51A1	35.09091	34.02932	31.38301	18.85356	22.67843	21.2554	8.58E-10	-0.68784
CHST11	8.718656	8.429015	8.22692	4.195742	5.767102	4.820464	8.65E-10	-0.80757
SMAD5	7.001749	10.59787	5.276434	3.516596	3.513333	2.293811	9.23E-10	-1.05184
SGK3	1.855036	1.873548	1.893181	0.910724	0.850084	0.816029	9.5E-10	-1.14973
PLS1	44.87174	36.8241	33.86551	25.24786	33.80709	22.22182	9.97E-10	-0.67654
Homo_sapiens_newGene_4442	1.195289	0.923825	0.898452	0.441748	0.357161	0.289897	1.01E-09	-1.33115
RNFT1	5.008274	4.432368	5.421854	2.079328	2.261719	2.19144	1.06E-09	-1.20582
RPS6KA3	19.93001	19.18748	18.48315	13.11337	14.36972	16.41514	1.09E-09	-0.73985
MICB	9.683811	8.953062	8.072781	4.940791	5.292447	4.851388	1.13E-09	-0.81407
TRAF5	4.160428	4.066768	3.604469	1.787209	2.260621	1.939911	1.16E-09	-0.95184
SELENOT	39.58638	24.745	23.87453	22.37345	13.3873	14.50108	1.23E-09	-0.95162
RAB23	3.911829	4.248953	4.018784	2.842623	2.513815	2.252886	1.33E-09	-0.76394
44085	18.62842	17.12943	17.41408	9.394447	8.930441	8.731203	1.33E-09	-1.35115
PLEKHA3	2.587276	2.124303	1.690822	1.305056	1.640206	1.232692	1.62E-09	-0.70303
PIK3R3	1.842302	1.995384	2.012308	1.683037	0.953905	0.79516	1.81E-09	-0.9839
EBP	59.45148	58.21781	56.70958	30.7049	38.14616	49.9541	1.98E-09	-0.76054
PLOD2	74.22201	67.54207	74.24063	39.58079	52.5668	45.41496	2.37E-09	-0.66493
AK3	5.1118	4.107674	5.358437	1.693084	2.467514	1.965162	2.75E-09	-1.13082
FOXN2	7.192623	6.466347	6.008573	3.617443	4.353521	3.167091	3E-09	-0.85343
KLHL2	8.330121	6.254451	6.672258	3.960738	3.91429	3.7179	3.43E-09	-0.86367
CCNG1	14.87387	23.51591	22.82989	8.501652	6.502466	10.12061	3.91E-09	-1.22729
PLSCR4	1.630323	2.415274	1.789239	0.387722	0.895193	0.361014	4.67E-09	-1.62052
DUSP6	23.6085	20.64878	22.96007	21.34382	15.23633	16.75689	5.06E-09	-0.62811
NT5DC3	2.98057	2.643445	2.914629	1.602561	1.785593	1.474963	5.1E-09	-0.8126

ACKR3	36.87105	37.95029	36.67825	13.44824	20.8354	20.69069	5.29E-09	-0.97815
SLC39A8	2.616396	2.786258	2.704596	1.024824	1.396602	1.140935	5.31E-09	-1.10245
SH2B3	19.8396	17.8255	17.6292	10.23231	12.58278	10.70078	5.33E-09	-0.71027
JAG1	77.62176	68.72722	71.97927	44.29493	52.40308	42.99747	5.5E-09	-0.64109
GSPT1	102.14	47.32067	52.57575	29.7762	32.71471	32.24762	5.94E-09	-0.62829
MYBL1	7.670222	5.868893	5.127059	3.139787	3.196681	2.974603	6.02E-09	-1.15696
AKIRIN1	21.72487	20.8689	21.00577	12.09222	11.95219	8.161904	6.46E-09	-0.90369
DCUN1D1	6.283023	6.043633	6.32288	4.236383	4.846825	7.300312	6.66E-09	-0.59778
NUP153	20.93873	17.55902	17.27495	10.81096	11.83247	8.62629	6.77E-09	-0.83061
SPRTN	6.174165	6.259628	6.341007	3.739565	4.096248	3.839907	6.95E-09	-0.6861
SSH2	11.81766	11.90409	16.40928	11.95345	13.31101	11.66577	7.08E-09	-0.67848
DNAJC24	5.586182	6.045963	5.281871	3.581499	2.932606	3.080292	7.64E-09	-0.95595
LSM5	21.19292	25.84803	24.27052	14.44454	14.17761	16.39611	8.09E-09	-0.66409
VPS13C	3.704049	3.159374	3.05521	1.941409	2.222412	1.351298	8.47E-09	-1.09538
KRAS	11.62933	10.66697	9.302357	6.551438	6.285542	5.280402	9.38E-09	-0.75975
AKNA	2.894185	2.92409	2.773702	0.861605	1.785789	0.794344	1.16E-08	-1.14159
BICDL1	8.816837	8.60556	8.447432	4.97009	4.917571	5.300036	1.16E-08	-0.73563
KLHL15	1.438135	1.24167	1.129587	0.509231	0.591527	0.585337	1.17E-08	-1.0972
RFK	14.97052	15.29382	15.36079	9.736553	10.82493	9.319748	1.18E-08	-0.6181
SLC37A1	8.413612	8.596269	9.186411	4.64811	5.715517	6.366717	1.21E-08	-0.71721
SNX13	6.973898	5.970138	6.498463	4.357006	4.666211	4.561711	1.23E-08	-0.63972
NHLRC2	3.357125	2.971671	2.781974	1.858569	1.946528	1.778848	1.32E-08	-0.70431
MCTS1	48.58996	57.63082	53.95218	32.51991	27.64231	34.16933	1.32E-08	-0.75578
STAM2	4.841128	4.450336	4.582228	2.995722	3.047707	2.90159	1.38E-08	-0.6376
GTF2E1	8.638707	9.044168	8.577883	5.499206	5.82032	5.234557	1.64E-08	-0.64266
PRKCI	13.37829	12.46668	12.45304	8.870935	8.30972	7.081921	1.68E-08	-0.66187

LIF	8.750734	8.070372	7.965643	3.268769	5.12505	4.053661	1.75E-08	-0.95133
GPATCH11	9.604715	9.970986	8.780269	5.02262	6.194353	5.487757	1.98E-08	-0.74422
SLC25A33	6.751038	6.825573	6.410794	3.726191	4.097512	4.367086	2.12E-08	-0.70907
SLC39A10	8.893613	9.075126	9.017321	5.823404	6.376621	5.615431	2.24E-08	-0.60769
CACNB3	10.66505	9.63748	11.10514	7.169995	6.79402	6.259087	2.36E-08	-0.78222
E2F2	4.228106	3.947171	3.875808	2.185095	2.536176	2.46395	2.41E-08	-0.73955
NAB1	11.03896	10.77618	9.257512	6.309021	5.897212	5.966806	2.45E-08	-0.75905
GNE	19.00715	19.28079	18.74454	13.58157	13.23438	10.87174	2.58E-08	-0.58915
RASSF6	2.861672	3.554666	3.439037	1.539644	1.804689	1.609813	2.77E-08	-1.04966
XPO4	2.779401	2.779082	2.905991	1.511137	1.888372	1.407259	2.87E-08	-0.80207
PDK1	3.45566	3.345892	3.601607	2.674334	3.70266	2.377926	2.94E-08	-0.72285
CDCP1	2.284791	2.204998	2.180408	0.764417	1.226003	1.033298	3.18E-08	-1.06787
CDC7	7.434969	7.330575	7.245965	4.234734	4.620009	3.106058	3.28E-08	-0.97014
CDKAL1	3.036783	3.501205	3.764851	1.573835	2.042044	1.536096	3.53E-08	-0.98205
ITGA2	11.63314	11.41369	6.028987	2.678464	4.52297	2.948847	3.64E-08	-1.31121
AP1G1	11.85162	15.45554	11.69752	7.73587	8.947094	7.774609	3.75E-08	-0.59455
EXTL3	7.638021	6.743568	7.741414	4.899378	4.922411	4.31478	3.76E-08	-0.66856
TLR4	2.167057	1.914316	2.004099	0.727885	0.831781	0.839804	3.77E-08	-1.22379
ZNF106	5.367668	5.128021	5.025661	3.43579	3.384657	2.807858	3.94E-08	-0.67424
KATNAL1	1.779561	1.39428	1.68342	1.167983	0.860026	0.896811	3.97E-08	-0.97002
CMC1	12.39647	15.04907	13.91764	10.40319	8.211107	9.801507	3.98E-08	-0.72465
CDKN3	33.98914	35.16254	30.34658	20.75933	20.78553	18.10194	5.17E-08	-0.74468
AKT3	0.67059	0.588865	0.743314	0.106132	0.450432	0.211198	5.3E-08	-1.51988
ACOX2	0.80541	0.816521	1.489494	0.228215	0.06145	0.104751	5.42E-08	-1.84359
CLN8	8.421257	7.703117	7.35945	3.754788	3.468944	3.510511	5.68E-08	-0.76589
N6AMT1	2.904657	4.175813	4.555515	2.658867	2.676484	3.108661	5.97E-08	-0.74073

RPS6KA5	0.162859	0.125876	0.316106	0.194261	0.030752	0.121479	6.14E-08	-1.52846
ZMAT3	3.173801	2.970487	2.350965	0.906848	1.40111	0.728117	6.14E-08	-1.30439
PHF6	18.13407	18.3066	17.34105	12.67562	12.5163	11.28932	6.16E-08	-0.60114
PRKCH	0.570709	0.40451	0.764762	0.101352	0.049875	0.161173	6.59E-08	-1.82514
ADAMTS1	2.722146	2.607849	2.341195	1.029481	1.349089	0.914464	6.62E-08	-1.17698
CLCN5	4.116919	4.466888	4.398046	1.898159	2.309375	1.654966	7.8E-08	-0.94419
MMD	13.67338	13.78937	15.63424	10.69242	8.63008	8.741108	8.35E-08	-0.61894
CHKA	28.62913	32.34533	31.89088	22.15233	21.01592	20.39203	9.18E-08	-0.71728
POLR2M	6.677891	6.432548	6.978505	4.730066	4.606255	4.26341	1.12E-07	-0.62665
TANGO2	13.88004	16.02649	15.57923	9.782885	5.642712	7.453827	1.16E-07	-0.9867
NXT2	7.771702	7.713079	9.207983	4.144367	4.675056	5.190834	1.17E-07	-0.78131
THBS1	54.27384	45.95338	44.96297	29.11533	34.34945	26.96359	1.21E-07	-0.66104
GNPTAB	7.301964	7.234548	6.356992	4.527867	5.091915	4.773515	1.23E-07	-0.62431
ZNF740	6.134509	5.096099	5.470755	3.654089	3.76404	3.517242	1.23E-07	-0.61483
SHOC2	9.032475	9.041368	8.152577	6.0766	4.946543	4.710787	1.26E-07	-0.72575
TXNDC16	1.569643	0.816624	1.07897	0.255509	0.163775	0.339527	1.34E-07	-1.5047
PLEKHB2	25.90655	24.71373	23.08337	16.03837	15.72134	13.56735	1.36E-07	-0.65093
FOXO3	8.740203	7.737643	7.466384	3.438148	4.601373	2.238152	1.41E-07	-1.12126
CCNE1	13.52983	12.8402	11.98887	7.745707	6.632103	7.168484	1.46E-07	-0.7479
CNNM3	7.233498	6.648169	6.357797	4.18103	4.517895	4.082765	1.52E-07	-0.66174
ADAMTS12	9.162875	7.843975	9.612967	4.911541	5.415758	8.769975	1.64E-07	-0.77679
SAMD9L	4.4761	4.417044	4.052305	1.374483	2.779688	1.54842	1.72E-07	-1.07088
CSF2	4.162037	2.957087	3.743353	0.533932	0.718849	1.18978	2E-07	-1.60233
PHF5A	18.41731	22.69574	21.40085	12.37548	10.56727	12.79777	2.12E-07	-0.78686
MYD88	8.922725	22.80884	16.00481	7.720352	9.856077	5.138862	2.52E-07	-1.1547
EREG	37.94881	32.85989	32.31736	19.30501	24.6478	19.05936	2.54E-07	-0.70489

FAM45A	9.831961	11.89525	10.35659	6.189843	5.461279	6.087736	2.55E-07	-0.75508
GJB3	3.831028	3.160616	4.497537	1.561588	1.971153	1.622298	2.57E-07	-1.08043
GNPDA2	6.634644	6.14784	7.700007	2.129417	3.0495	2.055516	2.58E-07	-1.06849
PURA	1.300963	1.281956	1.268147	0.849913	0.7139	0.712024	2.64E-07	-0.74629
HINT3	7.513506	6.903093	8.066645	4.126766	4.66653	4.868912	2.83E-07	-0.70985
TAF9B	22.9314	23.22648	20.38127	13.50989	16.04524	13.79787	2.84E-07	-0.62124
MTLN	9.366121	10.17164	12.15302	3.018504	3.502387	4.684615	2.99E-07	-1.30552
MAP2K1	14.90139	13.25637	15.32635	9.700137	8.737886	9.990098	3.07E-07	-0.61056
SGCE	30.06618	32.2019	29.39883	21.01873	17.04353	21.62961	3.07E-07	-0.64147
PAQR3	5.383681	6.347871	5.170033	3.379609	3.844701	3.850048	3.2E-07	-0.60277
NBPF1	11.44851	11.1708	10.51385	6.781472	8.163083	6.531359	3.26E-07	-0.62845
KCTD9	14.67655	15.09944	14.1584	8.678709	10.62218	8.955538	3.33E-07	-0.63122
CD109	23.28436	20.82414	18.78643	12.83142	14.67053	11.06558	3.35E-07	-0.70052
GPR63	0.446926	0.399193	0.403512	0.055522	0.137104	0.092831	3.59E-07	-1.58381
CHAC2	11.33979	13.13126	13.83018	7.349502	7.861984	7.473604	3.9E-07	-0.74307
MTO1	4.186256	3.156824	4.07586	1.800636	1.383352	1.375936	3.92E-07	-0.6568
Homo_sapiens_newGene_8824	4.914978	5.083949	6.156433	2.251354	3.394024	3.056903	3.97E-07	-0.75783
TMEM209	11.03024	9.827477	9.955591	7.534494	7.113181	5.815192	3.97E-07	-0.61104
NXPE3	10.66264	11.03469	10.43356	7.496312	7.85387	6.09273	4.01E-07	-0.59498
Homo_sapiens_newGene_15905	15.70266	13.50813	14.72127	8.052309	9.477821	7.704727	4.32E-07	-0.89992
NFKB1	15.76284	14.79301	14.82543	10.35347	11.44336	9.813779	4.57E-07	-0.5901
STK4	11.98185	9.670863	9.402933	5.447261	7.516917	4.593569	5.15E-07	-0.80557
PRKCE	4.093276	3.008569	3.117667	3.624609	4.111583	3.044822	5.41E-07	-0.85482
RBM12B	8.453647	10.26311	9.480205	6.334516	6.002987	6.560266	5.67E-07	-0.63883
MEGF6	10.16289	8.425575	9.700062	6.250656	6.068682	7.270393	5.77E-07	-0.63046
NR2C2	11.46684	10.21922	11.01233	7.545329	7.164199	4.834906	5.98E-07	-0.76647

VPS54	9.585845	10.20425	9.393446	6.139178	6.730785	4.522032	6.44E-07	-0.73781
MMACHC	1.484899	1.238706	1.383442	0.756545	0.501015	0.584312	7.07E-07	-1.05779
LEPROT	7.085268	8.253179	7.514245	5.026296	5.187923	3.933614	7.41E-07	-0.71251
HSPA14	6.215364	6.871466	6.40956	3.439809	4.556717	3.68962	7.51E-07	-0.74715
TARBP1	5.50581	5.632321	5.089588	3.734124	3.687425	3.182854	8.56E-07	-0.61551
PKDCC	8.428137	9.748361	9.794673	5.936878	6.121266	5.980016	9.79E-07	-0.63158
TFEC	1.196926	0.866007	0.868371	0.35647	0.406819	0.294531	9.86E-07	-1.15884
FMR1	13.68557	14.0785	11.94142	9.176918	8.607093	8.575409	1.02E-06	-0.69241
SMIM10L1	5.741904	6.028619	5.971978	3.650151	3.641346	4.230752	1.04E-06	-0.62119
SNRNP200	51.00565	44.41552	44.23484	30.85512	34.4765	27.00001	1.05E-06	-0.6007
C21orf91	0.916681	0.902297	0.936668	0.30071	0.370188	0.631757	1.1E-06	-1.13968
RFX7	2.881485	2.907574	2.346861	1.253465	1.716533	1.019081	1.11E-06	-0.95641
ANKRD13C	9.122205	8.744542	7.704031	5.246443	5.882224	5.727045	1.12E-06	-0.59971
IFNLR1	4.249788	3.303122	3.340047	1.968265	1.946938	2.273115	1.12E-06	-0.75914
PARG	6.926411	5.707673	5.837011	4.185904	3.363598	2.560903	1.15E-06	-0.77713
IFI16	18.62326	15.72028	14.57762	12.16556	10.1831	9.923886	1.27E-06	-0.58566
NUAK1	5.362793	4.459359	4.179868	2.994116	3.213965	2.68655	1.42E-06	-0.69106
FBLIM1	42.81343	38.03413	27.94021	17.42113	21.68514	16.29387	1.45E-06	-0.93593
SCOC	15.52026	15.86084	17.22104	11.60534	10.01823	11.15952	1.49E-06	-0.58734
MOSPD2	2.660584	2.778441	2.895431	1.690031	0.991522	1.078099	1.54E-06	-0.88243
TMEM170B	0.140407	0.211358	0.246235	0.020406	0.048807	0.019823	1.6E-06	-1.65748
CDKN1A	37.47391	41.19334	40.24906	18.63858	10.97194	22.86386	1.63E-06	-1.09078
SIRT1	7.202048	6.248649	7.742095	4.440461	4.646708	4.542672	1.69E-06	-0.63182
MICA	12.61371	15.42779	13.7317	9.463308	8.596439	9.78775	1.7E-06	-0.63109
KBTBD8	1.721732	1.12859	1.298727	0.790954	0.854797	1.01416	1.73E-06	-1.45009
MBLAC2	1.040259	1.2048	0.870225	0.354124	0.580186	0.303585	1.82E-06	-1.28446

RNF2	10.14679	9.462961	9.778449	5.148902	7.323887	7.198584	1.98E-06	-0.61638
PDZD8	10.0613	8.666489	8.875453	6.567632	6.121122	4.950451	1.99E-06	-0.64456
ORMDL1	15.8281	20.98814	21.81534	12.43166	11.8035	11.43515	2.4E-06	-0.73202
CAPN7	14.1112	12.14193	13.85358	10.01432	9.042862	7.211285	2.41E-06	-0.64446
FAM214B	7.282105	3.916576	6.887179	2.398178	2.201944	2.736978	2.51E-06	-1.05777
STYK1	3.465897	5.185616	4.069313	1.388607	1.92615	1.76353	2.55E-06	-0.96005
MSH2	22.91405	24.17934	19.3278	14.93955	15.54891	13.66689	2.55E-06	-0.5918
Homo_sapiens_newGene_19628	8.062128	9.016675	9.016402	5.329724	5.711824	5.901639	2.56E-06	-0.60933
PRSS23	105.376	106.9665	53.63103	26.54605	24.52118	23.64111	2.57E-06	-1.58641
VPS37C	13.43029	11.18688	11.01361	8.183329	7.629319	7.752896	2.74E-06	-0.60522
HSPA4L	3.395056	3.002157	3.340195	2.318032	2.242106	1.93345	2.82E-06	-0.58886
C10orf88	2.8416	2.739298	2.711339	1.527241	1.555954	1.285901	2.84E-06	-0.87664
TMED5	26.57946	19.18457	25.51244	17.02798	16.43363	15.7829	2.93E-06	-0.67382
PARPBP	10.21827	10.17502	6.434793	3.836311	5.377029	3.812187	2.96E-06	-0.95861
ZFAND1	23.2906	18.15287	22.37987	14.63964	15.58595	13.56393	3E-06	-0.67056
GM2A	6.948194	7.22762	6.509035	4.214588	3.896831	4.823338	3.03E-06	-0.64754
ANXA3	12.39595	14.72752	11.66592	6.251152	7.346245	8.228148	3.03E-06	-0.79428
GLIPR1	25.50912	25.3842	21.05506	9.572038	13.37435	13.16532	3.18E-06	-0.74787
LGR4	21.07696	18.98124	19.15499	13.14914	6.755781	9.670446	3.27E-06	-0.93867
PCDHB6	2.617905	3.28878	2.821635	1.548066	1.673115	1.470203	3.29E-06	-0.84097
C12orf29	25.53444	33.57689	31.53741	20.94862	24.92577	25.16939	3.31E-06	-0.59064
ELF2	7.83706	6.690193	6.43191	2.801804	4.388967	3.816587	3.71E-06	-0.88976
SMAP2	10.87778	9.617873	12.19715	6.500119	7.101263	7.236336	3.99E-06	-0.64415
CLDND1	22.4621	21.7212	24.19016	13.82283	14.07143	12.38589	4.18E-06	-0.63852
SEC11A	40.53966	46.11952	41.5657	23.33262	33.16394	22.53218	4.28E-06	-0.69794
PTBP2	3.117279	3.656828	2.259649	0.754564	0.667669	0.613493	4.55E-06	-1.13461

CLDN2	11.6865	10.7809	13.17133	8.572001	7.231712	6.979204	4.8E-06	-0.65655
B3GALT5	5.457934	4.844935	4.762276	2.75071	3.671277	2.76868	5.03E-06	-0.69054
TRIM23	1.986217	2.007234	2.51064	1.067224	1.399767	0.935757	5.5E-06	-0.9425
RCOR3	13.01282	9.282194	10.60962	5.315103	6.082769	6.633789	5.68E-06	-0.73888
BMP2K	4.819869	4.558577	4.688273	2.549698	3.639615	2.484993	5.84E-06	-0.78961
HSD17B7	6.48611	6.265319	5.459189	3.36751	2.927751	3.419616	5.9E-06	-0.9074
HECTD1	10.89251	12.46659	11.51961	8.338077	9.620973	7.961753	5.95E-06	-0.63977
SAMD4A	4.336813	3.796455	3.615644	2.272518	2.684497	2.409075	6.2E-06	-0.6473
TMTC4	1.450458	1.397594	1.316477	0.704621	0.535795	0.594686	6.9E-06	-1.05916
JADE1	7.274846	6.242167	6.740386	3.977529	4.91485	3.558123	7.27E-06	-0.79461
Homo_sapiens_newGene_16208	3.518017	3.081655	2.772133	1.705005	2.428523	1.510896	7.34E-06	-0.77411
PARP12	8.65571	8.905799	8.310686	4.93299	5.728525	6.44303	7.9E-06	-0.62912
BTBD7	5.94494	6.008066	4.692883	3.939357	4.474501	4.108951	8.6E-06	-0.60791
SERPINB9	2.385211	2.466167	2.37946	1.451812	1.47007	1.506894	9.48E-06	-0.68935
MOB1B	4.018845	3.007016	2.873105	1.966816	2.116881	2.133583	9.63E-06	-0.66741
AP1S2	6.579173	7.67026	6.175859	4.191779	5.076483	4.251195	9.85E-06	-0.69333
JAZF1	3.482534	2.819633	3.292113	1.981764	1.422855	1.763464	1E-05	-0.85632
HMGA2	53.54275	46.82759	37.68269	20.63979	32.109	27.14536	1.02E-05	-0.61908
MMP1	8.022429	9.048779	8.591561	6.069894	5.192417	5.102455	1.02E-05	-0.64261
BRIP1	5.202629	4.61297	4.17186	2.428893	3.282541	2.033482	1.02E-05	-0.80716
FYCO1	4.098398	4.645455	4.381827	3.131026	2.76524	2.46802	1.06E-05	-0.70102
LRRC8B	2.730061	2.547804	2.677654	1.45421	1.944654	1.668722	1.06E-05	-0.71248
AMPD3	9.139956	10.56486	7.210524	5.869171	5.94961	4.410438	1.07E-05	-0.71076
KRIT1	10.61038	10.61264	8.001034	2.815825	3.972217	3.599584	1.09E-05	-1.24402
FAM174B	8.315361	7.421566	8.676318	5.095992	5.252469	6.004533	1.18E-05	-0.59223
GLMN	5.615041	5.663918	5.040708	2.07448	2.320844	3.428343	1.22E-05	-0.95536

RUFY3	10.33901	9.446926	9.946834	7.000274	6.083643	7.613704	1.24E-05	-0.59413
PLAUR	23.74264	23.04948	23.52314	12.33597	14.21225	18.60441	1.24E-05	-0.69395
BRWD3	2.755342	2.482335	2.315043	1.635621	1.713157	1.225569	1.36E-05	-0.70746
KDR	3.931452	3.75319	3.812778	1.683737	2.703847	1.846872	1.38E-05	-0.82157
ARL15	0.954526	0.989972	1.257923	0.787398	0.37344	0.38498	1.45E-05	-1.20919
WDR7	1.758074	1.301153	1.618922	0.401435	0.860446	0.635992	1.46E-05	-0.94788
GCOM1	1.059266	0.646807	0.98377	0.286569	0.259801	0.964204	1.49E-05	-1.32265
KLHL4	7.542326	7.721651	7.540153	3.933893	5.700908	4.49838	1.5E-05	-0.67752
STC2	17.90388	18.00385	17.20269	8.990715	13.07792	11.61922	1.5E-05	-0.65575
GID4	5.045911	6.223976	5.870878	3.797304	4.052565	3.861823	1.51E-05	-0.63576
ZIC2	2.779116	2.85501	2.652352	1.561013	1.745203	1.58196	1.68E-05	-0.73799
ZDHHC23	2.300509	1.562199	2.114913	1.007925	1.022589	1.101254	1.68E-05	-0.78812
DDX3X	115.4731	60.01699	101.5731	48.21988	49.62802	48.56242	1.68E-05	-0.88224
OLFM2	7.877043	7.94923	6.761544	3.68442	4.1616	4.791705	1.7E-05	-0.81285
GPR180	1.500471	1.345118	1.536858	1.000832	0.838812	0.737157	1.7E-05	-0.7406
CAMKK1	10.87685	9.71429	9.601073	5.393031	7.032992	6.892794	1.83E-05	-0.64159
ZBTB41	4.664561	4.074577	4.272697	2.842557	3.142236	2.256246	1.86E-05	-0.65085
SUSD6	2.602264	2.704648	2.994721	1.782134	1.87819	1.476972	1.94E-05	-0.6794
GAS2L3	4.031784	3.399017	4.16899	2.043322	2.530951	1.959726	2.07E-05	-0.83549
NIN	3.916449	3.833026	3.610151	2.29546	2.920322	2.289078	2.12E-05	-0.60761
SPATA5	1.621001	1.327852	1.26244	0.868949	0.827116	0.785313	2.12E-05	-0.73609
ZNF488	2.131472	2.421535	2.32102	1.418073	1.294756	1.291821	2.12E-05	-0.747
TPK1	2.512079	1.716285	2.136225	0.986953	1.791791	1.218047	2.24E-05	-0.91867
CSNK2A1	14.7067	15.78415	28.67501	12.54353	10.10087	7.523177	2.39E-05	-0.86532
RNF19A	9.510724	9.298426	8.414932	5.437108	6.564069	4.950738	2.47E-05	-0.60043
CABYR	14.81404	16.03434	13.55306	9.797982	9.478358	10.10184	2.73E-05	-0.64321

Homo_sapiens_newGene_2494	2.900005	2.110137	2.924113	1.097869	1.06177	0.722403	3.01E-05	-1.09875
AREG	28.53556	26.29008	26.95053	15.44237	17.84815	20.24649	3.11E-05	-0.60415
SECISBP2L	4.692706	4.720672	3.686655	2.91899	2.835557	2.354319	3.21E-05	-0.65022
C16orf70	7.489851	6.60453	6.529711	4.308767	4.130068	4.752057	3.24E-05	-0.60579
KRTAP2-3	8.632206	6.839279	7.563092	1.867151	3.690796	3.734411	3.41E-05	-1.11025
B4GAT1	5.718373	5.547373	5.76782	3.926909	3.295611	3.608076	3.45E-05	-0.64121
KCTD6	5.206977	4.22617	5.428839	2.516645	3.097547	2.75461	3.49E-05	-0.78956
ERCC4	3.624141	3.199724	3.332967	2.119269	2.523968	2.045465	3.6E-05	-0.5937
ASB4	3.663327	3.186885	2.734283	1.169434	1.817135	1.742018	3.64E-05	-0.79655
FGD6	7.043692	7.244057	5.813357	4.644799	4.630703	3.46129	3.68E-05	-0.63745
ARID3B	2.72771	2.212015	2.113979	1.370993	1.456475	1.26384	3.92E-05	-0.75182
TRIM35	7.647156	8.307081	8.040123	5.162715	6.127264	5.373466	4.01E-05	-0.6048
SLC25A20	7.832706	7.803111	8.130949	4.279636	5.430205	5.071965	4.03E-05	-0.69104
LIPG	2.382795	1.842811	2.111398	0.235501	1.103549	1.412701	4.15E-05	-1.12523
FIGN	1.475553	1.787454	1.454854	1.362905	0.987784	0.769194	4.29E-05	-0.74408
CDKN2D	3.394567	4.515885	3.865254	1.470004	0.83151	1.747239	4.42E-05	-1.22079
LGR5	1.295128	1.249352	1.740802	0.928446	0.702335	0.679071	4.57E-05	-0.83892
COL4A2	1.380778	1.551264	1.516291	0.997355	0.929731	1.003984	4.98E-05	-0.68096
CENPQ	6.69979	7.547703	6.547004	4.201548	4.807887	3.971128	5.1E-05	-0.6619
CLIC3	5.89292	5.550517	6.990193	2.652058	2.462161	3.704369	5.41E-05	-0.95157
Homo_sapiens_newGene_3857	2.610745	2.230065	1.537798	1.33241	1.101145	0.949401	5.46E-05	-0.91128
Homo_sapiens_newGene_16827	1.568212	2.00547	2.239282	0.386649	0.856684	0.903358	5.47E-05	-1.17179
PEX3	11.46666	15.17072	11.15147	6.826303	8.962378	7.988989	5.67E-05	-0.62674
DUSP9	3.685347	3.513433	3.563032	1.704494	2.061512	2.339001	6.09E-05	-0.7705
MAP3K7	10.14609	7.406905	10.70256	5.483682	5.978009	5.333867	6.29E-05	-0.69228
SEC14L1	42.35573	36.98973	38.36213	15.34432	26.44474	21.36891	6.73E-05	-0.86026

ABHD13	1.150842	1.383621	1.22431	0.797174	0.63463	0.580651	6.75E-05	-0.83891
SPRYD7	5.774659	5.637672	6.236777	2.908293	3.903572	3.971723	7.24E-05	-0.68597
FAM234B	2.442026	2.51682	2.045604	1.53134	1.422674	1.418603	7.47E-05	-0.66092
KIAA0586	3.244228	3.910432	4.301388	3.225243	2.293912	1.989661	7.5E-05	-0.6484
Homo_sapiens_newGene_9227	14.73351	16.40599	18.99164	10.09584	9.240817	9.151646	7.7E-05	-0.77152
HOOK1	0.472009	0.408479	0.512041	0.216626	0.173443	0.116361	8.01E-05	-1.16731
Homo_sapiens_newGene_15489	0.769745	0.786453	1.215192	0.156261	0.140074	0.235932	8.06E-05	-1.41059
CCNE2	9.47261	8.884505	7.388038	4.097821	6.161351	5.539341	8.78E-05	-0.62489
ME2	17.08845	10.374	18.75435	4.010609	5.96088	4.166261	8.93E-05	-0.73028
Homo_sapiens_newGene_8846	1.842427	1.291785	1.684608	0.924407	0.843488	0.929217	9.19E-05	-0.78761
HIPK2	28.12949	26.68002	24.40971	17.98368	20.04389	12.97896	9.43E-05	-0.62327
TMEM168	5.158591	7.082632	7.864803	3.718307	3.317253	2.891897	9.76E-05	-0.81092
BNIP2	4.596211	4.611108	10.21917	3.79807	2.861847	3.341101	0.000106	-0.68511
XK	0.419635	0.380683	0.300515	0.141588	0.112646	0.115866	0.000106	-1.21269
MTIF3	7.986232	8.968259	9.142763	5.650946	5.280592	5.45449	0.000113	-0.65661
MINDY2	1.176176	1.185715	1.233188	0.623322	0.695934	0.332369	0.000113	-1.08303
TMEM182	3.984185	5.266492	3.577687	2.821687	2.140055	2.245176	0.000118	-0.65638
IRAK4	7.07284	4.756589	7.701523	6.773022	5.044531	5.156716	0.000122	-0.65032
Homo_sapiens_newGene_12702	2.490626	2.422151	2.562981	1.659913	1.630069	1.497931	0.000123	-0.63186
Homo_sapiens_newGene_12578	1.082623	1.155349	1.000053	0.508154	1.122951	0.361008	0.000128	-1.09794
CMTM4	9.863272	7.382687	7.619529	5.889945	5.295698	5.459092	0.000131	-0.60323
PIKFYVE	3.915636	3.747665	3.047715	2.413212	2.47364	2.093407	0.000132	-0.79823
CFL2	34.86176	39.03884	18.2021	9.572545	12.192	24.12473	0.000133	-0.89368
Homo_sapiens_newGene_8453	1.740656	1.876195	2.115223	0.735472	0.961425	0.650807	0.000151	-0.98095
Homo_sapiens_newGene_17275	3.015354	3.251053	3.966307	1.854574	2.006478	2.529689	0.00016	-0.7904
SACS	3.783437	2.986347	2.72967	1.787416	2.32779	1.576892	0.000168	-0.6977

MEF2C	2.650801	2.278139	2.29191	1.185607	1.582042	0.705333	0.000173	-0.93956
USF3	2.225349	2.243524	1.824861	1.649411	1.281118	0.801102	0.000186	-0.75757
KLHDC2	11.2983	11.21371	10.98415	2.335485	8.489676	7.031697	0.00019	-0.62752
LAMC2	11.74904	10.04461	10.74729	5.310519	7.858552	7.406005	0.000194	-0.64407
CYR61	13.12535	12.05594	11.90386	5.241487	8.778604	7.675845	0.000203	-0.73233
SYTL5	1.606234	2.002085	1.570138	1.124733	0.667916	0.864291	0.000203	-0.87384
Homo_sapiens_newGene_9169	1.44163	1.4218	1.066474	0.764055	0.649401	0.816273	0.000223	-0.81182
MPZL3	0.955378	1.067411	1.029527	0.455692	0.40456	0.224234	0.000225	-1.05362
SMPD2	9.131996	10.01033	10.41022	6.980497	5.234353	6.21538	0.00023	-0.63453
COPS7A	46.64922	51.94595	27.08964	23.36959	22.80231	25.80391	0.000243	-0.78066
FGF5	0.243745	0.21879	0.222803	0.049676	0.056889	0.021113	0.000245	-1.33526
WDR41	4.789701	6.105491	6.213394	3.981853	3.561652	4.312072	0.000288	-0.68236
FAM200B	3.79686	4.048278	3.948369	2.374556	2.45505	2.476976	0.000303	-0.64799
MANSC1	4.953033	3.999381	3.39915	3.427642	2.183911	2.231094	0.000303	-0.79424
LMCD1	34.75598	37.50883	37.67125	8.937755	31.76779	31.72223	0.000309	-0.64544
GPR160	1.547512	1.834101	1.631194	0.825895	0.713501	0.894741	0.000313	-0.9146
Homo_sapiens_newGene_100	5.025861	4.527966	4.431538	2.626101	3.906426	2.736674	0.00033	-0.74289
IL7R	5.037349	4.020629	3.54413	2.563111	2.87876	3.006989	0.000331	-0.67372
SPDEF	5.002212	4.943944	5.467262	3.645264	2.926568	3.291391	0.000336	-0.62606
SKIL	11.36568	8.516377	9.554148	4.899399	5.357537	7.316911	0.000337	-0.73323
LYSMD2	4.563041	4.072227	3.280002	2.323344	1.786528	2.361111	0.000342	-0.80652
PRKAG1	25.19664	27.21557	26.31018	15.19201	22.04372	14.98691	0.000361	-0.60281
GPR137C	0.707722	0.618554	0.624312	0.368619	0.185802	0.193885	0.000388	-1.08859
MDM1	3.290866	3.544907	3.114458	2.707729	2.181855	2.091358	0.000412	-0.62945
Homo_sapiens_newGene_18542	3.710249	3.364962	4.609682	1.471527	1.461339	2.427213	0.000416	-0.97568
CDV3	43.52198	78.35367	76.90977	24.10394	31.0022	33.84092	0.000421	-1.12165

Homo_sapiens_newGene_16980	2.598484	2.776858	2.973638	1.554231	1.619494	1.952613	0.000436	-0.67071
P2RY1	0.213603	0.183642	0.201162	0.067442	0.035305	0.064805	0.000444	-1.2145
CACNA1I	0.20612	0.289941	0.261587	0.081189	0.09895	0.020655	0.000454	-1.24659
RB1	1.966343	1.661987	1.89192	0.925398	1.328807	0.978403	0.000463	-0.72994
Homo_sapiens_newGene_11399	2.243752	2.802354	3.099891	0.979103	1.659215	1.174139	0.000493	-0.88138
CLCN3	12.53655	10.97957	12.30236	8.843424	9.139612	7.983661	0.000494	-0.61567
DIPK1A	8.893772	9.715146	8.012645	7.084871	5.731372	5.043564	0.000499	-0.58719
CMPK1	47.92205	41.03801	24.04635	8.042734	15.6242	8.205536	0.000516	-1.23055
COPS8	7.545383	12.54409	15.44163	6.852759	5.207225	6.706058	0.000522	-0.70378
TAF5	2.13839	2.163494	2.129882	1.277285	1.489441	1.269064	0.000533	-0.64631
Homo_sapiens_newGene_19903	2.902936	2.326894	2.499018	1.166193	0.939351	0.612434	0.000543	-1.13583
MB21D2	4.979819	4.960535	5.471019	2.574702	3.705842	3.4951	0.000554	-0.63299
ZFYVE16	3.395071	3.172915	3.330092	1.855583	2.612627	1.474638	0.000564	-0.72672
Homo_sapiens_newGene_13226	1.580193	0.839354	0.927878	0.574154	0.472801	0.401729	0.000573	-0.84948
NKX2-5	2.940364	3.006924	3.465864	1.671094	1.84958	1.882643	0.000582	-0.72354
ASB9	4.698831	5.98726	6.476386	3.949233	3.37617	3.117915	0.000616	-0.6559
TP53INP1	1.567057	1.436151	1.381568	0.902642	1.005605	0.649555	0.00064	-0.7295
Homo_sapiens_newGene_17135	2.757204	2.104944	2.138346	0.540363	1.445407	0.940526	0.000667	-1.04969
ZBTB37	0.696387	0.569985	0.682694	0.410689	0.271274	0.208923	0.000667	-0.84004
RAB28	7.413746	7.482098	6.667983	5.085837	5.793876	3.374165	0.000695	-0.65035
Homo_sapiens_newGene_14051	2.818861	1.88915	2.313554	1.640412	1.948629	1.263243	0.000705	-0.95139
CEP19	2.591156	2.052161	1.934452	1.085122	1.10534	1.13184	0.000708	-0.80494
SLC45A3	1.727894	2.046023	1.686153	0.985042	1.221438	0.969011	0.00071	-0.72779
CEP135	2.657929	2.273382	2.389698	1.700226	1.840266	1.108021	0.000719	-0.68301
CCDC68	0.922502	1.301971	0.863524	0.220685	0.503366	0.472035	0.000796	-1.03923
COX11	42.37619	29.61813	36.03726	34.95136	29.75645	37.11735	0.000796	-0.69817

43899	1.182498	1.23217	1.089839	0.590098	0.651418	0.587007	0.000808	-0.91541
CHIC1	9.076178	7.532756	7.867629	5.58692	3.602416	4.487496	0.000814	-0.8187
SLC10A7	2.028228	2.594711	2.684783	1.926923	1.068753	0.794431	0.000816	-0.80107
HMGCR	16.5069	14.09296	14.77233	9.021549	12.55303	8.919785	0.000821	-0.5992
PFKFB4	2.384261	1.845467	1.351516	1.029897	0.361395	0.696658	0.000917	-1.0378
MFSD2A	3.817554	2.929864	3.360341	1.68604	2.13736	2.356205	0.000952	-0.708
C16orf87	5.763619	5.380885	6.387978	3.710232	4.266215	4.332073	0.000985	-0.61751
FMN2	0.911106	0.815386	0.75463	0.54991	0.304049	0.443704	0.000986	-0.84205
BEND6	2.324583	2.39842	2.061242	1.78402	1.734893	1.295409	0.001013	-0.69409
PARP9	4.547189	4.666651	4.807304	3.17813	3.146272	2.258354	0.001135	-0.66544
YIPF5	10.32119	10.78678	10.74981	7.099135	6.712959	3.594267	0.001187	-0.77776
ARL6	1.385421	1.371893	1.139938	0.840975	0.625606	0.924565	0.001206	-0.73101
TLDC2	2.951453	3.018793	3.348482	1.303669	1.755096	2.129372	0.001334	-0.77734
Homo_sapiens_newGene_18157	1.943382	1.432361	1.629166	0.88483	1.140884	0.254102	0.001349	-1.14196
HSPB6	1.213359	1.239312	0.609235	0.151158	0.414451	0.208195	0.001361	-1.18719
Homo_sapiens_newGene_16414	1.686787	1.541676	1.843067	0.959412	1.14256	0.806888	0.001367	-0.79989
ZBTB20	6.947205	5.080212	4.261765	4.063944	4.121516	2.776556	0.001377	-0.64681
FAM241B	3.330268	4.464322	5.020137	2.399425	1.697807	2.398626	0.001543	-0.84506
DIXDC1	1.63589	1.365879	1.255851	0.933585	0.757004	0.708385	0.001577	-0.63878
FUT11	2.711235	3.241327	2.647483	1.647815	1.948699	1.623464	0.001624	-0.6763
ASB3	4.958206	5.978518	4.001578	3.178694	2.862237	3.46213	0.001632	-0.6471
CACNA2D4	0.667358	0.824113	0.843103	0.409167	0.425396	0.21056	0.001744	-0.8835
POMK	5.378973	2.879235	2.754001	1.197846	2.036263	0.731901	0.001762	-1.06081
F8A3	1.644137	4.331861	2.716994	1.492515	0.276116	0.481419	0.001863	-1.15918
TLR6	3.584819	4.208206	2.922797	2.446754	2.012676	1.393408	0.001873	-0.99276
DDHD2	4.378878	4.145189	4.069481	2.811997	2.739038	2.878735	0.001925	-0.61843

MECP2	20.40112	16.93138	10.31343	12.5604	28.66882	17.37929	0.002083	-0.62645
ERAP2	4.461188	3.684026	4.752633	3.172047	3.627136	3.610182	0.002169	-0.64178
DFFB	1.0951	1.036666	1.337874	0.651726	0.430252	0.568617	0.002238	-0.85016
FER1L6	0.393484	0.445076	0.192419	0.139833	0.068398	0.018049	0.002244	-1.16073
SATB2	2.134646	2.314922	2.406919	1.508137	1.349354	1.060909	0.002267	-0.61771
TNFAIP8	6.871059	3.62023	6.202576	3.231039	3.691074	2.31925	0.002553	-0.7425
TSPYL4	1.709124	1.600488	1.801034	1.065735	0.985772	1.242843	0.002636	-0.60215
EFEMP1	3.275656	2.720255	3.343313	1.498781	1.86399	2.232671	0.002873	-0.63689
ZNF711	1.229855	1.526063	0.997116	0.609294	0.464909	0.576671	0.003055	-0.80058
RHEBL1	0.740553	1.253153	0.754189	0.179674	0.340364	0.258733	0.003142	-1.09408
TNS4	1.540211	1.36955	1.407835	0.707241	0.909327	0.710167	0.003197	-0.67007
DENND2C	0.453597	0.400786	0.459166	0.12306	0.243741	0.229054	0.003234	-0.91513
Homo_sapiens_newGene_6435	1.187504	0.823004	0.944247	0.466384	0.637231	0.527613	0.003237	-0.81275
SLC14A1	13.01935	16.80068	14.20577	7.542338	12.30925	9.654557	0.003389	-0.66442
CHN1	1.79412	1.255068	1.632086	0.428952	0.923627	1.048422	0.003397	-0.88714
GRPR	1.687111	1.335784	1.736475	0.813724	0.974198	0.633228	0.003524	-0.82522
Homo_sapiens_newGene_16783	3.324822	2.706417	2.184516	0.68278	3.027366	0.862318	0.00354	-0.96731
NUBPL	1.660597	1.905453	1.749792	1.252279	0.819859	0.889898	0.003612	-0.70491
TMOD1	2.818585	1.459563	2.286288	1.311257	1.018505	1.208572	0.003755	-0.7794
UBE2A	29.02663	71.74635	71.21591	28.05149	29.36925	30.69785	0.003765	-0.74548
PLA2R1	0.407507	0.435586	0.34592	0.261356	0.235616	0.244798	0.003776	-0.62716
EDN2	1.333656	1.397974	0.961695	0.389583	0.284718	0.61461	0.003883	-1.02844
Homo_sapiens_newGene_11003	1.91423	2.780488	2.202742	0.974824	1.430323	1.422407	0.003922	-0.74939
LYRM7	1.7697	1.327915	1.214765	0.728147	0.829393	0.783485	0.00393	-0.78998
RNF144A	1.465359	1.128214	0.999325	0.711674	0.794531	0.785569	0.004034	-0.64977
PLPP6	1.018892	0.722138	0.783054	0.366829	0.504012	0.316805	0.004177	-0.88572

MAP3K8	4.247634	4.292259	3.943786	2.931032	2.675319	2.229954	0.004195	-0.6243
PPP6C	18.1841	19.93534	19.98051	8.113862	13.83723	14.38045	0.00438	-0.6319
DDHD1	1.280379	0.468259	0.968002	0.69942	0.344076	0.336249	0.004593	-0.63121
TSC22D3	4.797785	5.216566	6.553484	3.136087	3.300617	4.127657	0.004689	-0.61403
TTBK2	1.212823	0.776908	0.89249	0.309504	0.46478	0.695803	0.004833	-0.65104
NREP	1.275785	1.636268	1.234138	0.997482	0.716015	0.690854	0.005395	-0.78146
ENO2	10.11191	8.447873	7.223637	3.952365	6.309129	5.5904	0.006096	-0.65079
ZNF566	1.983824	2.747336	2.384424	2.200772	1.56676	1.509011	0.006154	-0.60976
ZNF460	7.222646	5.51512	2.858675	2.548243	3.397724	1.766231	0.006694	-0.79759
DGKH	2.655076	2.251328	1.776798	1.013777	1.204733	1.164391	0.006756	-0.80673
IL1B	2.807383	2.875452	3.342788	2.249478	1.902454	1.594713	0.006825	-0.65112
IL1A	1.744222	1.131654	1.423863	0.944682	0.58479	0.611062	0.006864	-0.82952
Homo_sapiens_newGene_9381	0.846686	0.957527	0.634182	0.499923	0.459259	0.344579	0.006883	-0.77042
MUC5AC	5.428519	4.14587	3.554224	2.640128	3.5578	1.774304	0.006904	-0.66068
TET2	1.574372	1.413961	1.318494	0.800504	1.266363	0.639523	0.006918	-0.64789
KIAA1324	0.595512	0.555009	0.74921	0.299403	0.356262	0.228964	0.006942	-0.85062
PGAP1	0.633873	0.731709	0.548574	0.390481	0.563801	0.242551	0.007413	-0.65626
GYG2	2.230565	2.034378	2.082052	1.60714	0.993089	0.850894	0.007979	-0.75413
NCR3LG1	2.943362	3.16244	2.291885	1.765037	2.235364	1.227447	0.007995	-0.63235
CYP4V2	3.626261	2.926301	3.21031	2.061473	2.436426	1.427779	0.008508	-0.65562
HNRNPUL2-BSCL2	0.734284	1.954682	1.33757	0.709124	0.539421	0.66528	0.008636	-0.84929
SMIM12	12.3479	13.21262	6.555788	5.95523	6.623606	8.304491	0.008832	-0.77113
Homo_sapiens_newGene_18015	2.412153	2.981985	2.744823	1.850464	1.315056	1.050363	0.00896	-0.7704
Homo_sapiens_newGene_3473	1.769805	1.728875	1.434468	0.463312	0.897298	0.871072	0.009053	-0.7624
ZBED6	2.872209	6.890346	3.334087	1.363568	2.252321	2.947912	0.009146	-0.7048
RASEF	0.98291	1.428639	1.071277	0.706203	0.901917	0.65385	0.00988	-0.6084

SMIM8	1.762505	1.978571	2.059238	1.499622	1.21533	1.088222	0.010509	-0.62313
M6PR	112.4689	74.20011	70.68014	27.16177	42.00726	40.54833	0.010869	-0.91069
BBIP1	5.407204	7.08917	6.854933	5.619515	4.253052	4.423478	0.011585	-0.62138
PCDHB2	1.070381	2.317392	1.995474	0.970748	1.098357	0.784126	0.012749	-0.81109
HECTD2	1.562695	1.896903	1.106978	1.096846	1.4231	0.761871	0.013414	-0.69446
MAP3K14	3.932573	3.523592	4.903115	1.515466	3.402665	3.252134	0.014045	-0.64355
Homo_sapiens_newGene_9941	0.921718	2.215119	1.62058	1.002614	0.483116	0.507041	0.014188	-0.78652
DCUN1D4	5.266047	5.963024	9.093846	3.51441	5.577426	3.878752	0.014665	-0.71814
ABAT	0.228322	0.345407	0.288923	0.110843	0.087918	0.131753	0.014665	-0.89257
Homo_sapiens_newGene_2442	2.110237	2.762366	0.972547	1.675915	1.556522	0.641263	0.014848	-0.77611
HRH1	7.784245	8.234079	10.63142	5.170688	5.990987	7.142766	0.01507	-0.58571
SYDE2	0.46687	0.52571	0.459329	0.309704	0.300218	0.210399	0.015517	-0.70728
SOST	0.630854	0.382971	0.433922	0.09892	0.258917	0.115611	0.016182	-0.93595
Homo_sapiens_newGene_2565	0.789087	0.92248	0.830477	0.372233	0.286051	0.350589	0.016362	-0.8961
RASSF5	0.360862	0.376555	0.216683	0	0.053304	0.069174	0.016431	-0.94195
RRAD	0.713031	0.599678	0.691778	0.182444	0.341339	0.269868	0.016443	-0.89882
LYNX1	0.654522	0.679714	0.739687	0.272496	0.315758	0.50724	0.016934	-0.7438
KIF17	0.629933	0.765524	0.669526	0.306566	0.458644	0.32317	0.017499	-0.7283
SHANK2	0.157632	0.204793	0.09115	0.046093	0.061733	0.166158	0.017888	-0.87727
GPR75	1.908388	1.739144	1.630834	1.351283	0.970303	0.685149	0.018746	-0.69704
WSCD1	1.26576	0.622022	0.847066	0.524002	0.827925	0.578168	0.018768	-0.70315
ANGPTL4	3.052991	2.461826	2.65051	2.191522	1.712359	1.759507	0.018986	-0.59642
Homo_sapiens_newGene_14605	0.869991	0.774893	0.713277	0.472717	0.400777	0.48765	0.019115	-0.67546
ZNF461	2.379655	2.625006	3.774701	1.681068	2.008917	1.682485	0.019388	-0.64176
PITPNB	32.90992	15.88405	14.86014	12.90569	13.70922	12.87924	0.019873	-0.63896
HNMT	2.638179	2.75444	2.812864	2.915529	2.138586	2.177175	0.019897	-0.65035

KIF21B	0.325556	0.268607	0.295011	0.183007	0.209777	0.135122	0.019899	-0.66588
STOX1	0.613619	0.65969	0.477787	0.398221	0.175695	0.187602	0.019961	-0.91499
TBC1D2	1.946001	1.281174	1.810924	1.168239	1.154764	0.846412	0.020377	-0.59363
GCNT4	0.386455	0.318465	0.241211	0.080359	0.192928	0.143289	0.020381	-0.84471
DCTN4	13.30918	6.708763	6.583013	5.243724	5.70718	5.400737	0.020405	-0.61502
ULBP3	4.530145	5.558226	3.476562	1.928683	3.005068	2.748092	0.021057	-0.69143
43894	0.894087	0.583579	0.751496	0.33517	0.506814	0.429774	0.021218	-0.68629
ATP11A	5.339116	5.35254	4.277499	3.630235	3.972212	2.708522	0.021699	-0.60956
C6orf223	0.412533	0.428406	0.401765	0.171172	0.165642	0.265263	0.022378	-0.79502
ELK3	8.81044	8.203709	6.576641	3.366344	6.030389	4.655154	0.023342	-0.62565
HOXB4	0.287639	0.341692	0.367469	0.08286	0.164107	0.175745	0.023588	-0.84724
SERPINB2	0.999767	0.647586	0.553644	0.052788	0.378952	0.217936	0.024046	-0.89983
PRDM16	0.219762	0.158797	0.190799	0.057798	0.088188	0.095353	0.024836	-0.78897
KLHL3	0.91225	0.733554	0.449753	0.414426	0.322249	0.444291	0.026264	-0.68041
TRIM16	65.47371	101.0562	69.66636	29.66058	44.99923	55.93444	0.028376	-0.80999
Homo_sapiens_newGene_4091	3.723103	3.530701	2.767599	1.864516	2.842361	2.271306	0.028836	-0.59181
CASP2	12.35615	11.30945	10.14302	6.077652	5.675148	2.431811	0.029245	-0.84768
TTLL7	0.626726	0.611691	0.448822	0.352627	0.421072	0.287586	0.029391	-0.60461
Homo_sapiens_newGene_4037	1.259538	1.256672	0.915253	0.493738	0.648507	0.826301	0.031301	-0.68608
SLC16A2	0.744281	0.786228	0.622309	0.365668	0.419714	0.232291	0.03273	-0.70441
CLDN12	5.693017	9.111269	4.640477	3.84175	4.32201	4.182759	0.032983	-0.60474
SRSF12	0.554057	0.419128	0.492656	0.309509	0.161193	0.137455	0.035683	-0.76059
ELF4	19.09968	17.10438	37.5084	15.30848	11.249	8.603636	0.035738	-0.77467
IGFBP1	5.227748	4.880016	9.899646	1.743768	4.576859	4.132454	0.035962	-0.72465
PDP2	2.947326	3.161161	3.67897	1.378737	1.118173	1.190587	0.037723	-0.83025
HYAL1	0.647562	1.440799	0.799953	0.72647	0.55379	0.454863	0.040701	-0.71927

DRAM1	2.583682	3.691201	2.840002	2.030944	2.77806	1.632382	0.044347	-0.66013
MTERF1	3.542802	7.794738	5.99038	2.964223	4.252183	4.056907	0.044493	-0.63431
Homo_sapiens_newGene_19415	1.872375	1.939341	1.076982	0.952992	0.819049	0.811055	0.044626	-0.61762
OTUB2	0.87187	0.592072	0.642193	0.52436	0.429121	0.311721	0.044937	-0.65597
ASAH2	0.35285	1.679346	0.489389	0.242084	0.211775	0.864112	0.045527	-0.61923
Homo_sapiens_newGene_1788	0.451422	0.3131	0.393857	0.218804	0.124978	0.201038	0.047937	-0.75819
APOLD1	3.775425	0.292918	11.72284	0.258494	0.822367	0.304073	0.048398	-0.70976

Here, we present the STR certificates for Huh7 (page 2-4), HCCLM3 (page 5-7) and MHCC97H (page 8-17) cell lines, respectively.

CH INA CENTER FOR TYI°E CULTURE COLLECTION (CCTCC)
Wu han University, Wu han 430072, China
Ph o we: 86-027-68752093 Fax: 86-027-G8754833 Email: slaenchao@whu.edu cn

05-21-2019

Entrusted by Zhejiang University, CCTCC has conducted identification experiments on the Huh-7 cell line, and come to the following conclusions:

1. There was no third allele found in Huh-7 cell line, it indicating that there was no cross-contaminant of human source cell line.
2. Compared the STR data of Huh-7 cell line in the databases of ATCC and DSMZ, all the locl of Huh-7 were exactly matched with the loci of HuH-7 (JCRB0403) cells found in DSMZ cell bank, so it is HuH-7 (JCRB0403) cell line (Table 1).

Manager:

China Center for Type Cu[illegible]ion

Note:

1. The result is only responsible for the test salrple, and the genomic DNA will be reserved for three month.
2. Reference of human cell line authentication: ANS I/ATCC ASN-0002-20 11

Table1. The alleles of 21 locations in Huh-7 cell line

Huh-7 cell line（Fig. No. XB19-744）		
Marker	Allele 1	Allele 2
D19S433	13	14
**D5S818**	**12**	**12**
D21S11	30	30
D18S51	15	15
D6S1043	13	15
**AMEL**	**X**	**X**
D3S1358	15	15
**D13S317**	**10**	**11**
**D7S820**	**11**	**11**
**D16S539**	**10**	**10**
**CSF1PO**	**11**	**11**
Penta D	12	12
D2S441	12	14
**vWA**	**16**	**18**
D8S1179	14	14
**TPOX**	**8**	**11**
Penta E	11	11
**TH01**	**7**	**7**
D12S391	20	21
D2S1338	19	19
FGA	22	23

CTCC

Sample Hame Panel S Q O S OS SQ SSPK MIX OMR .CGQ

X B19-744 MR21_v2.0

HsrASepleforDeleton

60 100 140 180 220 260 300 340 300 d20 d8O

1200

1000

800

600

400

200

0

30 l5

30 IJ

MR21_v2.0

barb S ampl e for 0 e1 et i on

60 100 140 180 220 260 300 340 300 d20 d8O

5000

4000

3000

2000

1000

0 IJ

II 10

II 10 11

X B19-T44 MR21_v2.0 100 180

140

#erd Semple Cor Deletion

60 220 260 300 340 380 d20 d80

1600

1200

800

OOO

0

X B19-744 MR21_v2.0

#srd Sample for Deletion

0J

60 3 200 100 140 180 220 260 300 3dO 380

0 0

3000 0

dOO 0

O 0

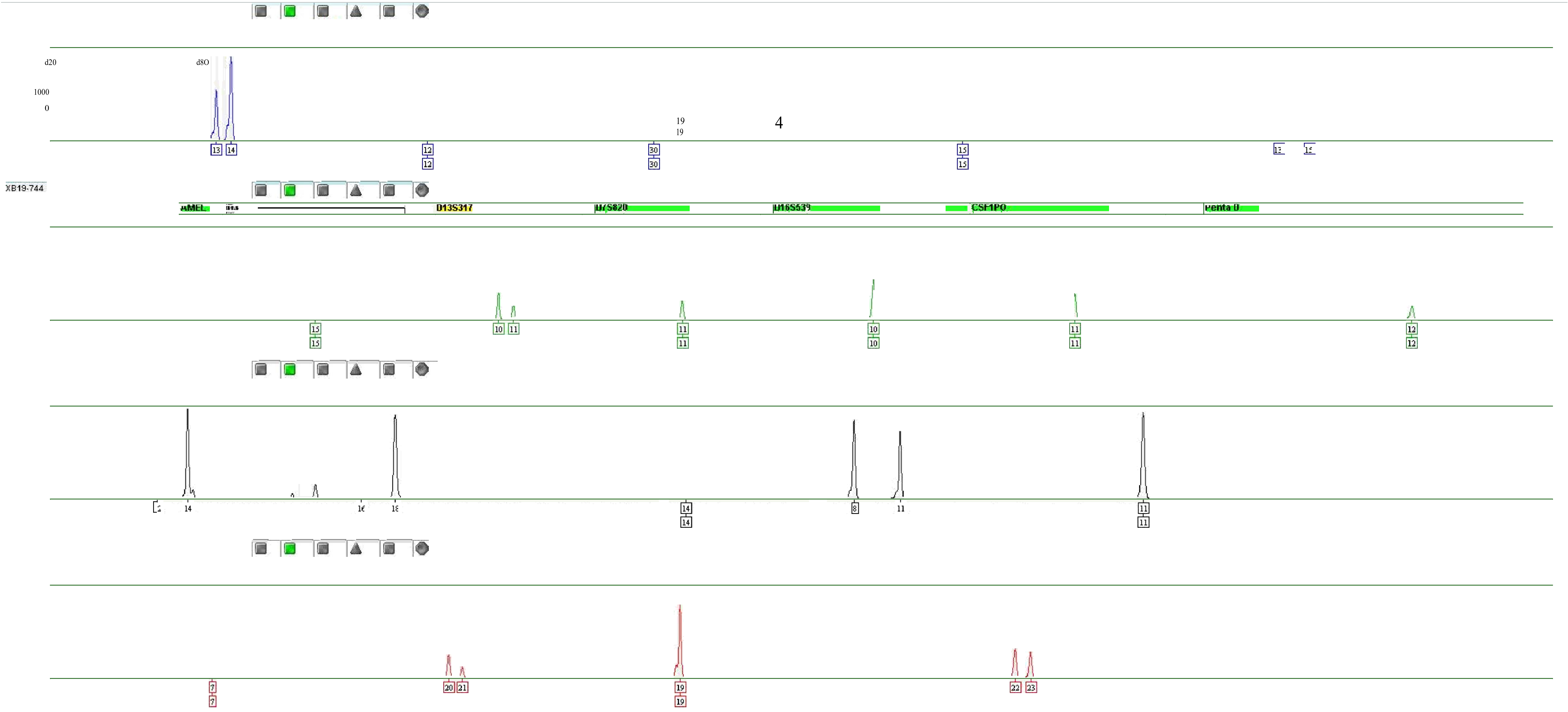
XB19-744
D13S317
CSF1PO
13 14
12 12
30 30
15 15
15 15
10 11
11 11
10 10
11 11
12 12
14
16
18
14 14
8 11
11 11
7 7
20 21
19 19
22 23

‹P @ iâi iLi 1“» N 4S lik ifi 't• 'é'
CH INA CENTEІІ FOR TY PU CULT U UE COLL ECT I ON (CCTCC)
¥V' u li a n U nivc rs ity, Yt' It li 9 In 4301172, C li ink
I•lione: SP-027-fa8752(J93 Fax: 86-027-68754833 Email: shenchao@whu.edu.cn

05-23-2019

Entrusted by Zhejiang UniVelsity, CCTCC has conducted identification experiments on the HCC-LM3 cell line, and come to the following conclusions:

1. There was no thii d allele found in HCC-LM3 cell line, it indicating that there was no cross-contaminant of human source cell line.
2. Compared the STR data of HCC-LM3 cell line in the databases of ATCC and DSMZ, its profile does not exactly match with any of the crlFl ent data (Table 1).
3. 1 he STR data of BICC-LM3 cell line and NCI-hI82 (HTB- 175) cell line hatches the highest rate of 73% in ATCC database.

Manager :

China Center fo pe u 11 CC I1 OU

Note:

1. T fee result is on ly responsib e or die test sample, and the genomic DNA will be reserved for three naontla.
2. Reference of human cell line authentication: ANS1/ATCC A SN-0002-2011

Table1. The alleles of 21 locations in HCC-LM3 cell line

HCC-LM3 cell line （Fig. No. XB XB19-750）		
Marker	Allele 1	Allele 2
D19S433	13	14
**D5S818**	**12**	**13**
D21S11	31.2	31.2
D18S51	13	22
D6S1043	12	20
**AMEL**	**X**	**Y**
D3S1358	15	16
**D13S317**	**8**	**8**
**D7S820**	**10**	**10**
**D16S539**	**12**	**12**
**CSF1PO**	**11**	**13**
Penta D	8	9
D2S441	15	15
**vWA**	**14**	**14**
D8S1179	12	13
**TPOX**	**8**	**8**
Penta E	17	17
**TH01**	**9**	**9**
D12S391	18	18
D2S1338	20	20
FGA	21	24

CCTCC

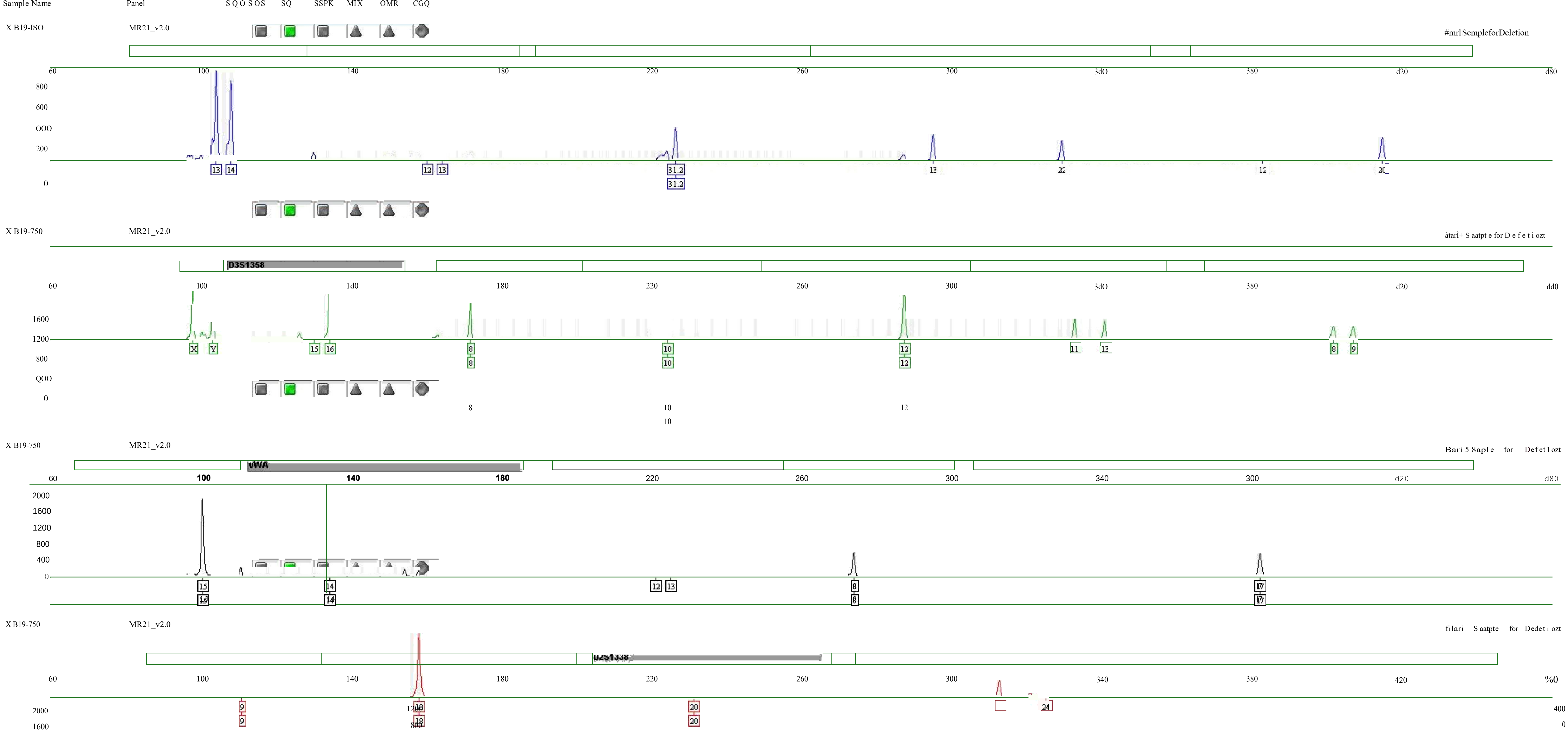
Sample Name
Panel
S Q O S O S
SQ
SSPK
MIX
OMR
CGQ
X B19-ISO
MR21_v2.0
#mrl SempleforDeletion
X B19-750
MR21_v2.0
àtarİ+ S aatpt e for D e f e t i ozt
D3S1358
X B19-750
MR21_v2.0
Bari 5 8apI e for Defet l ozt
vWA
X B19-750
MR21_v2.0
filari S aatpte for Dedet i ozt

9

18
9

18

7

21

上海传秋生物科技有限公司

细胞遗传质量鉴定检测

Cell Line Authentication Service

STR 基因型检测报告

样品信息

样品编号：

客户样本编号	公司编号
MHCC-97H	20190409-01

样品数量： 1

样品性状： 细胞系

检测项目： STR

www.chuanqiubio.com

检测方法： 用 Axygen 的基因组抽提试剂盒提取 DNA,采用 20- STR 扩增方案扩增,在 ABI 3730XL 型遗传分析仪上对 STR 位点和性别基因 Amelogenin 进行检测。

样品信息

（一）检验基本情况

公司编号	多等位基因	匹配细胞系	细胞库	EV 值	匹配说明
20190409-01	无	-	DSMZ	-	无匹配

样本基因型检验结果

- 多等位基因指三等位及以上基因现象。
- 本次检测各细胞分型结果良好。

（二）各样本描述

- 20190409-01：该株细胞 DNA 分型在细胞系检索中**没有找到匹配**的细胞系，本次检测在该细胞系中没有**发现多等位基因**。(该细胞系未发现多等位基因、未发现交叉污染，细胞系无异常，因数据库未收录 MHCC-97H 相关 STR 信息无法匹配，若为发表论文用，提交该数据给杂志即可)

备注：待测细胞系与收录于 ATCC，DSMZ，JCRB 和 RIKEN 数据库的细胞系 STR 数据进行比对，未收录于以上细胞库的细胞系将无法匹配。

（三）样本分析结果

**细胞 20190409-01 的 STR 位点和 Amelogenin 位点的基因分型结果**						
Loci	送检细胞 STR 信息			细胞库细胞 STR 信息		
	送检细胞名：MHCC-97H			细胞库细胞名：		
	Allele1	Allele2	Allele3	Allele1	Allele2	Allele3
D5S818	12	13				
D13S317	8	8				
D7S820	10	10				
D16S539	12	12				

VWA	14	14				
TH01	9	9				
AMEL	X	Y				
TPOX	8	8				
CSF1PO	11	13				
D12S391	18	18				
FGA	21	24				
D2S1338	20	20				
D21S11	31.2	31.2				
D18S51	13	22				
D8S1179	12	13				
D3S1358	15	16				
D6S1043	12	20				
PENTAE	11	17				
D19S433	13	14				
PENTAD	8	9				

其他说明

（一）分型方案及位点信息

	方案 1	方案 2	方案 3	方案 4
1	TH01	TPOX	D3S1358	AMEL
2	D12S391	VWA	D13S317	D5S818

3	D7S820	D8S1179	D6S1043	D2S1338
4	CSF1PO	PENTAD	D16S539	D21S11
5	FGA		D19S433	D18S51
6	PENTAE			

实验方案及位点

（二）STR 数据库比对

本公司采用 DSMZ tools 进行细胞系比对,其中包含来自于 ATCC, DSMZ, JCRB 和 RIKEN 数据库的 2455 个细胞系 STR 数据。如果待检测细胞未收录于以上细胞库或这是自行建立的新细胞系将无法进行比对，用户需根据细胞分型结果自行与其他数据库进行比对。

签发日期：2019-04-24

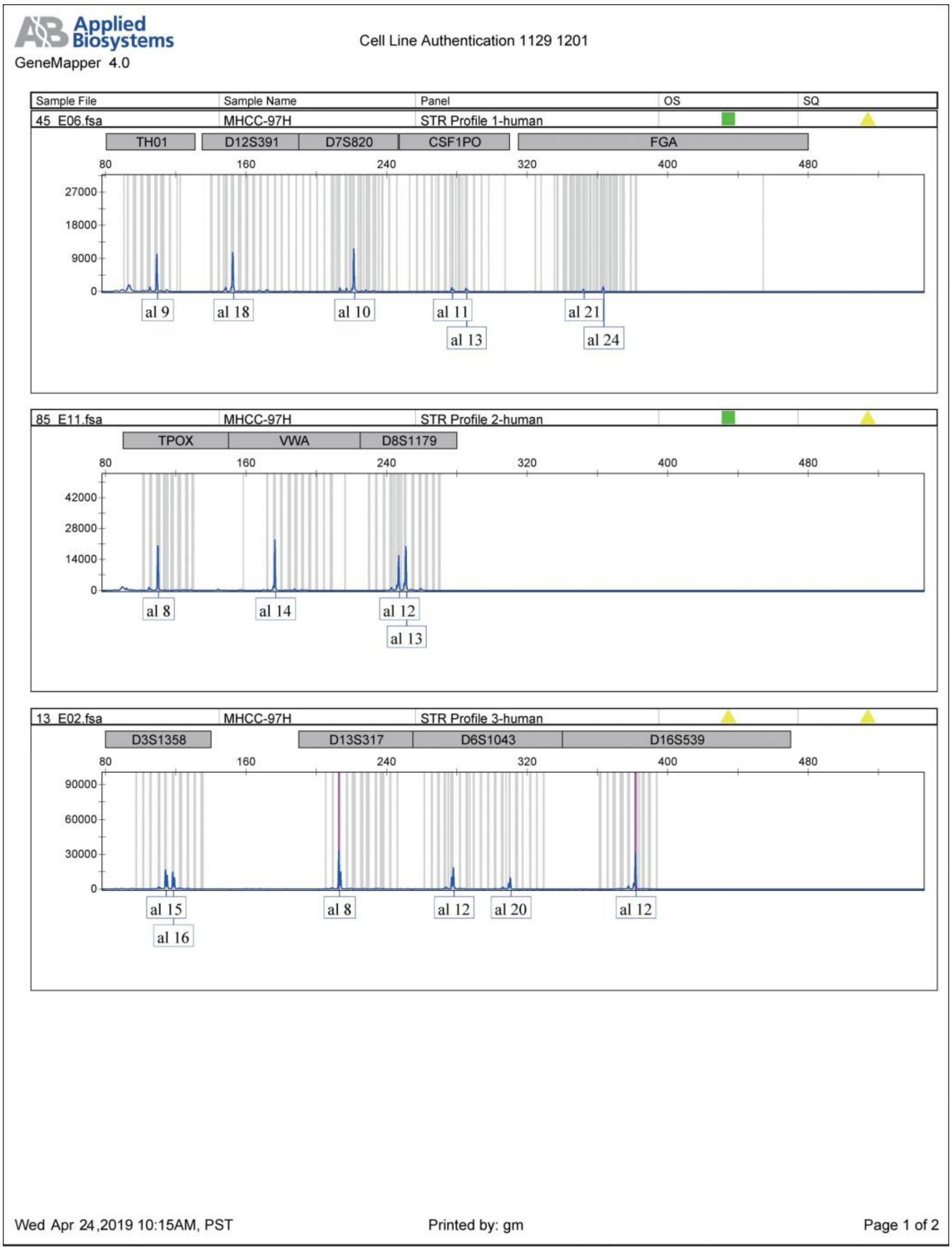
Applied Biosystems
GeneMapper 4.0
Cell Line Authentication 1129 1201
Sample File
Sample Name
Panel
OS
SQ
45_E06.fsa
MHCC-97H
STR Profile 1-human
TH01
D12S391
D7S820
CSF1PO
FGA
80
160
240
320
400
480
27000
18000
9000
0
al 9
al 18
al 10
al 11
al 13
al 21
al 24
85_E11.fsa
MHCC-97H
STR Profile 2-human
TPOX
VWA
D8S1179
42000
28000
14000
al 8
al 14
al 12
al 13
13_E02.fsa
MHCC-97H
STR Profile 3-human
D3S1358
D13S317
D6S1043
D16S539
90000
60000
30000
al 15
al 16
al 8
al 12
al 20
al 12
Wed Apr 24,2019 10:15AM, PST
Printed by: gm
Page 1 of 2

Applied Biosystems
GeneMapper 4.0

Cell Line Authentication 1129 1201

Sample File	Sample Name	Panel	OS	SQ
45_E06.fsa	MHCC-97H	STR Profile 4-human		

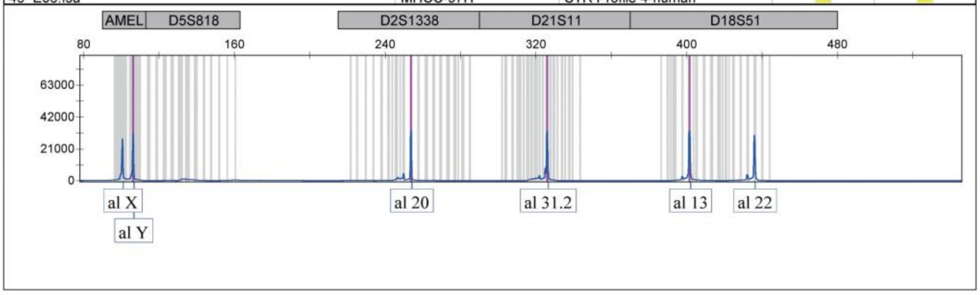

51_C07_DongJingJing1-5XiBaoJianDing1212.fsa	MHCC-97H	STR Profile 4-human		

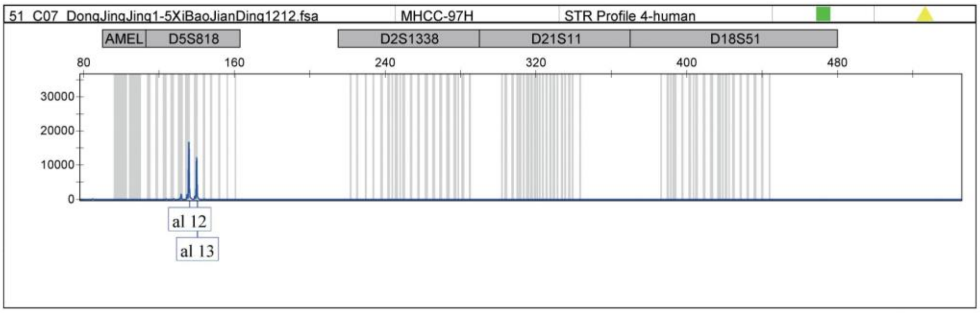

Wed Apr 24,2019 10:15AM, PST   Printed by: gm   Page 2 of 2

Applied Biosystems

GeneMapper 4.0

Cell Line Authentication 1129 1201

Sample File	Sample Name	Panel	OS	SQ
45_E06.fsa	MHCC-97H	STR Profile 1-human		

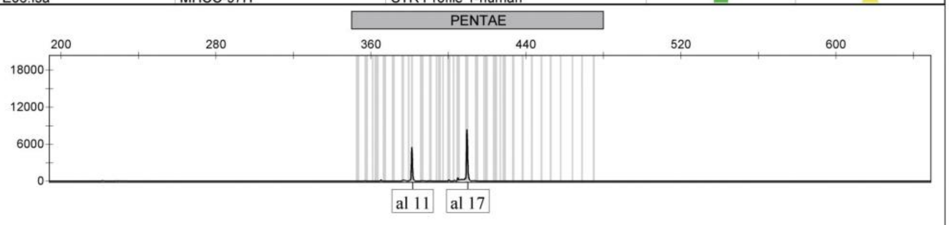

85_E11.fsa	MHCC-97H	STR Profile 2-human		

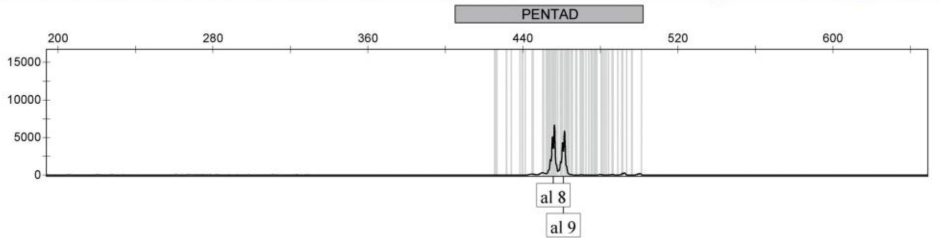

13_E02.fsa	MHCC-97H	STR Profile 3-human		

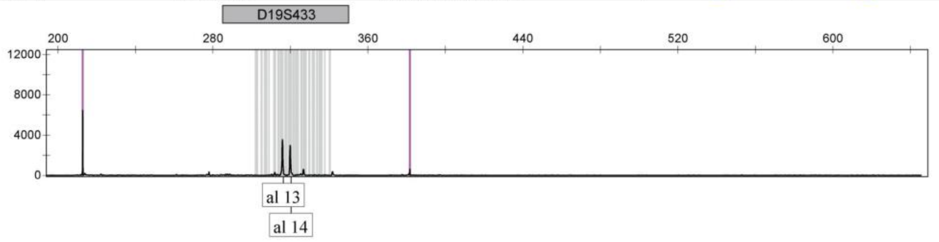

Wed Apr 24,2019 10:16AM, PST   Printed by: gm   Page 1 of 1

上海传秋生物科技有限公司

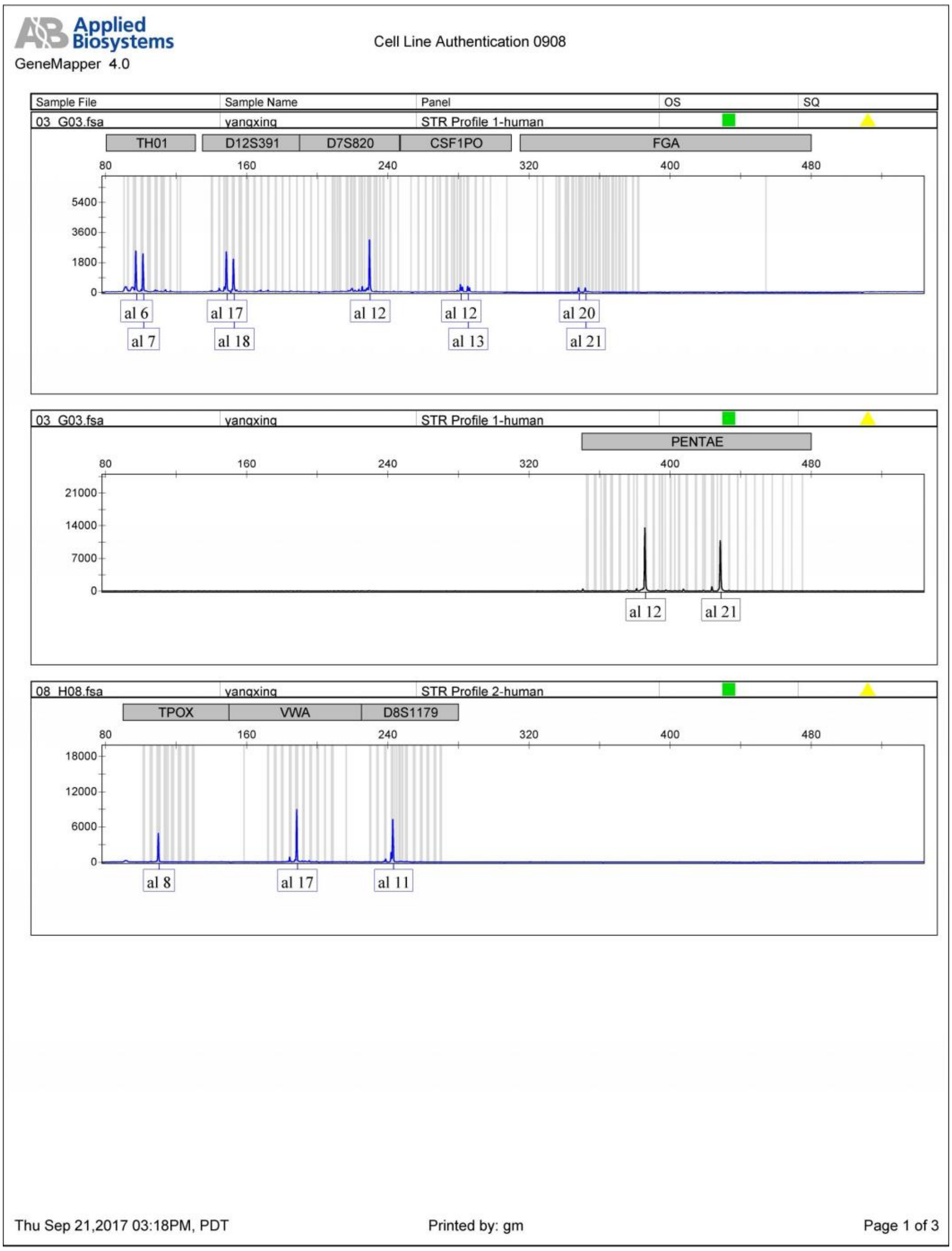

Applied Biosystems

GeneMapper 4.0

Cell Line Authentication 0908

Sample File	Sample Name	Panel	OS	SQ
08_H08.fsa	yangxing	STR Profile 2-human		

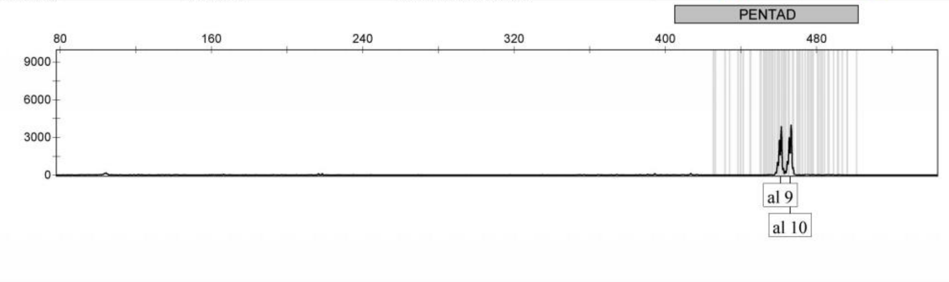

03_F03.fsa	yangxing	STR Profile 3-human		

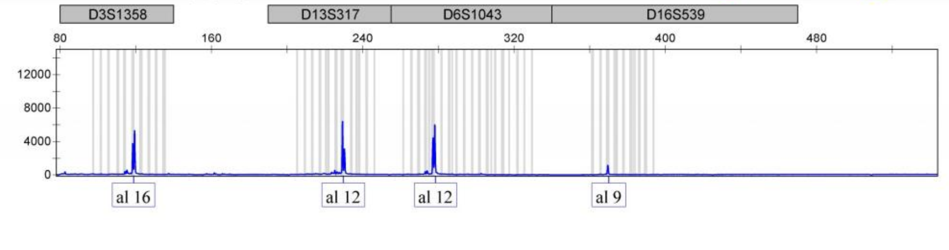

03_F03.fsa	yangxing	STR Profile 3-human		

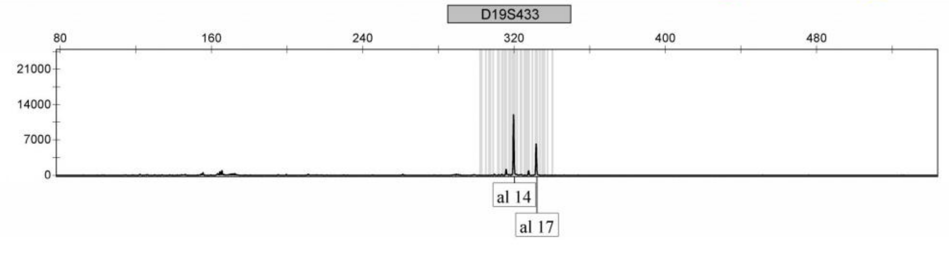

Thu Sep 21,2017 03:18PM, PDT   Printed by: gm   Page 2 of 3

Applied Biosystems

Cell Line Authentication 0908

GeneMapper 4.0

Sample File	Sample Name	Panel	OS	SQ
10_F10.fsa	yangxing	STR Profile 4-human		

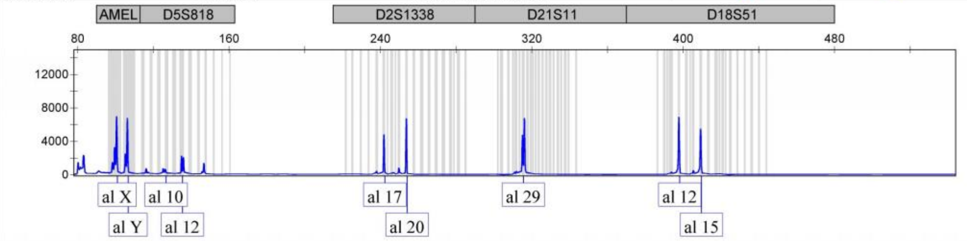

10_F10.fsa	yangxing	STR Profile 4-human		

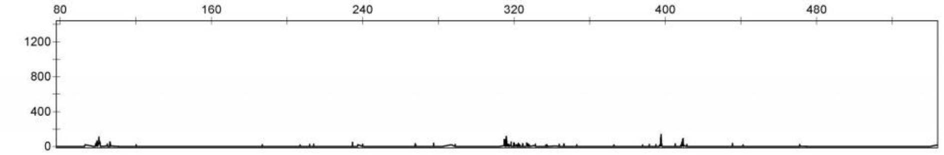

Thu Sep 21,2017 03:18PM, PDT

Printed by: gm

Page 3 of 3
