# Supplementary material for: RBM15 promotes hepatocellular carcinoma progression by regulating N6-methyladenosine modification of YES1 mRNA in an IGF2BP1-dependent manner
Source: Cell Death Discov. 2021 Oct 27;7:315. doi: 10.1038/s41420-021-00703-w (PMC8551180; doi:10.1038/s41420-021-00703-w)
Supplement: Supplementary file 2 — Supplementary methods [file 41420_2021_703_MOESM2_ESM.docx]

**Cell culture**

Human hepatocellular carcinoma cell lines Huh7 (RRID: CVCL_0336), HCC-LM3 (RRID: CVCL_6832), MHCC97H (RRID: CVCL_4972) were purchased from Shanghai Institutes of Biological Sciences (Shanghai, China). All cells were routinely cultured in the minimum necessary medium (MEM, BI, Israel) supplemented with 10% fetal bovine serum (FBS, BI), penicillin (100units/ml) and streptomycin (100g/ml), and cultured in a thermostatic incubator (Thermo Scientific, USA) with a humidified environment of 5% CO^2^ and 37 ℃.

**RNA extraction and RT-qPCR**

Total RNA was isolated using FastPure Cell/Tissue Total RNA Isolation Kit V2 （Vazyme Biotech, Nanjing, China）and cDNA was synthesized with HiScript II Q RT SuperMix for qPCR (Vazyme Biotech, Nanjing, China) according to the manufacturer’s protocol. Bio-Rad QX100 Droplet Digital PCR system (USA) was used to measure expression RNA by SYBR Green (Vazyme Biotech, Nanjing, China). The method for calculating the relative RNA amount was described in our previously study. All premiers were obtained from Tsingke Biological Technology (Beijing, China) and presented in Supplementary Table 1

**Western blotting**

Total proteins were extracted from tissues or cells using precooled RIPA buffer (Beyotime, Shanghai, China) containing protease and phosphatase inhibitors (Thermo Scientific, USA). Protein quantification was performed using the Bicinchoninic Acid protein assay kit (Thermo Scientific, USA). The same amount of protein samples was separated by 4-12% SDS-PAGE (GenScript, Nanjing, China) and then transferred to a 0.45 μm PVDF (Millipore, USA). After blocking with 5% skim milk in TBST buffer for 1 h, the membrane was incubated with the corresponding primary antibody at 4 ℃ overnight. After washing with TBST buffer for 3 times, the membrane was then incubated with enzyme-labeled secondary antibody at room temperature for 1 h. Imaging system (Bio-Rad, USA) and enhanced chemiluminescence detection kit (Servicebio, Wuhan, China) were used for Western blotting. GAPDH was chosen as loading control. All the antibodies used in the study were listed in Supplementary Table 2.

**Immunohistochemistry (IHC)**

The IHC staining of TMA cohort was applied to determine expression of RBM15 protein and the connection between RBM15 and prognosis of HCC patients. IHC intensity scores and percentage of positive cells was defined as we described in our previous study [10]. All score was assessed by two pathologists who were unaware of the prognosis of patients independently. Besides, subcutaneous tumor specimens from mice were fixed with formalin and embedded in paraffin. Then IHC staining of RBM15, YES1 and PCNA were performed on these tumors.

**RNA interference and plasmids.**

Small interfering RNAs (siRNA) against RBM15, YES1, IGF2BP1, IGF2BP3, YTHDF1 and negative control RNAs (siNC) were synthesized by Shangya Biotechnology (Hangzhou, China). pcDNA3.1-RBM15, pcDNA3.1-YES1 and empty vector were obtained from RPTbio (Hangzhou, China) for overexpression assays. Transient transfection was performed according to the manufacturer’s protocol by jetPRIME Polyplus Kit (France). All the sequences were summarized in Supplementary Table 3.

**Construction of stable knockdown cells**

Lentiviruses expression small hairpin RNAs (shRNAs) targeting RBM15 (shRBM15#1, shRBM15#2 and shNC) were obtained from Zorin(Shanghai, China). Huh7 and HCC-LM3 cells were applied to establish stable RBM15 knockdown models. Infected cells were selected by 3μg/ml puromycin for two weeks before subsequent assays. All the targeted sequences were listed in Supplementary Table 3.

**Cell proliferation assay, colony formation and EdU incorporation assay.**

Cell proliferation ability was determined by Cell Counting Kit-8 according to the manufacturer’s protocol. In colony formation assay, 1.5× 103 cells were plated in 6-well cell culture plate with three repetitions. After incubation of two weeks, the plates were fixed by paraformaldehyde and stained with 1% crystal violet for 10min. In Edu assay, A 5-ethynyl-20-deoxyuridine (EdU) assay kit (Ribobio, Guangzhou, China) was used to evaluate the proliferation ability of HCC cells according to the manufacturer’s protocol. The results were visualized by a fluorescence microscope.

**Migration and invasion assays**

In migration or invasion assays, a 24-well plate was used with a transwell filter insert (Corning, NY, USA) with a pore size of 8 μm. In the invasion experiment, diluted matrix was added to the transwell filter insert in advance. 8 × 10^4^ HCC cells in serum-free medium were placed in the upper cavity, and then the medium containing 10% fetal bovine serum was added to the lower cavity. After 48 h (migration) or 72 h (invasion) culture at 37 ° C, the submembranous cells were fixed and stained with crystal violet. Then cell counts in five random domains were then performed under the microscope.

**Subcutaneous xenograft experiments**

Four-week-old male Balb/c nude mice were obtained from Shanghai Experimental Animal Center of Chinese Academic of Sciences (Shanghai,China). 5 × 10^6^ Huh7 and HCC-LM3 cells resuspended in 100μl PBS were subcutaneously injected to the left flank of the mice (randomly selected, five mice per group for Huh7 cells in the first time and ten mice per group for HCC-LM3 cells in the second time. No blinding was performed). Tumor sizes were measured regularly. After feeding of more than 3 weeks, mice were sacrificed and tumors were surgically dissected for histology analyses. The tumor volume was calculated with the equation: (length×width^2^)/2. The animal experiments were approved by the Ethics Committee for Laboratory Animals of the First Affiliated Hospital, Zhejiang University.

**RNA immunoprecipitation (RIP)**

Magna RIP kit (Millipore, Germany) was applied to conduct RIP assay in accordance with manufacturer’s recommendation. In Brief, magnetic beads were mixed with anti-IGF2BP1 (Abclonal, China) and anti-rabbit IgG (Millipore, Germany) and added to sufficient cell lysates. Then, target RNA-protein complexes were eluted and purified for qPCR.

**Luciferase reporter assay**

cDNAs containing 3’ UTR sequence of YES1 were cloned into luciferase reporter vectors (pcDNA3.1 vector including firefly and renilla luciferase). For mutant report plasmids, two adenosine (A) in m6A sties were replaced with cytosine (C). RBM15-knockdown HCC cells were transfected with wild-type or mutated YES1 reporter plasmids. After 24h, the luciferase activity was tested using Dual Luciferase Reporter Assay Kit (Vazyme Biotech Co.,Ltd, China) The inserted sequences were listed Supplementary Table 4

**RNA decay assay**

RNA decay assay was performed to evaluate RNA stability. HCC cells were cultured in 6-well plates followed by treatment of RBM15 knock-down. Actinomycin D (MCE, HY-17559) was added into each well. After 0, 12 and 24h, we collected cells to quantify the relative abundance of YES1 mRNA (relative to 0h).

**Computer code for Rstudio**

library(rms)

library(foreign)

library(survival)

setwd("C:/R")

data<-read.csv("rbm15.csv")

View(data)

str(data)

data$TNM<-factor(data$TNM,labels=c('I', 'II', 'III', 'IV'))

data$RBM15<-factor(data$RBM15,labels=c('Low','High'))

data$Age<-factor(data$Age,labels=c('≤73','＞73'))

str(data)

dev<- data

y<-Surv(dev$OS,dev$Status ==1,type="right")

mod1<-coxph(y~ RBM15 + TNM+Age, data=dev)

summary(mod1)

y<-Surv(dev$OS,dev$Status ==1,type="right")

mod2<-coxph(y~ TNM, data=dev)

summary(mod2)

ddist <- datadist(dev)

options(datadist='ddist')

units(dev$OS) <- "Months"

fcox1 <- cph(Surv(OS, Status) ~ RBM15 + TNM+Age, surv=T,x=T, y=T,data=dev)

med <- Quantile(fcox1)

nom.sur1 <- nomogram(fcox1, fun=function(x) med(lp=x), funlabel="Median Survival Time",lp=F)

plot(nom.sur1)

surv <- Survival(fcox1)

nom1 <- nomogram(fcox1, fun=list(function(x) surv(36, x), function(x) surv(60, x)), funlabel=c("3-years Survival Probability", "5-years Survival Probability"),lp=F)

plot(nom1)

library(nomogramEx)

nomogramEx(nomo=nom1, np=2, digit=9)

dev$RBM15point <- ifelse(dev$RBM15=="Low",0, 19.45)

dev$TNMpoint <- ifelse(dev$TNM=="I",0, ifelse(dev$TNM=="II",13.75, ifelse(dev$TNM=="III",52.29, 100)))

dev$Agepoint <- ifelse(dev$Age=="≤73",0, 27.89)

dev$points1 <- dev$RBM15point + dev$Agepoint + dev$TNMpoint

dev$RBM15point <- ifelse(dev$RBM15=="Low",0, 100)

dev$TNMpoint <- ifelse(dev$TNM=="I",44.05, ifelse(dev$TNM=="II",0, ifelse(dev$TNM=="III",42.68, 90)))

dev$points1 <- dev$RBM15point + dev$TNMpoint

fcox2 <- cph(Surv(OS, Status) ~ TNM, surv=T,x=T, y=T,data=dev)

med <- Quantile(fcox2)

nom.sur2 <- nomogram(fcox2, fun=function(x) med(lp=x), funlabel="Median Survival Time",lp=F)

plot(nom.sur2)

surv <- Survival(fcox2)

nom2 <- nomogram(fcox2, fun=list(function(x) surv(36, x), function(x) surv(60, x)), funlabel=c("3-years Survival Probability", "5-years Survival Probability"),lp=F)

plot(nom2)

library(nomogramEx)

nomogramEx(nomo=nom2, np=2, digit=9)

source("stdca.R") dev$three.years.Survival.Probabilitynew1=c(summary(survfit(mod1,newdata=dev),times=36)$surv)

dev$three.years.Survival.Probabilitynew2=c(summary(survfit(mod2,newdata=dev),times=36)$surv)

library(survival)

library(survivalROC)

nobs<- NROW(dev)

cutoff1<- 36

SROC1= survivalROC(Stime = dev$OS, status = dev$Status, marker = dev$points1, predict.time =cutoff1, method= "KM" )

cut.op1= SROC1$cut.values[which.max(SROC1$TP-SROC1$FP)]

cut.op1 #

plot(SROC1$FP,SROC1$TP, type="l", xlim=c(0,1), ylim=c(0,1),

xlab = paste( " False Positive","\n", "AUC = ",round(SROC1$AUC,3)),

ylab = " True Positive", col="red")

abline(0,1)

legend("bottomright",c("ROC curve of 3-years overall survival"),col="red",lty=c(1,1))

SROC2= survivalROC(Stime = dev$OS, status = dev$Status, marker = dev$points2, predict.time =cutoff1, method= "KM" )

cut.op2= SROC2$cut.values[which.max(SROC2$TP-SROC2$FP)]

cut.op2 #

plot(SROC2$FP,SROC2$TP, type="l", xlim=c(0,1), ylim=c(0,1),

xlab = paste( " False Positive ","\n", "AUC = ",round(SROC2$AUC,3)),

ylab = " True Positive", col="blue")

abline(0,1)

legend("bottomright",c("ROC curve of 3-years overall survival"),col="blue",lty=c(1,1))

nobs<- NROW(dev)

cutoff1<- 60

SROC1= survivalROC(Stime = dev$OS, status = dev$Status, marker = dev$points1, predict.time =cutoff1, method= "KM" )

cut.op1= SROC1$cut.values[which.max(SROC1$TP-SROC1$FP)]

cut.op1

plot(SROC1$FP,SROC1$TP, type="l", xlim=c(0,1), ylim=c(0,1),

xlab = paste( " False Positive","\n", "AUC = ",round(SROC1$AUC,3)),

ylab = " True Positive", col="red")

abline(0,1)

legend("bottomright",c("ROC curve of 5-years overall survival"),col="red",lty=c(1,1))

SROC2= survivalROC(Stime = dev$OS, status = dev$Status, marker = dev$points2, predict.time =cutoff1, method= "KM" )

cut.op2= SROC2$cut.values[which.max(SROC2$TP-SROC2$FP)]

cut.op2

plot(SROC2$FP,SROC2$TP, type="l", xlim=c(0,1), ylim=c(0,1),

xlab = paste( " False Positive ","\n", "AUC = ",round(SROC2$AUC,3)),

ylab = " True Positive", col="blue")

abline(0,1)

legend("bottomright",c("ROC curve of 5-years overall survival"),col="blue",lty=c(1,1))

library(timeROC)

model1ROC <- timeROC(T=dev$OS,delta=dev$Status,marker=dev$points1,cause=1, weighting = "marginal",times=c(12, 24, 36,48, 60),iid=TRUE)

plotAUCcurve(model1ROC)

model2ROC <- timeROC(T=dev$OS,delta=dev$Status,marker=dev$points2,cause=1, weighting = "marginal",times=c(12, 24, 36,48, 60),iid=TRUE)

plotAUCcurve(model2ROC)

plotAUCcurve(model1ROC,conf.int=F,col="red")

plotAUCcurve(model2ROC,conf.int=F,col="blue",add=TRUE)

legend("topright",c("RBM15 model","TNM model"),col=c("red","blue"),lty=1,lwd=2)
