# Supplementary material for: RBM15 promotes hepatocellular carcinoma progression by regulating N6-methyladenosine modification of YES1 mRNA in an IGF2BP1-dependent manner
Source: Cell Death Discov. 2021 Oct 27;7:315. doi: 10.1038/s41420-021-00703-w (PMC8551180; doi:10.1038/s41420-021-00703-w)
Supplement: Supplementary file 3 — Supplementary figure legends [file 41420_2021_703_MOESM3_ESM.docx]

Figure S1 **The ROC curves represented the discrimination of models measured by the C-index**. **a**. for 3-years overall survival (red curve for RBM15 model and blue curve for TNM model); **b** for 5-years overall survival (red curve for RBM15 model and blue curve for TNM model)

Figure S2 **Knockdown efficiency on HCC cells via qPCR.** **a** The efficiency of RBM15 knockdown on Huh7, HCC-LM3, MHCC97H and SNU449; **b** The efficiency of YES1 Knockdown on HCC-LM3 and MHCC97H. **c** The efficiency of stable RBM15 Knockdown on Huh7 and HCC-LM3 (** p＜0.01, *** p＜0.001, **** p＜0.0001; and *t*-test). The data are presented as means ± SD.

Figure S3 **RBM15 promotes tumor growth and migration / invasion capability of HCC cell in vitro.** **a** Negative control or siRNA (si-RBM15#1 and #2) was transfected into Huh7. The efficiency of knockdown was tested by western blotting and the proliferation capacities of HCC cells were detected by CCK-8 and colony formation assays (** p＜0.01, *** p＜0.001, **** p＜0.0001; two-way ANOVA and *t*-test); **b** Edu assay was applied to compare the proliferation abilities of Huh7 cells(scale bar, 200μm); Bar charts showed the percentage of cells in S phage based on the results of Edu assay (** p＜0.01; *t*-test); **c** Wound healing assays were performed to compared the migration capabilities of Huh7 cells (scale bars, 200μm); The percentage of healed area were quantified by bar charts (* p＜0.1, *** p＜0.001, **** p＜0.0001; *t*-test); **d** Transwell assays were applied to detect the migration and invasion abilities of Huh7 cells after silencing RBM15 (scale bars, 200μm); Bar charts showed the relative count of HCC cells which passed through the chamber membranes when referred to negative control groups (*** p＜0.001, **** p＜0.0001; *t*-test); The data are presented as means ± SD.

Figure S4 **The results of GO analysis for the identification of DEGs in HCC.** **a** Biological process; **b** Cellular component; **c** Molecular function

Figure S5 **other results for identifying the “reader”. a** Expression of IGF2BP1 between tumor and normal tissues in HCC; **b** expression of IGF2BP3 between tumor and normal tissues in HCC; **c** expression of YTHDF1 between tumor and normal tissues in HCC; **d** YES1 was positively associated with IGF2BP1 expression; **e** High expression of IGF2BP1 indicated worse prognosis; **f** The alteration of YES1 after knockdown of IGF2BP3 and YTHDF1 (* p＜0.05, ** p＜0.01, *** p＜0.001; *t*-test); The data are presented as means ± SD.

Figure S6 **YES1 is associated with** **immune infiltration**. **a** YES1 and B cell; **b** YES1 and CD8^+^ T cell; **c** YES1 and CD4^+^ T cell; **d** YES1 and Macrophage; **e** YES1 and Neutrophil; **f** YES1 and Dendritic cell.
