# Supplementary figures and images for: RBM15 promotes hepatocellular carcinoma progression by regulating N6-methyladenosine modification of YES1 mRNA in an IGF2BP1-dependent manner
Source: Cell Death Discov. 2021 Oct 27;7:315. doi: 10.1038/s41420-021-00703-w (PMC8551180; doi:10.1038/s41420-021-00703-w)

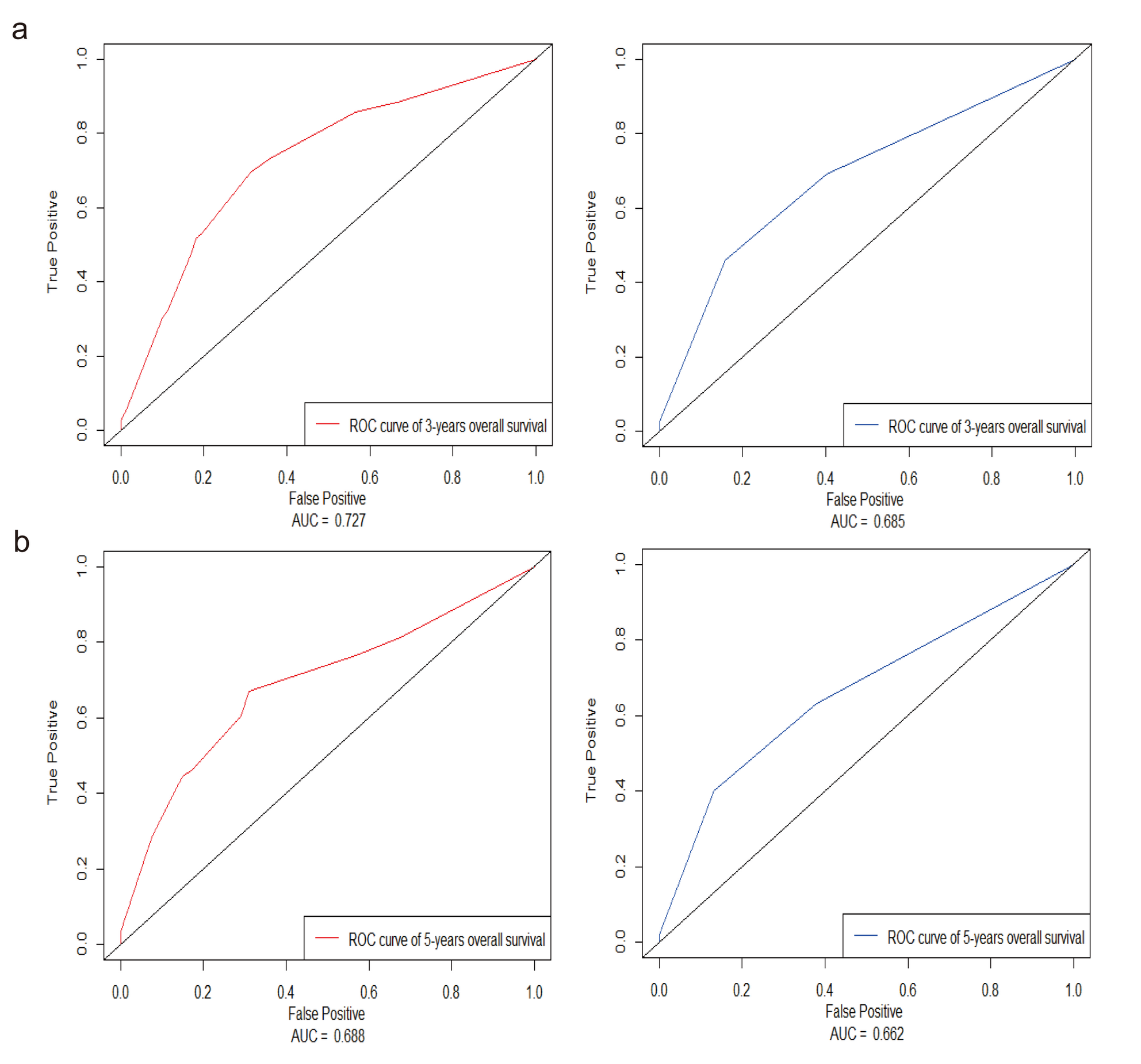

Supplement: Supplementary file 4 — Supplementary figure 1 [file 41420_2021_703_MOESM4_ESM.tif]

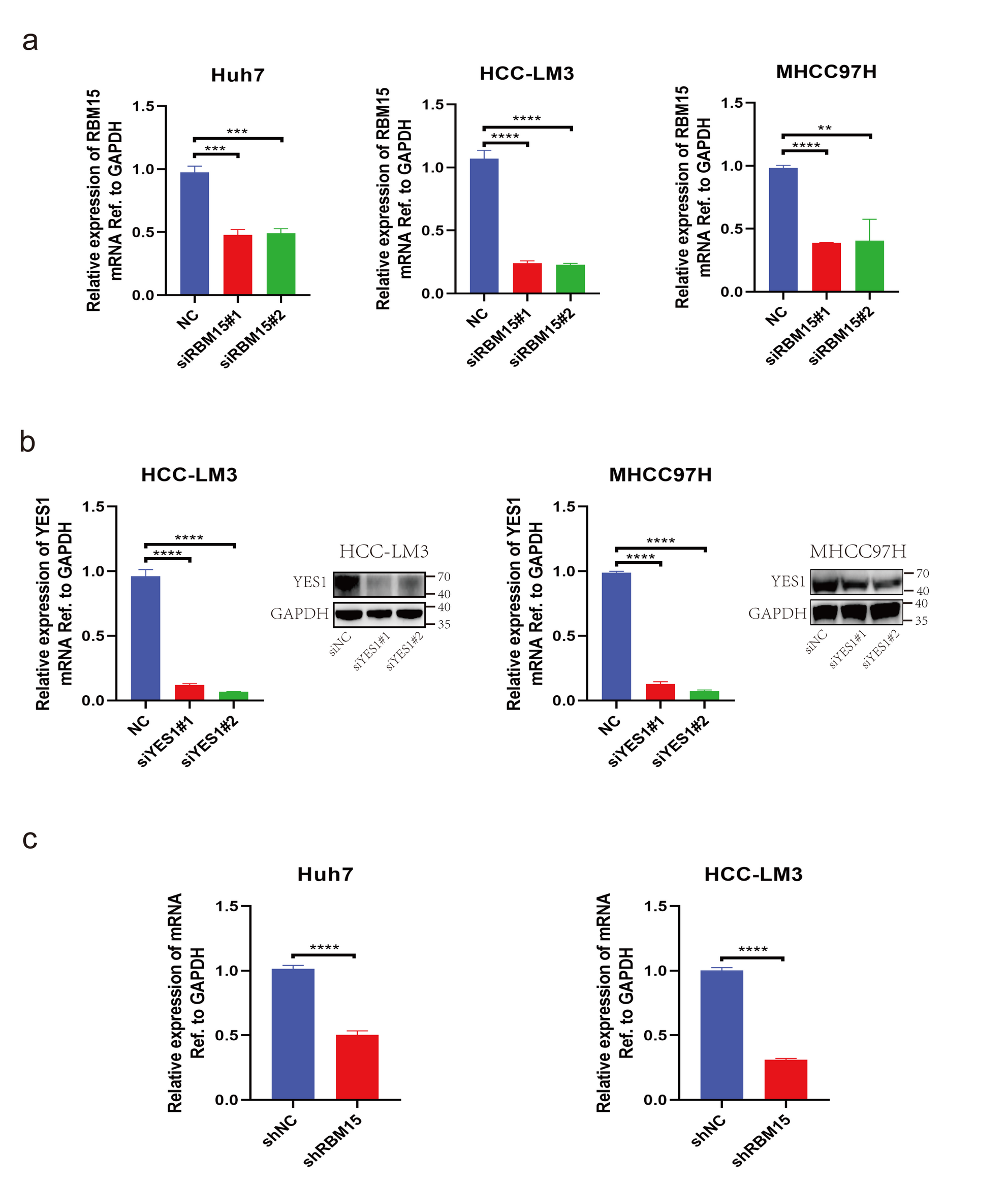

Supplement: Supplementary file 5 — Supplementary figure 2 [file 41420_2021_703_MOESM5_ESM.tif]

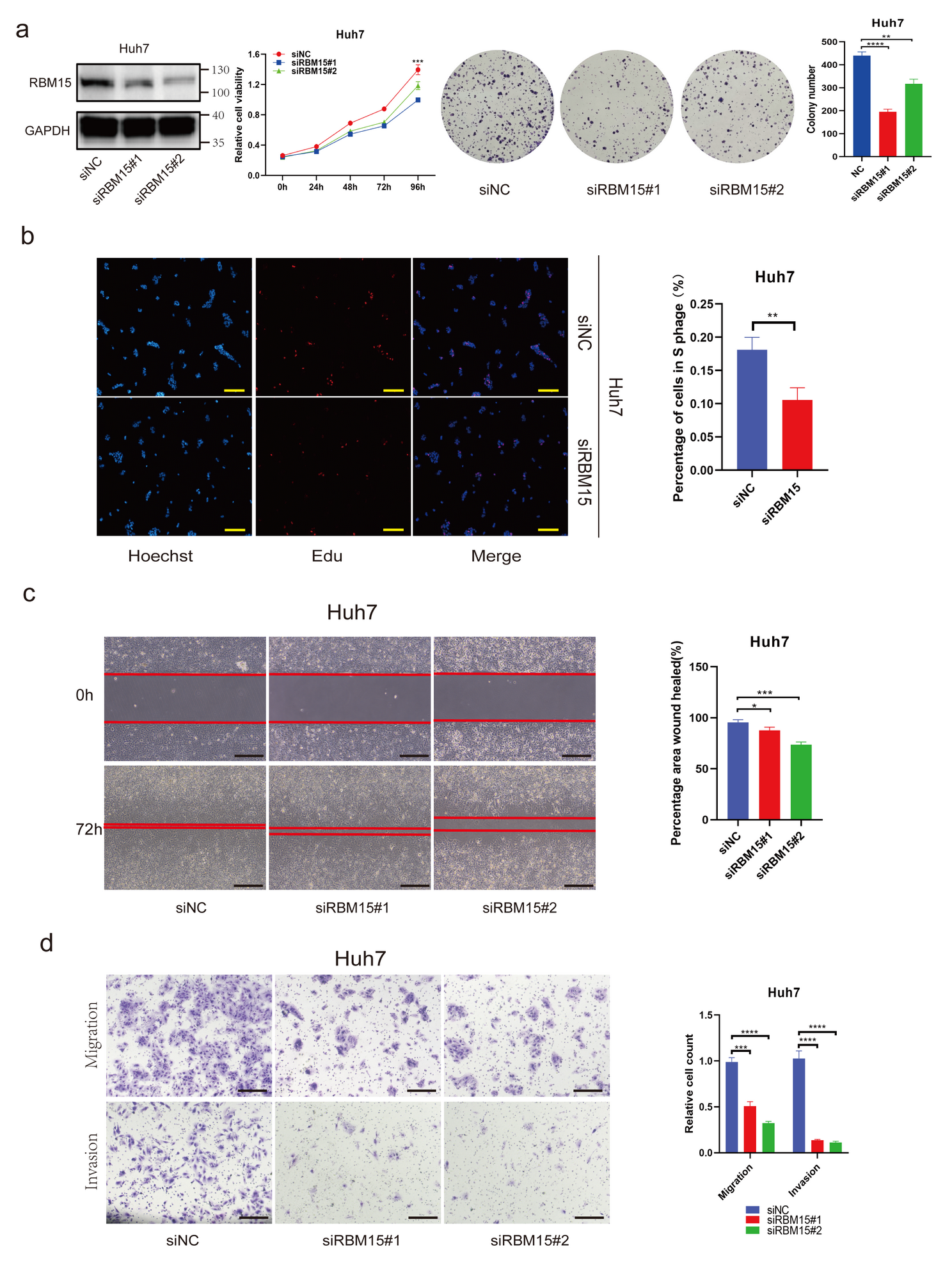

Supplement: Supplementary file 6 — Supplementary figure 3 [file 41420_2021_703_MOESM6_ESM.tif]

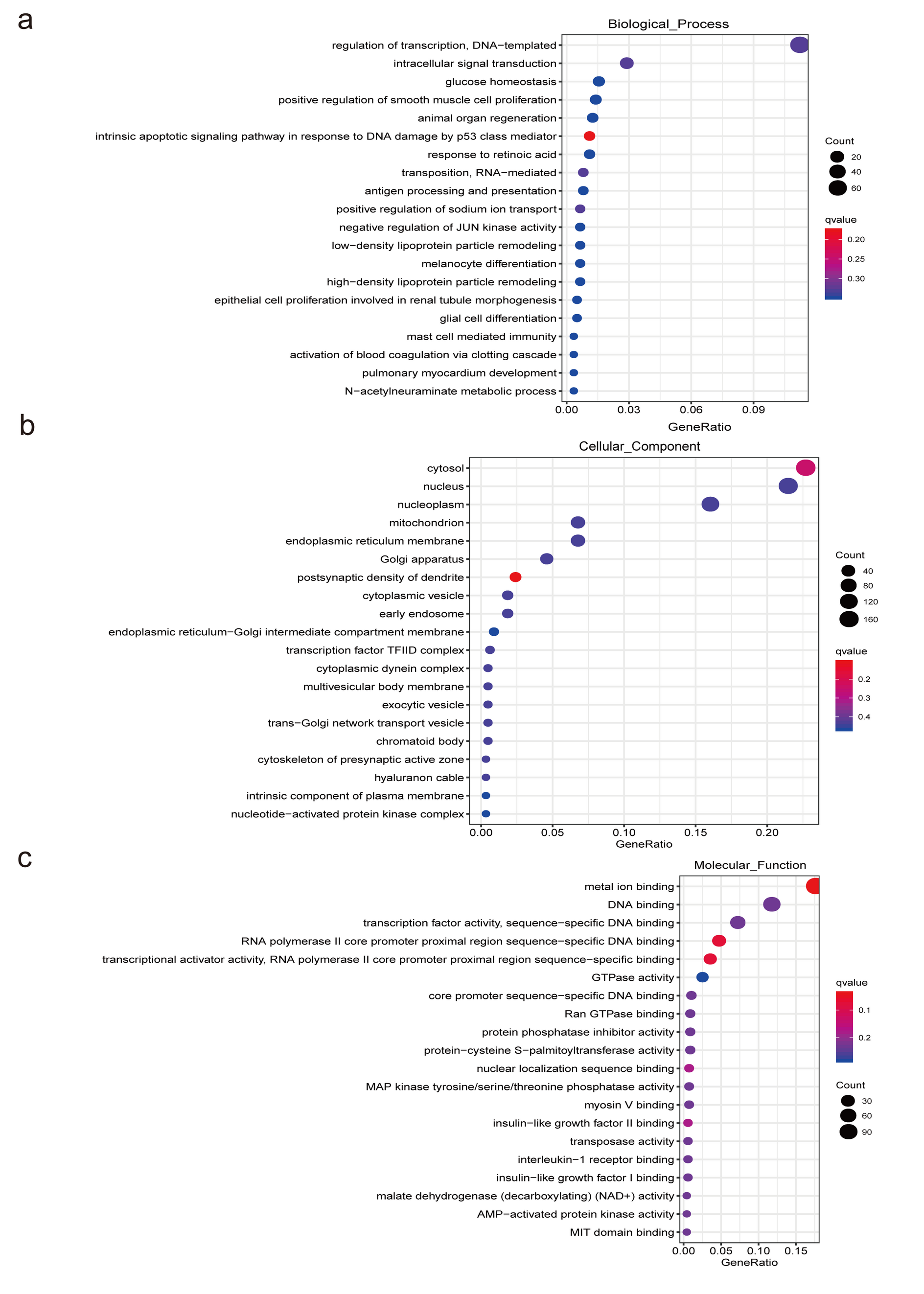

Supplement: Supplementary file 7 — Supplementary figure 4 [file 41420_2021_703_MOESM7_ESM.tif]

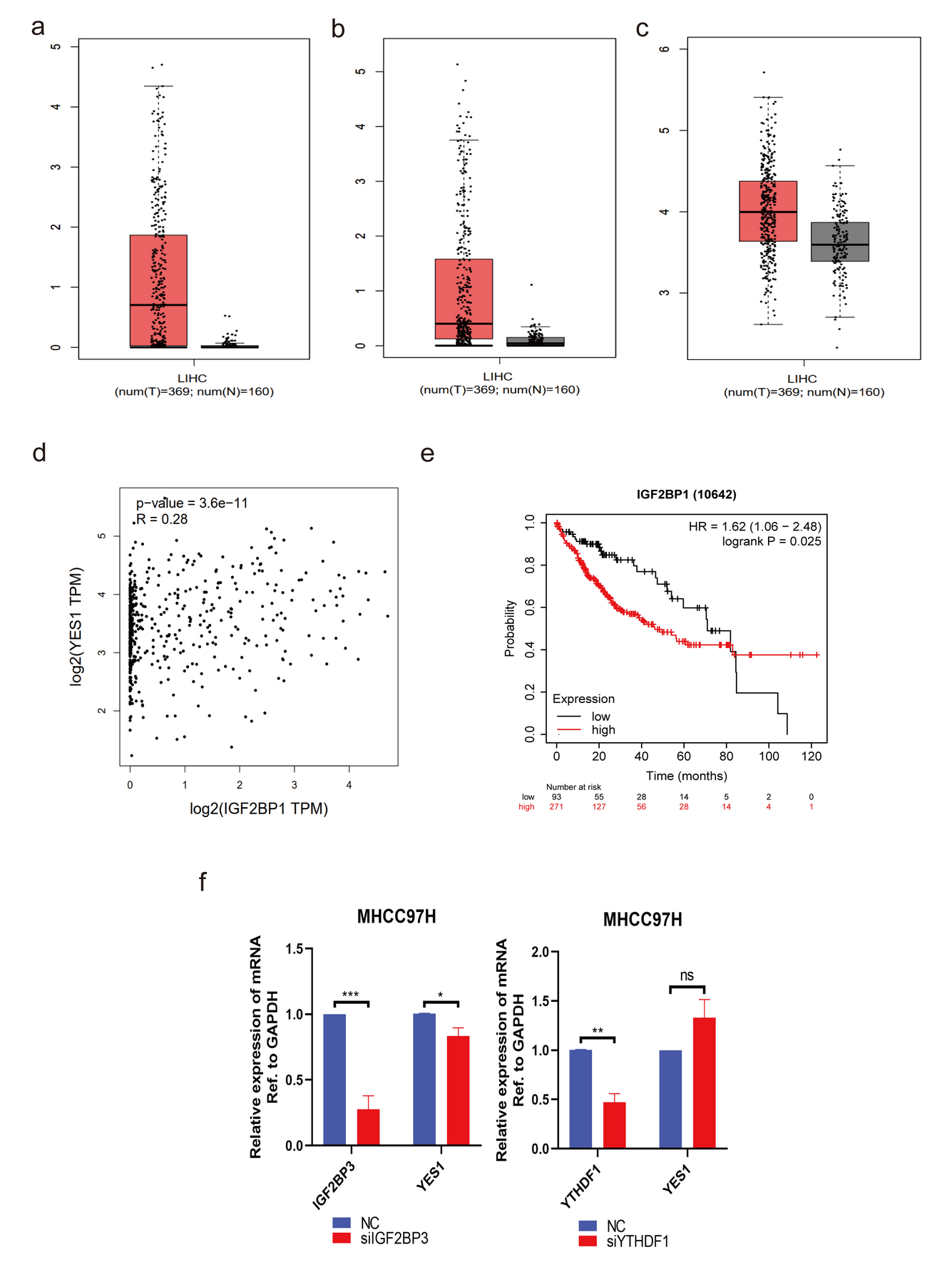

Supplement: Supplementary file 8 — Supplementary figure 5 [file 41420_2021_703_MOESM8_ESM.tif]

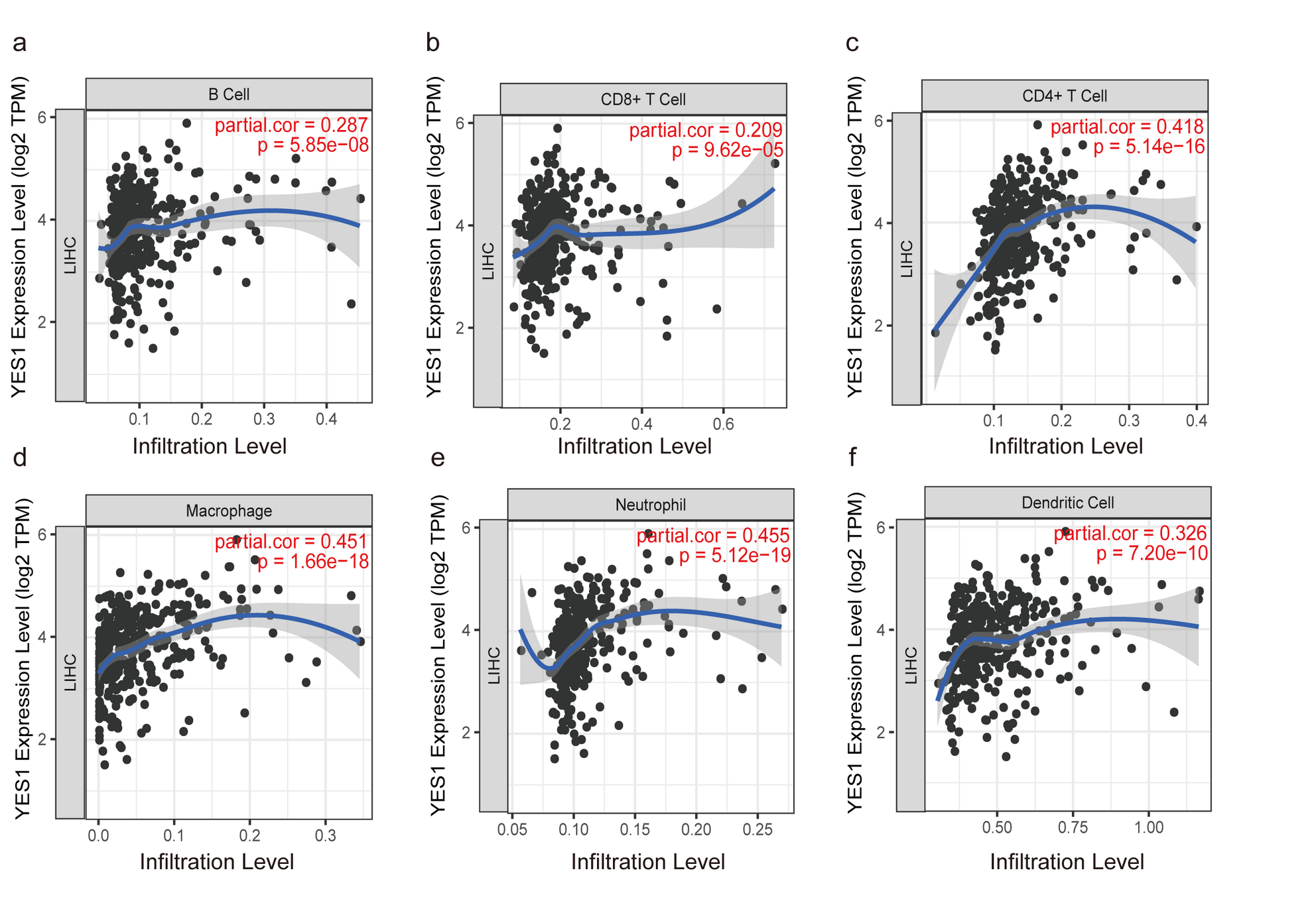

Supplement: Supplementary file 9 — Supplementary figure 6 [file 41420_2021_703_MOESM9_ESM.tif]
