# Supplementary material for: RBM15 promotes hepatocellular carcinoma progression by regulating N6-methyladenosine modification of YES1 mRNA in an IGF2BP1-dependent manner
Source: Cell Death Discov. 2021 Oct 27;7:315. doi: 10.1038/s41420-021-00703-w (PMC8551180; doi:10.1038/s41420-021-00703-w)
Supplement: Supplementary file 10 — supplementary table 1 [file 41420_2021_703_MOESM10_ESM.docx]

Supplementary Table 1. Primers employed in this study

| Gene symbol | Forward primer | Reverse primer |
| --- | --- | --- |
| GAPDH | GGAGCGAGATCCCTCCAAAAT | GGCTGTTGTCATACTTCTCATGG |
| RBM15 | AGCCGCGAGTATGATACCG | GCCCGAAGAATTTTTGGTGCTC |
| YES1 | CTCAGGGGTAACGCCTTTTGG | CACCACCTGTTAAACCAGCAG |
| ARL1 | TCTGTTTGGAACTCGGGAAATG | AAGGTTTTTGTACGTCACCGT |
| YES1-MeRIP-primer | TGCACAAATCTGCCAAAATATAAAG | TTTGTGCAACCATATCTGGGA |
| IGF2BP1 | CAAAGGAGCCGGAAAATTCAAAT | CGTCTCACTCTCGGTGTTCA |
| IGF2BP3 | TATATCGGAAACCTCAGCGAGA | GGACCGAGTGCTCAACTTCT |
| YTHDF1 | ACCTGTCCAGCTATTACCCG | TGGTGAGGTATGGAATCGGAG |
