# Supplementary material for: RBM15 promotes hepatocellular carcinoma progression by regulating N6-methyladenosine modification of YES1 mRNA in an IGF2BP1-dependent manner
Source: Cell Death Discov. 2021 Oct 27;7:315. doi: 10.1038/s41420-021-00703-w (PMC8551180; doi:10.1038/s41420-021-00703-w)
Supplement: Supplementary file 11 — supplementary table 2 [file 41420_2021_703_MOESM11_ESM.docx]

Supplementary Table 2. Antibody employed in this study

| Antibody | Source | Identifier | RRID |
| --- | --- | --- | --- |
| GAPDH | Proteintech | Cat#10494-1-AP | AB_2263076 |
| RBM15 | Cell Signaling Technology | Cat#A4936 | AB_2765940 |
| Anti-m6A antibody | Synaptic systems | Cat#202003 | AB_2279214 |
| YES1 | Proteintech | Cat#20243-1-AP | AB_10697656 |
| p-Src | Cell Signaling Technology | Cat# 2113 | AB_2106051 |
| t-p38 | Cell Signaling Technology | Cat#8690T | AB_10999090 |
| p-p38 | Cell Signaling Technology | Cat#4511T | AB_2139682 |
| t-p44/p42 (Erk1/2) | Cell Signaling Technology | Cat#4695T | AB_390779 |
| p-p44/p42 | Cell Signaling Technology | Cat#4370T | AB_2315112 |
| IGF2BP1 (RIP) | ABclonal | Cat#A13581 | AB_2760443 |
| PCNA (IHC) | Cell Signaling Technology | Cat#13110 | AB_2636979 |
