# Supplementary material for: RBM15 promotes hepatocellular carcinoma progression by regulating N6-methyladenosine modification of YES1 mRNA in an IGF2BP1-dependent manner
Source: Cell Death Discov. 2021 Oct 27;7:315. doi: 10.1038/s41420-021-00703-w (PMC8551180; doi:10.1038/s41420-021-00703-w)
Supplement: Supplementary file 12 — supplementary table 3 [file 41420_2021_703_MOESM12_ESM.docx]

Supplementary Table 3. RNA interference and plasmids employed in this study

| Gene symbol | Targeted sequence |
| --- | --- |
| siRBM15#1 | GGUGAUAGUUGGGCAUAUA |
| siRBM15#2 | UAGCAGGGCCCAAUGGUUA |
| shRBM15 | GGUGAUAGUUGGGCAUAUA |
| siYES1#1 | CCCACUACAGUGUCACCAU |
| siYES1#2 | GGUGGAUACUAUAUCACAA |
| siIGF2BP1#1 | CUCCAAAGUUCGUAUGGUUAU |
| siIGF2BP1#2 | UGAAGAUCCUGGCCCAUAAUA |
| siIGF2BP3#1 | CGGUGAAUGAACUUCAGAAUU |
| siIGF2BP3#2 | GCUGCUGAGAAGUCGAUUACU |
| siYTHDF1#1 | CCGCGUCUAGUUGUUCAUGAA |
| siYTHDF1#2 | CAGGCUGGAGAAUAACGACAA |
| RBM15 pcDNA 3.1 | ATGAGGACTGCGGGGCGGGACCCTGTGCCGCGGCGGAGTCCAAGATGGCGGCGTGCGGTTCCGCTGTGTGAAACGAGCGCGGGGCGGCGGGTTACTCAGCTCCGCGGAGACGACCTCCGACGACCCGCAACAATGAAGGGAAAAGAGCGCTCGCCAGTGAAGGCCAAACGCTCCCGTGGTGGTGAGGACTCGACTTCCCGCGGTGAGCGGAGCAAGAAGTTAGGGGGCTCTGGTGGCAGCAATGGGAGCAGCAGCGGAAAGACCGATAGCGGCGGTGGGTCGCGGCGGAGTCTCCACCTGGACAAGTCCAGCAGTCGAGGTGGCAGCCGCGAGTATGATACCGGTGGGGGCAGCTCCAGTAGCCGCTTGCATAGTTATAGCTCCCCGAGCACCAAAAATTCTTCGGGCGGGGGCGAGTCGCGCAGCAGCTCCCGGGGTGGAGGCGGGGAGTCACGTTCCTCTGGGGCCGCCTCCTCAGCTCCCGGCGGCGGGGACGGCGCGGAATACAAGACTCTGAAGATAAGCGAGTTGGGGTCCCAGCTTAGTGACGAAGCGGTGGAGGACGGCCTGTTTCATGAGTTCAAACGCTTCGGTGATGTAAGTGTGAAAATCAGTCATCTGTCGGGTTCTGGCAGCGGGGATGAGCGGGTAGCCTTTGTGAACTTCCGGCGGCCAGAGGACGCGCGGGCGGCCAAGCATGCCAGAGGCCGCCTGGTGCTCTATGACCGGCCTCTGAAGATAGAAGCTGTGTATGTGAGCCGGCGCCGCAGCCGCTCCCCTTTAGACAAAGATACTTATCCTCCATCAGCCAGTGTGGTCGGGGCCTCTGTAGGTGGTCACCGGCACCCCCCTGGAGGTGGTGGAGGCCAGAGATCACTTTCCCCTGGTGGCGCTGCTTTGGGATACAGAGACTACCGGCTGCAGCAGTTGGCTCTTGGCCGCCTGCCCCCTCCACCTCCGCCACCATTGCCTCGAGACCTGGAGAGAGAAAGAGACTACCCGTTCTATGAGAGAGTGCGCCCTGCATACAGTCTTGAGCCAAGGGTGGGAGCTGGAGCAGGTGCTGCTCCTTTCAGAGAAGTGGATGAGATTTCACCCGAGGATGATCAGCGAGCTAACCGGACGCTCTTCTTGGGCAACCTAGACATCACTGTAACGGAGAGTGATTTAAGAAGGGCGTTTGATCGCTTTGGAGTCATCACAGAAGTAGATATCAAGAGGCCTTCTCGCGGCCAGACTAGTACTTACGGCTTTCTCAAATTTGAGAACTTAGATATGTCTCACCGGGCCAAATTAGCAATGTCTGGCAAAATTATAATTCGGAATCCTATCAAAATTGGTTATGGTAAAGCTACACCCACCACCCGCCTCTGGGTGGGAGGCCTGGGACCTTGGGTTCCTCTTGCTGCCCTGGCACGAGAATTTGATCGATTTGGCACCATACGCACCATAGACTACCGAAAAGGTGATAGTTGGGCATATATCCAGTATGAAAGCCTGGATGCAGCGCATGCTGCCTGGACCCATATGCGGGGCTTCCCACTTGGTGGCCCAGATCGACGCCTTAGAGTAGACTTTGCCGACACCGAACATCGTTACCAGCAGCAGTATCTGCAGCCTCTGCCCTTGACTCATTATGAGCTGGTGACAGATGCTTTTGGACATCGGGCACCAGACCCTTTGAGGGGTGCTCGGGATAGGACACCACCCTTACTATACAGAGATCGTGATAGGGACCTTTATCCTGACTCTGATTGGGTGCCACCCCCACCCCCAGTCCGAGAACGCAGCACTCGGACTGCAGCTACTTCTGTGCCTGCTTACGAGCCACTGGATAGCCTAGATCGCAGGCGGGATGGTTGGTCCTTGGACCGGGACAGAGGTGATCGAGATCTGCCCAGCAGCAGAGACCAGCCTAGGAAGCGAAGGCTGCCTGAGGAGAGTGGAGGACGTCATCTGGATAGGTCTCCTGAGAGTGACCGCCCACGAAAACGTCACTGCGCTCCTTCTCCTGACCGCAGTCCAGAATTGAGCAGTAGCCGGGATCG |
| YES1 pcDNA 3.1 | GACGGATCGGGAGATCTCCCGATCCCCTATGGTGCACTCTCAGTACAATCTGCTCTGATGCCGCATAGTTAAGCCAGTATCTGCTCCCTGCTTGTGTGTTGGAGGTCGCTGAGTAGTGCGCGAGCAAAATTTAAGCTACAACAAGGCAAGGCTTGACCGACAATTGCATGAAGAATCTGCTTAGGGTTAGGCGTTTTGCGCTGCTTCGCGATGTACGGGCCAGATATACGCGTTGACATTGATTATTGACTAGTTATTAATAGTAATCAATTACGGGGTCATTAGTTCATAGCCCATATATGGAGTTCCGCGTTACATAACTTACGGTAAATGGCCCGCCTGGCTGACCGCCCAACGACCCCCGCCCATTGACGTCAATAATGACGTATGTTCCCATAGTAACGCCAATAGGGACTTTCCATTGACGTCAATGGGTGGAGTATTTACGGTAAACTGCCCACTTGGCAGTACATCAAGTGTATCATATGCCAAGTACGCCCCCTATTGACGTCAATGACGGTAAATGGCCCGCCTGGCATTATGCCCAGTACATGACCTTATGGGACTTTCCTACTTGGCAGTACATCTACGTATTAGTCATCGCTATTACCATGGTGATGCGGTTTTGGCAGTACATCAATGGGCGTGGATAGCGGTTTGACTCACGGGGATTTCCAAGTCTCCACCCCATTGACGTCAATGGGAGTTTGTTTTGGCACCAAAATCAACGGGACTTTCCAAAATGTCGTAACAACTCCGCCCCATTGACGCAAATGGGCGGTAGGCGTGTACGGTGGGAGGTCTATATAAGCAGAGCTCTCTGGCTAACTAGAGAACCCACTGCTTACTGGCTTATCGAAATTAATACGACTCACTATAGGGAGACCCAAGCTGGCTAGTTAAGCTTGGTACCGAGCTCGGATCCGCCACCatgggctgcattaaaagtaaagaaaacaaaagtccagccattaaatacagacctgaaaatactccagagcctgtcagtacaagtgtgagccattatggagcagaacccactacagtgtcaccatgtccgtcatcttcagcaaagggaacagcagttaatttcagcagtctttccatgacaccatttggaggatcctcaggggtaacgccttttggaggtgcatcttcctcattttcagtggtgccaagttcatatcctgctggtttaacaggtggtgttactatatttgtggccttatatgattatgaagctagaactacagaagacctttcatttaagaagggtgaaagatttcaaataattaacaatacggaaggagattggtgggaagcaagatcaatcgctacaggaaagaatggttatatcccgagcaattatgtagcgcctgcagattccattcaggcagaagaatggtattttggcaaaatggggagaaaagatgctgaaagattacttttgaatcctggaaatcaacgaggtattttcttagtaagagagagtgaaacaactaaaggtgcttattccctttctattcgtgattgggatgagataaggggtgacaatgtgaaacactacaaaattaggaaacttgacaatggtggatactatatcacaaccagagcacaatttgatactctgcagaaattggtgaaacactacacagaacatgctgatggtttatgccacaagttgacaactgtgtgtccaactgtgaaacctcagactcaaggtctagcaaaagatgcttgggaaatccctcgagaatctttgcgactagaggttaaactaggacaaggatgtttcggcgaagtgtggatgggaacatggaatggaaccacgaaagtagcaatcaaaacactaaaaccaggtacaatgatgccagaagctttccttcaagaagctcagataatgaaaaaattaagacatgataaacttgttccactatatgctgttgtttctgaagaaccaatttacattgtcactgaatttatgtcaaaaggaagcttattagatttccttaaggaaggagatggaaagtatttgaagcttccacagctggttgatatggctgctcagattgctgatggtatggcatatattgaaagaatgaactatattcaccgagatcttcgggctgctaatattcttgtaggagaaaatcttgtgtgcaaaatagcagactttggtttagcaaggttaattgaagacaatgaatacacagcaagacaaggtgcaaaatttccaatcaaatggacagctcctgaagctgcactgtatggtcggtttacaataaagtctgatgtctggtcatttggaattctgcaaacagaactagtaacaaagggccgagtgccatatccaggtatggtgaaccgtgaagtaCtagaacaagtggagcgaggatacaggatgccgtgccctcagggctgtccagaatccctccatgaattgatgaatctgtgttggaagaaggaccctgatgaaagaccaacatttgaatatattcagtccttcttggaagactacttcactgctacagagccacagtaccagccaggagaaaatttaCTCGAGTCTAGAGGGCCCTTCGACTACAAAGACCATGACGGTGATTATAAAGATCATGACATCGACTACAAGGATGACGATGACAAGTGAGTTTAAACCCGCTGATCAGCCTCGACTGTGCCTTCTAGTTGCCAGCCATCTGTTGTTTGCCCCTCCCCCGTGCCTTCCTTGACCCTGGAAGGTGCCACTCCCACTGTCCTTTCCTAATAAAATGAGGAAATTGCATCGCATTGTCTGAGTAGGTGTCATTCTATTCTGGGGGGTGGGGTGGGGCAGGACAGCAAGGGGGAGGATTGGGAAGACAATAGCAGGCATGCTGG  GGATGCGGTGGGCTCTATGGCTTCTGAGGCGGAAAGAACCAGCTGGGGCTCTAGGGGGTATCCCCACGCGCCCTGTAGCGGCGCATTAAGCGCGGCGGGTGTGGTGGTTACGCGCAGCGTGACCGCTACACTTGCCAGCGCCCTAGCGCCCGCTCCTTTCGCTTTCTTCCCTTCCTTTCTCGCCACGTTCGCCGGCTTTCCCCGTCAAGCTCTAAATCGGGGGCTCCCTTTAGGGTTCCGATTTAGTGCTTTACGGCACCTCGACCCCAAAAAACTTGATTAGGGTGATGGTTCACGTAGTGGGCCATCGCCCTGATAGACGGTTTTTCGCCCTTTGACGTTGGAGTCCACGTTCTTTAATAGTGGACTCTTGTTCCAAACTGGAACAACACTCAACCCTATCTCGGTCTATTCTTTTGATTTATAAGGGATTTTGCCGATTTCGGCCTATTGGTTAAAAAATGAGCTGATTTAACAAAAATTTAACGCGAATTAATTCTGTGGAATGTGTGTCAGTTAGGGTGTGGAAAGTCCCCAGGCTCCCCAGCAGGCAGAAGTATGCAAAGCATGCATCTCAATTAGTCAGCAACCAGGTGTGGAAAGTCCCCAGGCTCCCCAGCAGGCAGAAGTATGCAAAGCATGCATCTCAATTAGTCAGCAACCATAGTCCCGCCCCTAACTCCGCCCATCCCGCCCCTAACTCCGCCCAGTTCCGCCCATTCTCCGCCCCATGGCTGACTAATTTTTTTTATTTATGCAGAGGCCGAGGCCGCCTCTGCCTCTGAGCTATTCCAGAAGTAGTGAGGAGGCTTTTTTGGAGGCCTAGGCTTTTGCAAAAAGCTCCCGGGAGCTTGTATATCCATTTTCGGATCTGATCAAGAGACAGGATGAGGATCGTTTCGCATGATTGAACAAGATGGATTGCACGCAGGTTCTCCGGCCGCTTGGGTGGAGAGGCTATTCGGCTATGACTGGGCACAACAGACAATCGGCTGCTCTGATGCCGCCGTGTTCCGGCTGTCAGCGCAGGGGCGCCCGGTTCTTTTTGTCAAGACCGACCTGTCCGGTGCCCTGAATGAACTGCAGGACGAGGCAGCGCGGCTATCGTGGCTGGCCACGACGGGCGTTCCTTGCGCAGCTGTGCTCGACGTTGTCACTGAAGCGGGAAGGGACTGGCTGCTATTGGGCGAAGTGCCGGGGCAGGATCTCCTGTCATCTCACCTTGCTCCTGCCGAGAAAGTATCCATCATGGCTGATGCAATGCGGCGGCTGCATACGCTTGATCCGGCTACCTGCCCATTCGACCACCAAGCGAAACATCGCATCGAGCGAGCACGTACTCGGATGGAAGCCGGTCTTGTCGATCAGGATGATCTGGACGAAGAGCATCAGGGGCTCGCGCCAGCCGAACTGTTCGCCAGGCTCAAGGCGCGCATGCCCGACGGCGAGGATCTCGTCGTGACCCATGGCGATGCCTGCTTGCCGAATATCATGGTGGAAAATGGCCGCTTTTCTGGATTCATCGACTGTGGCCGGCTGGGTGTGGCGGACCGCTATCAGGACATAGCGTTGGCTACCCGTGATATTGCTGAAGAGCTTGGCGGCGAATGGGCTGACCGCTTCCTCGTGCTTTACGGTATCGCCGCTCCCGATTCGCAGCGCATCGCCTTCTATCGCCTT  CTTGACGAGTTCTTCTGAGCGGGACTCTGGGGTTCGCGAAATGACCGACCAAGCGACGCCCAACCTGCCATCACGAGATTTCGATTCCACCGCCGCCTTCTATGAAAGGTTGGGCTTCGGAATCGTTTTCCGGGACGCCGGCTGGATGATCCTCCAGCGCGGGGATCTCATGCTGGAGTTCTTCGCCCACCCCAACTTGTTTATTGCAGCTTATAATGGTTACAAATAAAGCAATAGCATCACAAATTTCACAAATAAAGCATTTTTTTCACTGCATTCTAGTTGTGGTTTGTCCAAACTCATCAATGTATCTTATCATGTCTGTATACCGTCGACCTCTAGCTAGAGCTTGGCGTAATCATGGTCATAGCTGTTTCCTGTGTGAAATTGTTATCCGCTCACAATTCCACACAACATACGAGCCGGAAGCATAAAGTGTAAAGCCTGGGGTGCCTAATGAGTGAGCTAACTCACATTAATTGCGTTGCGCTCACTGCCCGCTTTCCAGTCGGGAAACCTGTCGTGCCAGCTGCATTAATGAATCGGCCAACGCGCGGGGAGAGGCGGTTTGCGTATTGGGCGCTCTTCCGCTTCCTCGCTCACTGACTCGCTGCGCTCGGTCGTTCGGCTGCGGCGAGCGGTATCAGCTCACTCAAAGGCGGTAATACGGTTATCCACAGAATCAGGGGATAACGCAGGAAAGAACATGTGAGCAAAAGGCCAGCAAAAGGCCAGGAACCGTAAAAAGGCCGCGTTGCTGGCGTTTTTCCATAGGCTCCGCCCCCCTGACGAGCATCACAAAAATCGACGCTCAAGTCAGAGGTGGCGAAACCCGACAGGACTATAAAGATACCAGGCGTTTCCCCCTGGAAGCTCCCTCGTGCGCTCTCCTGTTCCGACCCTGCCGCTTACCGGATACCTGTCCGCCTTTCTCCCTTCGGGAAGCGTGGCGCTTTCTCATAGCTCACGCTGTAGGTATCTCAGTTCGGTGTAGGTCGTTCGCTCCAAGCTGGGCTGTGTGCACGAACCCCCCGTTCAGCCCGACCGCTGCGCCTTATCCGGTAACTATCGTCTTGAGTCCAACCCGGTAAGACACGACTTATCGCCACTGGCAGCAGCCACTGGTAACAGGATTAGCAGAGCGAGGTATGTAGGCGGTGCTACAGAGTTCTTGAAGTGGTGGCCTAACTACGGCTACACTAGAAGAACAGTATTTGGTATCTGCGCTCTGCTGAAGCCAGTTACCTTCGGAAAAAGAGTTGGTAGCTCTTGATCCGGCAAACAAACCACCGCTGGTAGCGGTGGTTTTTTTGTTTGCAAGCAGCAGATTACGCGCAGAAAAAAAGGATCTCAAGAAGATCCTTTGATCTTTTCTACGGGGTCTGACGCTCAGTGGAACGAAAACTCACGTTAAGGGATTTTGGTCATGAGATTATCAAAAAGGATCTTCACCTAGATCCTTTTAAATTAAAAATGAAGTTTTAAATCAATCTAAAGTATATATGAGTAAACTTGGTCTGACAGTTACCAATGCTTAATCAGTGAGGCACCTATCTCAGCGATCTGTCTATTTCGTTCATCCATAGTTGCCTGACTCCCCGTCGTGTAGATAACTACGATACGGGAGGGCTTACCATCTGGCCCCAGTGCTGCAATGATACCGCGAGACCCACGCTCACCGGCTCCAGATTTATCAGCAATAAACCAGCCAGCCGGAAGGGCCGAGCGCAGAAGTGGTCCTGCAACTTTATCCGCCTCCATCCAGTCTATTAATTGTTGCCGGGAAGCTAGAGTAAGTAGTTCGCCAGTTAATAGTTTGCGCAACGTTGTTGCCATTGCTACAGGCATCGTGGTGTCACGCTCGTCGTTTGGTATGGCTTCATTCAGCTCCGGTTCCCAACGATCAAGGCGAGTTACATGATCCCCCATGTTGTGCAAAAAAGCGGTTAGCTCCTTCGGTCCTCCGATCGTTGTCAGAAGTAAGTTGGCCGCAGTGTTATCACTCATGGTTATGGCAGCACTGCATAATTCTCTTACTGTCATGCCATCCGTAAGATGCTTTTCTGTGACTGGTGAGTACTCAACCAAGTCATTCTGAGAATAGTGTATGCGGCGACCGAGTTGCTCTTGCCCGGCGTCAATACGGGATAATACCGCGCCACATAGCAGAACTTTAAAAGTGCTCATCATTGGAAAACGTTCTTCGGGGCGAAAACTCTCAAGGATCTTACCGCTGTTGAGATCCAGTTCGATGTAACCCACTCGTGCACCCAACTGATCTTCAGCATCTTTTACTTTCACCAGCGTTTCTGGGTGAGCAAAAACAGGAAGGCAAAATGCCGCAAAAAAGGGAATAAGGGCGACACGGAAATGTTGAATACTCATACTCTTCCTTTTTCAATATTATTGAAGCATTTATCAGGGTTATTGTCTCATGAGCGGATACATATTTGAATGTATTTAGAAAAATAAACAAATAGGGGTTCCGCGCACATTTCCCCGAAAAGTGCCACCTGACGTC |
