# Supplementary material for: RBM15 promotes hepatocellular carcinoma progression by regulating N6-methyladenosine modification of YES1 mRNA in an IGF2BP1-dependent manner
Source: Cell Death Discov. 2021 Oct 27;7:315. doi: 10.1038/s41420-021-00703-w (PMC8551180; doi:10.1038/s41420-021-00703-w)
Supplement: Supplementary file 13 — supplementary table 4 [file 41420_2021_703_MOESM13_ESM.docx]

Supplementary Table 4 the inserted sequences employed in luciferase reporter assay

| Factor | Sequences |
| --- | --- |
| YES1-3UTR-WT: | TTCAAGTAGCCTATTTTATATGCACAAATCTGCCAAAATATAAAGAACTTGTGTA  GATTTTCTACAGGAATCAAAAGAAGAAAATCTTCTTTACTCTGCATGTTTTTAAT  GGTAAACTGGAATCCCAGATATGGTTGCACAAAACCACTTTTTTTTCCCCAAGT  ATTAAACTCTAATGTACCAATGATGAATTTATCAGCGTATTTCAGGGTCCAAACA  AAATAGAGCTAAGATACTGATGACAGTGTGGGTGACAGCATGGTAATGAAGGAC  AGTGAGGCTCCTGCTTATTTATAAATCATTTCCTTTCTTTTTTTCCCCAAAGTCAG  AATTGCTCAAAGAAAATTATTTATTGTTACAGATAAAACTTGAGAGATAAAAAGC  TATACCATAATAAAATCTAAAATTAAGGAATATCATGGGACCAAATAATTCCATTCC  AGTTTTTTAAAGTTTCTTGCATTTATTATTCTCAAAAGTTTTTTCTAAGTTAAACAG  TCAGTATGCAATCTTAATATATGCTTTCTTTTGCATGGACATGGGCCAGGTTTTTCA  AAAGGAATATAAACAGGATCTCAAACTTGATTAAATGTTAGACCACAGAAGTGGA  ATTTGAAAGTATAATGCAGTACATTAATATTCATGTTCATGGAACTGAAAGAATAAG  AACTTTTTCACTTCAGTCCTTTTCTGAAGAGTTTGACTTAGAATAATGAAGGTAAC  TAGAAAGTGAGTTAATCTTGTATGAGGTTGCATTGATTTTTTAAGGCAATATATAAT  TGAAACTACTGTCCAATCAA |
| YES1-3UTR-Mut | TTCAAGTAGCCTATTTTATATGCACAAATCTGCCAAAATATAAAGACCTTGTGTAGATTTT  CTACAGGAATCAAAAGAAGAAAATCTTCTTTACTCTGCATGTTTTTAATGGTAACCTGGA  ATCCCAGATATGGTTGCACAAAACCACTTTTTTTTCCCCAAGTATTAAACTCTAATGTAC  CAATGATGAATTTATCAGCGTATTTCAGGGTCCAAACAAAATAGAGCTAAGATACTGAT  GACAGTGTGGGTGACAGCATGGTAATGAAGGACAGTGAGGCTCCTGCTTATTTATAAA  TCATTTCCTTTCTTTTTTTCCCCAAAGTCAGAATTGCTCAAAGAAAATTATTTATTGTTA  CAGATAAAACTTGAGAGATAAAAAGCTATACCATAATAAAATCTAAAATTAAGGAATAT  CATGGGACCAAATAATTCCATTCCAGTTTTTTAAAGTTTCTTGCATTTATTATTCTCAAA  AGTTTTTTCTAAGTTAAACAGTCAGTATGCAATCTTAATATATGCTTTCTTTTGCATGGA  CATGGGCCAGGTTTTTCAAAAGGAATATAAACAGGATCTCAAACTTGATTAAATGTTAG  ACCACAGAAGTGGAATTTGAAAGTATAATGCAGTACATTAATATTCATGTTCATGGAAC  TGAAAGAATAAGAACTTTTTCACTTCAGTCCTTTTCTGAAGAGTTTGACTTAGAATAAT  GAAGGTAACTAGAAAGTGAGTTAATCTTGTATGAGGTTGCATTGATTTTTTAAGGCAAT  ATATAATTGAAACTACTGTCCAATCAA |
