# Supplementary material for: RBM15 promotes hepatocellular carcinoma progression by regulating N6-methyladenosine modification of YES1 mRNA in an IGF2BP1-dependent manner
Source: Cell Death Discov. 2021 Oct 27;7:315. doi: 10.1038/s41420-021-00703-w (PMC8551180; doi:10.1038/s41420-021-00703-w)
Supplement: Supplementary file 14 — supplementary table 5 [file 41420_2021_703_MOESM14_ESM.docx]

Supplementary Table 5. Univariate and multivariate Cox analysis of Cohort-1 for overall survival and disease-free survival

| Factors | Overall Survival | | | | | | Disease Free Survival | | | | | |
| --- | --- | --- | --- | --- | --- | --- | --- | --- | --- | --- | --- | --- |
|  | Univariate analysis | | | Multivariate analysis | | | Univariate analysis | | | Multivariate analysis | | |
|  | HR | 95%CI | *P* | HR | 95%CI | *P* | HR | 95%CI | *P* | HR | 95%CI | *P* |
| Age |  |  | 0.506 |  |  |  |  |  | 0.675 |  |  |  |
| ≤60 | 1 |  |  |  |  |  | 1 |  |  |  |  |  |
| ＞60 | 0.80 | 0.42-1.54 |  |  |  |  | 0.83 | 0.35-1.97 |  |  |  |  |
| AJCC stage |  |  | 0.845 |  |  |  |  |  | **0.030** |  |  | 0.057 |
| Stage I-II | 1 |  |  |  |  |  | 1 |  |  | 1 |  |  |
| Stage III-IV | 1.06 | 0.62-1.80 |  |  |  |  | 2.38 | 1.09-5.20 |  | 2.15 | 0.98-4.76 |  |
| Tumor number |  |  |  |  |  |  |  |  |  |  |  |  |
| Single | 1 |  | 0.088 |  |  |  | 1 |  | 0.768 |  |  |  |
| Multiple | 1.61 | 0.93-2.79 |  |  |  |  | 1.12 | 0.52-2.42 |  |  |  |  |
| Tumor size |  |  | 0.396 |  |  |  |  |  | 0.564 |  |  |  |
| ≤5cm | 1 |  |  |  |  |  | 1 |  |  |  |  |  |
| ＞5cm | 1.31 | 0.70-2.46 |  |  |  |  | 0.80 | 0.37-1.72 |  |  |  |  |
| Tumor encapsulation |  |  | **0.011** |  |  | 0.302 |  |  | 0.418 |  |  |  |
| Intact | 1 |  |  | 1 |  |  | 1 |  |  |  |  |  |
| Broken | 2.06 | 1.18-3.01 |  | 1.38 | 0.75-2.54 |  | 1.36 | 0.65-2.84 |  |  |  |  |
| Microvascular infiltration |  |  | **0.000** |  |  | **0.000** |  |  | 0.610 |  |  |  |
| No | 1 |  |  | 1 |  |  | 1 |  |  |  |  |  |
| Yes | 4.92 | 2.43-9.93 |  | 4.80 | 2.25-10.252 |  | 0.73 | 0.22-2.47 |  |  |  |  |
| HbsAg |  |  | 0.995 |  |  |  |  |  |  |  |  |  |
| Negative | 1 |  |  |  |  |  | 1 |  |  |  |  |  |
| Positive | 1.00 | 0.36-2.81 |  |  |  |  | 1.24 | 0.29-5.27 |  |  |  |  |
| Cirrhosis |  |  | 0.372 |  |  |  |  |  | 0.741 |  |  |  |
| Yes | 1 |  |  |  |  |  | 1 |  |  |  |  |  |
| No | 1.39 | 0.68-2.84 |  |  |  |  | 1.16 | 0.47-2.85 |  |  |  |  |
| AFP |  |  | 0.503 |  |  |  |  |  | 0.105 |  |  |  |
| ≤400 μg/L | 1 |  |  |  |  |  | 1 |  |  |  |  |  |
| ＞400 μg/L | 1.21 | 0.70-2.09 |  |  |  |  | 1.83 | 0.88-3.81 |  |  |  |  |
| RBM15 |  |  | **0.018** |  |  | **0.014** |  |  | **0.001** |  |  | **0.002** |
| Low | 1 |  |  | 1 |  |  | 1 |  |  | 1 |  |  |
| High | 1.98 | 1.13-3.48 |  | 2.06 | 1.16-3.68 |  | 3.82 | 1.68-8.68 |  | 3.57 | 1.57-8.12 |  |
