# Supplementary material for: RBM15 promotes hepatocellular carcinoma progression by regulating N6-methyladenosine modification of YES1 mRNA in an IGF2BP1-dependent manner
Source: Cell Death Discov. 2021 Oct 27;7:315. doi: 10.1038/s41420-021-00703-w (PMC8551180; doi:10.1038/s41420-021-00703-w)
Supplement: Supplementary file 15 — supplementary table 6 [file 41420_2021_703_MOESM15_ESM.docx]

**Supplementary Table 6. Down-regulated DEGs associated with RBM15**

| Symbol | NC1_FPKM | NC2_FPKM | NC3_FPKM | KD1_FPKM | KD2_FPKM | KD3_FPKM | FDR | log2FC |
| --- | --- | --- | --- | --- | --- | --- | --- | --- |
| ARL1 | 28.74541 | 30.53362 | 24.81846 | 3.644433 | 3.095859 | 4.618634 | 3.5E-115 | -3.14422 |
| B4GALT5 | 36.47572 | 35.52025 | 35.42973 | 10.46131 | 10.88463 | 10.14649 | 3.5E-115 | -1.76694 |
| EPB41L4B | 22.87252 | 22.10918 | 23.13082 | 5.989935 | 6.127581 | 5.606162 | 2.8E-106 | -1.92874 |
| VTI1B | 27.17256 | 31.3709 | 23.74763 | 3.627992 | 4.102638 | 2.849195 | 3.7E-106 | -2.60436 |
| TBL1XR1 | 28.21743 | 31.05428 | 28.7971 | 6.760668 | 9.314571 | 8.136907 | 1.1E-80 | -2.0808 |
| KRT80 | 51.40853 | 49.62909 | 46.82152 | 8.444625 | 11.27863 | 9.860626 | 7.47E-78 | -2.26293 |
| YES1 | 24.9545 | 24.55897 | 24.47059 | 6.609574 | 7.710465 | 6.764911 | 9.84E-77 | -1.78789 |
| LPGAT1 | 10.66443 | 10.38001 | 10.27737 | 3.554056 | 3.477351 | 3.248721 | 1.68E-75 | -1.60904 |
| UBE2G2 | 20.78699 | 20.27532 | 18.58916 | 7.06915 | 7.005584 | 6.96706 | 1.04E-72 | -1.80197 |
| CMTM6 | 22.14637 | 23.23198 | 24.01542 | 5.595442 | 4.758319 | 6.005949 | 6.34E-68 | -2.03705 |
| STK17B | 9.500625 | 8.873977 | 9.19608 | 2.454562 | 2.802248 | 2.873529 | 5.74E-66 | -1.81326 |
| SH3BGRL2 | 7.069653 | 7.16356 | 6.792233 | 1.325508 | 1.650054 | 1.491665 | 3.4E-65 | -2.16969 |
| RPA1 | 37.23274 | 39.88624 | 38.9987 | 15.8671 | 15.6534 | 14.5586 | 6.18E-65 | -1.3482 |
| EFCAB14 | 14.85284 | 14.27577 | 13.98861 | 4.10724 | 4.620489 | 4.434974 | 1.36E-62 | -1.68379 |
| RDH10 | 27.97268 | 24.96361 | 28.13972 | 8.718064 | 7.695217 | 8.326754 | 1.53E-61 | -1.70255 |
| COPZ1 | 117.1477 | 115.6929 | 106.7922 | 64.25903 | 52.77477 | 46.04437 | 2.32E-61 | -1.32041 |
| UXS1 | 104.831 | 107.0737 | 108.4104 | 40.49685 | 44.31445 | 43.47657 | 2.71E-59 | -1.32643 |
| NCEH1 | 31.6104 | 29.46279 | 30.18974 | 8.774097 | 10.55039 | 9.498687 | 5.89E-59 | -1.66613 |
| LRRC58 | 12.83303 | 10.99749 | 11.19432 | 3.541142 | 3.421082 | 2.937582 | 4.34E-55 | -1.78874 |
| NIPAL3 | 5.946472 | 7.254726 | 6.510549 | 3.192789 | 3.484843 | 3.395693 | 4.89E-54 | -1.85395 |
| CGGBP1 | 21.90593 | 21.0152 | 20.34236 | 7.659192 | 7.019073 | 8.689735 | 1.57E-53 | -1.42425 |
| LIN7C | 10.10886 | 8.905188 | 9.792185 | 2.584715 | 2.323075 | 1.960693 | 1.95E-53 | -2.00736 |
| AIDA | 25.48758 | 24.77009 | 23.33848 | 4.962245 | 6.704221 | 6.065707 | 5.68E-53 | -1.99216 |
| TTL | 8.665182 | 8.330934 | 8.175505 | 3.599081 | 3.684361 | 3.453499 | 4.51E-52 | -1.23227 |
| AMMECR1 | 17.6363 | 15.08504 | 16.97448 | 4.707182 | 5.468175 | 5.166052 | 2.23E-51 | -1.6617 |
| TM7SF3 | 24.21615 | 23.70859 | 21.69289 | 6.320369 | 5.31951 | 5.886151 | 4.51E-48 | -2.02743 |
| MBTPS2 | 7.918523 | 8.124281 | 8.139624 | 2.656119 | 2.873389 | 2.632615 | 8.17E-48 | -1.5435 |
| NT5E | 30.58139 | 29.66998 | 32.42075 | 12.26692 | 13.15077 | 12.98989 | 1.55E-47 | -1.39473 |
| LARP4B | 19.23254 | 17.8592 | 16.82643 | 5.676357 | 6.227329 | 4.525928 | 3.51E-47 | -1.68806 |
| ZMPSTE24 | 23.70745 | 24.09095 | 25.50526 | 9.21589 | 7.622664 | 7.948209 | 3.56E-46 | -1.54292 |
| GPD2 | 13.24944 | 12.51312 | 12.58571 | 5.546091 | 5.726716 | 4.469391 | 8.4E-46 | -1.46687 |
| DNAJC22 | 24.31088 | 27.03758 | 25.0775 | 9.446737 | 6.598421 | 8.098181 | 1.22E-45 | -1.4651 |
| PBK | 22.74305 | 23.21787 | 24.63073 | 7.105157 | 6.832409 | 7.795319 | 2.4E-44 | -1.56518 |
| ASF1A | 21.72884 | 20.52573 | 21.3968 | 7.110408 | 6.453619 | 7.668694 | 4.14E-44 | -1.55801 |
| MRPL18 | 50.41539 | 48.88665 | 56.32006 | 16.81074 | 13.41591 | 14.53518 | 4.67E-44 | -1.75194 |
| PALLD | 78.2922 | 72.57678 | 69.55036 | 29.14315 | 32.87326 | 29.83082 | 5.38E-44 | -1.26629 |
| MCM8 | 11.63453 | 17.57978 | 11.53906 | 4.746821 | 4.381496 | 3.855104 | 3.14E-43 | -1.42783 |
| ATL3 | 17.23831 | 18.44042 | 18.89965 | 8.268048 | 8.725532 | 7.88878 | 6E-43 | -1.183 |
| RANBP9 | 14.68298 | 15.11321 | 13.93107 | 5.738005 | 5.625168 | 5.637755 | 1.53E-42 | -1.35186 |
| SOS1 | 32.28664 | 27.68707 | 26.3257 | 9.384988 | 16.40633 | 9.117814 | 1.95E-42 | -1.54481 |
| FNDC3A | 5.124842 | 4.857385 | 4.522923 | 1.961323 | 1.357867 | 0.966089 | 3.75E-42 | -1.89334 |
| PPAT | 16.53039 | 14.84487 | 15.46475 | 6.710206 | 7.244393 | 5.63161 | 4.89E-42 | -1.36815 |
| MYL12A | 195.6394 | 210.6262 | 233.1606 | 66.26916 | 78.53112 | 76.48437 | 2.15E-41 | -1.51154 |
| PTBP3 | 60.6395 | 54.49788 | 59.57019 | 11.71421 | 21.42881 | 17.28635 | 5.22E-41 | -1.65078 |
| SCML1 | 8.031746 | 7.146125 | 8.068526 | 1.327005 | 1.613765 | 0.943644 | 7.07E-41 | -2.36998 |
| CHML | 17.04749 | 15.30676 | 17.02728 | 4.039838 | 6.045872 | 3.553244 | 7.1E-41 | -1.60454 |
| TMED7 | 16.11637 | 15.27174 | 15.19069 | 6.879817 | 6.633448 | 6.431461 | 3.37E-40 | -1.2217 |
| SURF6 | 13.80975 | 13.40221 | 13.37753 | 4.475162 | 5.345902 | 5.07243 | 6.46E-40 | -1.42818 |
| NUP43 | 9.074417 | 9.712737 | 9.941089 | 3.484334 | 3.48224 | 3.361411 | 6.6E-40 | -1.43059 |
| GTF2A2 | 30.69527 | 33.46264 | 31.98316 | 11.98004 | 10.79524 | 12.33501 | 1.16E-39 | -1.41196 |
| SLC12A7 | 36.58762 | 34.80914 | 34.64353 | 16.86096 | 16.74454 | 16.33723 | 3.74E-38 | -1.11371 |
| RCOR1 | 11.8228 | 11.18577 | 11.41897 | 4.773748 | 5.122908 | 4.418197 | 7.24E-38 | -1.26018 |
| CACYBP | 82.00807 | 87.21892 | 77.50968 | 32.54011 | 27.95286 | 32.47719 | 1.1E-37 | -1.32186 |
| TMED10 | 26.44424 | 26.07769 | 26.048 | 13.51605 | 13.34831 | 13.27592 | 5.18E-36 | -0.96777 |
| APPBP2 | 4.635037 | 5.000998 | 4.578528 | 1.76365 | 1.85054 | 1.690503 | 6.88E-35 | -1.39919 |
| WDR12 | 5.806025 | 6.794574 | 5.776958 | 2.216131 | 2.297171 | 2.298622 | 9.61E-35 | -1.40913 |
| Homo_sapiens_newGene_5798 | 2.742919 | 2.783887 | 2.684989 | 0.05271 | 0.06483 | 0.049082 | 1.33E-34 | -3.62083 |
| SLC7A1 | 7.465758 | 6.903471 | 6.042484 | 2.064974 | 2.458197 | 2.026174 | 1.7E-34 | -1.60089 |
| ACAA1 | 29.33656 | 30.40313 | 27.24112 | 12.2805 | 12.79827 | 12.73597 | 3.49E-34 | -1.20026 |
| SMAD2 | 10.10417 | 10.50718 | 9.627896 | 3.840884 | 2.910047 | 2.161134 | 3.99E-34 | -1.48117 |
| STARD3NL | 38.65163 | 43.25494 | 39.07102 | 15.66163 | 15.35146 | 15.89301 | 6.32E-34 | -1.27551 |
| C4orf46 | 8.968511 | 10.28763 | 10.0518 | 3.79377 | 3.454438 | 3.541422 | 1.3E-33 | -1.41267 |
| TTC5 | 3.719056 | 3.221918 | 4.29573 | 0.873836 | 0.952433 | 0.946263 | 1.97E-33 | -1.82948 |
| MRPL3 | 82.71386 | 90.13824 | 92.93935 | 20.52705 | 29.41912 | 26.40518 | 2.72E-33 | -1.75989 |
| PANK3 | 6.729047 | 6.244533 | 5.750834 | 1.961233 | 1.954035 | 1.342043 | 3.25E-33 | -1.77465 |
| NCOA7 | 7.772473 | 6.871818 | 6.811866 | 3.053184 | 2.162007 | 1.485315 | 7.55E-33 | -1.6357 |
| TMEM64 | 15.4949 | 20.03576 | 15.27968 | 5.414983 | 5.509087 | 5.500961 | 2.72E-31 | -1.56857 |
| CYP4F11 | 49.46324 | 47.77955 | 51.04465 | 24.56561 | 26.72605 | 24.81564 | 3.17E-31 | -0.95367 |
| SLC25A32 | 30.2669 | 29.43946 | 27.90813 | 10.49195 | 14.70656 | 12.81231 | 5.47E-31 | -1.32115 |
| RBM24 | 6.593407 | 6.348631 | 6.546616 | 1.894361 | 1.998407 | 2.14283 | 6.44E-31 | -1.6355 |
| PPP3R1 | 29.42695 | 27.29655 | 27.55698 | 12.28576 | 12.7581 | 13.67312 | 9.57E-31 | -1.11786 |
| SRGN | 55.80234 | 56.15007 | 61.30757 | 21.26296 | 21.80149 | 25.23936 | 1.24E-30 | -1.32213 |
| SEMA3C | 18.34705 | 16.60185 | 17.31725 | 5.880544 | 6.696572 | 5.512563 | 4.03E-30 | -1.34926 |
| CDCA4 | 26.79758 | 27.18217 | 27.32498 | 10.42112 | 10.92754 | 12.38699 | 6.91E-30 | -1.2441 |
| OSTM1 | 8.989867 | 8.851957 | 9.541959 | 4.065916 | 3.748229 | 4.058576 | 8.24E-30 | -1.19534 |
| SUN1 | 49.75098 | 53.71259 | 45.38528 | 20.35826 | 25.3843 | 23.26214 | 1.2E-29 | -1.21032 |
| TCEAL1 | 22.11175 | 17.2901 | 17.24595 | 7.70697 | 6.961827 | 6.337223 | 6.95E-29 | -1.42974 |
| ARSJ | 7.348838 | 5.964859 | 6.523873 | 2.144513 | 2.71178 | 2.470695 | 6.98E-29 | -1.34894 |
| TMEM167A | 14.24825 | 13.66037 | 14.09811 | 6.998728 | 6.793293 | 7.189332 | 7.03E-29 | -1.00393 |
| MTMR4 | 5.731653 | 4.894519 | 5.503007 | 1.814869 | 2.3516 | 2.264308 | 8.39E-29 | -1.43407 |
| SHC1 | 97.12461 | 93.449 | 93.09438 | 45.20124 | 50.37817 | 49.78532 | 8.69E-29 | -0.96825 |
| IPO5 | 27.9596 | 28.47363 | 26.69692 | 14.10638 | 14.38398 | 13.41899 | 9.26E-29 | -1.00361 |
| DYNLT3 | 10.4342 | 10.10166 | 10.94862 | 4.210216 | 4.196458 | 4.144729 | 3.32E-28 | -1.34973 |
| KPNA3 | 10.27929 | 9.922277 | 9.348353 | 4.544899 | 4.589352 | 3.973187 | 3.86E-28 | -1.17499 |
| CENPK | 13.22847 | 12.93681 | 12.24721 | 3.132801 | 4.308562 | 3.244181 | 4.64E-28 | -1.7839 |
| GABARAPL1 | 13.41808 | 14.52679 | 14.08764 | 5.793927 | 5.609308 | 6.471405 | 5.57E-28 | -1.40614 |
| ACER3 | 3.095125 | 3.188405 | 3.042457 | 1.403768 | 1.295152 | 1.330003 | 7.14E-28 | -1.34477 |
| PGM2L1 | 1.449122 | 1.328896 | 1.333997 | 0.359239 | 0.402301 | 0.31839 | 9.34E-28 | -1.81856 |
| KDM1B | 5.091975 | 5.305512 | 5.696611 | 1.218872 | 1.500253 | 1.266553 | 1.1E-27 | -1.91829 |
| BIRC3 | 8.9765 | 8.21479 | 7.650458 | 3.381236 | 2.785023 | 2.58549 | 1.28E-27 | -1.45502 |
| RAB18 | 13.19586 | 14.13112 | 13.21509 | 6.624243 | 6.725588 | 6.835261 | 1.97E-27 | -1.00172 |
| CLCN4 | 1.643039 | 1.654243 | 1.79818 | 0.270899 | 0.4082 | 0.391356 | 2.03E-27 | -2.06716 |
| MTX3 | 3.591093 | 3.172221 | 3.249399 | 1.768451 | 1.783899 | 1.190344 | 2.23E-27 | -1.33964 |
| ASAP2 | 7.576759 | 6.907536 | 6.578509 | 2.225885 | 2.857077 | 2.336651 | 3.22E-27 | -1.46804 |
| CBFB | 20.41524 | 20.35312 | 18.85222 | 6.890871 | 7.73454 | 5.471429 | 5.46E-27 | -1.48557 |
| SLC11A2 | 14.8244 | 15.25956 | 16.38023 | 9.914783 | 7.80862 | 8.190593 | 5.82E-27 | -1.03795 |
| TNFRSF10A | 16.94606 | 14.52407 | 14.41618 | 5.272395 | 6.285357 | 5.085163 | 7.47E-27 | -1.42607 |
| IMPAD1 | 22.51526 | 20.65914 | 20.43617 | 10.25817 | 11.39743 | 9.592266 | 8.99E-27 | -1.04832 |
| SLC20A1 | 55.8212 | 55.07697 | 54.96985 | 26.87999 | 30.34899 | 29.25604 | 1.09E-26 | -0.96649 |
| RAB3B | 7.284594 | 6.492922 | 6.418174 | 2.728925 | 2.768727 | 2.102386 | 1.36E-26 | -1.37856 |
| SOWAHC | 11.66386 | 10.6426 | 10.98314 | 5.109266 | 5.291384 | 4.482429 | 1.64E-26 | -1.15281 |
| PTAR1 | 9.108173 | 7.624466 | 7.203566 | 3.483466 | 3.665979 | 2.645468 | 1.95E-26 | -1.50601 |
| NSUN2 | 51.37794 | 49.24675 | 42.11924 | 22.89124 | 21.47856 | 21.45992 | 2.09E-26 | -1.12781 |
| TAP2 | 8.814885 | 10.11751 | 10.47754 | 3.901484 | 4.404969 | 3.984285 | 2.27E-26 | -1.23753 |
| CNOT11 | 26.12259 | 27.8333 | 28.16505 | 15.0183 | 13.74295 | 13.89682 | 2.71E-26 | -0.95636 |
| MAPK6 | 16.57918 | 15.23276 | 16.12069 | 6.584673 | 7.957207 | 6.922993 | 3.76E-26 | -1.14934 |
| ANKMY2 | 18.18636 | 16.64084 | 16.22232 | 7.816237 | 7.953649 | 7.490295 | 7.98E-26 | -1.11684 |
| PLAGL2 | 8.303217 | 7.591846 | 7.420292 | 3.508458 | 3.456738 | 2.94041 | 8.52E-26 | -1.21892 |
| STARD4 | 3.589239 | 2.979835 | 3.068562 | 1.107762 | 1.11394 | 1.075178 | 8.57E-26 | -1.75611 |
| ZNF41 | 2.742916 | 2.202939 | 2.254151 | 1.535485 | 1.015293 | 0.7685 | 1.6E-25 | -2.39096 |
| MSMO1 | 43.53156 | 43.98046 | 38.38704 | 18.03536 | 19.423 | 18.73788 | 2.57E-25 | -1.17323 |
| GFM1 | 38.90868 | 39.58482 | 35.03025 | 17.95841 | 20.1794 | 18.07922 | 5.23E-25 | -0.96913 |
| WSB2 | 93.30106 | 93.42344 | 92.7495 | 54.04521 | 54.21297 | 55.0107 | 1.43E-24 | -0.83213 |
| TPMT | 8.385954 | 8.67761 | 7.658602 | 3.309901 | 3.59033 | 3.07761 | 1.64E-24 | -1.28259 |
| KIF21A | 22.43393 | 21.97004 | 18.93271 | 9.444958 | 10.28451 | 9.583585 | 2.6E-24 | -1.12308 |
| INSIG1 | 13.26528 | 11.65511 | 13.39876 | 4.782569 | 5.991433 | 5.656058 | 2.91E-24 | -1.2622 |
| IMPA1 | 16.84089 | 17.93502 | 17.0366 | 8.359181 | 9.349678 | 7.762737 | 3.97E-24 | -1.13871 |
| NAA20 | 113.1942 | 118.4719 | 119.4946 | 73.59705 | 62.01468 | 67.05653 | 5.67E-24 | -0.85238 |
| GABPA | 4.36247 | 3.969309 | 4.121811 | 1.549622 | 1.769725 | 1.437508 | 6.09E-24 | -1.34322 |
| SPIN4 | 4.943042 | 5.215259 | 4.169314 | 1.483756 | 1.691591 | 1.635987 | 7.42E-24 | -1.51285 |
| G3BP1 | 59.81687 | 42.79146 | 26.5865 | 31.49876 | 18.59114 | 18.28229 | 1.14E-23 | -0.93283 |
| SPRED1 | 11.82176 | 10.05951 | 10.62419 | 5.051626 | 5.355017 | 4.274687 | 1.51E-23 | -1.15663 |
| IL1RAP | 7.838084 | 7.712685 | 6.872734 | 2.590118 | 3.4911 | 2.977892 | 2.11E-23 | -1.37833 |
| CDC27 | 23.17664 | 19.82941 | 19.15695 | 12.0795 | 13.47602 | 10.59601 | 2.21E-23 | -0.94777 |
| CCND1 | 139.1134 | 136.2727 | 138.0125 | 69.15014 | 79.29709 | 76.2469 | 2.88E-23 | -0.88449 |
| NDFIP2 | 4.752372 | 5.558014 | 4.801637 | 1.321543 | 1.438177 | 1.430486 | 2.91E-23 | -1.6517 |
| PRKACB | 11.19748 | 9.775894 | 10.81342 | 4.395522 | 4.879783 | 4.668973 | 3.04E-23 | -1.22992 |
| NCL | 278.387 | 289.7608 | 275.8538 | 144.8015 | 172.4992 | 163.2467 | 3.1E-23 | -0.88704 |
| BET1 | 24.36208 | 26.79758 | 21.02708 | 7.77715 | 6.868331 | 9.062654 | 4.35E-23 | -1.53088 |
| INPP5F | 4.714703 | 4.408676 | 3.810432 | 2.362571 | 2.024554 | 1.791155 | 4.35E-23 | -1.28561 |
| CDIPT | 26.87911 | 31.36225 | 23.46855 | 6.655445 | 4.412656 | 7.625311 | 4.77E-23 | -1.95254 |
| MTRR | 9.881795 | 9.486517 | 9.642146 | 4.722337 | 5.218885 | 4.955717 | 6.01E-23 | -1.1017 |
| BAG2 | 10.10868 | 10.34493 | 10.65415 | 5.598313 | 5.205192 | 5.66578 | 8.39E-23 | -0.9199 |
| UHMK1 | 24.10898 | 21.93385 | 20.66206 | 10.33561 | 11.17314 | 8.904852 | 1.19E-22 | -1.12184 |
| ADGRG6 | 3.664738 | 3.006914 | 3.101842 | 1.222898 | 1.079796 | 0.881604 | 2.1E-22 | -1.61039 |
| MFSD1 | 18.9713 | 26.57475 | 22.33842 | 8.254621 | 10.08968 | 9.010436 | 5.34E-22 | -1.41323 |
| RYK | 14.76074 | 16.95537 | 16.90864 | 7.341099 | 8.517071 | 6.805195 | 6.24E-22 | -1.13356 |
| SSR3 | 37.21162 | 44.6404 | 43.06923 | 18.10707 | 19.90166 | 21.83652 | 7.78E-22 | -1.01395 |
| RAPGEF2 | 11.48605 | 13.31991 | 10.75643 | 4.532859 | 5.706817 | 4.865592 | 1.1E-21 | -1.23916 |
| LOXL2 | 27.89292 | 25.58984 | 29.52561 | 12.59791 | 14.46004 | 14.03016 | 1.72E-21 | -1.03254 |
| FEM1C | 7.935014 | 7.961368 | 7.306895 | 2.619575 | 3.536672 | 2.649478 | 1.81E-21 | -1.35509 |
| CYP24A1 | 21.99578 | 20.30965 | 21.89847 | 12.02216 | 11.02736 | 11.17119 | 1.83E-21 | -0.90103 |
| SLC7A11 | 41.54996 | 36.24771 | 32.96609 | 14.80072 | 15.72407 | 10.91259 | 2.05E-21 | -1.3905 |
| SCML2 | 2.574931 | 2.630419 | 2.117033 | 0.63662 | 0.951523 | 0.784737 | 2.18E-21 | -1.75764 |
| NOC3L | 10.80086 | 9.711715 | 10.00207 | 5.151358 | 5.017905 | 4.744494 | 2.41E-21 | -1.02706 |
| TMEM123 | 119.6228 | 112.8223 | 114.6146 | 73.11109 | 72.84435 | 67.91801 | 3.81E-21 | -0.71416 |
| ATL2 | 26.27011 | 29.13751 | 23.48508 | 11.6993 | 10.83639 | 10.14523 | 3.91E-21 | -1.16567 |
| CPOX | 37.30756 | 36.60058 | 37.27827 | 22.85481 | 21.34954 | 20.62861 | 4.05E-21 | -0.7907 |
| SREBF2 | 18.78513 | 18.03993 | 17.7576 | 9.65824 | 10.38486 | 10.37111 | 4.45E-21 | -0.84388 |
| SGPL1 | 7.333767 | 6.444595 | 6.999157 | 3.106386 | 3.577725 | 3.432783 | 4.81E-21 | -1.13041 |
| NUP58 | 7.715537 | 8.040838 | 7.269484 | 2.317415 | 3.212612 | 2.764125 | 5.38E-21 | -1.36547 |
| KBTBD2 | 17.01362 | 16.12193 | 17.19275 | 8.915002 | 8.931198 | 7.547903 | 6.26E-21 | -0.99034 |
| FADS1 | 34.27963 | 35.258 | 36.30966 | 21.51343 | 21.60522 | 21.32516 | 7.53E-21 | -0.74787 |
| OXR1 | 12.12461 | 11.59068 | 15.57219 | 6.087603 | 6.8465 | 8.166373 | 8.29E-21 | -1.12167 |
| DDX3Y | 11.07223 | 9.844075 | 9.864278 | 4.752701 | 4.768159 | 4.565199 | 1.09E-20 | -1.13555 |
| CASD1 | 6.011863 | 5.546245 | 5.427166 | 1.599069 | 2.220747 | 1.926185 | 1.61E-20 | -1.54525 |
| NDP | 1.600342 | 2.209137 | 2.697861 | 0.118744 | 0.09664 | 0.205278 | 1.62E-20 | -2.77262 |
| THRAP3 | 25.65501 | 24.79688 | 24.79598 | 14.79021 | 15.46089 | 14.07742 | 1.75E-20 | -0.77122 |
| MFSD9 | 7.704881 | 8.531549 | 8.089143 | 4.775223 | 4.182095 | 3.928963 | 2.47E-20 | -1.12429 |
| PTP4A2 | 45.10903 | 48.41587 | 49.00073 | 26.00409 | 25.9025 | 27.67983 | 2.66E-20 | -0.84863 |
| MED1 | 15.66588 | 14.45025 | 13.60835 | 5.802045 | 6.548816 | 5.18337 | 2.66E-20 | -1.3382 |
| ARL4C | 89.87814 | 82.67106 | 89.53975 | 50.88504 | 53.69322 | 52.86658 | 3.85E-20 | -0.75287 |
| ARPP19 | 53.71417 | 50.72828 | 52.94299 | 37.92917 | 36.89229 | 34.96872 | 6.01E-20 | -0.71759 |
| CBX5 | 22.24585 | 20.16635 | 19.55238 | 10.91144 | 11.33261 | 9.506923 | 6.62E-20 | -1.02439 |
| FECH | 10.96567 | 12.68416 | 10.73422 | 5.359246 | 5.830276 | 5.277712 | 8.92E-20 | -1.0577 |
| LSM14B | 23.7199 | 22.83346 | 22.8761 | 12.90723 | 13.12476 | 12.74654 | 1.11E-19 | -0.81495 |
| STRN | 7.928499 | 7.117821 | 6.968051 | 3.481186 | 3.914494 | 3.268039 | 1.92E-19 | -1.02602 |
| NIPA1 | 5.569887 | 4.763236 | 4.647272 | 1.366564 | 1.091767 | 1.425392 | 2.87E-19 | -1.81114 |
| KBTBD11 | 2.411434 | 1.909177 | 2.244423 | 0.701707 | 0.777002 | 0.556155 | 3.45E-19 | -1.58495 |
| YWHAZ | 655.3565 | 635.5443 | 455.2493 | 431.0689 | 414.6036 | 397.5927 | 3.87E-19 | -0.74294 |
| AGFG1 | 70.55584 | 66.51491 | 59.44682 | 38.19145 | 29.81644 | 15.98317 | 4.29E-19 | -0.71384 |
| DEGS1 | 42.89526 | 44.75615 | 44.76272 | 25.76421 | 27.22587 | 24.62784 | 5.27E-19 | -0.77912 |
| TNPO1 | 44.15944 | 37.81254 | 37.9994 | 23.35851 | 25.55563 | 19.69523 | 5.81E-19 | -0.83618 |
| EXOC6B | 5.811896 | 5.028815 | 4.801325 | 1.684841 | 2.324273 | 1.915243 | 6.51E-19 | -1.32997 |
| MCL1 | 93.2441 | 86.46058 | 88.22351 | 49.39501 | 43.41928 | 40.63908 | 7.91E-19 | -0.95927 |
| DYNC1LI2 | 15.82476 | 15.22778 | 15.18177 | 9.052386 | 9.54248 | 8.555409 | 8.92E-19 | -0.81463 |
| CCNA2 | 26.83374 | 28.99108 | 28.39015 | 16.04403 | 16.37053 | 16.55548 | 9.91E-19 | -0.78935 |
| RPE | 22.16522 | 20.95941 | 20.20835 | 11.66675 | 10.90943 | 10.43862 | 1.06E-18 | -1.01866 |
| SPCS3 | 24.23807 | 24.51232 | 25.9616 | 13.68107 | 14.73635 | 14.75982 | 1.14E-18 | -0.79758 |
| CIPC | 2.265748 | 2.502096 | 2.060084 | 0.699914 | 0.495584 | 0.65394 | 1.28E-18 | -1.71312 |
| MTMR6 | 2.766779 | 2.83773 | 2.828365 | 1.049333 | 1.27452 | 1.120099 | 1.82E-18 | -1.25192 |
| SPOPL | 2.804689 | 3.438055 | 2.476344 | 1.241171 | 1.509421 | 1.255682 | 2.25E-18 | -1.41413 |
| CCDC6 | 14.56431 | 13.15987 | 13.03625 | 6.905591 | 7.544363 | 6.145671 | 3.06E-18 | -0.97889 |
| DUSP5 | 44.79121 | 41.96184 | 42.9837 | 21.36766 | 24.39772 | 24.60231 | 3.48E-18 | -0.88281 |
| HEATR3 | 8.537539 | 8.090407 | 8.657671 | 4.543245 | 4.447668 | 4.336493 | 4.99E-18 | -0.92249 |
| CHMP3 | 30.07757 | 31.48601 | 28.80084 | 16.24634 | 17.62192 | 17.55134 | 6.9E-18 | -0.8404 |
| GARS | 72.08261 | 75.52407 | 70.79706 | 45.51598 | 42.47635 | 44.8303 | 1.11E-17 | -0.65572 |
| ACSL4 | 26.65087 | 23.13815 | 23.91711 | 14.95645 | 13.81764 | 13.09186 | 1.24E-17 | -0.91073 |
| ATP6V1C1 | 36.99153 | 36.6022 | 36.91336 | 34.42245 | 30.45066 | 29.6539 | 1.7E-17 | -0.82442 |
| RBM15 | 9.025878 | 7.925065 | 6.814704 | 3.427954 | 2.838424 | 2.67792 | 2.16E-17 | -1.33601 |
| WEE1 | 9.163701 | 14.56589 | 13.75213 | 3.811411 | 3.990032 | 4.376202 | 2.91E-17 | -1.64656 |
| AGO2 | 16.35505 | 13.94398 | 13.94083 | 7.056006 | 8.507078 | 6.849019 | 3.2E-17 | -0.94604 |
| ACAT2 | 21.70306 | 22.02354 | 21.87402 | 12.5341 | 12.67861 | 13.46653 | 3.48E-17 | -0.76834 |
| HIF1A | 76.83529 | 70.47671 | 69.58522 | 36.58319 | 42.17984 | 32.97167 | 4.03E-17 | -0.95251 |
| TRIM44 | 10.83646 | 10.30052 | 9.668257 | 5.532959 | 6.2437 | 5.453698 | 4.25E-17 | -0.84113 |
| FAM136A | 55.73652 | 56.41453 | 57.3703 | 29.79557 | 31.40548 | 34.24843 | 4.73E-17 | -0.82863 |
| LNPEP | 3.726953 | 3.513608 | 3.300715 | 1.249776 | 1.619048 | 1.026977 | 5.09E-17 | -1.37031 |
| EI24 | 67.0332 | 73.93672 | 77.27892 | 44.81265 | 45.63562 | 46.06135 | 6.28E-17 | -0.72798 |
| FMNL2 | 10.89688 | 9.250035 | 8.716004 | 4.69085 | 4.80507 | 4.102136 | 7.03E-17 | -1.0679 |
| EEF2K | 7.449889 | 6.452866 | 6.665805 | 3.837879 | 4.109549 | 3.618809 | 7.24E-17 | -0.89767 |
| Homo_sapiens_newGene_16497 | 4.606889 | 4.413771 | 5.969942 | 1.647341 | 1.90668 | 1.744482 | 7.69E-17 | -1.39266 |
| CPEB4 | 2.396564 | 2.606552 | 2.312761 | 0.936398 | 1.652871 | 0.832327 | 7.69E-17 | -1.59315 |
| SLC5A3 | 3.555299 | 2.952891 | 3.024264 | 1.247619 | 1.207809 | 0.791129 | 8.05E-17 | -1.46609 |
| HMGCS1 | 19.82673 | 22.19371 | 21.64977 | 11.10676 | 12.47705 | 11.36339 | 1.03E-16 | -0.86326 |
| ZNF367 | 13.31506 | 13.9844 | 14.78643 | 7.273821 | 8.178779 | 7.218453 | 1.41E-16 | -0.89125 |
| SUCLA2 | 12.5955 | 12.12384 | 11.74449 | 5.375322 | 5.067763 | 6.009795 | 1.64E-16 | -1.23871 |
| RPL23A | 363.8268 | 388.2195 | 380.262 | 231.5468 | 213.1132 | 239.8081 | 1.8E-16 | -0.72203 |
| ARL6IP6 | 11.14733 | 10.44345 | 10.43118 | 5.05542 | 4.896258 | 5.757687 | 1.82E-16 | -1.02605 |
| RPAP1 | 7.3845 | 6.715033 | 8.226437 | 3.772675 | 4.205421 | 3.633325 | 1.95E-16 | -0.94312 |
| F3 | 23.37973 | 20.42126 | 21.58859 | 6.964597 | 9.847327 | 9.359399 | 1.97E-16 | -1.26652 |
| CMAS | 46.62217 | 47.5994 | 51.13067 | 25.78902 | 26.86149 | 28.77567 | 2E-16 | -0.84691 |
| RMI1 | 11.37784 | 9.376592 | 8.97182 | 4.512714 | 3.603985 | 4.043521 | 2.11E-16 | -1.28064 |
| UBA5 | 10.8663 | 11.52649 | 10.95958 | 4.788308 | 7.539801 | 5.649494 | 2.5E-16 | -1.00624 |
| GBP1 | 5.965701 | 5.596421 | 5.700931 | 2.675035 | 2.611281 | 2.831291 | 2.82E-16 | -1.06599 |
| YOD1 | 5.806047 | 5.655834 | 5.211443 | 2.777705 | 2.756347 | 2.162854 | 3.83E-16 | -1.09146 |
| ICK | 6.324473 | 6.055271 | 5.772827 | 3.079903 | 3.212217 | 2.53991 | 4.14E-16 | -1.02437 |
| EIF5A2 | 6.651932 | 6.205779 | 6.284606 | 3.439471 | 3.429292 | 3.348607 | 5.02E-16 | -0.88243 |
| IPO8 | 12.06407 | 12.07273 | 10.76796 | 6.668486 | 5.263856 | 5.013671 | 5.66E-16 | -1.03897 |
| 44082 | 7.478356 | 7.793283 | 7.019019 | 3.379082 | 3.631173 | 3.408889 | 7.32E-16 | -1.16328 |
| ENTPD7 | 7.332263 | 6.010925 | 6.540262 | 2.088161 | 2.943421 | 2.182554 | 8.44E-16 | -1.39518 |
| RAD51AP1 | 31.42594 | 23.38833 | 25.75236 | 13.22828 | 11.07605 | 14.01113 | 1.02E-15 | -0.97945 |
| ZDHHC21 | 0.631452 | 0.523198 | 0.490443 | 0.115248 | 0.130117 | 0.125988 | 1.17E-15 | -1.86217 |
| CNIH1 | 20.60832 | 21.51056 | 22.46148 | 18.40413 | 17.89039 | 14.04872 | 1.26E-15 | -0.76527 |
| SAMD5 | 8.678831 | 7.626638 | 8.155488 | 3.559248 | 4.192167 | 2.914772 | 1.58E-15 | -1.16252 |
| RDX | 37.65006 | 43.42393 | 42.58406 | 22.29322 | 24.81245 | 20.96097 | 2.38E-15 | -0.87478 |
| DLGAP5 | 10.61486 | 10.97102 | 10.26757 | 6.067578 | 5.75058 | 5.200274 | 2.63E-15 | -0.88492 |
| SORBS3 | 27.30915 | 26.54208 | 29.31085 | 14.29817 | 11.53609 | 14.32198 | 2.76E-15 | -0.97968 |
| BACH1 | 8.493102 | 9.03717 | 7.307593 | 4.426062 | 4.650349 | 3.658126 | 3.35E-15 | -1.03436 |
| WDR44 | 16.3697 | 14.8689 | 15.43604 | 9.057102 | 9.374413 | 8.154789 | 3.52E-15 | -0.80263 |
| CKAP2 | 9.12677 | 8.741675 | 9.671244 | 4.823429 | 4.256554 | 3.643732 | 3.71E-15 | -1.10599 |
| SQLE | 50.98898 | 47.91178 | 44.45987 | 23.76758 | 22.7918 | 27.90547 | 4E-15 | -0.93881 |
| GFOD1 | 2.862937 | 2.481991 | 2.903987 | 1.120257 | 1.213916 | 1.591291 | 5.25E-15 | -1.04811 |
| AEN | 37.10089 | 36.75949 | 34.90144 | 22.33849 | 23.46721 | 22.34596 | 5.35E-15 | -0.66253 |
| GNPNAT1 | 19.00389 | 20.18081 | 19.74256 | 10.55325 | 11.96621 | 11.37053 | 5.98E-15 | -0.752 |
| TMEM167B | 6.912259 | 7.039153 | 7.063794 | 3.774759 | 3.770813 | 3.147627 | 6.91E-15 | -0.96307 |
| ARMCX6 | 13.30957 | 15.34839 | 15.21913 | 7.654574 | 7.649048 | 7.283055 | 8.31E-15 | -0.94875 |
| ARNTL2 | 14.45347 | 14.68413 | 13.36949 | 6.589275 | 9.191108 | 7.866166 | 1.02E-14 | -0.96513 |
| GULP1 | 10.25835 | 11.00815 | 11.06925 | 5.060496 | 5.554861 | 5.455069 | 1.1E-14 | -0.9657 |
| IST1 | 30.52334 | 28.16011 | 23.73543 | 11.36104 | 11.61353 | 10.34614 | 1.12E-14 | -1.20657 |
| TRPM7 | 6.451991 | 6.301129 | 5.476909 | 3.006068 | 3.827423 | 2.910405 | 1.43E-14 | -0.93567 |
| PLSCR3 | 10.09458 | 9.821503 | 7.827215 | 4.654759 | 2.872148 | 3.382321 | 1.58E-14 | -1.36057 |
| CEP55 | 48.90282 | 50.01187 | 46.40598 | 25.66005 | 29.32371 | 29.54343 | 1.6E-14 | -0.7853 |
| STK38L | 10.3976 | 9.805267 | 9.947583 | 5.739444 | 6.232678 | 5.998526 | 1.76E-14 | -0.82189 |
| HLTF | 10.17434 | 10.43132 | 9.563752 | 5.198139 | 5.894264 | 5.564802 | 1.79E-14 | -0.88421 |
| PHTF2 | 24.89809 | 24.88267 | 21.79815 | 16.61914 | 15.77644 | 16.30132 | 2.15E-14 | -0.64873 |
| SAR1A | 24.53507 | 23.23443 | 24.26388 | 14.12154 | 13.42295 | 15.50966 | 2.51E-14 | -0.77878 |
| NCF2 | 2.659327 | 2.470366 | 2.257126 | 0.082403 | 0.213897 | 0.517841 | 2.64E-14 | -2.28945 |
| RADX | 6.882922 | 6.790021 | 6.377358 | 3.096515 | 3.64213 | 2.786542 | 2.92E-14 | -1.0728 |
| HK1 | 56.11864 | 52.92432 | 57.30827 | 33.854 | 37.37098 | 35.096 | 3.01E-14 | -0.66123 |
| TRAK2 | 5.135375 | 5.033579 | 5.331987 | 3.135163 | 2.989747 | 2.805375 | 3.08E-14 | -0.81915 |
| GLS | 24.47036 | 22.85467 | 22.95781 | 14.84188 | 15.85935 | 15.11057 | 3.92E-14 | -0.62487 |
| TRIP13 | 22.13151 | 21.91917 | 23.27708 | 11.54863 | 13.2258 | 13.34422 | 4.48E-14 | -0.81948 |
| INIP | 7.211404 | 9.517282 | 8.855678 | 4.194379 | 4.26897 | 4.087782 | 4.55E-14 | -0.87402 |
| MEST | 16.10456 | 16.04321 | 16.18165 | 9.827129 | 10.08613 | 9.373134 | 4.92E-14 | -0.73266 |
| PRKAG2 | 9.131658 | 9.316425 | 10.79351 | 4.72394 | 5.639905 | 5.834415 | 5E-14 | -0.95598 |
| TOMM20 | 67.20547 | 70.59664 | 70.43481 | 47.55357 | 44.8702 | 46.65873 | 5.77E-14 | -0.59544 |
| TMEM184C | 17.63875 | 16.93259 | 17.22371 | 11.35374 | 10.59925 | 9.813076 | 5.85E-14 | -0.71496 |
| MIGA1 | 6.713398 | 6.627362 | 5.250706 | 2.90705 | 3.60607 | 2.680474 | 7.15E-14 | -1.0635 |
| PRKAA1 | 11.41797 | 11.50502 | 10.78999 | 5.974282 | 6.751081 | 5.146386 | 7.25E-14 | -0.94067 |
| TMEM65 | 7.18335 | 6.725716 | 7.016698 | 4.422152 | 4.410115 | 3.973945 | 7.73E-14 | -0.71612 |
| NR1D2 | 16.77191 | 17.13018 | 15.50977 | 9.719325 | 10.71407 | 9.618622 | 7.81E-14 | -0.72988 |
| DENND6A | 7.417016 | 6.177359 | 6.32716 | 4.426109 | 4.148103 | 3.657603 | 8.58E-14 | -0.86062 |
| DNAJB1 | 65.06618 | 65.5985 | 67.3208 | 45.03189 | 44.42218 | 40.6795 | 8.6E-14 | -0.6166 |
| GTPBP2 | 7.119647 | 6.819847 | 7.090555 | 3.270126 | 3.181738 | 4.079252 | 1.04E-13 | -1.0432 |
| RAB2B | 3.09146 | 3.115138 | 2.570623 | 1.059878 | 0.865841 | 0.999733 | 1.09E-13 | -1.40435 |
| GTF2A1 | 8.254318 | 7.514576 | 8.195298 | 4.602631 | 5.044914 | 4.673191 | 1.16E-13 | -0.73887 |
| RUFY2 | 6.389568 | 5.752575 | 4.930779 | 2.72725 | 2.59066 | 2.642966 | 1.2E-13 | -1.09442 |
| HMGN3 | 20.03348 | 20.77726 | 23.48129 | 10.0173 | 9.754955 | 10.88128 | 1.64E-13 | -1.04024 |
| FRS2 | 9.501539 | 11.44444 | 10.09513 | 3.880956 | 5.12377 | 3.854337 | 1.68E-13 | -1.06981 |
| OTUD4 | 16.95606 | 14.13736 | 13.42535 | 7.362926 | 8.642451 | 6.555514 | 2.3E-13 | -1.0068 |
| GNG12 | 17.41603 | 17.56238 | 17.15998 | 10.71003 | 11.71763 | 10.48114 | 2.6E-13 | -0.6733 |
| MTMR9 | 3.299303 | 2.465097 | 2.455044 | 1.276433 | 1.176106 | 1.150483 | 3.51E-13 | -1.06559 |
| CHFR | 18.7826 | 19.49096 | 19.60222 | 10.58693 | 11.87202 | 11.72118 | 3.64E-13 | -0.7814 |
| MAN2A1 | 14.76396 | 11.96924 | 12.72323 | 6.696976 | 6.803632 | 5.083696 | 3.79E-13 | -1.05819 |
| CLOCK | 6.215948 | 5.483184 | 4.833722 | 2.385078 | 3.126217 | 2.501648 | 4.16E-13 | -1.13711 |
| HSD17B4 | 16.88602 | 15.8896 | 21.17297 | 10.17075 | 9.876658 | 9.435896 | 4.17E-13 | -0.73575 |
| HIPK3 | 13.34142 | 11.80737 | 11.55838 | 7.041966 | 6.978996 | 5.557452 | 6.53E-13 | -0.90181 |
| FAM126B | 2.430783 | 1.823126 | 1.620177 | 0.590078 | 0.771707 | 0.452125 | 7.52E-13 | -1.45367 |
| SNX16 | 3.386069 | 3.133492 | 2.911372 | 1.156682 | 1.149191 | 1.415915 | 8.79E-13 | -1.41436 |
| ADIPOR1 | 42.93837 | 44.29485 | 43.983 | 27.92338 | 29.16892 | 24.77856 | 9.01E-13 | -0.69033 |
| CHMP2B | 70.58084 | 79.33971 | 81.57708 | 43.51065 | 51.02797 | 46.96428 | 9.11E-13 | -0.84026 |
| ERCC6L | 7.951437 | 7.058427 | 6.666328 | 3.848551 | 4.10389 | 3.535043 | 9.38E-13 | -0.90204 |
| GCC2 | 6.393803 | 6.38629 | 6.41249 | 3.950153 | 4.439037 | 3.525884 | 1.03E-12 | -0.83568 |
| RAB1A | 71.59407 | 69.76597 | 60.20862 | 45.59993 | 39.68786 | 48.74541 | 1.03E-12 | -0.66864 |
| CISD1 | 13.52626 | 13.60797 | 13.7572 | 8.045673 | 7.037371 | 8.107063 | 1.15E-12 | -0.81545 |
| RC3H2 | 14.26861 | 12.18252 | 8.282001 | 7.521554 | 7.132274 | 6.498641 | 1.28E-12 | -0.98009 |
| PANX2 | 4.877983 | 5.569793 | 5.695592 | 2.378375 | 2.128975 | 2.49254 | 1.64E-12 | -1.14472 |
| RAB27A | 6.222607 | 6.211638 | 6.840766 | 3.455708 | 3.27017 | 3.216854 | 1.82E-12 | -0.87594 |
| THAP5 | 12.7885 | 11.88544 | 10.82448 | 5.898362 | 5.848984 | 5.643667 | 1.93E-12 | -0.85389 |
| MAPRE3 | 20.9217 | 20.96057 | 17.01007 | 9.456418 | 9.105046 | 10.45798 | 2.03E-12 | -0.9901 |
| XPO1 | 67.01318 | 63.49806 | 56.67646 | 35.87082 | 37.27725 | 33.98922 | 2.41E-12 | -0.66894 |
| KLHL5 | 33.12888 | 28.15199 | 26.7409 | 20.53925 | 17.97177 | 16.34656 | 2.46E-12 | -0.73538 |
| PLCXD2 | 4.330872 | 4.271847 | 3.498971 | 1.523661 | 2.044582 | 1.742864 | 2.69E-12 | -1.09508 |
| GBA2 | 19.67119 | 18.83308 | 18.91991 | 13.40644 | 12.98385 | 11.85359 | 2.78E-12 | -0.65425 |
| PARD6B | 13.65076 | 12.40973 | 12.91102 | 7.848026 | 7.994533 | 7.421515 | 3.07E-12 | -0.81031 |
| ERO1A | 9.687148 | 9.703666 | 10.06329 | 8.016826 | 8.603018 | 7.196596 | 3.9E-12 | -0.7414 |
| SMC2 | 10.7746 | 10.85374 | 10.56068 | 6.216256 | 6.803939 | 5.450583 | 4.29E-12 | -0.80877 |
| PTCH1 | 4.079058 | 5.085064 | 3.851122 | 1.79288 | 2.836701 | 1.598213 | 4.73E-12 | -1.0533 |
| SHCBP1 | 24.27875 | 24.90097 | 24.6174 | 15.97412 | 17.01171 | 16.23468 | 5.51E-12 | -0.61689 |
| MRPS17 | 17.70673 | 17.07312 | 17.47656 | 10.24392 | 9.477661 | 10.78488 | 5.63E-12 | -0.78102 |
| RAB27B | 13.82501 | 12.81213 | 12.90891 | 8.748221 | 8.180927 | 7.219507 | 6.69E-12 | -0.78027 |
| RGPD6 | 4.56442 | 4.625857 | 4.639326 | 2.559291 | 2.290737 | 2.077101 | 7.06E-12 | -1.03833 |
| SGPP1 | 5.917478 | 4.490981 | 4.576412 | 2.256473 | 2.288659 | 2.099186 | 7.58E-12 | -1.12473 |
| CCSAP | 3.393229 | 3.419591 | 3.428414 | 1.859235 | 1.954253 | 1.738079 | 8.29E-12 | -0.87295 |
| KLHL23 | 1.805701 | 1.593877 | 2.144243 | 0.800727 | 0.626572 | 0.531481 | 9.27E-12 | -1.64425 |
| C1GALT1C1 | 13.27914 | 14.84317 | 14.09754 | 7.947792 | 7.349044 | 8.003788 | 9.31E-12 | -0.84804 |
| MFSD6 | 2.824887 | 2.650981 | 2.614496 | 0.914518 | 1.276509 | 0.842706 | 9.39E-12 | -1.20999 |
| LSM11 | 1.81191 | 1.578429 | 1.813332 | 0.782836 | 0.807308 | 0.819006 | 9.84E-12 | -1.06763 |
| CDC6 | 29.83413 | 31.82767 | 31.91443 | 18.73549 | 17.67968 | 19.97907 | 9.96E-12 | -0.72248 |
| RAB11FIP2 | 4.167715 | 3.49985 | 3.417639 | 1.328316 | 1.906521 | 1.386859 | 1.06E-11 | -1.19533 |
| SLC19A2 | 6.59315 | 6.360991 | 6.001145 | 3.488715 | 3.868033 | 2.773787 | 1.14E-11 | -0.94232 |
| FGF2 | 6.237356 | 5.874445 | 5.047178 | 2.929284 | 3.282708 | 2.602343 | 1.16E-11 | -0.99639 |
| ANAPC16 | 12.75846 | 13.59927 | 12.71404 | 8.093117 | 8.182948 | 8.367302 | 1.18E-11 | -0.67454 |
| SNX24 | 4.836359 | 3.693586 | 4.650316 | 1.625248 | 1.665535 | 2.799391 | 1.22E-11 | -1.44879 |
| C12orf4 | 10.56824 | 9.739733 | 9.667588 | 4.55724 | 5.624994 | 5.765092 | 1.34E-11 | -0.84236 |
| RGPD8 | 3.550859 | 3.360179 | 2.837991 | 1.33243 | 2.0163 | 1.134494 | 1.45E-11 | -1.04745 |
| RCAN1 | 15.23863 | 14.5758 | 11.92251 | 6.893516 | 7.25179 | 7.339698 | 1.47E-11 | -0.91049 |
| VDR | 6.041012 | 5.696419 | 5.161799 | 1.573372 | 2.795959 | 2.328482 | 1.55E-11 | -1.096 |
| SMIM13 | 4.481725 | 4.329517 | 4.18933 | 2.422983 | 1.883112 | 1.708157 | 1.56E-11 | -1.07222 |
| ELMSAN1 | 11.35328 | 10.95906 | 9.904436 | 6.12056 | 6.660741 | 5.559598 | 1.67E-11 | -0.73689 |
| BHLHB9 | 3.238935 | 3.374683 | 3.254836 | 1.457981 | 1.669095 | 1.699145 | 1.69E-11 | -0.99856 |
| ARHGEF10 | 5.844513 | 5.180374 | 5.917323 | 2.860811 | 3.330633 | 3.346007 | 1.76E-11 | -0.84145 |
| EPHA2 | 51.71989 | 43.66747 | 45.67346 | 25.91855 | 30.17494 | 27.87429 | 1.88E-11 | -0.74939 |
| NUP188 | 27.89024 | 24.43793 | 23.55746 | 14.10058 | 16.27403 | 14.77255 | 1.99E-11 | -0.75016 |
| CARD19 | 27.28097 | 29.8809 | 30.76416 | 8.580099 | 7.175841 | 13.18709 | 1.99E-11 | -1.3363 |
| FAM198B | 13.11818 | 12.59412 | 12.71541 | 7.56751 | 7.884911 | 6.057211 | 2.02E-11 | -0.81633 |
| ZCCHC10 | 7.039029 | 7.596799 | 5.98032 | 3.477768 | 2.188537 | 1.602965 | 2.19E-11 | -1.48661 |
| TAP1 | 7.339839 | 7.420756 | 8.056802 | 4.046252 | 4.239741 | 4.447639 | 2.58E-11 | -0.83347 |
| PURB | 9.025339 | 8.475496 | 8.374969 | 5.30374 | 5.690684 | 5.743305 | 2.8E-11 | -0.63697 |
| HAT1 | 48.84898 | 51.39066 | 51.34627 | 31.38014 | 26.89838 | 32.32214 | 3.07E-11 | -0.74307 |
| SEC24D | 8.494981 | 8.432493 | 8.77424 | 4.176253 | 5.342668 | 4.501257 | 3.51E-11 | -0.86265 |
| PYURF | 55.43224 | 57.90345 | 56.39559 | 38.54265 | 33.66727 | 36.63901 | 4.12E-11 | -0.64876 |
| ZDHHC17 | 6.180944 | 4.898178 | 5.125258 | 3.379234 | 3.880966 | 3.133892 | 4.71E-11 | -0.80717 |
| PREP | 11.12373 | 10.71946 | 11.40913 | 6.379931 | 4.632369 | 7.93417 | 4.85E-11 | -1.02162 |
| SNRPD3 | 48.87669 | 65.19426 | 62.83256 | 31.76618 | 28.05584 | 31.33653 | 5E-11 | -0.92944 |
| APOL6 | 2.651716 | 2.415776 | 2.323042 | 1.245072 | 1.43622 | 1.087921 | 5.14E-11 | -0.94867 |
| PFN2 | 73.77328 | 77.11352 | 76.01695 | 46.2541 | 50.946 | 48.48445 | 5.14E-11 | -0.65626 |
| ZBTB33 | 14.00491 | 11.99122 | 10.9872 | 7.092353 | 7.292213 | 6.770395 | 5.28E-11 | -0.80961 |
| AP4E1 | 3.003888 | 2.473489 | 2.375972 | 1.86534 | 1.89987 | 1.095606 | 5.38E-11 | -1.14992 |
| RBPMS2 | 7.342079 | 9.843316 | 8.378478 | 2.372323 | 3.3066 | 3.781612 | 5.38E-11 | -1.26478 |
| GNA13 | 58.82754 | 54.98437 | 53.40198 | 34.64964 | 39.01143 | 32.8903 | 5.58E-11 | -0.66145 |
| Homo_sapiens_newGene_4925 | 3.138613 | 2.478959 | 2.131828 | 0.83472 | 0.826132 | 0.717056 | 5.64E-11 | -1.50768 |
| SLC26A2 | 0.76741 | 0.975979 | 0.684889 | 0.302604 | 0.26454 | 0.221309 | 5.87E-11 | -1.38322 |
| USP46 | 2.379063 | 2.854683 | 2.309152 | 0.685662 | 1.209177 | 0.978755 | 5.98E-11 | -1.29303 |
| MORC4 | 61.86181 | 61.17106 | 58.61087 | 35.68161 | 42.2288 | 39.26199 | 7.26E-11 | -0.64772 |
| Homo_sapiens_newGene_16135 | 6.288061 | 6.116919 | 6.232737 | 2.902456 | 2.138687 | 2.74479 | 7.38E-11 | -1.25564 |
| TUBD1 | 6.466142 | 5.382719 | 5.520253 | 2.488615 | 2.130697 | 2.336288 | 7.87E-11 | -1.12398 |
| PRDM1 | 2.715328 | 2.396633 | 2.858109 | 1.264971 | 1.432215 | 1.199537 | 8.11E-11 | -0.99408 |
| FAM220A | 9.807083 | 10.18494 | 9.321479 | 5.649728 | 4.747351 | 5.492578 | 8.39E-11 | -0.87112 |
| SREK1IP1 | 2.436548 | 2.539484 | 2.433381 | 1.422019 | 1.081547 | 1.188111 | 9.56E-11 | -0.97646 |
| PPP1CC | 80.47932 | 91.81699 | 79.92663 | 53.25435 | 50.19107 | 55.45184 | 1.06E-10 | -0.68549 |
| COX7A2L | 37 | 47.86542 | 47.27987 | 29.0114 | 27.61552 | 25.4222 | 1.19E-10 | -0.70049 |
| BAX | 106.7446 | 117.4919 | 111.0928 | 66.65822 | 64.68192 | 73.76826 | 1.2E-10 | -0.70292 |
| PCSK6 | 9.797878 | 7.600414 | 8.673409 | 5.27625 | 5.668654 | 4.571962 | 1.43E-10 | -0.76898 |
| DHCR7 | 43.50794 | 35.44779 | 41.42539 | 20.72864 | 18.09447 | 18.66029 | 1.43E-10 | -0.93296 |
| CEP97 | 2.891288 | 2.545895 | 2.174169 | 1.003531 | 1.360991 | 1.419131 | 1.59E-10 | -1.09853 |
| SERTAD2 | 23.75719 | 21.63441 | 20.14284 | 10.89255 | 14.00876 | 10.78663 | 1.62E-10 | -0.86399 |
| CDK6 | 12.57552 | 10.94926 | 9.351342 | 6.10655 | 6.831169 | 5.602173 | 1.64E-10 | -0.89079 |
| MVK | 12.75115 | 12.15391 | 12.45875 | 7.480469 | 7.90275 | 7.663496 | 1.73E-10 | -0.76398 |
| SLC7A5 | 313.3391 | 297.1553 | 300.9046 | 130.5171 | 75.07403 | 145.5702 | 1.8E-10 | -1.27156 |
| RAD1 | 8.497853 | 6.400993 | 6.200901 | 6.628508 | 6.260266 | 2.230433 | 1.8E-10 | -0.81563 |
| CLIC4 | 21.99539 | 21.27961 | 22.0923 | 14.59685 | 14.05015 | 11.77424 | 1.87E-10 | -0.69811 |
| LPIN1 | 2.245588 | 1.925706 | 1.852425 | 0.908318 | 1.058488 | 1.018367 | 1.99E-10 | -1.06737 |
| LRRC59 | 102.5718 | 106.0634 | 109.4681 | 62.66976 | 70.57056 | 73.32314 | 2.03E-10 | -0.64544 |
| RGPD5 | 3.759609 | 4.224288 | 3.983339 | 2.73006 | 1.595774 | 2.309284 | 2.05E-10 | -0.98837 |
| SLC43A3 | 5.906282 | 6.229545 | 5.462065 | 1.972729 | 2.382826 | 2.993865 | 2.09E-10 | -1.15973 |
| ANLN | 72.66121 | 65.90334 | 66.9992 | 44.94278 | 46.1034 | 39.53627 | 2.2E-10 | -0.65477 |
| AREL1 | 6.879568 | 6.630849 | 7.190785 | 3.418638 | 4.030425 | 3.267237 | 2.51E-10 | -0.88823 |
| ZSCAN25 | 14.23848 | 10.63966 | 11.11799 | 7.431986 | 8.149695 | 7.941595 | 2.76E-10 | -0.72213 |
| MPST | 23.90412 | 29.52179 | 27.96863 | 12.99731 | 7.816951 | 12.28224 | 3.03E-10 | -1.17619 |
| CYTH4 | 3.175506 | 1.989826 | 2.498714 | 0.84998 | 0.910229 | 0.969657 | 3.03E-10 | -1.29418 |
| DPY19L4 | 11.40593 | 9.526063 | 11.5193 | 7.494471 | 6.207956 | 4.385621 | 3.35E-10 | -0.96507 |
| FAM3C | 28.07145 | 27.02363 | 25.31987 | 14.17328 | 15.52677 | 17.54748 | 3.98E-10 | -0.76073 |
| UBASH3B | 28.90571 | 26.30843 | 27.22947 | 14.22931 | 18.46521 | 15.8695 | 4.5E-10 | -0.76041 |
| SLC16A6 | 4.38271 | 3.504933 | 3.206278 | 1.757175 | 1.773936 | 1.905121 | 5E-10 | -1.23226 |
| CCNH | 17.31402 | 15.36931 | 14.78209 | 8.753974 | 9.586015 | 9.94904 | 5.1E-10 | -0.79664 |
| RASSF8 | 5.197741 | 4.419874 | 6.315344 | 2.992074 | 2.923313 | 4.106702 | 5.15E-10 | -0.77546 |
| LEPROTL1 | 12.42852 | 11.64056 | 12.1491 | 8.564748 | 7.601353 | 8.041526 | 5.52E-10 | -0.63616 |
| NFU1 | 17.92232 | 18.45881 | 20.62011 | 6.487596 | 8.394645 | 10.2107 | 5.79E-10 | -1.09496 |
| PIK3C2A | 15.5336 | 13.49208 | 12.83607 | 7.723339 | 8.0109 | 5.570572 | 6.05E-10 | -0.95136 |
| MPV17 | 28.11762 | 32.3186 | 32.47694 | 15.80627 | 12.58889 | 16.81756 | 6.28E-10 | -0.97876 |
| HAUS6 | 4.986204 | 4.604542 | 4.11638 | 2.216249 | 2.731826 | 2.015705 | 6.37E-10 | -0.94768 |
| ABT1 | 10.26331 | 10.27989 | 10.18031 | 6.334494 | 6.166654 | 6.732177 | 6.39E-10 | -0.67878 |
| EOGT | 6.046958 | 4.926733 | 5.867849 | 3.008654 | 3.317833 | 2.620379 | 6.53E-10 | -0.9181 |
| SLC25A30 | 1.642449 | 1.590182 | 1.40967 | 0.586483 | 0.482519 | 0.388027 | 6.81E-10 | -1.37732 |
| C5orf51 | 5.316044 | 4.885446 | 5.009355 | 3.140126 | 2.843904 | 2.398362 | 7.15E-10 | -0.84739 |
| RAB8B | 6.531288 | 8.295492 | 6.705046 | 3.300155 | 4.259683 | 5.091741 | 7.5E-10 | -0.89225 |
| DUSP2 | 5.366708 | 6.601095 | 7.20129 | 2.966496 | 2.28502 | 2.726433 | 8.15E-10 | -1.17779 |
| CYP51A1 | 35.09091 | 34.02932 | 31.38301 | 18.85356 | 22.67843 | 21.2554 | 8.58E-10 | -0.68784 |
| CHST11 | 8.718656 | 8.429015 | 8.22692 | 4.195742 | 5.767102 | 4.820464 | 8.65E-10 | -0.80757 |
| SMAD5 | 7.001749 | 10.59787 | 5.276434 | 3.516596 | 3.513333 | 2.293811 | 9.23E-10 | -1.05184 |
| SGK3 | 1.855036 | 1.873548 | 1.893181 | 0.910724 | 0.850084 | 0.816029 | 9.5E-10 | -1.14973 |
| PLS1 | 44.87174 | 36.8241 | 33.86551 | 25.24786 | 33.80709 | 22.22182 | 9.97E-10 | -0.67654 |
| Homo_sapiens_newGene_4442 | 1.195289 | 0.923825 | 0.898452 | 0.441748 | 0.357161 | 0.289897 | 1.01E-09 | -1.33115 |
| RNFT1 | 5.008274 | 4.432368 | 5.421854 | 2.079328 | 2.261719 | 2.19144 | 1.06E-09 | -1.20582 |
| RPS6KA3 | 19.93001 | 19.18748 | 18.48315 | 13.11337 | 14.36972 | 16.41514 | 1.09E-09 | -0.73985 |
| MICB | 9.683811 | 8.953062 | 8.072781 | 4.940791 | 5.292447 | 4.851388 | 1.13E-09 | -0.81407 |
| TRAF5 | 4.160428 | 4.066768 | 3.604469 | 1.787209 | 2.260621 | 1.939911 | 1.16E-09 | -0.95184 |
| SELENOT | 39.58638 | 24.745 | 23.87453 | 22.37345 | 13.3873 | 14.50108 | 1.23E-09 | -0.95162 |
| RAB23 | 3.911829 | 4.248953 | 4.018784 | 2.842623 | 2.513815 | 2.252886 | 1.33E-09 | -0.76394 |
| 44085 | 18.62842 | 17.12943 | 17.41408 | 9.394447 | 8.930441 | 8.731203 | 1.33E-09 | -1.35115 |
| PLEKHA3 | 2.587276 | 2.124303 | 1.690822 | 1.305056 | 1.640206 | 1.232692 | 1.62E-09 | -0.70303 |
| PIK3R3 | 1.842302 | 1.995384 | 2.012308 | 1.683037 | 0.953905 | 0.79516 | 1.81E-09 | -0.9839 |
| EBP | 59.45148 | 58.21781 | 56.70958 | 30.7049 | 38.14616 | 49.9541 | 1.98E-09 | -0.76054 |
| PLOD2 | 74.22201 | 67.54207 | 74.24063 | 39.58079 | 52.5668 | 45.41496 | 2.37E-09 | -0.66493 |
| AK3 | 5.1118 | 4.107674 | 5.358437 | 1.693084 | 2.467514 | 1.965162 | 2.75E-09 | -1.13082 |
| FOXN2 | 7.192623 | 6.466347 | 6.008573 | 3.617443 | 4.353521 | 3.167091 | 3E-09 | -0.85343 |
| KLHL2 | 8.330121 | 6.254451 | 6.672258 | 3.960738 | 3.91429 | 3.7179 | 3.43E-09 | -0.86367 |
| CCNG1 | 14.87387 | 23.51591 | 22.82989 | 8.501652 | 6.502466 | 10.12061 | 3.91E-09 | -1.22729 |
| PLSCR4 | 1.630323 | 2.415274 | 1.789239 | 0.387722 | 0.895193 | 0.361014 | 4.67E-09 | -1.62052 |
| DUSP6 | 23.6085 | 20.64878 | 22.96007 | 21.34382 | 15.23633 | 16.75689 | 5.06E-09 | -0.62811 |
| NT5DC3 | 2.98057 | 2.643445 | 2.914629 | 1.602561 | 1.785593 | 1.474963 | 5.1E-09 | -0.8126 |
| ACKR3 | 36.87105 | 37.95029 | 36.67825 | 13.44824 | 20.8354 | 20.69069 | 5.29E-09 | -0.97815 |
| SLC39A8 | 2.616396 | 2.786258 | 2.704596 | 1.024824 | 1.396602 | 1.140935 | 5.31E-09 | -1.10245 |
| SH2B3 | 19.8396 | 17.8255 | 17.6292 | 10.23231 | 12.58278 | 10.70078 | 5.33E-09 | -0.71027 |
| JAG1 | 77.62176 | 68.72722 | 71.97927 | 44.29493 | 52.40308 | 42.99747 | 5.5E-09 | -0.64109 |
| GSPT1 | 102.14 | 47.32067 | 52.57575 | 29.7762 | 32.71471 | 32.24762 | 5.94E-09 | -0.62829 |
| MYBL1 | 7.670222 | 5.868893 | 5.127059 | 3.139787 | 3.196681 | 2.974603 | 6.02E-09 | -1.15696 |
| AKIRIN1 | 21.72487 | 20.8689 | 21.00577 | 12.09222 | 11.95219 | 8.161904 | 6.46E-09 | -0.90369 |
| DCUN1D1 | 6.283023 | 6.043633 | 6.32288 | 4.236383 | 4.846825 | 7.300312 | 6.66E-09 | -0.59778 |
| NUP153 | 20.93873 | 17.55902 | 17.27495 | 10.81096 | 11.83247 | 8.62629 | 6.77E-09 | -0.83061 |
| SPRTN | 6.174165 | 6.259628 | 6.341007 | 3.739565 | 4.096248 | 3.839907 | 6.95E-09 | -0.6861 |
| SSH2 | 11.81766 | 11.90409 | 16.40928 | 11.95345 | 13.31101 | 11.66577 | 7.08E-09 | -0.67848 |
| DNAJC24 | 5.586182 | 6.045963 | 5.281871 | 3.581499 | 2.932606 | 3.080292 | 7.64E-09 | -0.95595 |
| LSM5 | 21.19292 | 25.84803 | 24.27052 | 14.44454 | 14.17761 | 16.39611 | 8.09E-09 | -0.66409 |
| VPS13C | 3.704049 | 3.159374 | 3.05521 | 1.941409 | 2.222412 | 1.351298 | 8.47E-09 | -1.09538 |
| KRAS | 11.62933 | 10.66697 | 9.302357 | 6.551438 | 6.285542 | 5.280402 | 9.38E-09 | -0.75975 |
| AKNA | 2.894185 | 2.92409 | 2.773702 | 0.861605 | 1.785789 | 0.794344 | 1.16E-08 | -1.14159 |
| BICDL1 | 8.816837 | 8.60556 | 8.447432 | 4.97009 | 4.917571 | 5.300036 | 1.16E-08 | -0.73563 |
| KLHL15 | 1.438135 | 1.24167 | 1.129587 | 0.509231 | 0.591527 | 0.585337 | 1.17E-08 | -1.0972 |
| RFK | 14.97052 | 15.29382 | 15.36079 | 9.736553 | 10.82493 | 9.319748 | 1.18E-08 | -0.6181 |
| SLC37A1 | 8.413612 | 8.596269 | 9.186411 | 4.64811 | 5.715517 | 6.366717 | 1.21E-08 | -0.71721 |
| SNX13 | 6.973898 | 5.970138 | 6.498463 | 4.357006 | 4.666211 | 4.561711 | 1.23E-08 | -0.63972 |
| NHLRC2 | 3.357125 | 2.971671 | 2.781974 | 1.858569 | 1.946528 | 1.778848 | 1.32E-08 | -0.70431 |
| MCTS1 | 48.58996 | 57.63082 | 53.95218 | 32.51991 | 27.64231 | 34.16933 | 1.32E-08 | -0.75578 |
| STAM2 | 4.841128 | 4.450336 | 4.582228 | 2.995722 | 3.047707 | 2.90159 | 1.38E-08 | -0.6376 |
| GTF2E1 | 8.638707 | 9.044168 | 8.577883 | 5.499206 | 5.82032 | 5.234557 | 1.64E-08 | -0.64266 |
| PRKCI | 13.37829 | 12.46668 | 12.45304 | 8.870935 | 8.30972 | 7.081921 | 1.68E-08 | -0.66187 |
| LIF | 8.750734 | 8.070372 | 7.965643 | 3.268769 | 5.12505 | 4.053661 | 1.75E-08 | -0.95133 |
| GPATCH11 | 9.604715 | 9.970986 | 8.780269 | 5.02262 | 6.194353 | 5.487757 | 1.98E-08 | -0.74422 |
| SLC25A33 | 6.751038 | 6.825573 | 6.410794 | 3.726191 | 4.097512 | 4.367086 | 2.12E-08 | -0.70907 |
| SLC39A10 | 8.893613 | 9.075126 | 9.017321 | 5.823404 | 6.376621 | 5.615431 | 2.24E-08 | -0.60769 |
| CACNB3 | 10.66505 | 9.63748 | 11.10514 | 7.169995 | 6.79402 | 6.259087 | 2.36E-08 | -0.78222 |
| E2F2 | 4.228106 | 3.947171 | 3.875808 | 2.185095 | 2.536176 | 2.46395 | 2.41E-08 | -0.73955 |
| NAB1 | 11.03896 | 10.77618 | 9.257512 | 6.309021 | 5.897212 | 5.966806 | 2.45E-08 | -0.75905 |
| GNE | 19.00715 | 19.28079 | 18.74454 | 13.58157 | 13.23438 | 10.87174 | 2.58E-08 | -0.58915 |
| RASSF6 | 2.861672 | 3.554666 | 3.439037 | 1.539644 | 1.804689 | 1.609813 | 2.77E-08 | -1.04966 |
| XPO4 | 2.779401 | 2.779082 | 2.905991 | 1.511137 | 1.888372 | 1.407259 | 2.87E-08 | -0.80207 |
| PDK1 | 3.45566 | 3.345892 | 3.601607 | 2.674334 | 3.70266 | 2.377926 | 2.94E-08 | -0.72285 |
| CDCP1 | 2.284791 | 2.204998 | 2.180408 | 0.764417 | 1.226003 | 1.033298 | 3.18E-08 | -1.06787 |
| CDC7 | 7.434969 | 7.330575 | 7.245965 | 4.234734 | 4.620009 | 3.106058 | 3.28E-08 | -0.97014 |
| CDKAL1 | 3.036783 | 3.501205 | 3.764851 | 1.573835 | 2.042044 | 1.536096 | 3.53E-08 | -0.98205 |
| ITGA2 | 11.63314 | 11.41369 | 6.028987 | 2.678464 | 4.52297 | 2.948847 | 3.64E-08 | -1.31121 |
| AP1G1 | 11.85162 | 15.45554 | 11.69752 | 7.73587 | 8.947094 | 7.774609 | 3.75E-08 | -0.59455 |
| EXTL3 | 7.638021 | 6.743568 | 7.741414 | 4.899378 | 4.922411 | 4.31478 | 3.76E-08 | -0.66856 |
| TLR4 | 2.167057 | 1.914316 | 2.004099 | 0.727885 | 0.831781 | 0.839804 | 3.77E-08 | -1.22379 |
| ZNF106 | 5.367668 | 5.128021 | 5.025661 | 3.43579 | 3.384657 | 2.807858 | 3.94E-08 | -0.67424 |
| KATNAL1 | 1.779561 | 1.39428 | 1.68342 | 1.167983 | 0.860026 | 0.896811 | 3.97E-08 | -0.97002 |
| CMC1 | 12.39647 | 15.04907 | 13.91764 | 10.40319 | 8.211107 | 9.801507 | 3.98E-08 | -0.72465 |
| CDKN3 | 33.98914 | 35.16254 | 30.34658 | 20.75933 | 20.78553 | 18.10194 | 5.17E-08 | -0.74468 |
| AKT3 | 0.67059 | 0.588865 | 0.743314 | 0.106132 | 0.450432 | 0.211198 | 5.3E-08 | -1.51988 |
| ACOX2 | 0.80541 | 0.816521 | 1.489494 | 0.228215 | 0.06145 | 0.104751 | 5.42E-08 | -1.84359 |
| CLN8 | 8.421257 | 7.703117 | 7.35945 | 3.754788 | 3.468944 | 3.510511 | 5.68E-08 | -0.76589 |
| N6AMT1 | 2.904657 | 4.175813 | 4.555515 | 2.658867 | 2.676484 | 3.108661 | 5.97E-08 | -0.74073 |
| RPS6KA5 | 0.162859 | 0.125876 | 0.316106 | 0.194261 | 0.030752 | 0.121479 | 6.14E-08 | -1.52846 |
| ZMAT3 | 3.173801 | 2.970487 | 2.350965 | 0.906848 | 1.40111 | 0.728117 | 6.14E-08 | -1.30439 |
| PHF6 | 18.13407 | 18.3066 | 17.34105 | 12.67562 | 12.5163 | 11.28932 | 6.16E-08 | -0.60114 |
| PRKCH | 0.570709 | 0.40451 | 0.764762 | 0.101352 | 0.049875 | 0.161173 | 6.59E-08 | -1.82514 |
| ADAMTS1 | 2.722146 | 2.607849 | 2.341195 | 1.029481 | 1.349089 | 0.914464 | 6.62E-08 | -1.17698 |
| CLCN5 | 4.116919 | 4.466888 | 4.398046 | 1.898159 | 2.309375 | 1.654966 | 7.8E-08 | -0.94419 |
| MMD | 13.67338 | 13.78937 | 15.63424 | 10.69242 | 8.63008 | 8.741108 | 8.35E-08 | -0.61894 |
| CHKA | 28.62913 | 32.34533 | 31.89088 | 22.15233 | 21.01592 | 20.39203 | 9.18E-08 | -0.71728 |
| POLR2M | 6.677891 | 6.432548 | 6.978505 | 4.730066 | 4.606255 | 4.26341 | 1.12E-07 | -0.62665 |
| TANGO2 | 13.88004 | 16.02649 | 15.57923 | 9.782885 | 5.642712 | 7.453827 | 1.16E-07 | -0.9867 |
| NXT2 | 7.771702 | 7.713079 | 9.207983 | 4.144367 | 4.675056 | 5.190834 | 1.17E-07 | -0.78131 |
| THBS1 | 54.27384 | 45.95338 | 44.96297 | 29.11533 | 34.34945 | 26.96359 | 1.21E-07 | -0.66104 |
| GNPTAB | 7.301964 | 7.234548 | 6.356992 | 4.527867 | 5.091915 | 4.773515 | 1.23E-07 | -0.62431 |
| ZNF740 | 6.134509 | 5.096099 | 5.470755 | 3.654089 | 3.76404 | 3.517242 | 1.23E-07 | -0.61483 |
| SHOC2 | 9.032475 | 9.041368 | 8.152577 | 6.0766 | 4.946543 | 4.710787 | 1.26E-07 | -0.72575 |
| TXNDC16 | 1.569643 | 0.816624 | 1.07897 | 0.255509 | 0.163775 | 0.339527 | 1.34E-07 | -1.5047 |
| PLEKHB2 | 25.90655 | 24.71373 | 23.08337 | 16.03837 | 15.72134 | 13.56735 | 1.36E-07 | -0.65093 |
| FOXO3 | 8.740203 | 7.737643 | 7.466384 | 3.438148 | 4.601373 | 2.238152 | 1.41E-07 | -1.12126 |
| CCNE1 | 13.52983 | 12.8402 | 11.98887 | 7.745707 | 6.632103 | 7.168484 | 1.46E-07 | -0.7479 |
| CNNM3 | 7.233498 | 6.648169 | 6.357797 | 4.18103 | 4.517895 | 4.082765 | 1.52E-07 | -0.66174 |
| ADAMTS12 | 9.162875 | 7.843975 | 9.612967 | 4.911541 | 5.415758 | 8.769975 | 1.64E-07 | -0.77679 |
| SAMD9L | 4.4761 | 4.417044 | 4.052305 | 1.374483 | 2.779688 | 1.54842 | 1.72E-07 | -1.07088 |
| CSF2 | 4.162037 | 2.957087 | 3.743353 | 0.533932 | 0.718849 | 1.18978 | 2E-07 | -1.60233 |
| PHF5A | 18.41731 | 22.69574 | 21.40085 | 12.37548 | 10.56727 | 12.79777 | 2.12E-07 | -0.78686 |
| MYD88 | 8.922725 | 22.80884 | 16.00481 | 7.720352 | 9.856077 | 5.138862 | 2.52E-07 | -1.1547 |
| EREG | 37.94881 | 32.85989 | 32.31736 | 19.30501 | 24.6478 | 19.05936 | 2.54E-07 | -0.70489 |
| FAM45A | 9.831961 | 11.89525 | 10.35659 | 6.189843 | 5.461279 | 6.087736 | 2.55E-07 | -0.75508 |
| GJB3 | 3.831028 | 3.160616 | 4.497537 | 1.561588 | 1.971153 | 1.622298 | 2.57E-07 | -1.08043 |
| GNPDA2 | 6.634644 | 6.14784 | 7.700007 | 2.129417 | 3.0495 | 2.055516 | 2.58E-07 | -1.06849 |
| PURA | 1.300963 | 1.281956 | 1.268147 | 0.849913 | 0.7139 | 0.712024 | 2.64E-07 | -0.74629 |
| HINT3 | 7.513506 | 6.903093 | 8.066645 | 4.126766 | 4.66653 | 4.868912 | 2.83E-07 | -0.70985 |
| TAF9B | 22.9314 | 23.22648 | 20.38127 | 13.50989 | 16.04524 | 13.79787 | 2.84E-07 | -0.62124 |
| MTLN | 9.366121 | 10.17164 | 12.15302 | 3.018504 | 3.502387 | 4.684615 | 2.99E-07 | -1.30552 |
| MAP2K1 | 14.90139 | 13.25637 | 15.32635 | 9.700137 | 8.737886 | 9.990098 | 3.07E-07 | -0.61056 |
| SGCE | 30.06618 | 32.2019 | 29.39883 | 21.01873 | 17.04353 | 21.62961 | 3.07E-07 | -0.64147 |
| PAQR3 | 5.383681 | 6.347871 | 5.170033 | 3.379609 | 3.844701 | 3.850048 | 3.2E-07 | -0.60277 |
| NBPF1 | 11.44851 | 11.1708 | 10.51385 | 6.781472 | 8.163083 | 6.531359 | 3.26E-07 | -0.62845 |
| KCTD9 | 14.67655 | 15.09944 | 14.1584 | 8.678709 | 10.62218 | 8.955538 | 3.33E-07 | -0.63122 |
| CD109 | 23.28436 | 20.82414 | 18.78643 | 12.83142 | 14.67053 | 11.06558 | 3.35E-07 | -0.70052 |
| GPR63 | 0.446926 | 0.399193 | 0.403512 | 0.055522 | 0.137104 | 0.092831 | 3.59E-07 | -1.58381 |
| CHAC2 | 11.33979 | 13.13126 | 13.83018 | 7.349502 | 7.861984 | 7.473604 | 3.9E-07 | -0.74307 |
| MTO1 | 4.186256 | 3.156824 | 4.07586 | 1.800636 | 1.383352 | 1.375936 | 3.92E-07 | -0.6568 |
| Homo_sapiens_newGene_8824 | 4.914978 | 5.083949 | 6.156433 | 2.251354 | 3.394024 | 3.056903 | 3.97E-07 | -0.75783 |
| TMEM209 | 11.03024 | 9.827477 | 9.955591 | 7.534494 | 7.113181 | 5.815192 | 3.97E-07 | -0.61104 |
| NXPE3 | 10.66264 | 11.03469 | 10.43356 | 7.496312 | 7.85387 | 6.09273 | 4.01E-07 | -0.59498 |
| Homo_sapiens_newGene_15905 | 15.70266 | 13.50813 | 14.72127 | 8.052309 | 9.477821 | 7.704727 | 4.32E-07 | -0.89992 |
| NFKB1 | 15.76284 | 14.79301 | 14.82543 | 10.35347 | 11.44336 | 9.813779 | 4.57E-07 | -0.5901 |
| STK4 | 11.98185 | 9.670863 | 9.402933 | 5.447261 | 7.516917 | 4.593569 | 5.15E-07 | -0.80557 |
| PRKCE | 4.093276 | 3.008569 | 3.117667 | 3.624609 | 4.111583 | 3.044822 | 5.41E-07 | -0.85482 |
| RBM12B | 8.453647 | 10.26311 | 9.480205 | 6.334516 | 6.002987 | 6.560266 | 5.67E-07 | -0.63883 |
| MEGF6 | 10.16289 | 8.425575 | 9.700062 | 6.250656 | 6.068682 | 7.270393 | 5.77E-07 | -0.63046 |
| NR2C2 | 11.46684 | 10.21922 | 11.01233 | 7.545329 | 7.164199 | 4.834906 | 5.98E-07 | -0.76647 |
| VPS54 | 9.585845 | 10.20425 | 9.393446 | 6.139178 | 6.730785 | 4.522032 | 6.44E-07 | -0.73781 |
| MMACHC | 1.484899 | 1.238706 | 1.383442 | 0.756545 | 0.501015 | 0.584312 | 7.07E-07 | -1.05779 |
| LEPROT | 7.085268 | 8.253179 | 7.514245 | 5.026296 | 5.187923 | 3.933614 | 7.41E-07 | -0.71251 |
| HSPA14 | 6.215364 | 6.871466 | 6.40956 | 3.439809 | 4.556717 | 3.68962 | 7.51E-07 | -0.74715 |
| TARBP1 | 5.50581 | 5.632321 | 5.089588 | 3.734124 | 3.687425 | 3.182854 | 8.56E-07 | -0.61551 |
| PKDCC | 8.428137 | 9.748361 | 9.794673 | 5.936878 | 6.121266 | 5.980016 | 9.79E-07 | -0.63158 |
| TFEC | 1.196926 | 0.866007 | 0.868371 | 0.35647 | 0.406819 | 0.294531 | 9.86E-07 | -1.15884 |
| FMR1 | 13.68557 | 14.0785 | 11.94142 | 9.176918 | 8.607093 | 8.575409 | 1.02E-06 | -0.69241 |
| SMIM10L1 | 5.741904 | 6.028619 | 5.971978 | 3.650151 | 3.641346 | 4.230752 | 1.04E-06 | -0.62119 |
| SNRNP200 | 51.00565 | 44.41552 | 44.23484 | 30.85512 | 34.4765 | 27.00001 | 1.05E-06 | -0.6007 |
| C21orf91 | 0.916681 | 0.902297 | 0.936668 | 0.30071 | 0.370188 | 0.631757 | 1.1E-06 | -1.13968 |
| RFX7 | 2.881485 | 2.907574 | 2.346861 | 1.253465 | 1.716533 | 1.019081 | 1.11E-06 | -0.95641 |
| ANKRD13C | 9.122205 | 8.744542 | 7.704031 | 5.246443 | 5.882224 | 5.727045 | 1.12E-06 | -0.59971 |
| IFNLR1 | 4.249788 | 3.303122 | 3.340047 | 1.968265 | 1.946938 | 2.273115 | 1.12E-06 | -0.75914 |
| PARG | 6.926411 | 5.707673 | 5.837011 | 4.185904 | 3.363598 | 2.560903 | 1.15E-06 | -0.77713 |
| IFI16 | 18.62326 | 15.72028 | 14.57762 | 12.16556 | 10.1831 | 9.923886 | 1.27E-06 | -0.58566 |
| NUAK1 | 5.362793 | 4.459359 | 4.179868 | 2.994116 | 3.213965 | 2.68655 | 1.42E-06 | -0.69106 |
| FBLIM1 | 42.81343 | 38.03413 | 27.94021 | 17.42113 | 21.68514 | 16.29387 | 1.45E-06 | -0.93593 |
| SCOC | 15.52026 | 15.86084 | 17.22104 | 11.60534 | 10.01823 | 11.15952 | 1.49E-06 | -0.58734 |
| MOSPD2 | 2.660584 | 2.778441 | 2.895431 | 1.690031 | 0.991522 | 1.078099 | 1.54E-06 | -0.88243 |
| TMEM170B | 0.140407 | 0.211358 | 0.246235 | 0.020406 | 0.048807 | 0.019823 | 1.6E-06 | -1.65748 |
| CDKN1A | 37.47391 | 41.19334 | 40.24906 | 18.63858 | 10.97194 | 22.86386 | 1.63E-06 | -1.09078 |
| SIRT1 | 7.202048 | 6.248649 | 7.742095 | 4.440461 | 4.646708 | 4.542672 | 1.69E-06 | -0.63182 |
| MICA | 12.61371 | 15.42779 | 13.7317 | 9.463308 | 8.596439 | 9.78775 | 1.7E-06 | -0.63109 |
| KBTBD8 | 1.721732 | 1.12859 | 1.298727 | 0.790954 | 0.854797 | 1.01416 | 1.73E-06 | -1.45009 |
| MBLAC2 | 1.040259 | 1.2048 | 0.870225 | 0.354124 | 0.580186 | 0.303585 | 1.82E-06 | -1.28446 |
| RNF2 | 10.14679 | 9.462961 | 9.778449 | 5.148902 | 7.323887 | 7.198584 | 1.98E-06 | -0.61638 |
| PDZD8 | 10.0613 | 8.666489 | 8.875453 | 6.567632 | 6.121122 | 4.950451 | 1.99E-06 | -0.64456 |
| ORMDL1 | 15.8281 | 20.98814 | 21.81534 | 12.43166 | 11.8035 | 11.43515 | 2.4E-06 | -0.73202 |
| CAPN7 | 14.1112 | 12.14193 | 13.85358 | 10.01432 | 9.042862 | 7.211285 | 2.41E-06 | -0.64446 |
| FAM214B | 7.282105 | 3.916576 | 6.887179 | 2.398178 | 2.201944 | 2.736978 | 2.51E-06 | -1.05777 |
| STYK1 | 3.465897 | 5.185616 | 4.069313 | 1.388607 | 1.92615 | 1.76353 | 2.55E-06 | -0.96005 |
| MSH2 | 22.91405 | 24.17934 | 19.3278 | 14.93955 | 15.54891 | 13.66689 | 2.55E-06 | -0.5918 |
| Homo_sapiens_newGene_19628 | 8.062128 | 9.016675 | 9.016402 | 5.329724 | 5.711824 | 5.901639 | 2.56E-06 | -0.60933 |
| PRSS23 | 105.376 | 106.9665 | 53.63103 | 26.54605 | 24.52118 | 23.64111 | 2.57E-06 | -1.58641 |
| VPS37C | 13.43029 | 11.18688 | 11.01361 | 8.183329 | 7.629319 | 7.752896 | 2.74E-06 | -0.60522 |
| HSPA4L | 3.395056 | 3.002157 | 3.340195 | 2.318032 | 2.242106 | 1.93345 | 2.82E-06 | -0.58886 |
| C10orf88 | 2.8416 | 2.739298 | 2.711339 | 1.527241 | 1.555954 | 1.285901 | 2.84E-06 | -0.87664 |
| TMED5 | 26.57946 | 19.18457 | 25.51244 | 17.02798 | 16.43363 | 15.7829 | 2.93E-06 | -0.67382 |
| PARPBP | 10.21827 | 10.17502 | 6.434793 | 3.836311 | 5.377029 | 3.812187 | 2.96E-06 | -0.95861 |
| ZFAND1 | 23.2906 | 18.15287 | 22.37987 | 14.63964 | 15.58595 | 13.56393 | 3E-06 | -0.67056 |
| GM2A | 6.948194 | 7.22762 | 6.509035 | 4.214588 | 3.896831 | 4.823338 | 3.03E-06 | -0.64754 |
| ANXA3 | 12.39595 | 14.72752 | 11.66592 | 6.251152 | 7.346245 | 8.228148 | 3.03E-06 | -0.79428 |
| GLIPR1 | 25.50912 | 25.3842 | 21.05506 | 9.572038 | 13.37435 | 13.16532 | 3.18E-06 | -0.74787 |
| LGR4 | 21.07696 | 18.98124 | 19.15499 | 13.14914 | 6.755781 | 9.670446 | 3.27E-06 | -0.93867 |
| PCDHB6 | 2.617905 | 3.28878 | 2.821635 | 1.548066 | 1.673115 | 1.470203 | 3.29E-06 | -0.84097 |
| C12orf29 | 25.53444 | 33.57689 | 31.53741 | 20.94862 | 24.92577 | 25.16939 | 3.31E-06 | -0.59064 |
| ELF2 | 7.83706 | 6.690193 | 6.43191 | 2.801804 | 4.388967 | 3.816587 | 3.71E-06 | -0.88976 |
| SMAP2 | 10.87778 | 9.617873 | 12.19715 | 6.500119 | 7.101263 | 7.236336 | 3.99E-06 | -0.64415 |
| CLDND1 | 22.4621 | 21.7212 | 24.19016 | 13.82283 | 14.07143 | 12.38589 | 4.18E-06 | -0.63852 |
| SEC11A | 40.53966 | 46.11952 | 41.5657 | 23.33262 | 33.16394 | 22.53218 | 4.28E-06 | -0.69794 |
| PTBP2 | 3.117279 | 3.656828 | 2.259649 | 0.754564 | 0.667669 | 0.613493 | 4.55E-06 | -1.13461 |
| CLDN2 | 11.6865 | 10.7809 | 13.17133 | 8.572001 | 7.231712 | 6.979204 | 4.8E-06 | -0.65655 |
| B3GALT5 | 5.457934 | 4.844935 | 4.762276 | 2.75071 | 3.671277 | 2.76868 | 5.03E-06 | -0.69054 |
| TRIM23 | 1.986217 | 2.007234 | 2.51064 | 1.067224 | 1.399767 | 0.935757 | 5.5E-06 | -0.9425 |
| RCOR3 | 13.01282 | 9.282194 | 10.60962 | 5.315103 | 6.082769 | 6.633789 | 5.68E-06 | -0.73888 |
| BMP2K | 4.819869 | 4.558577 | 4.688273 | 2.549698 | 3.639615 | 2.484993 | 5.84E-06 | -0.78961 |
| HSD17B7 | 6.48611 | 6.265319 | 5.459189 | 3.36751 | 2.927751 | 3.419616 | 5.9E-06 | -0.9074 |
| HECTD1 | 10.89251 | 12.46659 | 11.51961 | 8.338077 | 9.620973 | 7.961753 | 5.95E-06 | -0.63977 |
| SAMD4A | 4.336813 | 3.796455 | 3.615644 | 2.272518 | 2.684497 | 2.409075 | 6.2E-06 | -0.6473 |
| TMTC4 | 1.450458 | 1.397594 | 1.316477 | 0.704621 | 0.535795 | 0.594686 | 6.9E-06 | -1.05916 |
| JADE1 | 7.274846 | 6.242167 | 6.740386 | 3.977529 | 4.91485 | 3.558123 | 7.27E-06 | -0.79461 |
| Homo_sapiens_newGene_16208 | 3.518017 | 3.081655 | 2.772133 | 1.705005 | 2.428523 | 1.510896 | 7.34E-06 | -0.77411 |
| PARP12 | 8.65571 | 8.905799 | 8.310686 | 4.93299 | 5.728525 | 6.44303 | 7.9E-06 | -0.62912 |
| BTBD7 | 5.94494 | 6.008066 | 4.692883 | 3.939357 | 4.474501 | 4.108951 | 8.6E-06 | -0.60791 |
| SERPINB9 | 2.385211 | 2.466167 | 2.37946 | 1.451812 | 1.47007 | 1.506894 | 9.48E-06 | -0.68935 |
| MOB1B | 4.018845 | 3.007016 | 2.873105 | 1.966816 | 2.116881 | 2.133583 | 9.63E-06 | -0.66741 |
| AP1S2 | 6.579173 | 7.67026 | 6.175859 | 4.191779 | 5.076483 | 4.251195 | 9.85E-06 | -0.69333 |
| JAZF1 | 3.482534 | 2.819633 | 3.292113 | 1.981764 | 1.422855 | 1.763464 | 1E-05 | -0.85632 |
| HMGA2 | 53.54275 | 46.82759 | 37.68269 | 20.63979 | 32.109 | 27.14536 | 1.02E-05 | -0.61908 |
| MMP1 | 8.022429 | 9.048779 | 8.591561 | 6.069894 | 5.192417 | 5.102455 | 1.02E-05 | -0.64261 |
| BRIP1 | 5.202629 | 4.61297 | 4.17186 | 2.428893 | 3.282541 | 2.033482 | 1.02E-05 | -0.80716 |
| FYCO1 | 4.098398 | 4.645455 | 4.381827 | 3.131026 | 2.76524 | 2.46802 | 1.06E-05 | -0.70102 |
| LRRC8B | 2.730061 | 2.547804 | 2.677654 | 1.45421 | 1.944654 | 1.668722 | 1.06E-05 | -0.71248 |
| AMPD3 | 9.139956 | 10.56486 | 7.210524 | 5.869171 | 5.94961 | 4.410438 | 1.07E-05 | -0.71076 |
| KRIT1 | 10.61038 | 10.61264 | 8.001034 | 2.815825 | 3.972217 | 3.599584 | 1.09E-05 | -1.24402 |
| FAM174B | 8.315361 | 7.421566 | 8.676318 | 5.095992 | 5.252469 | 6.004533 | 1.18E-05 | -0.59223 |
| GLMN | 5.615041 | 5.663918 | 5.040708 | 2.07448 | 2.320844 | 3.428343 | 1.22E-05 | -0.95536 |
| RUFY3 | 10.33901 | 9.446926 | 9.946834 | 7.000274 | 6.083643 | 7.613704 | 1.24E-05 | -0.59413 |
| PLAUR | 23.74264 | 23.04948 | 23.52314 | 12.33597 | 14.21225 | 18.60441 | 1.24E-05 | -0.69395 |
| BRWD3 | 2.755342 | 2.482335 | 2.315043 | 1.635621 | 1.713157 | 1.225569 | 1.36E-05 | -0.70746 |
| KDR | 3.931452 | 3.75319 | 3.812778 | 1.683737 | 2.703847 | 1.846872 | 1.38E-05 | -0.82157 |
| ARL15 | 0.954526 | 0.989972 | 1.257923 | 0.787398 | 0.37344 | 0.38498 | 1.45E-05 | -1.20919 |
| WDR7 | 1.758074 | 1.301153 | 1.618922 | 0.401435 | 0.860446 | 0.635992 | 1.46E-05 | -0.94788 |
| GCOM1 | 1.059266 | 0.646807 | 0.98377 | 0.286569 | 0.259801 | 0.964204 | 1.49E-05 | -1.32265 |
| KLHL4 | 7.542326 | 7.721651 | 7.540153 | 3.933893 | 5.700908 | 4.49838 | 1.5E-05 | -0.67752 |
| STC2 | 17.90388 | 18.00385 | 17.20269 | 8.990715 | 13.07792 | 11.61922 | 1.5E-05 | -0.65575 |
| GID4 | 5.045911 | 6.223976 | 5.870878 | 3.797304 | 4.052565 | 3.861823 | 1.51E-05 | -0.63576 |
| ZIC2 | 2.779116 | 2.85501 | 2.652352 | 1.561013 | 1.745203 | 1.58196 | 1.68E-05 | -0.73799 |
| ZDHHC23 | 2.300509 | 1.562199 | 2.114913 | 1.007925 | 1.022589 | 1.101254 | 1.68E-05 | -0.78812 |
| DDX3X | 115.4731 | 60.01699 | 101.5731 | 48.21988 | 49.62802 | 48.56242 | 1.68E-05 | -0.88224 |
| OLFM2 | 7.877043 | 7.94923 | 6.761544 | 3.68442 | 4.1616 | 4.791705 | 1.7E-05 | -0.81285 |
| GPR180 | 1.500471 | 1.345118 | 1.536858 | 1.000832 | 0.838812 | 0.737157 | 1.7E-05 | -0.7406 |
| CAMKK1 | 10.87685 | 9.71429 | 9.601073 | 5.393031 | 7.032992 | 6.892794 | 1.83E-05 | -0.64159 |
| ZBTB41 | 4.664561 | 4.074577 | 4.272697 | 2.842557 | 3.142236 | 2.256246 | 1.86E-05 | -0.65085 |
| SUSD6 | 2.602264 | 2.704648 | 2.994721 | 1.782134 | 1.87819 | 1.476972 | 1.94E-05 | -0.6794 |
| GAS2L3 | 4.031784 | 3.399017 | 4.16899 | 2.043322 | 2.530951 | 1.959726 | 2.07E-05 | -0.83549 |
| NIN | 3.916449 | 3.833026 | 3.610151 | 2.29546 | 2.920322 | 2.289078 | 2.12E-05 | -0.60761 |
| SPATA5 | 1.621001 | 1.327852 | 1.26244 | 0.868949 | 0.827116 | 0.785313 | 2.12E-05 | -0.73609 |
| ZNF488 | 2.131472 | 2.421535 | 2.32102 | 1.418073 | 1.294756 | 1.291821 | 2.12E-05 | -0.747 |
| TPK1 | 2.512079 | 1.716285 | 2.136225 | 0.986953 | 1.791791 | 1.218047 | 2.24E-05 | -0.91867 |
| CSNK2A1 | 14.7067 | 15.78415 | 28.67501 | 12.54353 | 10.10087 | 7.523177 | 2.39E-05 | -0.86532 |
| RNF19A | 9.510724 | 9.298426 | 8.414932 | 5.437108 | 6.564069 | 4.950738 | 2.47E-05 | -0.60043 |
| CABYR | 14.81404 | 16.03434 | 13.55306 | 9.797982 | 9.478358 | 10.10184 | 2.73E-05 | -0.64321 |
| Homo_sapiens_newGene_2494 | 2.900005 | 2.110137 | 2.924113 | 1.097869 | 1.06177 | 0.722403 | 3.01E-05 | -1.09875 |
| AREG | 28.53556 | 26.29008 | 26.95053 | 15.44237 | 17.84815 | 20.24649 | 3.11E-05 | -0.60415 |
| SECISBP2L | 4.692706 | 4.720672 | 3.686655 | 2.91899 | 2.835557 | 2.354319 | 3.21E-05 | -0.65022 |
| C16orf70 | 7.489851 | 6.60453 | 6.529711 | 4.308767 | 4.130068 | 4.752057 | 3.24E-05 | -0.60579 |
| KRTAP2-3 | 8.632206 | 6.839279 | 7.563092 | 1.867151 | 3.690796 | 3.734411 | 3.41E-05 | -1.11025 |
| B4GAT1 | 5.718373 | 5.547373 | 5.76782 | 3.926909 | 3.295611 | 3.608076 | 3.45E-05 | -0.64121 |
| KCTD6 | 5.206977 | 4.22617 | 5.428839 | 2.516645 | 3.097547 | 2.75461 | 3.49E-05 | -0.78956 |
| ERCC4 | 3.624141 | 3.199724 | 3.332967 | 2.119269 | 2.523968 | 2.045465 | 3.6E-05 | -0.5937 |
| ASB4 | 3.663327 | 3.186885 | 2.734283 | 1.169434 | 1.817135 | 1.742018 | 3.64E-05 | -0.79655 |
| FGD6 | 7.043692 | 7.244057 | 5.813357 | 4.644799 | 4.630703 | 3.46129 | 3.68E-05 | -0.63745 |
| ARID3B | 2.72771 | 2.212015 | 2.113979 | 1.370993 | 1.456475 | 1.26384 | 3.92E-05 | -0.75182 |
| TRIM35 | 7.647156 | 8.307081 | 8.040123 | 5.162715 | 6.127264 | 5.373466 | 4.01E-05 | -0.6048 |
| SLC25A20 | 7.832706 | 7.803111 | 8.130949 | 4.279636 | 5.430205 | 5.071965 | 4.03E-05 | -0.69104 |
| LIPG | 2.382795 | 1.842811 | 2.111398 | 0.235501 | 1.103549 | 1.412701 | 4.15E-05 | -1.12523 |
| FIGN | 1.475553 | 1.787454 | 1.454854 | 1.362905 | 0.987784 | 0.769194 | 4.29E-05 | -0.74408 |
| CDKN2D | 3.394567 | 4.515885 | 3.865254 | 1.470004 | 0.83151 | 1.747239 | 4.42E-05 | -1.22079 |
| LGR5 | 1.295128 | 1.249352 | 1.740802 | 0.928446 | 0.702335 | 0.679071 | 4.57E-05 | -0.83892 |
| COL4A2 | 1.380778 | 1.551264 | 1.516291 | 0.997355 | 0.929731 | 1.003984 | 4.98E-05 | -0.68096 |
| CENPQ | 6.69979 | 7.547703 | 6.547004 | 4.201548 | 4.807887 | 3.971128 | 5.1E-05 | -0.6619 |
| CLIC3 | 5.89292 | 5.550517 | 6.990193 | 2.652058 | 2.462161 | 3.704369 | 5.41E-05 | -0.95157 |
| Homo_sapiens_newGene_3857 | 2.610745 | 2.230065 | 1.537798 | 1.33241 | 1.101145 | 0.949401 | 5.46E-05 | -0.91128 |
| Homo_sapiens_newGene_16827 | 1.568212 | 2.00547 | 2.239282 | 0.386649 | 0.856684 | 0.903358 | 5.47E-05 | -1.17179 |
| PEX3 | 11.46666 | 15.17072 | 11.15147 | 6.826303 | 8.962378 | 7.988989 | 5.67E-05 | -0.62674 |
| DUSP9 | 3.685347 | 3.513433 | 3.563032 | 1.704494 | 2.061512 | 2.339001 | 6.09E-05 | -0.7705 |
| MAP3K7 | 10.14609 | 7.406905 | 10.70256 | 5.483682 | 5.978009 | 5.333867 | 6.29E-05 | -0.69228 |
| SEC14L1 | 42.35573 | 36.98973 | 38.36213 | 15.34432 | 26.44474 | 21.36891 | 6.73E-05 | -0.86026 |
| ABHD13 | 1.150842 | 1.383621 | 1.22431 | 0.797174 | 0.63463 | 0.580651 | 6.75E-05 | -0.83891 |
| SPRYD7 | 5.774659 | 5.637672 | 6.236777 | 2.908293 | 3.903572 | 3.971723 | 7.24E-05 | -0.68597 |
| FAM234B | 2.442026 | 2.51682 | 2.045604 | 1.53134 | 1.422674 | 1.418603 | 7.47E-05 | -0.66092 |
| KIAA0586 | 3.244228 | 3.910432 | 4.301388 | 3.225243 | 2.293912 | 1.989661 | 7.5E-05 | -0.6484 |
| Homo_sapiens_newGene_9227 | 14.73351 | 16.40599 | 18.99164 | 10.09584 | 9.240817 | 9.151646 | 7.7E-05 | -0.77152 |
| HOOK1 | 0.472009 | 0.408479 | 0.512041 | 0.216626 | 0.173443 | 0.116361 | 8.01E-05 | -1.16731 |
| Homo_sapiens_newGene_15489 | 0.769745 | 0.786453 | 1.215192 | 0.156261 | 0.140074 | 0.235932 | 8.06E-05 | -1.41059 |
| CCNE2 | 9.47261 | 8.884505 | 7.388038 | 4.097821 | 6.161351 | 5.539341 | 8.78E-05 | -0.62489 |
| ME2 | 17.08845 | 10.374 | 18.75435 | 4.010609 | 5.96088 | 4.166261 | 8.93E-05 | -0.73028 |
| Homo_sapiens_newGene_8846 | 1.842427 | 1.291785 | 1.684608 | 0.924407 | 0.843488 | 0.929217 | 9.19E-05 | -0.78761 |
| HIPK2 | 28.12949 | 26.68002 | 24.40971 | 17.98368 | 20.04389 | 12.97896 | 9.43E-05 | -0.62327 |
| TMEM168 | 5.158591 | 7.082632 | 7.864803 | 3.718307 | 3.317253 | 2.891897 | 9.76E-05 | -0.81092 |
| BNIP2 | 4.596211 | 4.611108 | 10.21917 | 3.79807 | 2.861847 | 3.341101 | 0.000106 | -0.68511 |
| XK | 0.419635 | 0.380683 | 0.300515 | 0.141588 | 0.112646 | 0.115866 | 0.000106 | -1.21269 |
| MTIF3 | 7.986232 | 8.968259 | 9.142763 | 5.650946 | 5.280592 | 5.45449 | 0.000113 | -0.65661 |
| MINDY2 | 1.176176 | 1.185715 | 1.233188 | 0.623322 | 0.695934 | 0.332369 | 0.000113 | -1.08303 |
| TMEM182 | 3.984185 | 5.266492 | 3.577687 | 2.821687 | 2.140055 | 2.245176 | 0.000118 | -0.65638 |
| IRAK4 | 7.07284 | 4.756589 | 7.701523 | 6.773022 | 5.044531 | 5.156716 | 0.000122 | -0.65032 |
| Homo_sapiens_newGene_12702 | 2.490626 | 2.422151 | 2.562981 | 1.659913 | 1.630069 | 1.497931 | 0.000123 | -0.63186 |
| Homo_sapiens_newGene_12578 | 1.082623 | 1.155349 | 1.000053 | 0.508154 | 1.122951 | 0.361008 | 0.000128 | -1.09794 |
| CMTM4 | 9.863272 | 7.382687 | 7.619529 | 5.889945 | 5.295698 | 5.459092 | 0.000131 | -0.60323 |
| PIKFYVE | 3.915636 | 3.747665 | 3.047715 | 2.413212 | 2.47364 | 2.093407 | 0.000132 | -0.79823 |
| CFL2 | 34.86176 | 39.03884 | 18.2021 | 9.572545 | 12.192 | 24.12473 | 0.000133 | -0.89368 |
| Homo_sapiens_newGene_8453 | 1.740656 | 1.876195 | 2.115223 | 0.735472 | 0.961425 | 0.650807 | 0.000151 | -0.98095 |
| Homo_sapiens_newGene_17275 | 3.015354 | 3.251053 | 3.966307 | 1.854574 | 2.006478 | 2.529689 | 0.00016 | -0.7904 |
| SACS | 3.783437 | 2.986347 | 2.72967 | 1.787416 | 2.32779 | 1.576892 | 0.000168 | -0.6977 |
| MEF2C | 2.650801 | 2.278139 | 2.29191 | 1.185607 | 1.582042 | 0.705333 | 0.000173 | -0.93956 |
| USF3 | 2.225349 | 2.243524 | 1.824861 | 1.649411 | 1.281118 | 0.801102 | 0.000186 | -0.75757 |
| KLHDC2 | 11.2983 | 11.21371 | 10.98415 | 2.335485 | 8.489676 | 7.031697 | 0.00019 | -0.62752 |
| LAMC2 | 11.74904 | 10.04461 | 10.74729 | 5.310519 | 7.858552 | 7.406005 | 0.000194 | -0.64407 |
| CYR61 | 13.12535 | 12.05594 | 11.90386 | 5.241487 | 8.778604 | 7.675845 | 0.000203 | -0.73233 |
| SYTL5 | 1.606234 | 2.002085 | 1.570138 | 1.124733 | 0.667916 | 0.864291 | 0.000203 | -0.87384 |
| Homo_sapiens_newGene_9169 | 1.44163 | 1.4218 | 1.066474 | 0.764055 | 0.649401 | 0.816273 | 0.000223 | -0.81182 |
| MPZL3 | 0.955378 | 1.067411 | 1.029527 | 0.455692 | 0.40456 | 0.224234 | 0.000225 | -1.05362 |
| SMPD2 | 9.131996 | 10.01033 | 10.41022 | 6.980497 | 5.234353 | 6.21538 | 0.00023 | -0.63453 |
| COPS7A | 46.64922 | 51.94595 | 27.08964 | 23.36959 | 22.80231 | 25.80391 | 0.000243 | -0.78066 |
| FGF5 | 0.243745 | 0.21879 | 0.222803 | 0.049676 | 0.056889 | 0.021113 | 0.000245 | -1.33526 |
| WDR41 | 4.789701 | 6.105491 | 6.213394 | 3.981853 | 3.561652 | 4.312072 | 0.000288 | -0.68236 |
| FAM200B | 3.79686 | 4.048278 | 3.948369 | 2.374556 | 2.45505 | 2.476976 | 0.000303 | -0.64799 |
| MANSC1 | 4.953033 | 3.999381 | 3.39915 | 3.427642 | 2.183911 | 2.231094 | 0.000303 | -0.79424 |
| LMCD1 | 34.75598 | 37.50883 | 37.67125 | 8.937755 | 31.76779 | 31.72223 | 0.000309 | -0.64544 |
| GPR160 | 1.547512 | 1.834101 | 1.631194 | 0.825895 | 0.713501 | 0.894741 | 0.000313 | -0.9146 |
| Homo_sapiens_newGene_100 | 5.025861 | 4.527966 | 4.431538 | 2.626101 | 3.906426 | 2.736674 | 0.00033 | -0.74289 |
| IL7R | 5.037349 | 4.020629 | 3.54413 | 2.563111 | 2.87876 | 3.006989 | 0.000331 | -0.67372 |
| SPDEF | 5.002212 | 4.943944 | 5.467262 | 3.645264 | 2.926568 | 3.291391 | 0.000336 | -0.62606 |
| SKIL | 11.36568 | 8.516377 | 9.554148 | 4.899399 | 5.357537 | 7.316911 | 0.000337 | -0.73323 |
| LYSMD2 | 4.563041 | 4.072227 | 3.280002 | 2.323344 | 1.786528 | 2.361111 | 0.000342 | -0.80652 |
| PRKAG1 | 25.19664 | 27.21557 | 26.31018 | 15.19201 | 22.04372 | 14.98691 | 0.000361 | -0.60281 |
| GPR137C | 0.707722 | 0.618554 | 0.624312 | 0.368619 | 0.185802 | 0.193885 | 0.000388 | -1.08859 |
| MDM1 | 3.290866 | 3.544907 | 3.114458 | 2.707729 | 2.181855 | 2.091358 | 0.000412 | -0.62945 |
| Homo_sapiens_newGene_18542 | 3.710249 | 3.364962 | 4.609682 | 1.471527 | 1.461339 | 2.427213 | 0.000416 | -0.97568 |
| CDV3 | 43.52198 | 78.35367 | 76.90977 | 24.10394 | 31.0022 | 33.84092 | 0.000421 | -1.12165 |
| Homo_sapiens_newGene_16980 | 2.598484 | 2.776858 | 2.973638 | 1.554231 | 1.619494 | 1.952613 | 0.000436 | -0.67071 |
| P2RY1 | 0.213603 | 0.183642 | 0.201162 | 0.067442 | 0.035305 | 0.064805 | 0.000444 | -1.2145 |
| CACNA1I | 0.20612 | 0.289941 | 0.261587 | 0.081189 | 0.09895 | 0.020655 | 0.000454 | -1.24659 |
| RB1 | 1.966343 | 1.661987 | 1.89192 | 0.925398 | 1.328807 | 0.978403 | 0.000463 | -0.72994 |
| Homo_sapiens_newGene_11399 | 2.243752 | 2.802354 | 3.099891 | 0.979103 | 1.659215 | 1.174139 | 0.000493 | -0.88138 |
| CLCN3 | 12.53655 | 10.97957 | 12.30236 | 8.843424 | 9.139612 | 7.983661 | 0.000494 | -0.61567 |
| DIPK1A | 8.893772 | 9.715146 | 8.012645 | 7.084871 | 5.731372 | 5.043564 | 0.000499 | -0.58719 |
| CMPK1 | 47.92205 | 41.03801 | 24.04635 | 8.042734 | 15.6242 | 8.205536 | 0.000516 | -1.23055 |
| COPS8 | 7.545383 | 12.54409 | 15.44163 | 6.852759 | 5.207225 | 6.706058 | 0.000522 | -0.70378 |
| TAF5 | 2.13839 | 2.163494 | 2.129882 | 1.277285 | 1.489441 | 1.269064 | 0.000533 | -0.64631 |
| Homo_sapiens_newGene_19903 | 2.902936 | 2.326894 | 2.499018 | 1.166193 | 0.939351 | 0.612434 | 0.000543 | -1.13583 |
| MB21D2 | 4.979819 | 4.960535 | 5.471019 | 2.574702 | 3.705842 | 3.4951 | 0.000554 | -0.63299 |
| ZFYVE16 | 3.395071 | 3.172915 | 3.330092 | 1.855583 | 2.612627 | 1.474638 | 0.000564 | -0.72672 |
| Homo_sapiens_newGene_13226 | 1.580193 | 0.839354 | 0.927878 | 0.574154 | 0.472801 | 0.401729 | 0.000573 | -0.84948 |
| NKX2-5 | 2.940364 | 3.006924 | 3.465864 | 1.671094 | 1.84958 | 1.882643 | 0.000582 | -0.72354 |
| ASB9 | 4.698831 | 5.98726 | 6.476386 | 3.949233 | 3.37617 | 3.117915 | 0.000616 | -0.6559 |
| TP53INP1 | 1.567057 | 1.436151 | 1.381568 | 0.902642 | 1.005605 | 0.649555 | 0.00064 | -0.7295 |
| Homo_sapiens_newGene_17135 | 2.757204 | 2.104944 | 2.138346 | 0.540363 | 1.445407 | 0.940526 | 0.000667 | -1.04969 |
| ZBTB37 | 0.696387 | 0.569985 | 0.682694 | 0.410689 | 0.271274 | 0.208923 | 0.000667 | -0.84004 |
| RAB28 | 7.413746 | 7.482098 | 6.667983 | 5.085837 | 5.793876 | 3.374165 | 0.000695 | -0.65035 |
| Homo_sapiens_newGene_14051 | 2.818861 | 1.88915 | 2.313554 | 1.640412 | 1.948629 | 1.263243 | 0.000705 | -0.95139 |
| CEP19 | 2.591156 | 2.052161 | 1.934452 | 1.085122 | 1.10534 | 1.13184 | 0.000708 | -0.80494 |
| SLC45A3 | 1.727894 | 2.046023 | 1.686153 | 0.985042 | 1.221438 | 0.969011 | 0.00071 | -0.72779 |
| CEP135 | 2.657929 | 2.273382 | 2.389698 | 1.700226 | 1.840266 | 1.108021 | 0.000719 | -0.68301 |
| CCDC68 | 0.922502 | 1.301971 | 0.863524 | 0.220685 | 0.503366 | 0.472035 | 0.000796 | -1.03923 |
| COX11 | 42.37619 | 29.61813 | 36.03726 | 34.95136 | 29.75645 | 37.11735 | 0.000796 | -0.69817 |
| 43899 | 1.182498 | 1.23217 | 1.089839 | 0.590098 | 0.651418 | 0.587007 | 0.000808 | -0.91541 |
| CHIC1 | 9.076178 | 7.532756 | 7.867629 | 5.58692 | 3.602416 | 4.487496 | 0.000814 | -0.8187 |
| SLC10A7 | 2.028228 | 2.594711 | 2.684783 | 1.926923 | 1.068753 | 0.794431 | 0.000816 | -0.80107 |
| HMGCR | 16.5069 | 14.09296 | 14.77233 | 9.021549 | 12.55303 | 8.919785 | 0.000821 | -0.5992 |
| PFKFB4 | 2.384261 | 1.845467 | 1.351516 | 1.029897 | 0.361395 | 0.696658 | 0.000917 | -1.0378 |
| MFSD2A | 3.817554 | 2.929864 | 3.360341 | 1.68604 | 2.13736 | 2.356205 | 0.000952 | -0.708 |
| C16orf87 | 5.763619 | 5.380885 | 6.387978 | 3.710232 | 4.266215 | 4.332073 | 0.000985 | -0.61751 |
| FMN2 | 0.911106 | 0.815386 | 0.75463 | 0.54991 | 0.304049 | 0.443704 | 0.000986 | -0.84205 |
| BEND6 | 2.324583 | 2.39842 | 2.061242 | 1.78402 | 1.734893 | 1.295409 | 0.001013 | -0.69409 |
| PARP9 | 4.547189 | 4.666651 | 4.807304 | 3.17813 | 3.146272 | 2.258354 | 0.001135 | -0.66544 |
| YIPF5 | 10.32119 | 10.78678 | 10.74981 | 7.099135 | 6.712959 | 3.594267 | 0.001187 | -0.77776 |
| ARL6 | 1.385421 | 1.371893 | 1.139938 | 0.840975 | 0.625606 | 0.924565 | 0.001206 | -0.73101 |
| TLDC2 | 2.951453 | 3.018793 | 3.348482 | 1.303669 | 1.755096 | 2.129372 | 0.001334 | -0.77734 |
| Homo_sapiens_newGene_18157 | 1.943382 | 1.432361 | 1.629166 | 0.88483 | 1.140884 | 0.254102 | 0.001349 | -1.14196 |
| HSPB6 | 1.213359 | 1.239312 | 0.609235 | 0.151158 | 0.414451 | 0.208195 | 0.001361 | -1.18719 |
| Homo_sapiens_newGene_16414 | 1.686787 | 1.541676 | 1.843067 | 0.959412 | 1.14256 | 0.806888 | 0.001367 | -0.79989 |
| ZBTB20 | 6.947205 | 5.080212 | 4.261765 | 4.063944 | 4.121516 | 2.776556 | 0.001377 | -0.64681 |
| FAM241B | 3.330268 | 4.464322 | 5.020137 | 2.399425 | 1.697807 | 2.398626 | 0.001543 | -0.84506 |
| DIXDC1 | 1.63589 | 1.365879 | 1.255851 | 0.933585 | 0.757004 | 0.708385 | 0.001577 | -0.63878 |
| FUT11 | 2.711235 | 3.241327 | 2.647483 | 1.647815 | 1.948699 | 1.623464 | 0.001624 | -0.6763 |
| ASB3 | 4.958206 | 5.978518 | 4.001578 | 3.178694 | 2.862237 | 3.46213 | 0.001632 | -0.6471 |
| CACNA2D4 | 0.667358 | 0.824113 | 0.843103 | 0.409167 | 0.425396 | 0.21056 | 0.001744 | -0.8835 |
| POMK | 5.378973 | 2.879235 | 2.754001 | 1.197846 | 2.036263 | 0.731901 | 0.001762 | -1.06081 |
| F8A3 | 1.644137 | 4.331861 | 2.716994 | 1.492515 | 0.276116 | 0.481419 | 0.001863 | -1.15918 |
| TLR6 | 3.584819 | 4.208206 | 2.922797 | 2.446754 | 2.012676 | 1.393408 | 0.001873 | -0.99276 |
| DDHD2 | 4.378878 | 4.145189 | 4.069481 | 2.811997 | 2.739038 | 2.878735 | 0.001925 | -0.61843 |
| MECP2 | 20.40112 | 16.93138 | 10.31343 | 12.5604 | 28.66882 | 17.37929 | 0.002083 | -0.62645 |
| ERAP2 | 4.461188 | 3.684026 | 4.752633 | 3.172047 | 3.627136 | 3.610182 | 0.002169 | -0.64178 |
| DFFB | 1.0951 | 1.036666 | 1.337874 | 0.651726 | 0.430252 | 0.568617 | 0.002238 | -0.85016 |
| FER1L6 | 0.393484 | 0.445076 | 0.192419 | 0.139833 | 0.068398 | 0.018049 | 0.002244 | -1.16073 |
| SATB2 | 2.134646 | 2.314922 | 2.406919 | 1.508137 | 1.349354 | 1.060909 | 0.002267 | -0.61771 |
| TNFAIP8 | 6.871059 | 3.62023 | 6.202576 | 3.231039 | 3.691074 | 2.31925 | 0.002553 | -0.7425 |
| TSPYL4 | 1.709124 | 1.600488 | 1.801034 | 1.065735 | 0.985772 | 1.242843 | 0.002636 | -0.60215 |
| EFEMP1 | 3.275656 | 2.720255 | 3.343313 | 1.498781 | 1.86399 | 2.232671 | 0.002873 | -0.63689 |
| ZNF711 | 1.229855 | 1.526063 | 0.997116 | 0.609294 | 0.464909 | 0.576671 | 0.003055 | -0.80058 |
| RHEBL1 | 0.740553 | 1.253153 | 0.754189 | 0.179674 | 0.340364 | 0.258733 | 0.003142 | -1.09408 |
| TNS4 | 1.540211 | 1.36955 | 1.407835 | 0.707241 | 0.909327 | 0.710167 | 0.003197 | -0.67007 |
| DENND2C | 0.453597 | 0.400786 | 0.459166 | 0.12306 | 0.243741 | 0.229054 | 0.003234 | -0.91513 |
| Homo_sapiens_newGene_6435 | 1.187504 | 0.823004 | 0.944247 | 0.466384 | 0.637231 | 0.527613 | 0.003237 | -0.81275 |
| SLC14A1 | 13.01935 | 16.80068 | 14.20577 | 7.542338 | 12.30925 | 9.654557 | 0.003389 | -0.66442 |
| CHN1 | 1.79412 | 1.255068 | 1.632086 | 0.428952 | 0.923627 | 1.048422 | 0.003397 | -0.88714 |
| GRPR | 1.687111 | 1.335784 | 1.736475 | 0.813724 | 0.974198 | 0.633228 | 0.003524 | -0.82522 |
| Homo_sapiens_newGene_16783 | 3.324822 | 2.706417 | 2.184516 | 0.68278 | 3.027366 | 0.862318 | 0.00354 | -0.96731 |
| NUBPL | 1.660597 | 1.905453 | 1.749792 | 1.252279 | 0.819859 | 0.889898 | 0.003612 | -0.70491 |
| TMOD1 | 2.818585 | 1.459563 | 2.286288 | 1.311257 | 1.018505 | 1.208572 | 0.003755 | -0.7794 |
| UBE2A | 29.02663 | 71.74635 | 71.21591 | 28.05149 | 29.36925 | 30.69785 | 0.003765 | -0.74548 |
| PLA2R1 | 0.407507 | 0.435586 | 0.34592 | 0.261356 | 0.235616 | 0.244798 | 0.003776 | -0.62716 |
| EDN2 | 1.333656 | 1.397974 | 0.961695 | 0.389583 | 0.284718 | 0.61461 | 0.003883 | -1.02844 |
| Homo_sapiens_newGene_11003 | 1.91423 | 2.780488 | 2.202742 | 0.974824 | 1.430323 | 1.422407 | 0.003922 | -0.74939 |
| LYRM7 | 1.7697 | 1.327915 | 1.214765 | 0.728147 | 0.829393 | 0.783485 | 0.00393 | -0.78998 |
| RNF144A | 1.465359 | 1.128214 | 0.999325 | 0.711674 | 0.794531 | 0.785569 | 0.004034 | -0.64977 |
| PLPP6 | 1.018892 | 0.722138 | 0.783054 | 0.366829 | 0.504012 | 0.316805 | 0.004177 | -0.88572 |
| MAP3K8 | 4.247634 | 4.292259 | 3.943786 | 2.931032 | 2.675319 | 2.229954 | 0.004195 | -0.6243 |
| PPP6C | 18.1841 | 19.93534 | 19.98051 | 8.113862 | 13.83723 | 14.38045 | 0.00438 | -0.6319 |
| DDHD1 | 1.280379 | 0.468259 | 0.968002 | 0.69942 | 0.344076 | 0.336249 | 0.004593 | -0.63121 |
| TSC22D3 | 4.797785 | 5.216566 | 6.553484 | 3.136087 | 3.300617 | 4.127657 | 0.004689 | -0.61403 |
| TTBK2 | 1.212823 | 0.776908 | 0.89249 | 0.309504 | 0.46478 | 0.695803 | 0.004833 | -0.65104 |
| NREP | 1.275785 | 1.636268 | 1.234138 | 0.997482 | 0.716015 | 0.690854 | 0.005395 | -0.78146 |
| ENO2 | 10.11191 | 8.447873 | 7.223637 | 3.952365 | 6.309129 | 5.5904 | 0.006096 | -0.65079 |
| ZNF566 | 1.983824 | 2.747336 | 2.384424 | 2.200772 | 1.56676 | 1.509011 | 0.006154 | -0.60976 |
| ZNF460 | 7.222646 | 5.51512 | 2.858675 | 2.548243 | 3.397724 | 1.766231 | 0.006694 | -0.79759 |
| DGKH | 2.655076 | 2.251328 | 1.776798 | 1.013777 | 1.204733 | 1.164391 | 0.006756 | -0.80673 |
| IL1B | 2.807383 | 2.875452 | 3.342788 | 2.249478 | 1.902454 | 1.594713 | 0.006825 | -0.65112 |
| IL1A | 1.744222 | 1.131654 | 1.423863 | 0.944682 | 0.58479 | 0.611062 | 0.006864 | -0.82952 |
| Homo_sapiens_newGene_9381 | 0.846686 | 0.957527 | 0.634182 | 0.499923 | 0.459259 | 0.344579 | 0.006883 | -0.77042 |
| MUC5AC | 5.428519 | 4.14587 | 3.554224 | 2.640128 | 3.5578 | 1.774304 | 0.006904 | -0.66068 |
| TET2 | 1.574372 | 1.413961 | 1.318494 | 0.800504 | 1.266363 | 0.639523 | 0.006918 | -0.64789 |
| KIAA1324 | 0.595512 | 0.555009 | 0.74921 | 0.299403 | 0.356262 | 0.228964 | 0.006942 | -0.85062 |
| PGAP1 | 0.633873 | 0.731709 | 0.548574 | 0.390481 | 0.563801 | 0.242551 | 0.007413 | -0.65626 |
| GYG2 | 2.230565 | 2.034378 | 2.082052 | 1.60714 | 0.993089 | 0.850894 | 0.007979 | -0.75413 |
| NCR3LG1 | 2.943362 | 3.16244 | 2.291885 | 1.765037 | 2.235364 | 1.227447 | 0.007995 | -0.63235 |
| CYP4V2 | 3.626261 | 2.926301 | 3.21031 | 2.061473 | 2.436426 | 1.427779 | 0.008508 | -0.65562 |
| HNRNPUL2-BSCL2 | 0.734284 | 1.954682 | 1.33757 | 0.709124 | 0.539421 | 0.66528 | 0.008636 | -0.84929 |
| SMIM12 | 12.3479 | 13.21262 | 6.555788 | 5.95523 | 6.623606 | 8.304491 | 0.008832 | -0.77113 |
| Homo_sapiens_newGene_18015 | 2.412153 | 2.981985 | 2.744823 | 1.850464 | 1.315056 | 1.050363 | 0.00896 | -0.7704 |
| Homo_sapiens_newGene_3473 | 1.769805 | 1.728875 | 1.434468 | 0.463312 | 0.897298 | 0.871072 | 0.009053 | -0.7624 |
| ZBED6 | 2.872209 | 6.890346 | 3.334087 | 1.363568 | 2.252321 | 2.947912 | 0.009146 | -0.7048 |
| RASEF | 0.98291 | 1.428639 | 1.071277 | 0.706203 | 0.901917 | 0.65385 | 0.00988 | -0.6084 |
| SMIM8 | 1.762505 | 1.978571 | 2.059238 | 1.499622 | 1.21533 | 1.088222 | 0.010509 | -0.62313 |
| M6PR | 112.4689 | 74.20011 | 70.68014 | 27.16177 | 42.00726 | 40.54833 | 0.010869 | -0.91069 |
| BBIP1 | 5.407204 | 7.08917 | 6.854933 | 5.619515 | 4.253052 | 4.423478 | 0.011585 | -0.62138 |
| PCDHB2 | 1.070381 | 2.317392 | 1.995474 | 0.970748 | 1.098357 | 0.784126 | 0.012749 | -0.81109 |
| HECTD2 | 1.562695 | 1.896903 | 1.106978 | 1.096846 | 1.4231 | 0.761871 | 0.013414 | -0.69446 |
| MAP3K14 | 3.932573 | 3.523592 | 4.903115 | 1.515466 | 3.402665 | 3.252134 | 0.014045 | -0.64355 |
| Homo_sapiens_newGene_9941 | 0.921718 | 2.215119 | 1.62058 | 1.002614 | 0.483116 | 0.507041 | 0.014188 | -0.78652 |
| DCUN1D4 | 5.266047 | 5.963024 | 9.093846 | 3.51441 | 5.577426 | 3.878752 | 0.014665 | -0.71814 |
| ABAT | 0.228322 | 0.345407 | 0.288923 | 0.110843 | 0.087918 | 0.131753 | 0.014665 | -0.89257 |
| Homo_sapiens_newGene_2442 | 2.110237 | 2.762366 | 0.972547 | 1.675915 | 1.556522 | 0.641263 | 0.014848 | -0.77611 |
| HRH1 | 7.784245 | 8.234079 | 10.63142 | 5.170688 | 5.990987 | 7.142766 | 0.01507 | -0.58571 |
| SYDE2 | 0.46687 | 0.52571 | 0.459329 | 0.309704 | 0.300218 | 0.210399 | 0.015517 | -0.70728 |
| SOST | 0.630854 | 0.382971 | 0.433922 | 0.09892 | 0.258917 | 0.115611 | 0.016182 | -0.93595 |
| Homo_sapiens_newGene_2565 | 0.789087 | 0.92248 | 0.830477 | 0.372233 | 0.286051 | 0.350589 | 0.016362 | -0.8961 |
| RASSF5 | 0.360862 | 0.376555 | 0.216683 | 0 | 0.053304 | 0.069174 | 0.016431 | -0.94195 |
| RRAD | 0.713031 | 0.599678 | 0.691778 | 0.182444 | 0.341339 | 0.269868 | 0.016443 | -0.89882 |
| LYNX1 | 0.654522 | 0.679714 | 0.739687 | 0.272496 | 0.315758 | 0.50724 | 0.016934 | -0.7438 |
| KIF17 | 0.629933 | 0.765524 | 0.669526 | 0.306566 | 0.458644 | 0.32317 | 0.017499 | -0.7283 |
| SHANK2 | 0.157632 | 0.204793 | 0.09115 | 0.046093 | 0.061733 | 0.166158 | 0.017888 | -0.87727 |
| GPR75 | 1.908388 | 1.739144 | 1.630834 | 1.351283 | 0.970303 | 0.685149 | 0.018746 | -0.69704 |
| WSCD1 | 1.26576 | 0.622022 | 0.847066 | 0.524002 | 0.827925 | 0.578168 | 0.018768 | -0.70315 |
| ANGPTL4 | 3.052991 | 2.461826 | 2.65051 | 2.191522 | 1.712359 | 1.759507 | 0.018986 | -0.59642 |
| Homo_sapiens_newGene_14605 | 0.869991 | 0.774893 | 0.713277 | 0.472717 | 0.400777 | 0.48765 | 0.019115 | -0.67546 |
| ZNF461 | 2.379655 | 2.625006 | 3.774701 | 1.681068 | 2.008917 | 1.682485 | 0.019388 | -0.64176 |
| PITPNB | 32.90992 | 15.88405 | 14.86014 | 12.90569 | 13.70922 | 12.87924 | 0.019873 | -0.63896 |
| HNMT | 2.638179 | 2.75444 | 2.812864 | 2.915529 | 2.138586 | 2.177175 | 0.019897 | -0.65035 |
| KIF21B | 0.325556 | 0.268607 | 0.295011 | 0.183007 | 0.209777 | 0.135122 | 0.019899 | -0.66588 |
| STOX1 | 0.613619 | 0.65969 | 0.477787 | 0.398221 | 0.175695 | 0.187602 | 0.019961 | -0.91499 |
| TBC1D2 | 1.946001 | 1.281174 | 1.810924 | 1.168239 | 1.154764 | 0.846412 | 0.020377 | -0.59363 |
| GCNT4 | 0.386455 | 0.318465 | 0.241211 | 0.080359 | 0.192928 | 0.143289 | 0.020381 | -0.84471 |
| DCTN4 | 13.30918 | 6.708763 | 6.583013 | 5.243724 | 5.70718 | 5.400737 | 0.020405 | -0.61502 |
| ULBP3 | 4.530145 | 5.558226 | 3.476562 | 1.928683 | 3.005068 | 2.748092 | 0.021057 | -0.69143 |
| 43894 | 0.894087 | 0.583579 | 0.751496 | 0.33517 | 0.506814 | 0.429774 | 0.021218 | -0.68629 |
| ATP11A | 5.339116 | 5.35254 | 4.277499 | 3.630235 | 3.972212 | 2.708522 | 0.021699 | -0.60956 |
| C6orf223 | 0.412533 | 0.428406 | 0.401765 | 0.171172 | 0.165642 | 0.265263 | 0.022378 | -0.79502 |
| ELK3 | 8.81044 | 8.203709 | 6.576641 | 3.366344 | 6.030389 | 4.655154 | 0.023342 | -0.62565 |
| HOXB4 | 0.287639 | 0.341692 | 0.367469 | 0.08286 | 0.164107 | 0.175745 | 0.023588 | -0.84724 |
| SERPINB2 | 0.999767 | 0.647586 | 0.553644 | 0.052788 | 0.378952 | 0.217936 | 0.024046 | -0.89983 |
| PRDM16 | 0.219762 | 0.158797 | 0.190799 | 0.057798 | 0.088188 | 0.095353 | 0.024836 | -0.78897 |
| KLHL3 | 0.91225 | 0.733554 | 0.449753 | 0.414426 | 0.322249 | 0.444291 | 0.026264 | -0.68041 |
| TRIM16 | 65.47371 | 101.0562 | 69.66636 | 29.66058 | 44.99923 | 55.93444 | 0.028376 | -0.80999 |
| Homo_sapiens_newGene_4091 | 3.723103 | 3.530701 | 2.767599 | 1.864516 | 2.842361 | 2.271306 | 0.028836 | -0.59181 |
| CASP2 | 12.35615 | 11.30945 | 10.14302 | 6.077652 | 5.675148 | 2.431811 | 0.029245 | -0.84768 |
| TTLL7 | 0.626726 | 0.611691 | 0.448822 | 0.352627 | 0.421072 | 0.287586 | 0.029391 | -0.60461 |
| Homo_sapiens_newGene_4037 | 1.259538 | 1.256672 | 0.915253 | 0.493738 | 0.648507 | 0.826301 | 0.031301 | -0.68608 |
| SLC16A2 | 0.744281 | 0.786228 | 0.622309 | 0.365668 | 0.419714 | 0.232291 | 0.03273 | -0.70441 |
| CLDN12 | 5.693017 | 9.111269 | 4.640477 | 3.84175 | 4.32201 | 4.182759 | 0.032983 | -0.60474 |
| SRSF12 | 0.554057 | 0.419128 | 0.492656 | 0.309509 | 0.161193 | 0.137455 | 0.035683 | -0.76059 |
| ELF4 | 19.09968 | 17.10438 | 37.5084 | 15.30848 | 11.249 | 8.603636 | 0.035738 | -0.77467 |
| IGFBP1 | 5.227748 | 4.880016 | 9.899646 | 1.743768 | 4.576859 | 4.132454 | 0.035962 | -0.72465 |
| PDP2 | 2.947326 | 3.161161 | 3.67897 | 1.378737 | 1.118173 | 1.190587 | 0.037723 | -0.83025 |
| HYAL1 | 0.647562 | 1.440799 | 0.799953 | 0.72647 | 0.55379 | 0.454863 | 0.040701 | -0.71927 |
| DRAM1 | 2.583682 | 3.691201 | 2.840002 | 2.030944 | 2.77806 | 1.632382 | 0.044347 | -0.66013 |
| MTERF1 | 3.542802 | 7.794738 | 5.99038 | 2.964223 | 4.252183 | 4.056907 | 0.044493 | -0.63431 |
| Homo_sapiens_newGene_19415 | 1.872375 | 1.939341 | 1.076982 | 0.952992 | 0.819049 | 0.811055 | 0.044626 | -0.61762 |
| OTUB2 | 0.87187 | 0.592072 | 0.642193 | 0.52436 | 0.429121 | 0.311721 | 0.044937 | -0.65597 |
| ASAH2 | 0.35285 | 1.679346 | 0.489389 | 0.242084 | 0.211775 | 0.864112 | 0.045527 | -0.61923 |
| Homo_sapiens_newGene_1788 | 0.451422 | 0.3131 | 0.393857 | 0.218804 | 0.124978 | 0.201038 | 0.047937 | -0.75819 |
| APOLD1 | 3.775425 | 0.292918 | 11.72284 | 0.258494 | 0.822367 | 0.304073 | 0.048398 | -0.70976 |
