# Supplementary material for: RBM15 promotes hepatocellular carcinoma progression by regulating N6-methyladenosine modification of YES1 mRNA in an IGF2BP1-dependent manner
Source: Cell Death Discov. 2021 Oct 27;7:315. doi: 10.1038/s41420-021-00703-w (PMC8551180; doi:10.1038/s41420-021-00703-w)
Supplement: Supplementary file 16 — STR for cells [file 41420_2021_703_MOESM16_ESM.docx]

## Here, we present the STR certificates for Huh7 (page 2-4), HCCLM3 (page 5-7) and MHCC97H (page 8-17) cell lines, respectively.

CH INA CENTER FOR TYI°E CULTURE COLLECTION (CCTCC)

Wu han University, Wu han 430072, China

Ph o we: 86-027-68752093

Fax: 86-027-G8754833 Email: [slaenchao@whu.edu](mailto:slaenchao@whu.edu) cn

.. .... . . . . .

05-21-2019

Entrusted by Zhejiang University, CCTCC has conducted identification experiments on the Huh-7 cell line, and come to the following conclusions:

1. There was no third allele found in Huh-7 cell line, it indicating that there was no cross-contaminant of human source cell line.
2.
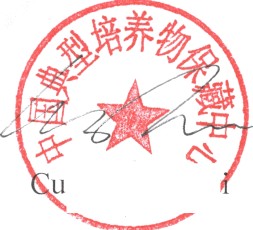
Compared the STR data of Huh-7 cell line in the databases of ATCC and DSMZ, all the locl of Huh-7 were exactly matched with the loci of HuH-7 (JCRB0403) cells found in DSMZ cell bank, so it is HuH-7 (JCRB0403) cell line (Table 1).

Manager:

China Center for Type l “‘ on

Note:

1. The result is only responsible for the test salrple, and the genomic DNA will be reserved for three month.
2. Reference of human cell line authentication: ANS I/ATCC ASN-0002-20 11

Table1. The alleles of 21 locations in Huh-7 cell line

| Huh-7 cell line（Fig. No. XB19-744） | | |
| --- | --- | --- |
| Marker | Allele 1 | Allele 2 |
| D19S433 | 13 | 14 |
| **D5S818** | **12** | **12** |
| D21S11 | 30 | 30 |
| D18S51 | 15 | 15 |
| D6S1043 | 13 | 15 |
| **AMEL** | **X** | **X** |
| D3S1358 | 15 | 15 |
| **D13S317** | **10** | **11** |
| **D7S820** | **11** | **11** |
| **D16S539** | **10** | **10** |
| **CSF1PO** | **11** | **11** |
| Penta D | 12 | 12 |
| D2S441 | 12 | 14 |
| **vWA** | **16** | **18** |
| D8S1179 | 14 | 14 |
| **TPOX** | **8** | **11** |
| Penta E | 11 | 11 |
| **TH01** | **7** | **7** |
| D12S391 | 20 | 21 |
| D2S1338 | 19 | 19 |
| FGA | 22 | 23 |

Sa mpl e Ha me

X B19-744

Panel

MR21_v2.0

S Q O S O S S Q S SPK MIX O MR . C G Q

HsrA Seple for Deleton

|  |  |  |  |  |  |  |  |  |
| --- | --- | --- | --- | --- | --- | --- | --- | --- |

60

1200

100

140

180

220

260

300

340

300

d20

d8O

1000

800

600

400

200

0

: -- - " MR21_v2.0

30 l5

30 IJ

barb S ampl e for 0 e1 et i on

60

5000

4000

3000

2000

1000

0

X B19-T44

60

1600

1200

800

OOO

” MR21_v2.0

100

100

|  |  |  |  |  |  |  |
| --- | --- | --- | --- | --- | --- | --- |

140

IJ

140

180

180

220

II II

220

260

10

10

260

300

300

340

11

340

300

380

d20

d20

d8O

#erd Semple Cor Deletion

d80

0

X B19-744

MR21_v2.0

#srd Sample for Deleti on

|  |  |  |  | OJ |  |
| --- | --- | --- | --- | --- | --- |

60

3000 dOOO 3000

2000

100

140

180

220

260

300

3dO

380

d20

d8O

1000


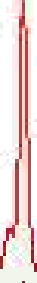

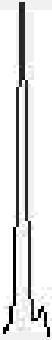

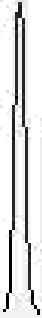

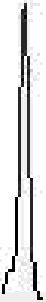

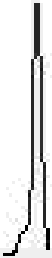

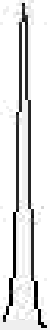

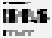

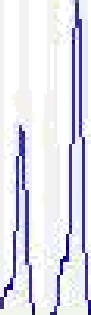

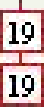

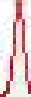

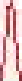

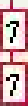

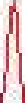

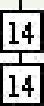

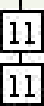

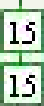

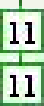

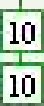

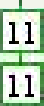

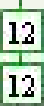

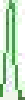

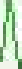

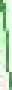

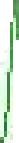

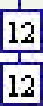

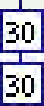

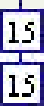

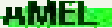

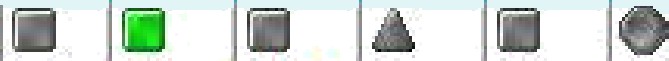

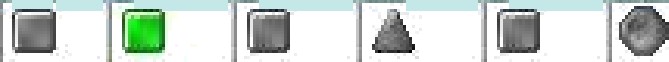

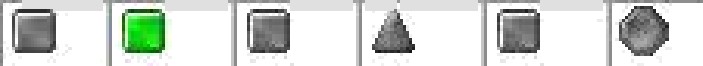

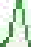

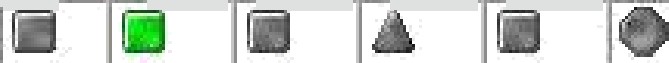

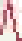

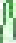

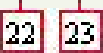

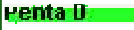

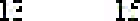

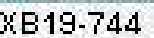

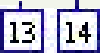

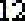

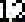

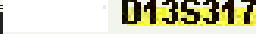

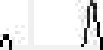

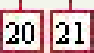

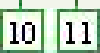

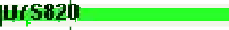

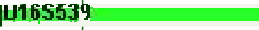

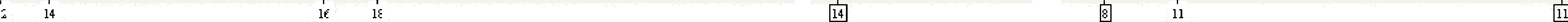

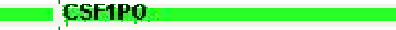

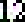

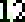


0

19 4

19

‹P @ iâi iIi 1“» N 4S lik ifi 't• 'é'

CH INA CENTElI FOR TY PU CULT U UE COLL ECT I ON (CCTCC)

¥V' u li a n U nivc rs ity, Yt' It li 9 In 4301172, C li ink

I•lione: SP-027-fa8752(J93
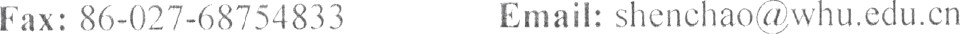


05-23-2019

Entrusted by Zhejiang UniVelsity, CCTCC has conducted identification experiments on the HCC-LM3 cell line, and come to the following conclusions:

1. There was no thii d allele found in HCC-LM3 cell line, it indicating

that there was no cross-contaminant of human source cell line.

1. Compared the STR data of HCC-LM3 cell line in the databases of ATCC and DSMZ, its profile does not exactly match with any of the crlFl ent data (Table 1).
2. 1 he STR data of BICC-LM3 cell line and NCI-hI82 (HTB- 175) cell

line hatches the highest rate of 73% in ATCC database.


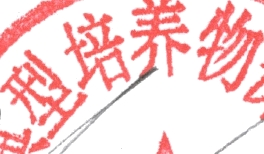

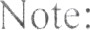
Manager : China Center fo


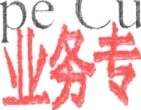


e u

11 CC I l OU

I . T fee result is on ly responsib e or die test sample, and the genomic DNA will be reserved for three naontla.

2. Reference of human cell line authentication: ANS1/ATCC A SN-0002-201 1

Table1. The alleles of 21 locations in HCC-LM3 cell line

| HCC-LM3 cell line （Fig. No. XB XB19-750） | | |
| --- | --- | --- |
| Marker | Allele 1 | Allele 2 |
| D19S433 | 13 | 14 |
| **D5S818** | **12** | **13** |
| D21S11 | 31.2 | 31.2 |
| D18S51 | 13 | 22 |
| D6S1043 | 12 | 20 |
| **AMEL** | **X** | **Y** |
| D3S1358 | 15 | 16 |
| **D13S317** | **8** | **8** |
| **D7S820** | **10** | **10** |
| **D16S539** | **12** | **12** |
| **CSF1PO** | **11** | **13** |
| Penta D | 8 | 9 |
| D2S441 | 15 | 15 |
| **vWA** | **14** | **14** |
| D8S1179 | 12 | 13 |
| **TPOX** | **8** | **8** |
| Penta E | 17 | 17 |
| **TH01** | **9** | **9** |
| D12S391 | 18 | 18 |
| D2S1338 | 20 | 20 |
| FGA | 21 | 24 |

Sa mpl e Na me

X B19-ISO

Panel

MR21_v2.0

S Q O S O S S Q S SPK MI X O MR C G Q

#mrl Semple for Deletion

|  |  |  |  |  |  |  |
| --- | --- | --- | --- | --- | --- | --- |

60

800

600

OOO

200

100

140

180

220

260

300

3dO

380

d20

d80

0

X B19-750

MR21_v2.0

àtarÌ+ S aatpt e for D e f e t i ozt

|  | 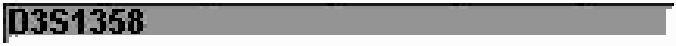 |  |  |  |  |  |  |  |
| --- | --- | --- | --- | --- | --- | --- | --- | --- |

60 100

1d0

180

220

260

300

3dO

380

d20

dd0

1600

1200

800

QOO

0

8 10 12

10

X B19-750

| 60 | 100 |  | 140 | 180 | 220 | 260 |  | 300 | 340 | 300 | d20 | d80 |
| --- | --- | --- | --- | --- | --- | --- | --- | --- | --- | --- | --- | --- |
| 2000 |  |  |  |  |  |  |  |  |  |  |  |  |
| 1600 |  |  |  |  |  |  |  |  |  |  |  |  |
| 1200 |  |  |  |  |  |  |  |  |  |  |  |  |
| 800 |  |  |  |  |  |  |  |  |  |  |  |  |
| 400 |  |  |  |  |  |  |  |  |  |  |  |  |
| 0 |  |  |  |  |  |  | 8 |  |  | l7 |  |  |
|  | 1J | 14 |  |  |  |  | 8 |  |  | l7 |  |  |

X B19-750

MR21_v2.0


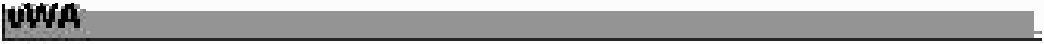
MR21_v2.0


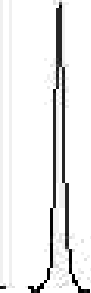

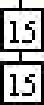

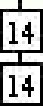

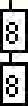

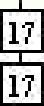

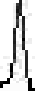

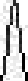

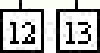

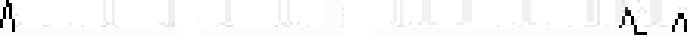


|  |  |  |  |  |  |  |
| --- | --- | --- | --- | --- | --- | --- |

Bari 5 8apI e for D e f e t 1 ozt

filari S aatpt e for D ed e t i ozt

|  |  |  | 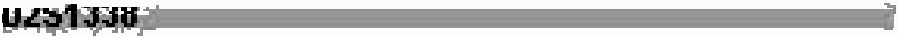 |  |  |
| --- | --- | --- | --- | --- | --- |

60 100

140

180

220

260

300

340

380

420 %0


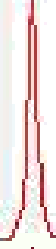

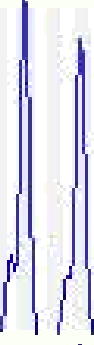

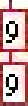

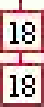

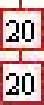

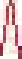

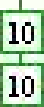

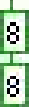

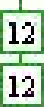

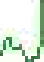

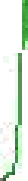

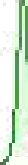

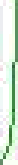

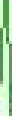

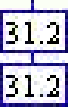

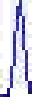

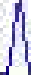

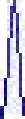

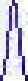

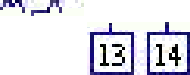

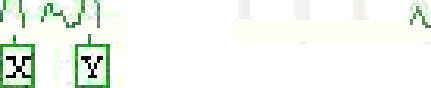

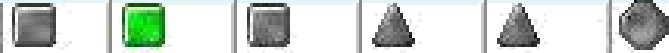

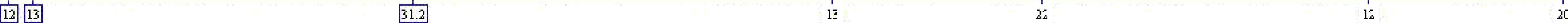

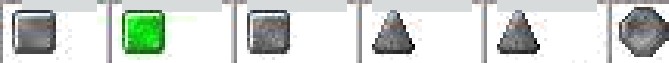

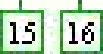

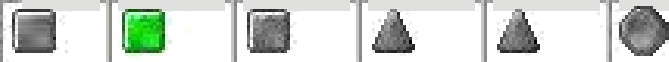

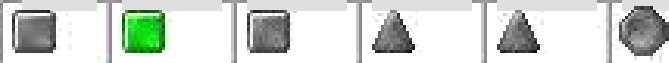

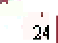

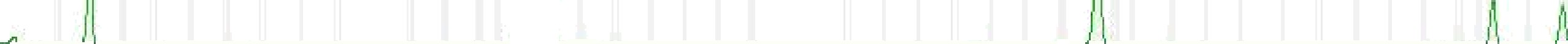

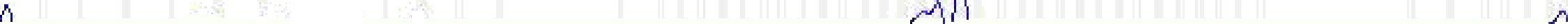

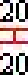


2000

1600

1200

800

400

0

9 18

9 18

7 21

**细胞遗传质量鉴定检测**

**Cell Line Authentication Service**

**STR 基因型检测报告**

# 样品信息

**样品编号：**

| 客户样本编号 | 公司编号 |
| --- | --- |
| MHCC-97H | 20190409-01 |

**样品数量：**1

**样品性状：**细胞系**检测项目：**STR

**检测方法：**用 Axygen 的基因组抽提试剂盒提取 DNA,采用 20- STR 扩增方案扩增,在ABI 3730XL 型遗传分析仪上对 STR 位点和性别基因 Amelogenin 进行检测。

# 样品信息

**（一）检验基本情况**

| 公司编号 | 多等位基因 | 匹配细胞系 | 细胞库 | EV 值 | 匹配说明 |
| --- | --- | --- | --- | --- | --- |
| 20190409-01 | 无 | - | DSMZ | - | 无匹配 |

样本基因型检验结果

- 多等位基因指三等位及以上基因现象。
- 本次检测各细胞分型结果良好。

**（二）各样本描述**

- 20190409-01：该株细胞 DNA 分型在细胞系检索中**没有找到匹配**的细胞系，本次检测在该细胞系中没有**发现多等位基因**。（该细胞系未发现多等位基因、未发现交叉污染，细胞系无异常，因数据库未收录 MHCC-97H 相关 STR 信息无法匹配，若为发表论文用，提交该数据给杂志即可）

**备注**：待测细胞系与收录于 ATCC，DSMZ，JCRB 和 RIKEN 数据库的细胞系 STR 数据进行比对，未收录于以上细胞库的细胞系将无法匹配。

**（三）样本分析结果**

| **细胞 20190409-01 的 STR 位点和 Amelogenin 位点的基因分型结果** | | | | | | |
| --- | --- | --- | --- | --- | --- | --- |
| Loci | 送检细胞 STR 信息 | | | 细胞库细胞 STR 信息 | | |
|  | 送检细胞名：MHCC-97H | | | 细胞库细胞名： | | |
|  | Allele1 | Allele2 | Allele3 | Allele1 | Allele2 | Allele3 |
| D5S818 | 12 | 13 |  |  |  |  |
| D13S317 | 8 | 8 |  |  |  |  |
| D7S820 | 10 | 10 |  |  |  |  |
| D16S539 | 12 | 12 |  |  |  |  |

| VWA | 14 | 14 |  |  |  |  |
| --- | --- | --- | --- | --- | --- | --- |
| TH01 | 9 | 9 |  |  |  |  |
| AMEL | X | Y |  |  |  |  |
| TPOX | 8 | 8 |  |  |  |  |
| CSF1PO | 11 | 13 |  |  |  |  |
| D12S391 | 18 | 18 |  |  |  |  |
| FGA | 21 | 24 |  |  |  |  |
| D2S1338 | 20 | 20 |  |  |  |  |
| D21S11 | 31.2 | 31.2 |  |  |  |  |
| D18S51 | 13 | 22 |  |  |  |  |
| D8S1179 | 12 | 13 |  |  |  |  |
| D3S1358 | 15 | 16 |  |  |  |  |
| D6S1043 | 12 | 20 |  |  |  |  |
| PENTAE | 11 | 17 |  |  |  |  |
| D19S433 | 13 | 14 |  |  |  |  |
| PENTAD | 8 | 9 |  |  |  |  |

# 其他说明

**（一）分型方案及位点信息**

|  | **方案 1** | **方案 2** | **方案 3** | **方案 4** |
| --- | --- | --- | --- | --- |
| 1 | TH01 | TPOX | D3S1358 | AMEL |
| 2 | D12S391 | VWA | D13S317 | D5S818 |

| 3 | D7S820 | D8S1179 | D6S1043 | D2S1338 |
| --- | --- | --- | --- | --- |
| 4 | CSF1PO | PENTAD | D16S539 | D21S11 |
| 5 | FGA |  | D19S433 | D18S51 |
| 6 | PENTAE |  |  |  |

实验方案及位点

**（二）STR 数据库比对**

本公司采用 DSMZ tools 进行细胞系比对,其中包含来自于 ATCC，DSMZ，JCRB 和 RIKEN 数据库的 2455 个细胞系 STR 数据。如果待检测细胞未收录于以上细胞库或这是自行建立的新细胞系将无法进行比对，用户需根据细胞分型结果自行与其他数据库进行比对。

**签发日期：2019-04-24**
